# Nickel-catalyzed C–H/N–H annulation of aromatic amides with alkynes in the absence of a specific chelation system†
†Electronic supplementary information (ESI) available. See DOI: 10.1039/c7sc01750b



**DOI:** 10.1039/c7sc01750b

**Published:** 2017-07-24

**Authors:** Atsushi Obata, Yusuke Ano, Naoto Chatani

**Affiliations:** a Department of Applied Chemistry , Faculty of Engineering , Osaka University , Suita , Osaka 565-0871 , Japan

## Abstract

The Ni-catalyzed reaction of aromatic amides with alkynes in the presence of KOBu^*t*^ involves C–H/N–H oxidative annulation to give 1(2*H*)-isoquinolinones. A key to the success of the reaction is the use of a catalytic amount of strong base, such as KOBu^*t*^. The reaction shows a high functional group compatibility. The reaction with unsymmetrical alkynes, such as 1-arylalkynes, gives the corresponding 1(2*H*)-isoquinolinones with a high level of regioselectivity. This discovery would lead to the development of Ni-catalyzed chelation-assisted C–H functionalization reactions without the need for a specific chelation system.

## Abstract 2

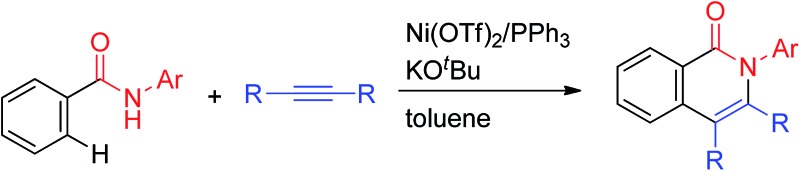
We report the development of a new system for C–H functionalizations catalyzed by nickel complexes. The findings show that aromatic amides with a simple directing group can participate in Ni-catalyzed C–H functionalization.

## Introduction

**Scheme 1:**
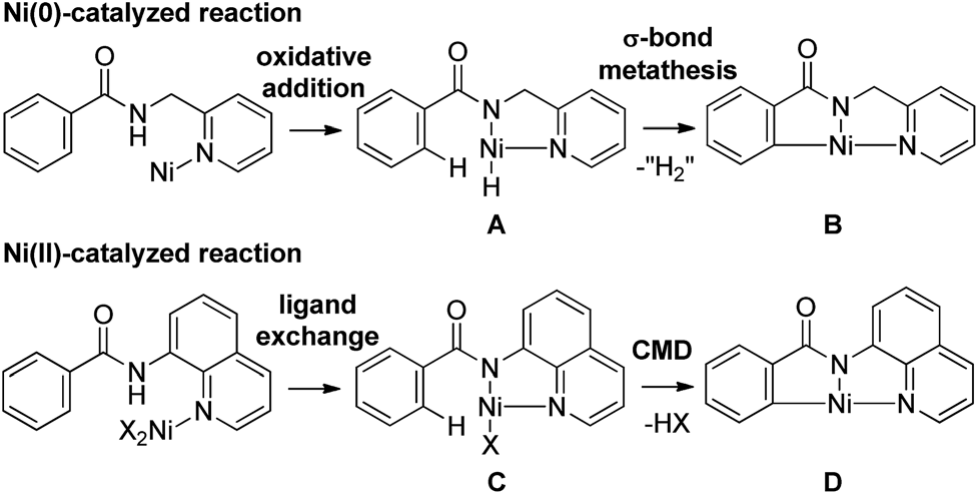
Key steps for the activation of C–H bonds by nickel complexes in a bidentate chelation system. For simplicity, groups that are not involved in the transformation, such as ligands and reagents, are omitted.

The direct functionalization of C–H bonds has emerged as an increasingly valuable tool for step-economical organic synthesis. A wide variety of transition metal complexes, including Pd, Ru, Rh and Ir, can be used as catalysts in a variety of catalytic functionalizations of C–H bonds.^1^ Nickel-catalyzed C–H functionalizations have recently become a subject of great interest, owing to the low cost of the reaction, the use of readily available nickel and the uniqueness of the reaction. However, the functionalization of C–H bonds catalyzed by Ni complexes was limited to C–H bonds in specific aromatic systems, such as pyridine or activated pyridine derivatives, perfluorinated benzene, azoles and indoles, all of which contain an acidic C–H bond.^2^ No general and reliable system for the nickel-catalyzed functionalization of non-acidic C–H bonds in benzene rings was available.^3^ In 2011, we reported the Ni(0)-catalyzed reaction of aromatic amides that contain a 2-pyridinylmethylamine moiety as a directing group, with alkynes, leading to the production of isoquinolinones.^4^ In 2013, we also reported the Ni(ii)-catalyzed C–H alkylation of aromatic amides that contain an 8-aminoquinoline moiety as a directing group, with alkyl halides.^5*a*^ Since then, significant advances in Ni(ii)-catalyzed C–H functionalization reactions that involve the use of a bidentate chelation system have been reported by many other groups, as well as our group.^5–7^ A newly developed chelation system, in which a bidentate directing group is used, is now recognized as a powerful and reliable strategy for developing Ni-catalyzed C–H functionalizations. The presence of both N(sp^2^) and NH groups is crucial for the reaction to proceed. The reactions reported to date can be classified into two types, depending on the oxidation state of the key catalytic species (Scheme 1). In the case of a Ni(0)-catalyzed system, the coordination of an N(sp^2^) atom to the nickel(0) center, followed by oxidative addition of an N–H bond, gives complex **A**.^8,9^ Subsequently, the cleavage of a C–H bond proceeds through σ-bond metathesis to generate the cyclometalated complex, **B**, with concomitant generation of formal “H_2_”, which is then trapped by a hydrogen acceptor such as an alkyne. In the Ni(ii)-catalyzed system, the coordination of an N(sp^2^) atom to a nickel(ii) center followed by ligand exchange gives complex **C**, which, after the cleavage of a C–H bond through a concerted metalation deprotonation mechanism (CMD), generates cyclometalated complex **D**. After metalacycle **B** or **D** is formed, various reagents can then be used in the remainder of the reaction. Irrespective of the mechanism, the role of the N(sp^2^) atom is to bring the nickel catalyst into close proximity of the NH bond by coordination, followed by the formation of a covalent N–Ni bond *via* oxidative addition or ligand exchange. The nickel atom in the resulting intermediates, **A** and **C**, is now sufficiently close to activate the *ortho* C–H bonds, which are then cleaved. Thus, the formation of a covalent N–Ni bond is a key step in the activation of C–H bonds in the Ni-catalyzed bidentate chelation system. In the case of Pd, Ru, Rh and Ir-catalyzed reactions, a wide variety of chelating groups are known to function as directing groups that are capable of activating C–H bonds. Even the weak coordination of a heteroatom to these metals can promote the activation of C–H bonds. In sharp contrast, when the heteroatom is weakly coordinated to nickel, the C–H bonds are not activated. Because of this, the pre-coordination of an N(sp^2^) atom to the nickel center is required to allow the nickel to come into close proximity of the N–H bonds in order to form an N–Ni bond.

**Scheme 2:**
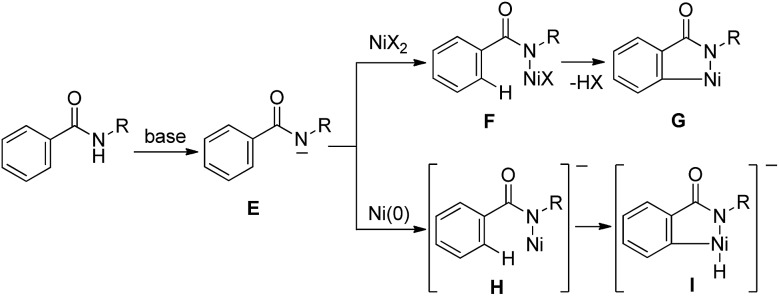
Working hypothesis.

A bidentate chelation system using 2-pyridinylmethylamine and 8-aminoquinoline as the directing group is recognized as a powerful and reliable strategy for developing new types of Ni-catalyzed C–H functionalization. However, developing these directing groups into useful functional groups is not an easy task.^10^ Our next target involves the use of a simpler directing group instead of strong and specific directing groups, such as 2-pyridinylmethylamine and 8-aminoquinoline. Our working hypothesis is that if a strong base could be used, the anion generated by the deprotonation of an amide NH would easily react with the Ni(ii) catalyst to produce a new bond between N and Ni, as in **F**, which would function as a key intermediate for C–H activation (Scheme 2). As mentioned above, C–H bond cleavage would then take place, resulting in the generation of metalacycle **G**. In the case of a Ni(0) catalyst, the nickel complex **H** would be expected to participate in the oxidative addition of C–H bonds to generate complex **I**.^9,11^ Thus, no pre-coordination of an N(sp^2^) atom to a nickel center would be required.

We recently realized such a reaction. Herein, we report the Ni-catalyzed oxidative annulation of C–H bonds in aromatic amides with alkynes, leading to the production of 1(2*H*)-isoquinolinones. A specific directing group is not required for the success of the reaction. A key to the success of the reaction is the use of a catalytic amount of KOBu^*t*^.^12^

## Results and discussion

The reaction of aromatic amide **1a** with 5 equivalents of diphenylacetylene in the presence of Ni(OTf)_2_ (10 mol%) and PPh_3_ (20 mol%) in toluene (0.25 mL) at 160 °C for 14 h gave 2-(4-methoxyphenyl)-3,4-diphenyl-1(2*H*)-isoquinolinone (**2a**) in 67% NMR yield (entry 1 in Table 1). It was found that the catalytic activity of Ni(cod)_2_ is comparable to that of Ni(OTf)_2_ (entry 2). Among the ligands examined (entries 3–5), PPh_3_ was the ligand of choice. Although the use of 4,4′-di-*tert*-butyl-2,2′-bipyridine resulted in a higher yield of **2a** than the phosphine ligands, a small amount of the saturated product, **3a**, was also formed (entry 6). The reaction proceeded efficiently in the presence of a strong base (entries 7 and 8). In sharp contrast, no reaction took place when weak bases were used (entries 9 and 10). Finally, the use of *m*-xylene (0.6 mL) as the solvent and an alkyne (10 equivalents) gave **2a** in 89% isolated yield (entry 14).

**Table 1:** Ni-catalyzed reaction of aromatic amides with diphenylacetylene^*a*^

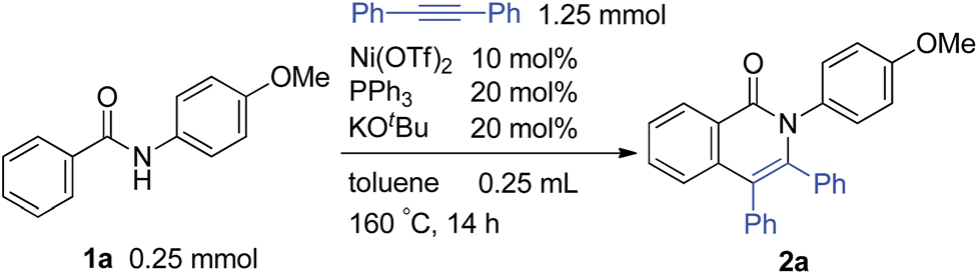			
Entry	Notes	NMR yields	
		**2a**	Recovered **1a**
1	—	67% (61%)	31%
2	Ni(cod)_2_	63% + **3a**, 8%	27%
3	PCy_3_	55%	31%
4	PBu_3_	56%	35%
5	P(OPh)_3_	0%	71%
6	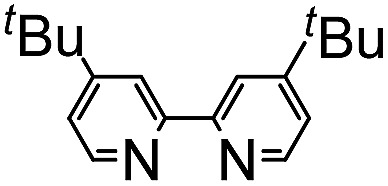	72% + **3a**, 4%	4%
7	LiO^*t*^Bu	62%	37%
8	KOMe	71% + **3a**, 5%	24%
9	KOAc	No reaction	
10	K_3_PO_4_	No reaction	
11	*m*-Xylene	63%	33%
12	140 °C	69% + **3a**, 6%	27%
13	*m*-Xylene, 10 equiv. alkyne	81%	17%
14	*m*-Xylene 0.6 mL, 10 equiv. alkyne	90% (89%; **2a** : **3a** = 39 : 1)	4%
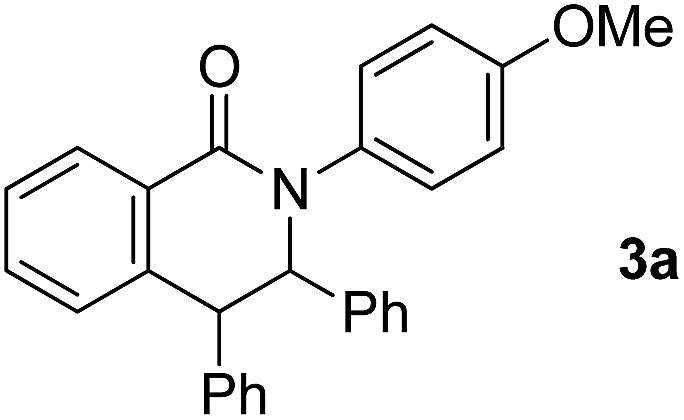			

^*a*^The number in parentheses refers to the isolated yield.

**Scheme 3:**
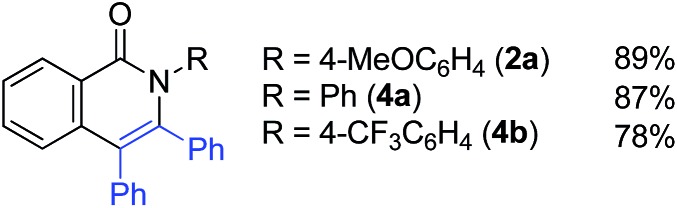
Effect of substituents on the amide nitrogen.

The effect of substituents on the amide nitrogen was examined under optimized reaction conditions (entry 14 in Table 1). Aryl groups containing both electron-donating and electron-withdrawing groups gave the corresponding isoquinolinones in high yields, while alkyl groups, such as methyl and *tert*-butyl groups, gave the corresponding products in poor yields (Scheme 3).

The scope of this oxidative annulation reaction was investigated with respect to the amide used (Table 2). The reaction shows a broad substrate scope and a high functional group tolerance. Various functional groups, including methoxy, dimethylamino, cyano, fluoride and trifluoromethyl groups, were tolerated. In the case of *meta*-trifluoromethyl-substituted aromatic amide **1l**, the less-hindered C–H bond was exclusively activated to give **2l** in 91% isolated yield as a single isomer. On the other hand, in the case of the *meta*-methyl-substituted aromatic amide **1m**, a 2 : 1 mixture of regioisomers of **2m** was obtained.

**Table 2:** Scope of amides^*a*^ ^,^ ^*b*^

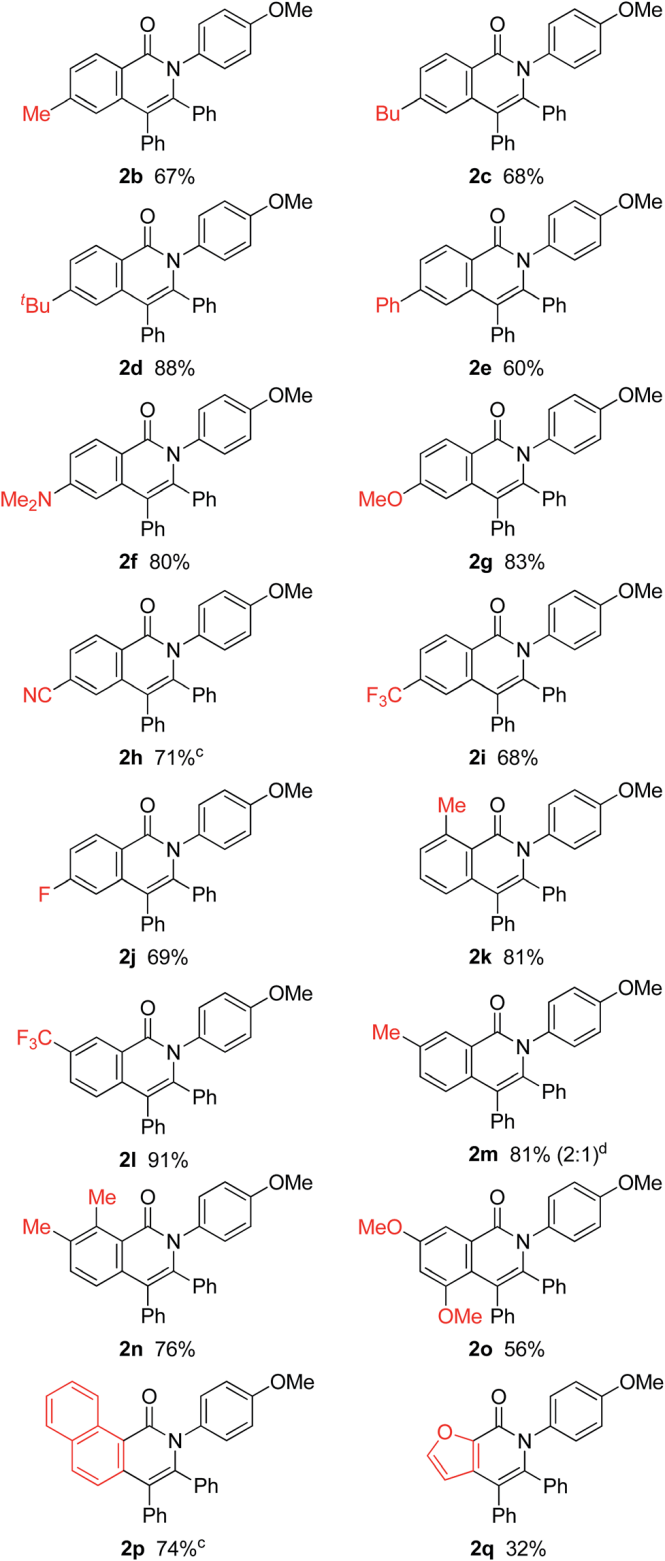

^*a*^Reaction conditions: amide (0.25 mmol), diphenylacetylene (2.5 mmol), Ni(OTf)_2_ (0.025 mmol), PPh_3_ (0.05 mmol) and KOBu^*t*^ (0.05 mmol) in *m*-xylene (0.6 mL) at 160 °C for 14 h.

^*b*^Isolated yields.

^*c*^For 48 h.

^*d*^The number in parentheses refers to the regioselectivity.

The scope of alkynes was also examined (Table 3). Although terminal alkynes did not give the corresponding isoquinolinones, various internal alkynes were applicable to the reaction. When 1-phenyl-1-propyne was used as the alkyne under the standard reaction conditions, the product yield of **2r** was moderate (26% yield) and a regioisomeric mixture (3 : 1) was produced. Gratifyingly, after screening various reaction parameters, the reaction was dramatically improved to give a 77% yield with a high degree of regioselectivity (>50 : 1) when 4,4′-di-*tert*-butyl-2,2′-bipyridine was used as the ligand instead of PPh_3_. The regioselectivity was not affected by the electronic effects of substituents on the aromatic ring of both amides and alkynes, as in **2r**, **2s**, **2t** and **2u**. The use of 4,4′-di-*tert*-butyl-2,2′-bipyridine also dramatically improved the product yield of **2x** in the reaction of **1a** with 4-octyne. The use of the unsymmetrical dialkylacetylene gave nearly a 1 : 1 ratio of regioisomers of **2y**.

**Table 3:** Scope of alkynes^*a*^ ^,^ ^*b*^

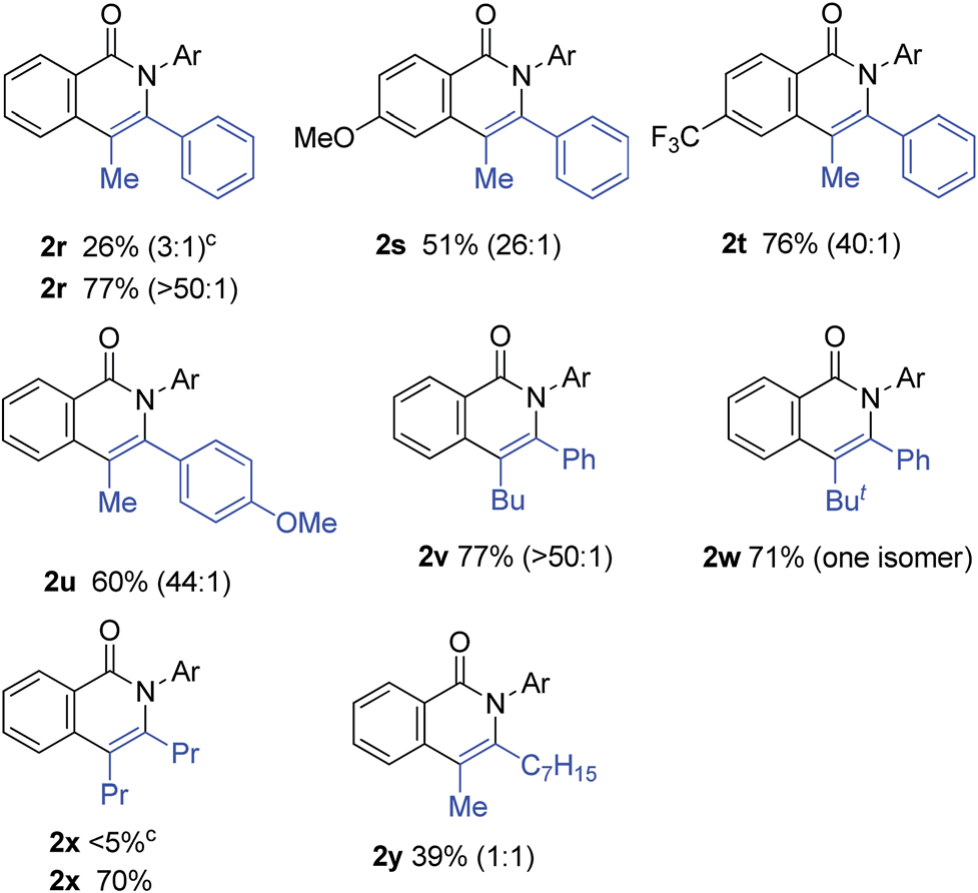

^*a*^Reaction conditions: amide (0.25 mmol), alkyne (2.5 mmol), Ni(OTf)_2_ (0.025 mmol), 4,4′-di-*tert*-butyl-2,2′-bipyridine (0.05 mmol) and KOBu^*t*^ (0.05 mmol) in *m*-xylene (0.6 mL) at 160 °C for 48 h.

^*b*^Isolated yields. The number in parentheses refers to the regioselectivity.

^*c*^The reaction was carried out under the conditions shown in entry 14 in Table 1.

**Scheme 4:**
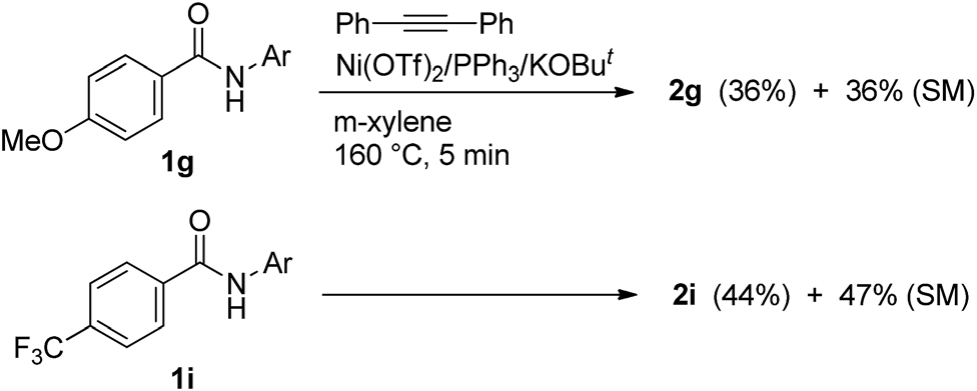
Competition experiments.

To gain mechanistic insights into the reaction, some additional experiments were conducted. We performed a competition experiment using 4-methoxy- and 4-trifluoromethyl-substituted aromatic amides (Scheme 4). However, no significant difference in electronic effects between the two substituents was observed.

**Scheme 5:**
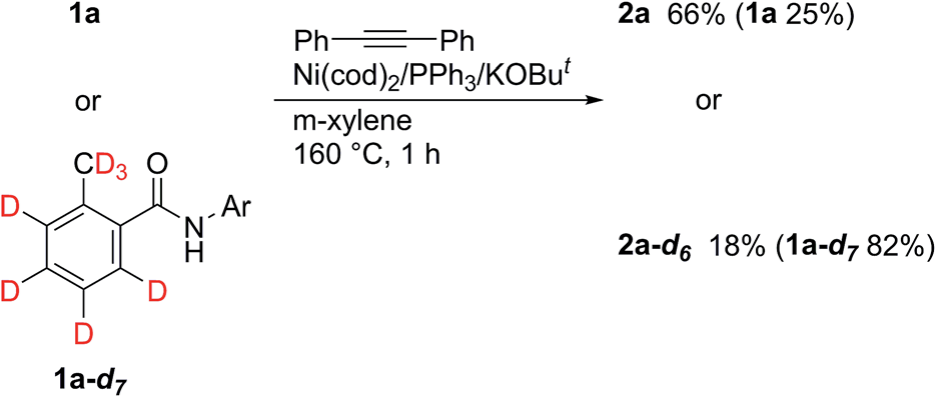
Deuterium labeling experiments.

Deuterium-labeling experiments were also carried out (Scheme 5). No H/D exchange was observed, both in the product and the recovered amide, even at the *ortho*-position, indicating that the cleavage of C–H bonds is irreversible. These results are completely different to those obtained when an 8-aminoquinoline directing group was used.^5^ In addition, a KIE of 3.7 was determined, suggesting that the cleavage of C–H bonds is the rate determining step.

**Scheme 6:**
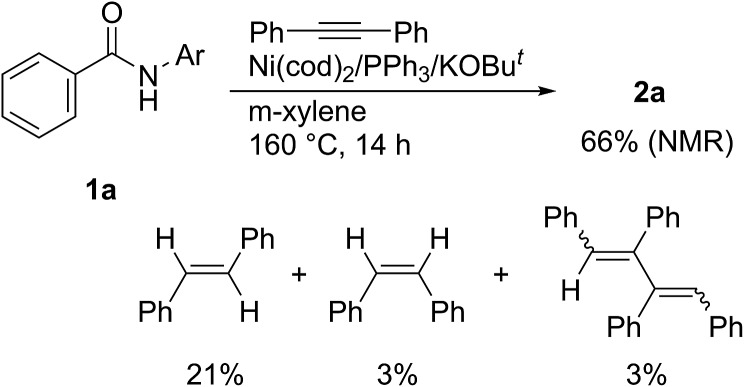
Detection of alkenes.

Although the material balance was not high, it was found that the alkyne functions not only as a two-component coupling partner, but also as a hydrogen acceptor (Scheme 6).

**Scheme 7:**
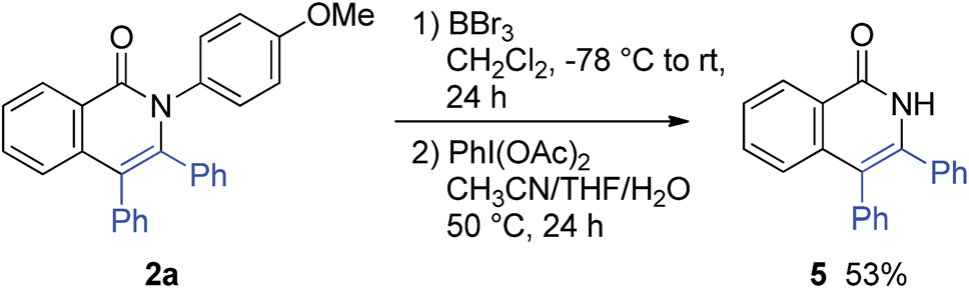
Removal of the *para*-methoxyphenyl group.

The *para*-methoxyphenyl group was successfully removed (Scheme 7). The treatment of **2a** with BBr_3_, followed by PhI(OAc)_2_ in CH_3_CN/THF/H_2_O, gave 3,4-diphenylisoquinolin-1(2*H*)-one (**5**).

**Scheme 8:**
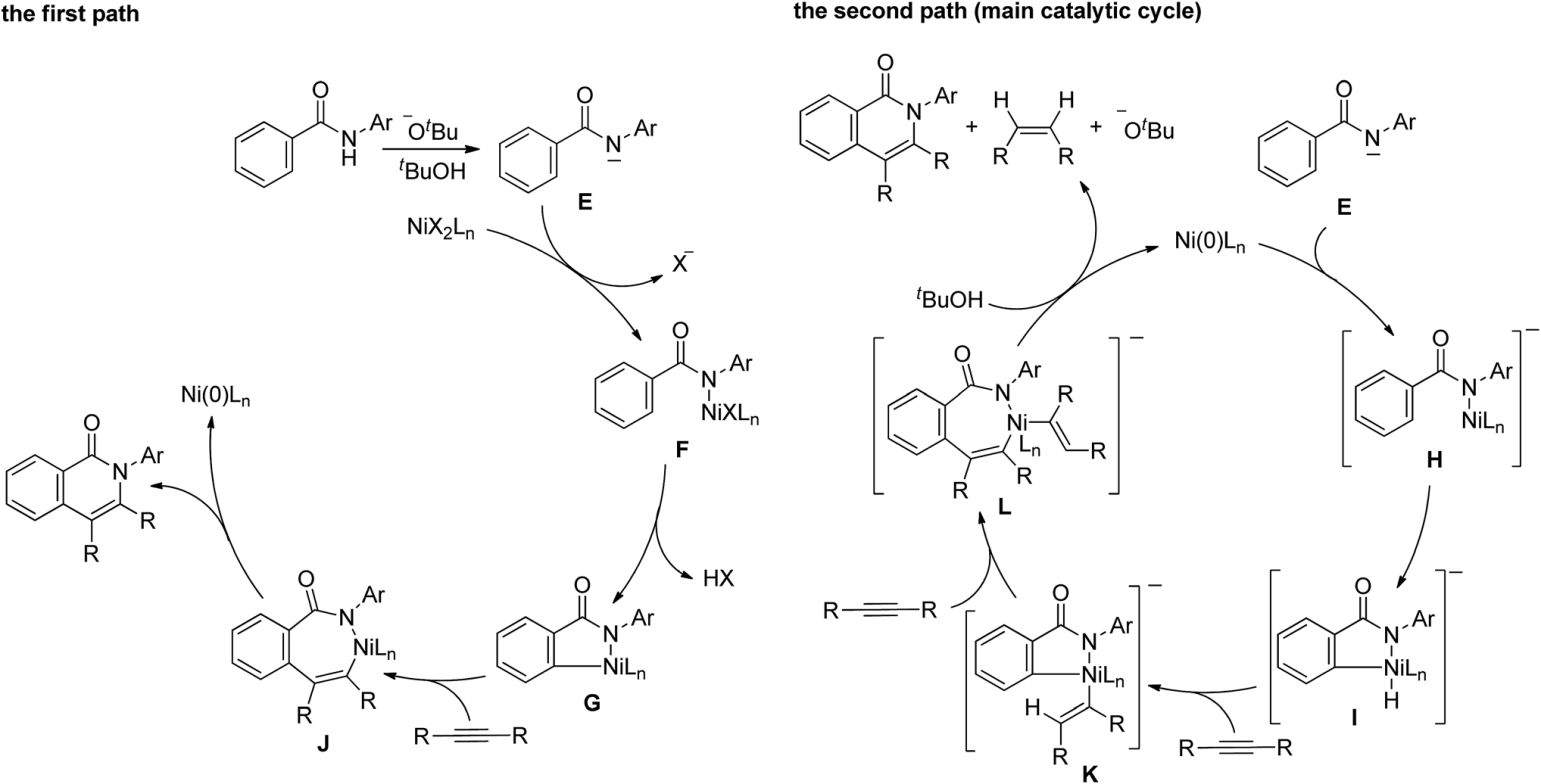
Proposed mechanism.

A proposed mechanism for the above reaction is shown in Scheme 8. The mechanism involves two paths. The Ni(ii) complex initiates the first path (the left scheme). A proton is abstracted from an amide by KOBu^*t*^ to generate the amidate anion, **E**, which reacts with Ni(ii) to give complex **F**. The cleavage of the *ortho* C–H bonds gives nickelacycle **G**, followed by the insertion of an alkyne into a N–Ni bond, which then undergoes reductive elimination to give the isoquinolinone and Ni(0). Ni(0) cannot be oxidized to Ni(ii) under the reaction conditions employed. However, the main catalytic cycle, in which Ni(0) is the key catalytic species, initiates the reaction (the right scheme). The amidate anion, **E**, reacts with Ni(0) to give nickel complex **H**, which is sufficiently reactive to undergo oxidative addition to generate the nickel hydride species, **I**, because the complex is sufficiently electron rich.^11,13^ The successive insertion of an alkyne into H–Ni and C–Ni bonds gives complex **L**. Reductive elimination, followed by protonation by ^*t*^BuOH, affords the isoquinolinone with the regeneration of Ni(0) and KOBu^*t*^, with the concomitant formation of an alkene.

**Scheme 9:**
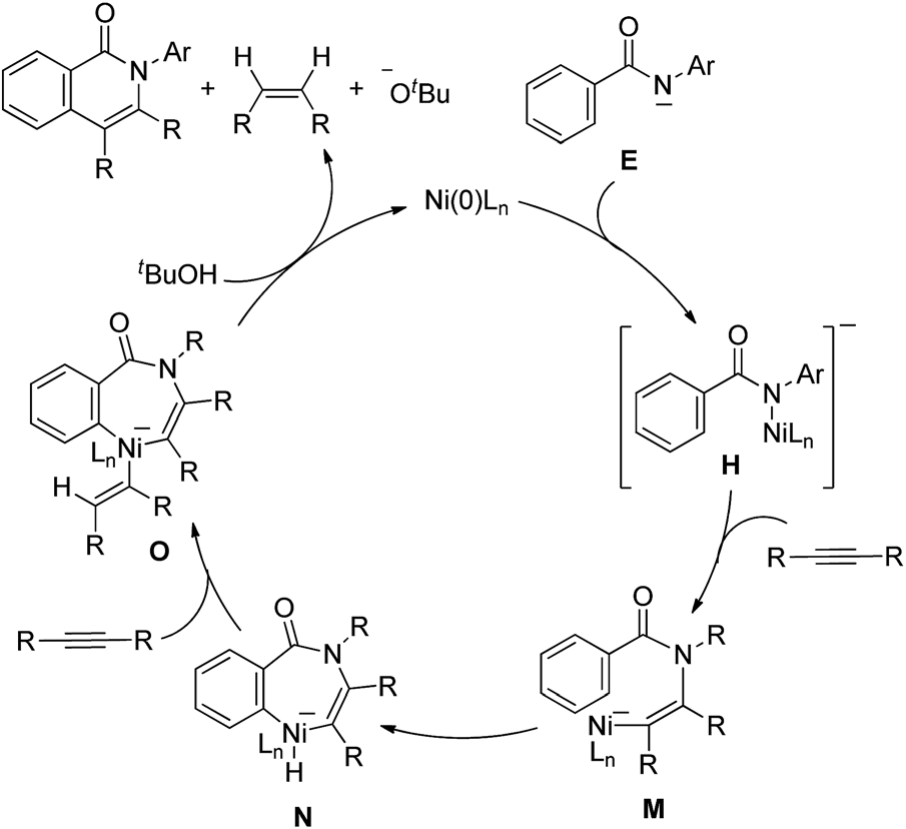
Alternative mechanism.

An alternative mechanism would involve the insertion of an alkyne into the N–Ni bond in **H** to generate complex **M** (Scheme 9).^14^ The oxidative addition of the *ortho* C–H bond gives a seven-membered nickelacycle, **N**, which then permits the insertion of an alkyne to give complex **O**. Complex **O** undergoes reductive elimination and protonation by ^*t*^BuOH to afford the isoquinolinone with the regeneration of Ni(0) and KOBu^*t*^, with the concomitant formation of an alkene. Based on the above findings, this alternative mechanism cannot be excluded.

## Conclusions

In summary, we report the development of a new system for C–H functionalizations catalyzed by nickel complexes. In the past, an *N*,*N*-bidentate chelation system was the only reliable, general and powerful system for developing Ni-catalyzed C–H functionalizations.^6a,b^ The above findings show that aromatic amides with a simple directing group can also participate in Ni-catalyzed C–H functionalization. The reaction displays a broad substrate scope and has a high functional group tolerance. A specific directing group, such as 2-pyridinylmethylamine or 8-aminoquinoline, is not required for the reaction to proceed. A key to the success of the reaction is the use of a catalytic amount of KOBu^*t*^, which forms an N–Ni bond which permits the C–H bond to be activated. The nickel-catalyzed synthesis of isoquinolinones with the extraction of CO or N_2_, or the cleavage of C–halogen bonds that are already present on the aromatic ring, has been reported.^15^ Our new system has the potential for applications in new types of Ni-catalyzed functionalization of C–H bonds, which continues to be a challenging issue.
