# Supplementary material for: Nickel-catalyzed C–H/N–H annulation of aromatic amides with alkynes in the absence of a specific chelation system
Source: Chem Sci. 2017 Jul 24;8(9):6650–5. doi: 10.1039/c7sc01750b (PMC5625264; doi:10.1039/c7sc01750b)
Supplement: Supplementary file 1 [file SC-008-C7SC01750B-s001.pdf]

Electronic Supplementary Material (ESI) for Chemical Science.
This journal is © The Royal Society of Chemistry 2017

**Supporting Information**

# Nickel-Catalyzed C-H/N-H Annulation of Aromatic Amides with Alkynes without a Specific Chelation System

Atsushi Obata, Yusuke Ano, and Naoto Chatani*

*Department of Applied chemistry, Faculty of Engineering, Osaka University, Suita, Osaka 565-0871, Japan*

*chatani@chem.eng.osaka-u.ac.jp*

## Contents

1. **General Information** S2
2. **Materials** S2
3. **General Procedure for the Preparation of Starting Materials** S3
4. **General procedure for the Ni-catalyzed oxidative annulation** S11
5. **Procedure for Deprotection** S24
6. **Copies of $^{1}$H and $^{13}$C NMR Spectra** S26

## 1. General Information

$^{1}$H and $^{13}$C NMR spectra were recorded on a JEOL ECS-400 spectrometer (JEOL, Tokyo, Japan) in $CDCl_3$ or Acetone-$d_6$ with tetramethylsilane as the internal standard. Data are reported as follows: chemical shifts (δ) in ppm, multiplicity (s = singlet, d = doublet, t = triplet, q = quartet, br s = broad singlet, m = multiplet, c = complex), coupling constant (*J*) in hertz (Hz), and integration. Infrared spectra (IR) were obtained using a JASCO FT/IR-4200 spectrometer; absorptions have been reported in reciprocal centimeters with the following relative intensitites: vs (very strong), s (strong), m (medium), w (weak). Mass spectra were obtained on a Shimadzu GCMS-QP 2014 or Shimadzu GCMS-QP 5000 instruments using ionization voltages of 70 eV. High resolution mass spectra (HRMS) were obtained on a JEOL JMS-DX303 system. Melying points (Mp) were determined using a Yamato melting point apparatus. Column chromatography was performed with Silicycle Silia Flash F60 (230-400 mesh, Silycycle Inc.). Most of the compounds were purified by LC-908 HPLC (GPC).

## 2. Materials

All chemicals were used as received.

**Nickel sources**: $Ni(cod)_2$ (Strem Chemicals), $Ni(OTf)_2$ (prepared according to the literature[1]).

**Ligand**: $PCy_3$ (Aldrich), $PBu_3$ (Tokyo Chemical Industry Co., Ltd), $P(OPh)_3$ (Tokyo Chemical Industry Co., Ltd), 4,4'-di-tert-butyl-2,2'-bipyridyl (Tokyo Chemical Industry Co., Ltd), $PPh_3$ (Wako Pure Chemicals Indutries, Ltd).

**Benzamides**: p-anisidine (Aldrich), p-benzanisidide (Tokyo Chemical Industry Co., Ltd).

**Alkynes**: Diphenylacetylene (Tokyo Chemical Industry Co., Ltd), 1-phenyl-1-propyne (Tokyo Chemical Industry Co., Ltd), 1-phenyl-1-butyne (Tokyo Chemical Industry Co., Ltd), 4-octyne (Wako Pure Chemical Industries, Ltd).

**Bases**: KOMe (Aldrich), LiO$^t$Bu (Aldrich), $K_3PO_4$ (Kanto Chemical Co., Inc), KO$^t$Bu (Tokyo Chemical Industry Co., Ltd), KOAc (Wako Pure Chemical Industries, Ltd).

[1] Y. Aihara, N. Chatani, *J. Am. Chem. Soc.* **2013**, *135*, 5308.

**Solvents**: Toluene, super dehydrated (Wako Pure Chemical Industries, Ltd), m-xylene (Wako Pure Chemical Industries, Ltd).

## 3. General Procedure for the Preparation of Starting Materials

All amides were prepared by reacting the corresponding acid or the acid chlorides with p-anisidine.

### (1) Synthesis of amides from acid chlorides.

The acid chloride (15 mmol) was dissolved in $CH_2Cl_2$ (20 mL) and the solution cooled to 0 °C. A solution of p-anisidine (15 mmol) and triethylamine (36 mmol) in 10 mL of $CH_2Cl_2$ was then added dropwise. The resulting mixture was allowed to warm to rt and was then stirred overnight. The crude mixture was washed with a saturated aqueous solution of $NaHCO_3$ (20 mL), and $CH_2Cl_2$ (3x20 mL). The combined organic layers were washed with 1 M HCl aq. (20 mL), dried over anhydrous $Na_2SO_4$ and the solution evaporated taken to dryness.The resulting crude amide was purified by flash chromatography on silica gel (eluent: hexanes/EtOAc = 5/1).

### (2) Synthesis of amides from carboxylic acid.

To a stirred solution of the carboxylic acid (15 mmol) and DMF (5 drops) in $CH_2Cl_2$ (10 mL), $(COCl)_2$ (1.5 mL, 18 mmol) was added dropwise. The solution was magnetically stirred at room temperature for 2 h. The solvent was then eliminated under reduced pressure, and the resulting residue was dissolved in $CH_2Cl_2$ (15 mL). After cooling the reaction mixture to 0 °C, a solution of p-anisidine (15 mmol) and triethylamine (36 mmol) in 10 mL of the same solvent was added dropwise. The resulting mixture was allowed to warm to rt and stirred overnight. The crude product was washed with saturated aqueous $NaHCO_3$ (20 mL), and $CH_2Cl_2$ (3x20 mL) and the organic phase was washed with 1 M HCl aq. (20 mL).The organic phase was dried over anhydrous $Na_2SO_4$ and the solvent removed by evaporation. The resulting crude amide was purified by flash chromatography on silica gel (eluent: hexanes/EtOAc = 5/1).

**p-anisidine (1a)**[2]

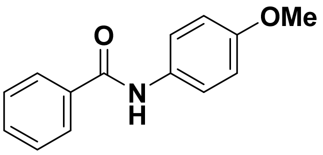


**$^1$H NMR** (399.78 MHz, $CDCl_3$): δ 3.81 (s, 3H), 6.90 (d, *J* = 8.7, 2H), 7.45-7.55 (m, 5H), 7.83-7.86 (m, 3H); **$^{13}$C NMR** (100.53 MHz, $CDCl_3$): δ 55.6, 114.3, 122.2, 127.1, 128.9, 131.1, 131.8, 135.1, 156.7, 165.8.

**N-(4-methoxyphenyl)-4-methylbenzamide (1b)**[3]

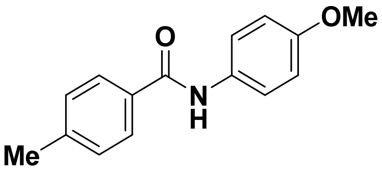


**$^1$H NMR** (399.78 MHz, $CDCl_3$): δ 2.40 (s, 3H), 3.79 (s, 3H), 6.87 (d, *J* = 8.7, 2H), 7.23 (d, *J* = 8.2, 2H), 7.51 (d, *J* = 8.7, 2H), 7.74 (d, *J* = 7.8, 2H), 7.84 (br s, 1H); **$^{13}$C NMR** (100.53 MHz, $CDCl_3$): δ 21.6, 55.6, 114.3, 122.2, 127.1, 129.5, 131.2, 132.2, 142.3, 156.6, 165.8.

**4-butyl-N-(4-methoxyphenyl)benzamide (1c)**

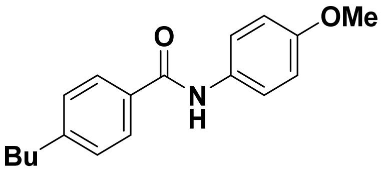


White solid, Mp = 142.5-143.5 °C. $R_f$ = 0.31 (toluene:EtOAc 10:1). **$^1$H NMR** (399.78 MHz, $CDCl_3$): δ 0.94 (t, *J* = 7.3, 3H), 1.37 (m, 2H), 1.62 (m 2H), 2.67 (t, *J* = 7.4, 2H), 3.81 (s, 3H), 6.89 (d, *J* = 9.2, 2H), 7.27 (d, *J* = 8.3, 2H), 7.53 (d, *J* = 8.7, 2H), 7.76-7.80 (m, 3H); **$^{13}$C NMR** (100.53 MHz, $CDCl_3$): δ 14.1, 22.4, 33.5, 35.7, 55.6, 114.3, 122.2, 127.1, 128.9, 131.2, 132.4, 147.3, 156.6, 165.8. **IR** (ATR): 3322 w, 2969 w, 1641 w, 1601 w, 1532 w, 1516 w, 1461 w,

[2] a) C. A. Faler, M. M. Joullié, *Tetrahedron Lett.* **2006**, *47*, 7229 ; b) M. A. Mohamed, K. Yamada, K. Tomeoka, *Tetrahedron Lett.* **2009**, *50*, 3436.

[3] Z. Yao, X. Wei, *Chin. J. Chem.* **2010**, *28*, 2260 ; b) S. Hwang, S. Y. Choi, J. H. Lee, S. Kim, J. In, S. K. Ha, E. Lee, T.-Y. Kim, S. Y. Kim, S. Choi, S. Kim, *Bioorg. Med. Chem.* **2010**, *18*, 5602.

1413 w, 1229 w, 1107 w, 1030 w, 903 w, 821 w, 751 w, 661 w. **MS** (EI+): 283 ($M^+$, 31), 162 (12), 161 (100), 91 (12). **HRMS** (EI+) Calcd for $C_{18}H_{21}NO_2$: 283.1572; Found: 283.1573.

**4-(tert-butyl)-N-(4-methoxyphenyl)benzamide (1d)**[4]

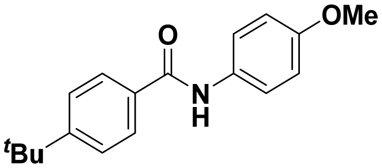


**$^1$H NMR** (399.78 MHz, DMSO-d$_6$): δ 1.32 (s, 9H), 3.74 (s, 3H), 6.93 (d, *J* = 9.2, 2H), 7.53 (d, *J* = 8.7, 2H), 7.69 (d, *J* = 9.2, 2H), 7.88 (d, *J* = 8.2, 2H), 10.08 (s, 1H); **$^{13}$C NMR** (100.53 MHz, DMSO-d$_6$): δ 31.0, 34.7, 55.2, 113.7, 121.9, 125.1, 127.5, 132.4, 154.2, 155.5, 165.1.

**N-(4-methoxyphenyl)-[1,1'-biphenyl]-4-carboxamide (1e)**

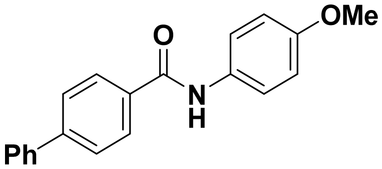


White solid, Mp = 248-249 °C. $R_f$ = 0.33 (toluene:EtOAc 10:1). **$^1$H NMR** (399.78 MHz, DMSO-d$_6$): δ 3.75 (s, 3H), 6.93 (d, *J* = 9.2, 2H), 7.42 (dd, *J* = 7.4, *J* = 7.4, 1H), 7.51 (dd, *J* = 7.4, *J* = 7.4, 2H), 7.70 (d, *J* = 9.2, 2H), 7.76 (d, *J* = 7.4, 2H), 7.83 (d, *J* = 8.2, 2H), 8.05 (d, *J* = 8.2, 2H), 10.2, (s, 1H); **$^{13}$C NMR** (100.53 MHz, DMSO-d$_6$): δ 55.2, 113.8, 122.0, 126.6, 126.9, 128.2, 128.3, 129.1, 132.3, 133.8, 139.1, 142.9, 155.5, 164.7. **IR** (ATR): 3339 w, 1648 w, 1530 w, 1511 w, 1467 w, 1410 w, 1318 w, 1246 w, 1165 m, 1125 m, 1065 w, 1029 w, 899 w, 864 w, 822 m, 770 w. **MS** (EI+): 303 ($M^+$, 40), 182 (14), 181 (100), 153 (18), 152 (26). **HRMS** (EI+) Calcd for $C_{20}H_{17}NO_2$: 303.1259; Found: 303.1258.

**4-(dimethylamino)-N-(4-methoxyphenyl)benzamide (1f)**

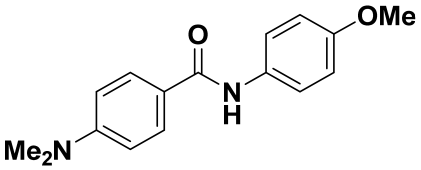


[4] Q.-L. Dong, G.-S. Liu, H.-B. Zhou, L. Chen, Z.-Jun. Yao, *Tetrahedron Lett.* **2008**, *49*, 1636.

White solid, Mp = 175-176 °C. $R_f$ = 0.11 (toluene:EtOAc 10:1). **$^1$H NMR** (399.78 MHz, $CDCl_3$): δ 3.04 (s, 6H), 3.81 (s, 3H), 6.70 (d, *J* = 9.2, 2H), 6.89 (d, *J* = 8.7, 2H), 7.53 (d, *J* = 9.2, 2H), 7.65 (br s, 1H), 7.77 (d, *J* = 9.2, 2H); **$^{13}$C NMR** (100.53 MHz, $CDCl_3$): δ 40.3, 55.6, 111.2, 114.3, 121.5, 122.0, 128.6, 131.7, 152.7, 156.3, 165.6. **IR** (ATR): 1633 w, 1612 w, 1516 w, 1406 w, 1223 w, 1029 w, 822 w, 771 w. **MS** (EI+): 270 ($M^+$, 17), 149 (10), 148 (100). **HRMS** (EI+) Calcd for $C_{16}H_{18}N_2O_2$: 270.1368; Found: 270.1369.

**4-methoxy-N-(4-methoxyphenyl)benzamide (1g)**[5]

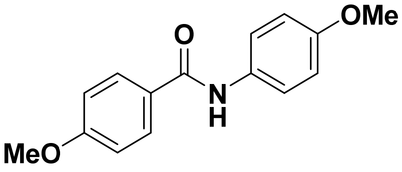


**$^1$H NMR** (399.78 MHz, $CDCl_3$): δ 3.82 (s, 3H), 3.88 (s, 3H), 6.91 (d, *J* = 9.2, 2H), 6.97 (d, *J* = 8.7, 2H), 7.53 (d, *J* = 8.7, 2H), 7.65 (br s, 1H), 7.84 (d, *J* = 8.7, 2H); **$^{13}$C NMR** (100.53 MHz, $CDCl_3$): δ 55.6, 55.7, 114.1, 114.4, 122.2, 127.3, 129.0, 131.3, 156.6, 162.5.

**4-cyano-N-(4-methoxyphenyl)benzamide (1h)**

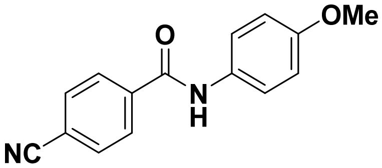


White solid, Mp = 175-176 °C. $R_f$ = 0.11 (toluene:EtOAc 10:1). **$^1$H NMR** (399.78 MHz, $CDCl_3$): δ 3.82 (s, 3H), 6.92 (d *J* = 8.7, 2H), 7.53 (d, *J* = 9.2, 2H), 7.77 (d, *J* = 8.3, 2H), 7.84 (br s, 1H), 7.96 (d, *J* = 8.3, 2H); **$^{13}$C NMR** (100.53 MHz, $CDCl_3$): δ 55.7, 114.4, 115.4, 118.1, 122.4, 127.9, 130.3, 132.7, 139.0, 157.2, 163.9. **IR** (ATR): 3278 w, 1640 w, 1535 w, 1511 w, 1410 w, 1248 w, 1027 w, 823 w, 772 w, 687 w. **MS** (EI+): 253 (14), 252 ($M^+$, 85), 130 (100), 122 (63), 102 (31). **HRMS** (EI+) Calcd for $C_{15}H_{12}N_2O_2$: 252.0899; Found: 252.0902.

**N-(4-methoxyphenyl)-4-(trifluoromethyl)benzamide (1i)**

---

[5] a) Q.-L. Dong, G.-S. Liu, H.-B. Zhou, L. Chen, Z.-J. Yao, *Tetrahedron Lett.* **2008**, *49*, 1636 ; b) B. Karimi, H. Behzadnia, *Synlett* **2010**, *13*, 2019 ; c) F. Shi, J. Li, C. Li, X. Jia, *Tetrahedron Lett.* **2010**, *51*, 6049.

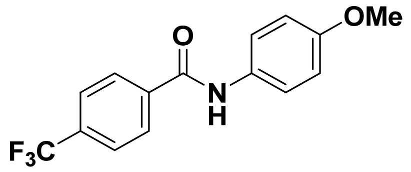

White solid, Mp = 222-223 °C. $R_f$ = 0.33 (toluene:EtOAc 10:1). **$^1$H NMR** (399.78 MHz, DMSO-$d_6$): δ 3.75 (s, 3H), 6.94 (d, *J* = 9.2, 2H), 7.69 (d, *J* = 8.7, 2H), 7.89 (d, *J* = 8.2, 2H), 8.14 (d, *J* = 8.2, 2H), 10.37 (s, 1H); **$^{13}$C NMR** (100.53 MHz, DMSO-$d_6$): δ 55.2, 113.8, 122.1, 124.0 (q, $J_{CF}$ = 270.8), 125.4, 128.5, 131.3 (q, $J_{CF}$ = 31.4), 131.9, 138.9, 155.8, 164.0. **IR** (ATR): 3338 w, 1649 w, 1529 w, 1511 m, 1466 w, 1410 w, 1316 m, 1245 w, 1164 m, 1123 m, 1064 m, 1029 m, 899 w, 864 w, 822 m, 770 w. **MS** (EI+): 296 (10), 295 ($M^+$, 61), 173 (100), 145 (35), 122 (22). **HRMS** (EI+) Calcd for $C_{15}H_{12}F_3NO_2$: 295.0820; Found: 295.0819.

**4-fluoro-N-(4-methoxyphenyl)benzamide (1j)[6]**

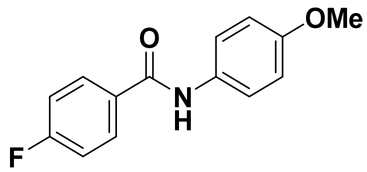

**$^1$H NMR** (399.78 MHz, DMSO-$d_6$): δ 3.74 (s, 3H), 6.93 (d, *J* = 9.2, 2H). 7.35 (dd, *J* = 8.9, *J* = 8.9, 2H), 7.67 (d, *J* = 9.2, 2H), 8.03 (dd, *J* = 8.7, *J* = 5.5, 2H), 10.16 (s, 1H); **$^{13}$C NMR** (100.53 MHz, DMSO-$d_6$): δ 55.2, 113.8, 115.3 (d, $J_{CF}$ = 21.9), 122.1, 130.3 (d, $J_{CF}$ = 8.6), 131.5 (d, $J_{CF}$ = 2.9), 132.1, 155.6, 164.0 (d, $J_{CF}$ = 247.9), 164.0.

**N-(4-methoxyphenyl)-2-methylbenzamide (1k)**

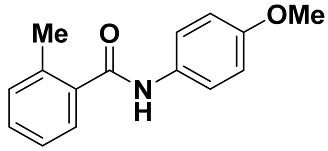

White solid, Mp = 144-145 °C. $R_f$ = 0.23 (toluene:EtOAc 10:1). **$^1$H NMR** (399.78 MHz, $CDCl_3$): δ 2.48 (s, 3H), 3.80 (s, 3H), 6.88 (d, *J* = 9.2, 2H), 7.20-7.26 (m, 2H), 7.34 (dd, *J* = 7.3, *J* = 7.3, 1H), 7.44 (d, *J* = 7.3, 1H), 7.50-7.53 (m, 3H); **$^{13}$C NMR** (100.53 MHz, $CDCl_3$): δ

[6] a) K. Waisser, J. Kune š, L. Kubicová, M. Buděšínský, O. Exner, *Magn. Reson. Chem.* **1997**, *35*, 543 ; b) K. Serdons, C. Terwinghe, P. Vermaelen, K. V. Laere, H. Kung, L. Mortelmans, G. Bormans, A. Verbruggen, *J. Med. Chem.* **2009**, *52*, 1428.

19.9, 55.6, 114.3, 121.8, 125.9, 126.7, 130.3, 131.2, 131.3, 136.5, 136.6, 156.6, 168.1. **IR** (ATR): 3279 w, 1645 m, 1599 w, 1510 s, 1460 w, 1410 m, 1324 w, 1301 w, 1266 w, 1243 m, 1178 w, 1033 m, 903 w, 825 m, 795 w, 740 m, 694 w. **MS** (EI+): 241 ($M^+$, 40), 119 (100), 91 (32). **HRMS** (EI+) Calcd for $C_{15}H_{15}NO_2$: 241.1103; Found: 241.1102.

**N-(4-methoxyphenyl)-3-(trifluoromethyl)benzamide (1l)**

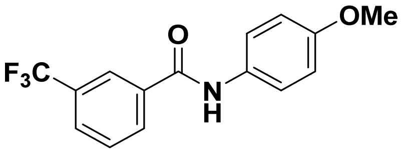


White solid, Mp = 125-126 °C. $R_f$ = 0.20 (toluene:EtOAc 10:1). **$^1$H NMR** (399.78 MHz, $CDCl_3$): δ 3.75 (s, 3H), 6.80 (d, *J* = 8.7, 2H), 7.45-7.48 (m, 3H), 7.70 (d, *J* = 7.8, 1H), 7.96 (d, *J* = 7.8, 1H), 8.05 (s, 1H), 8.54 (br s, 1H); **$^{13}$C NMR** (100.53 MHz, $CDCl_3$): δ 55.5, 114.2, 114.2, 122.8, 123.7 (q, $J_{CF}$ = 271.2), 124.2, 128.2, 129.3, 130.5, 131.0 (q, $J_{CF}$ = 32.0), 135.8, 156.9, 164.8. **IR** (ATR): 3330 w, 1647 w, 1598 w, 1510 m, 1482 w, 1412 w, 1317 w, 1226 w, 1175 w, 1106 w, 1034 w, 900 w, 826 w, 745 w, 694 w. **MS** (EI+): 296 (10), 295 ($M^+$, 63), 173 (100), 145 (34), 122 (21). **HRMS** (EI+) Calcd for $C_{15}H_{12}F_3NO_2$: 295.0820; Found: 295.0820.

**N-(4-methoxyphenyl)-3-methylbenzamide (1m)**

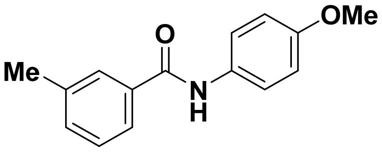


White solid, Mp = 127.5-128.5 °C. $R_f$ = 0.25 (toluene:EtOAc 10:1). **$^1$H NMR** (399.78 MHz, $CDCl_3$): δ 2.39 (s, 3H), 3.80 (s, 3H), 6.87 (d, *J* = 7.3, 2H), 7.32 (d, *J* = 5.4, 2H), 7.53 (d, *J* = 8.7, 2H), 7.61-7.66 (m, 2H), 7.91 (br s, 1H); **$^{13}$C NMR** (100.53 MHz, $CDCl_3$): δ 21.5, 55.6, 114.3, 122.2, 124.1, 127.9, 128.7, 131.2, 132.5, 135.1, 138.7, 156.6, 166.0. **IR** (ATR): 3299 w, 1643 m, 1601 w, 1511 s, 1411 w, 1320 w, 1235 m, 1176 w, 1034 w, 828 w, 806 w, 740 w, 690 w. **MS** (EI+): 241 ($M^+$, 47), 119 (100), 91 (31). **HRMS** (EI+) Calcd for $C_{15}H_{15}NO_2$: 241.1103; Found: 241.1102.

**N-(4-methoxyphenyl)-2,3-dimethylbenzamide (1n)**

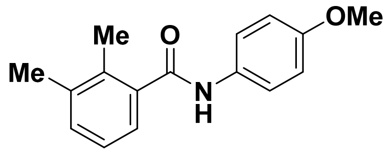

White solid, Mp = 149-150 °C. $R_f$ = 0.28 (toluene:EtOAc 10:1). **$^1$H NMR** (399.78 MHz, $CDCl_3$): δ 2.32 (s, 3H), 2.37 (s, 3H), 3.82 (s, 3H), 6.91 (d, *J* = 9.2, 2H), 7.15 (dd, *J* = 7.8, *J* = 7.3, 1H), 7.24 (d, *J* = 7.4, 1H), 7.29 (d, *J* = 7.8, 1H), 7.39 (br s, 1H), 7.53 (d, *J* = 8.7, 2H); **$^{13}$C NMR** (100.53 MHz, $CDCl_3$): δ 16.5, 20.5, 55.7, 114.3, 121.7, 124.3, 125.8, 131.2, 131.6, 134.5, 137.5, 138.3, 156.6, 168.8. **IR** (ATR): 3296 w, 1643 m, 1599 w, 1511 m, 1462 w, 1410 w, 1319 w, 1235 m, 1176 w, 1032 w, 823 m, 771 w. **MS** (EI+): 255 ($M^+$, 34), 133 (100), 105 (22). **HRMS** (EI+) Calcd for $C_{16}H_{17}NO_2$: 255.1259; Found: 255.1262.

**3,5-dimethoxy-N-(4-methoxyphenyl)benzamide (1o)**

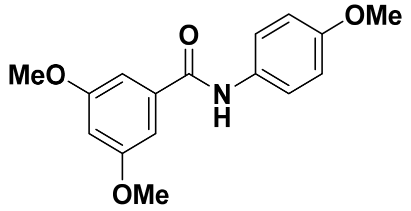

White solid, Mp = 103-104.5 °C. $R_f$ = 0.17 (toluene:EtOAc 10:1). **$^1$H NMR** (399.78 MHz, $CDCl_3$): δ 3.80 (s, 9H), 6.58 (s, 1H), 6.88 (d, *J* = 8.7, 2H), 6.96 (s, 2H), 7.52 (d, *J* = 9.2, 2H), 7.89 (br s, 1H); **$^{13}$C NMR** (100.53 MHz, $CDCl_3$): 55.6, 55.7, 103.7, 105.0, 114.3, 122.2, 131.0, 137.3, 156.7, 161.0, 165.6. **IR** (ATR): 3287 w, 1646 w, 1591 m, 1510 s, 1457 m, 1425 m, 1411 m, 1348 m, 1328 m, 1301 m, 1237 m, 1204 m, 1154 s, 1049 m, 927 w, 828 m, 796 m, 758 w, 681 w. **MS** (EI+): 287 ($M^+$, 47), 165 (100), 137 (19), 122 (13). **HRMS** (EI+) Calcd for $C_{16}H_{17}NO_4$: 287.1158; Found: 287.1160.

**N-(4-methoxyphenyl)-1-naphthamide (1p)**

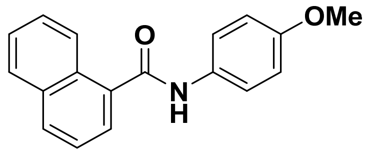

Orange solid, Mp = 177-178 °C. $R_f$ = 0.33 (toluene:EtOAc 10:1). **$^1$H NMR** (399.78 MHz, $CDCl_3$): δ 3.83 (s, 3H), 6.93 (d, *J* = 8.7, 2H), 7.46-7.60 (m, 5H), 7.67-7.72 (m, 2H), 7.89 (d, *J* = 9.2, 1H), 7.95 (d, *J* = 8.2, 1H), 8.36 (d, *J* = 8.7, 1H); **$^{13}$C NMR** (100.53 MHz, $CDCl_3$): δ

55.7, 114.4, 121.9, 124.9, 125.2, 125.4, 126.7, 127.4, 128.5, 130.2, 131.1, 131.2, 133.9, 134.7, 156.8, 167.5. **IR** (ATR): 3270 w, 1643 m, 1600 w, 1508 s, 1463 w, 1412 w, 1320 w, 1243 m, 1177 w, 1141 w, 1033 m, 899 w, 828 m, 800 m, 774 s. **MS** (EI+): 277 ($M^+$, 35), 156 (12), 155 (100), 127 (46). **HRMS** (EI+) Calcd for $C_{18}H_{15}NO_2$: 277.1103; Found: 277.1100.

**N-(4-methoxyphenyl)furan-2-carboxamide (1q)**[7]

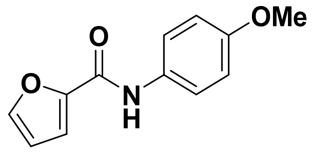


**$^1$H NMR** (399.78 MHz, DMSO-$d_6$): δ 3.73 (s, 3H), 6.68-6.69 (m, 1H), 6.91 (d, $J$ = 9.2, 2H), 7.28 (d, $J$ = 3.2, 1H), 7.64 (d, $J$ = 9.2, 2H), 7.92 (d, $J$ = 0.7, 1H), 10.08 (s, 1H); **$^{13}$C NMR** (100.53 MHz, DMSO-$d_6$): δ 55.2, 112.1, 113.8, 114.3, 122.0, 131.5, 145.5, 147.7, 155.6, 156.0.

**N-(4-methoxyphenyl)-2-(methyl-d$_3$)benzamide-3,4,5,6-d$_4$ (1a-*d*$_7$)**

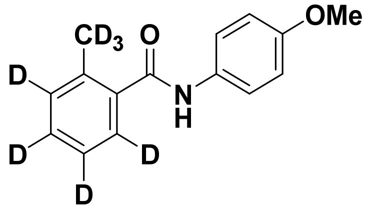


White solid, Mp = 143-144 °C. $R_f$ = 0.22 (toluene:EtOAc 10:1). **$^1$H NMR** (399.78 MHz, $CDCl_3$): δ 3.80 (s, 3H), 6.88 (d, $J$ = 8.7, 2H), 7.50 (d, $J$ = 8.7, 2H), 7.58 (br s, 1H); **$^{13}$C NMR** (100.53 MHz, $CDCl_3$): δ 19.2 (q, $J_{CD}$ = 20.0), 55.6, 114.3, 121.9, 125.4 (t, $J_{CD}$ = 24.8), 126.3 (t, $J_{CD}$ = 23.8), 129.7 (t, $J_{CD}$ = 25.7), 130.8 (t, $J_{CD}$ = 23.8), 131.2, 136.3, 136.5, 156.6, 168.1. **IR** (ATR): 3280 w, 1643 w, 1514 w, 1411 w, 1244 w, 1032 w, 822 w, 772 w, 685 w. **MS** (EI+): 248 ($M^+$, 52), 126 (100), 98 (30). **HRMS** (EI+) Calcd for $C_{15}H_8D_7NO_2$: 248.1542; Found 248.1541.

[7] C. K. Lee, J. S. Yu, Y. R. Ji, *J. Heterocyclic Chem.* **2002**, *39*, 1219.

## 4. General procedure for the Ni-catalyzed oxidative annulation

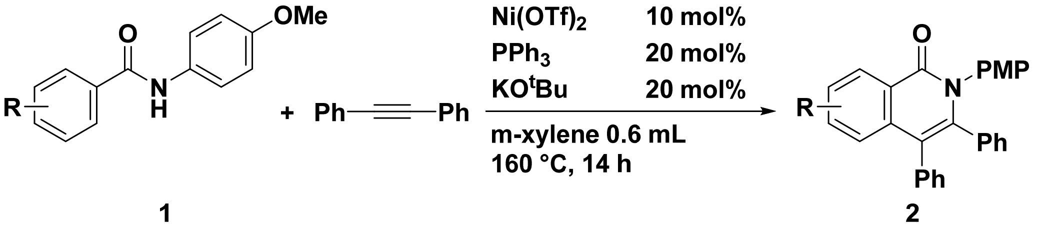


1 0.25 mmol 2.5 mmol 2

To an oven-dried 5 mL screw-capped vial, in a glove box p-anisidine (**1a**, 56.8 mg, 0.25 mmol), diphenylacetylen (450 mg, 2.5 mmol), $Ni(OTf)_2$ (8.9 mg, 0.025 mmol), $PPh_3$ (13.1 mg, 0.05 mmol), KOtBu$^t$ (5.7 mg, 0.05 mmol) and m-xylene (0.6 mL) were added. The mixture was stirred for 14 h at 160 °C followed by cooling. The resulting mixture was filtered through a celite pad and the filtrate concentrated in vacuo. The residue was purified by column chromatography on silica gel (eluent : toluene/EtOAc= 10/1) to afford the desired product **2a** (89.5 mg, 89%) as a white solid.

### 2-(4-methoxyphenyl)-3,4-diphenylisoquinolin-1(2H)-one (2a)

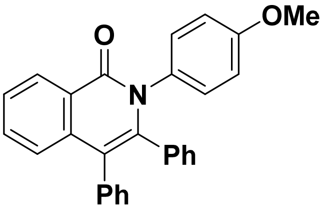


White solid, Mp = 220-221 °C. $R_f$ = 0.17 (toluene:EtOAc 10:1). Yield = 89%, m = 89.5 mg. $^1$**H NMR** (399.78 MHz, $CDCl_3$): δ 3.70 (s, 3H), 6.72 (d, $J$ = 9.2, 2H), 6.88-6.92 (m, 5H), 7.01 (d, $J$ = 9.2, 2H), 7.12-7.26 (m, 6H), 7.49-7.60 (m, 2H), 8.57 (d, $J$ = 8.2, 1H); $^{13}$**C NMR** (100.53 MHz, $CDCl_3$): δ 55.4, 114.0, 118.8, 125.6, 125.7, 126.9, 127.3, 127.3, 128.1, 128.4, 130.4, 131.1, 131.7, 132.3, 132.6, 135.0, 136.5, 137.7, 141.5, 158.5, 163.0. **IR** (ATR): 3057 w, 1712 w, 1655 m, 1606 w, 1510 m, 1442 w, 1329 w, 1299 w, 1247 m, 1031 w, 778 w, 701 m. **MS** (EI+): 404 (30), 403 ($M^+$, 100), 402 (50), 280 (10). **HRMS** (EI+) Calcd for $C_{28}H_{21}NO_2$: 403.1572; Found: 403.1574.

### 2-(4-methoxyphenyl)-6-methyl-3,4-diphenylisoquinolin-1(2H)-one (2b)

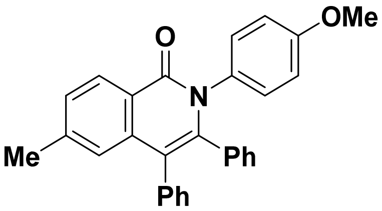

White solid, Mp = 268-269 °C. $R_f$ = 0.17 (toluene:EtOAc 10:1). Yield = 67%, m = 69.9 mg. **$^1$H NMR** (399.78 MHz, $CDCl_3$): δ 2.38 (s, 3H), 3.71 (s, 3H), 6.72 (d, *J* = 9.2, 2H), 6.86-6.92 (m, 5H), 6.99-7.01 (m, 3H), 7.11-7.23 (m, 5H), 7.34 (d, *J* = 8.2, 1H), 8.45 (d, *J* = 8.2, 1H); **$^{13}$C NMR** (100.53 MHz, $CDCl_3$): δ 22.2, 55.4, 114.0, 118.7, 123.4, 125.4, 126.9, 127.2, 128.1, 128.5, 128.6, 130.5, 131.1, 131.8, 132.5, 135.2, 136.7, 137.8, 141.6, 143.2, 158.5, 163.0. **IR** (ATR): 2992 w, 1641 w, 1614 w, 1508 w, 1442 w, 1330 w, 1246 w, 1166 w, 1030 w, 770 m, 700 w. **MS** (EI+): 418 (31), 417 ($M^+$, 100), 416 (52). **HRMS** (EI+) Calcd for $C_{29}H_{23}NO_2$: 417.1729; Found: 417.1727.

**6-butyl-2-(4-methoxyphenyl)-3,4-diphenylisoquinolin-1(2H)-one (2c)**

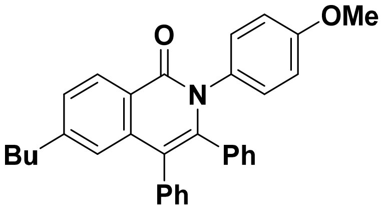

White solid, Mp = 186-187 °C. $R_f$ = 0.17 (toluene:EtOAc 10:1). Yield = 68%, m = 78.0 mg. **$^1$H NMR** (399.78 MHz, $CDCl_3$): δ 0.88 (t, *J* = 7.4, 3H), 1.28-1.34 (m, 2H), 1.53-1.56 (m, 2H), 2.62 (t, *J* = 7.8, 2H), 3.70 (s, 3H), 6.72 (d, *J* = 8.7, 2H), 6.86-6.91 (m, 5H), 6.99-7.02 (m, 3H), 7.16-7.25 (m, 5H), 7.36 (d, *J* = 8.2, 1H), 8.48 (d, *J* = 8.2, 1H); **$^{13}$C NMR** (100.53 MHz, $CDCl_3$): δ 14.0, 22.4, 33.5, 36.2, 55.4, 113.9, 118.7, 123.6, 124.9, 126.8, 127.2, 127.8, 128.0, 128.5, 130.5, 131.1, 131.8, 132.4, 135.2, 136.7, 137.8, 141.5, 148.1, 158.5, 163.0. **IR** (ATR): 2930 w, 1654 m, 1613 m, 1509 m, 1478 w, 1442 w, 1332 w, 1298 w, 1247 m, 1030 w, 698 m. **MS** (EI+): 460 (34), 459 ($M^+$, 100), 458 (36). **HRMS** (EI+) Calcd for $C_{32}H_{29}NO_2$: 459.2198; Found: 459.2196.

**6-(tert-butyl)-2-(4-methoxyphenyl)-3,4-diphenylisoquinolin-1(2H)-one (2d)**

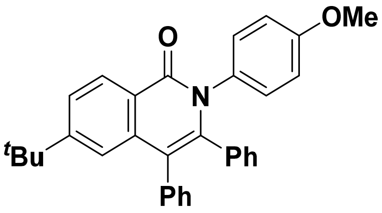

White solid, Mp = 257-258 °C. $R_f$ = 0.14 (toluene:EtOAc 10:1). Yield = 88%, m = 101.4 mg. **$^{1}$H NMR** (399.78 MHz, $CDCl_3$): δ 1.25 (s, 9H), 3.70 (s, 3H), 6.72 (d, *J* = 8.7, 2H), 6.86-6.93 (m, 5H), 6.99 (d, *J* = 8.7, 2H), 7.12-7.25 (m, 6H), 7.59 (d, *J* = 8.2, 1H), 8.49 (d, *J* = 8.2, 1H); **$^{13}$C NMR** (100.53 MHz, $CDCl_3$): δ 31.1, 35.4, 55.4, 114.0, 119.1, 121.8, 123.4, 125.0, 126.9, 127.2, 128.0, 128.2, 130.5, 131.2, 131.7, 132.5, 135.2, 136.7, 137.6, 141.5, 156.1, 158.5, 162.9. **IR** (ATR): 2962 w, 1654 m, 1611 m, 1510 m, 1481 w, 1328 w, 1299 w, 1247 m, 1030 w, 755 m, 704 m. **MS** (EI+): 460 (35), 459 ($M^{+}$, 100), 458 (35). **HRMS** (EI+) Calcd for $C_{32}H_{29}NO_2$: 459.2198; Found: 459.2194.

**2-(4-methoxyphenyl)-3,4,6-triphenylisoquinolin-1(2H)-one (2e)**

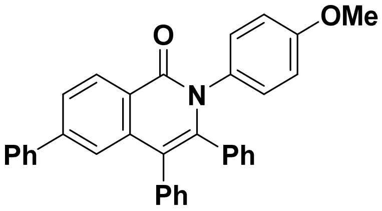

White solid, Mp = 298-299 °C. $R_f$ = 0.11 (toluene:EtOAc 10:1). Yield = 60%, m = 69.2 mg. **$^{1}$H NMR** (399.78 MHz, $CDCl_3$): δ 3.72 (s, 3H), 6.74 (d, *J* = 8.7, 2H), 6.89-6.94 (m, 5H), 7.03 (d, *J* = 8.7, 2H), 7.15-7.23 (m, 5H), 7.35-7.45 (m, 4H), 7.51-7.53 (m, 2H), 7.75 (d, *J* = 8.2, 1H), 8.63 (d, *J* = 8.7, 1H); **$^{13}$C NMR** (100.53 MHz, $CDCl_3$): δ 55.4, 114.0, 114.4, 119.0, 123.9, 124.5, 126.2, 127.0, 127.3, 127.4, 127.6, 128.2, 129.0, 129.1, 130.5, 131.1, 131.8, 132.4, 135.1, 136.5, 138.2, 140.5, 142.0, 145.4, 158.6, 163.0. **IR** (ATR): 1653 w, 1603 w, 1507 w, 1326 w, 1243 w, 1026 w, 815 w, 768 w, 696 w. **MS** (EI+): 480 (36), 479 ($M^{+}$, 100), 478 (42). **HRMS** (EI+) Calcd for $C_{34}H_{25}NO_2$: 479.1885; Found: 479.1880.

**6-(dimethylamino)-2-(4-methoxyphenyl)-3,4-diphenylisoquinolin-1(2H)-one (2f)**

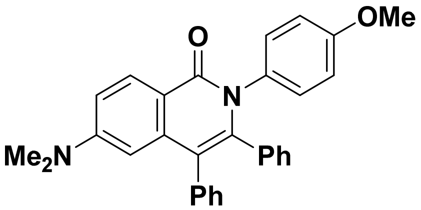

White solid, Mp = 286-288 °C. $R_f$ = 0.06 (toluene:EtOAc 10:1). Yield = 80%, m = 89.8 mg. **$^1$H NMR** (399.78 MHz, $CDCl_3$): δ 2.90 (s, 6H), 3.69 (s, 3H), 6.25 (d, *J* = 2.7, 1H), 6.69 (d, *J* = 9.2, 2H), 6.86-6.94 (m, 6H), 6.99 (d, *J* = 8.7, 2H), 7.11-7.17 (m, 5H), 8.38 (d, *J* = 9.2, 1H); **$^{13}$C NMR** (100.53 MHz, $CDCl_3$): δ 40.1, 55.4, 105.2, 112.8, 113.8, 115.2, 118.6, 126.7, 127.1, 127.1, 127.9, 129.9, 130.7, 131.1, 131.8, 132.7, 135.5, 137.2, 139.4, 141.6, 153.1, 158.3, 162.9. **IR** (ATR): 2925 w, 2360 w, 1649 w, 1602 w, 1511 w, 1367 w, 1228 w, 1029 w, 706 w. **MS** (EI+): 447 (32), 446 ($M^+$, 100), 445 (62), 223 (16). **HRMS** (EI+) Calcd for $C_{30}H_{26}N_2O_2$: 446.1994; Found: 446.1995.

**6-methoxy-2-(4-methoxyphenyl)-3,4-diphenylisoquinolin-1(2H)-one (2g)**

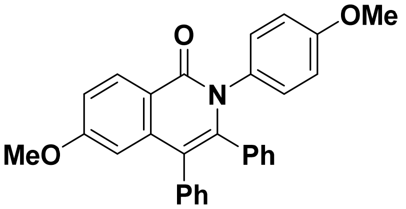


White solid, Mp = 251-252 °C. $R_f$ = 0.06 (toluene:EtOAc 10:1). Yield = 83%, m = 90.4 mg. **$^1$H NMR** (399.78 MHz, $CDCl_3$): δ 3.70 (s, 3H), 3.72 (s, 3H), 6.61 (d, *J* = 2.8, 1H), 6.72 (d, *J* = 8.7, 2H), 6.86-6.93 (m, 5H), 7.00 (d, *J* = 8.7, 2H), 7.07-7.22 (m, 6H), 8.48 (d, *J* = 8.7, 1H); **$^{13}$C NMR** (100.53 MHz, $CDCl_3$): δ 55.4, 55.4, 107.6, 114.0, 115.6, 118.5, 119.5, 127.0, 127.3, 127.3, 128.1, 130.6, 130.6, 131.0, 131.7, 132.4, 135.1, 136.6, 139.8, 142.2, 158.5, 162.7, 163.1. **IR** (ATR): 2924 w, 1649 w, 1604 w, 1510 w, 1373 w, 1218 w, 1028 w, 771 w, 698 w. **MS** (EI+): 434 (31), 433 ($M^+$, 100), 432 (57). **HRMS** (EI+) Calcd for $C_{29}H_{23}NO_3$: 433.1678; Found: 433.1681.

**2-(4-methoxyphenyl)-1-oxo-3,4-diphenyl-1,2-dihydroisoquinoline-6-carbonitrile (2h)**

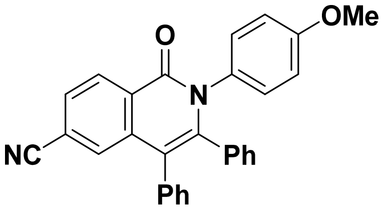


White solid, Mp = 219-222 °C. $R_f$ = 0.14 (toluene:EtOAc 10:1). Yield = 71%, m = 76.1 mg. **$^1$H NMR** (399.78 MHz, $CDCl_3$): δ 3.72 (s, 3H), 6.74 (d, *J* = 9.2, 2H), 6.86-6.89 (m, 2H), 6.92-6.96 (m, 3H), 7.00 (d, *J* = 8.7, 2H), 7.09 (d, *J* = 7.3, 2H), 7.21-7.27 (m, 3H), 7.58 (s, 1H), 7.70 (d, *J* = 8.3, 1H), 8.64 (d, *J* = 8.7, 1H); **$^{13}$C NMR** (100.53 MHz, $CDCl_3$): δ 55.5, 114.2,

116.1, 117.9, 118.6 127.5, 127.6, 127.8, 128.0, 128.0, 128.6, 128.7, 129.3, 129.6, 130.2, 130.5, 130.6, 130.8, 131.5, 131.6, 134.2, 135.1, 138.0, 143.7, 158.8, 162.0. **IR** (ATR): 3021 w, 2232 w, 1659 m, 1613 w, 1552 w, 1509 m, 1474 w, 1443 w, 1324 m, 1297 w, 1248 m, 1173 w, 1029 w, 819 w, 751 m, 697 m, 666 w. **MS** (EI+): 429 (31), 428 ($M^+$, 100), 427 (47). **HRMS** (EI+) Calcd for $C_{29}H_{20}N_2O_2$: 428.1525; Found: 428.1524.

**2-(4-methoxyphenyl)-3,4-diphenyl-6-(trifluoromethyl)isoquinolin-1(2H)-one (2i)**

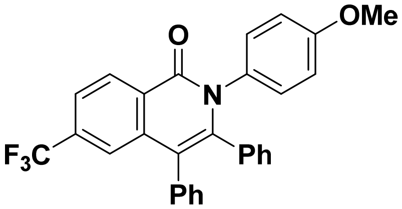


White solid, Mp = 234-235 °C. $R_f$ = 0.23 (toluene:EtOAc 10:1). Yield = 68%, m = 80.5 mg. **$^1$H NMR** (399.78 MHz, $CDCl_3$): δ 3.71 (s, 3H), 6.73 (d, *J* = 8.7, 2H), 6.86-6.90 (m, 2H), 6.91-6.94 (m, 3H), 7.00 (d, *J* = 9.2, 2H), 7.09-7.12 (m, 2H), 7.17-7.24 (m, 3H), 7.51 (s, 1H), 7.71 (d, *J* = 8.5, 1H), 8.66 (d, *J* = 8.2, 1H); **$^{13}$C NMR** (100.53 MHz, $CDCl_3$): δ 55.4, 114.1, 118.6, 122.9, 122.9, 123.8 (q, $J_{CF}$ = 271.7), 127.4, 127.6, 127.7, 128.4, 129.6, 130.3, 130.9, 131.6, 131.9, 134.2 (q, $J_{CF}$ = 32.4), 134.5, 135.5, 137.8, 143.2, 158.8, 162.3. **IR** (ATR): 1660 w, 1590 w, 1561 w, 1510 w, 1443 w, 1314 m, 1279 w, 1249 w, 1170 w, 1127 m, 1074 w, 1031 w, 928 w, 829 w, 794 w, 763 w, 722 w, 700 w, 676 w. **MS** (EI+): 472 (31), 471 ($M^+$, 100), 470 (49). **HRMS** (EI+) Calcd for $C_{29}H_{20}F_3NO_2$: 471.1446; Found: 471.1447.

**6-fluoro-2-(4-methoxyphenyl)-3,4-diphenylisoquinolin-1(2H)-one (2j)**

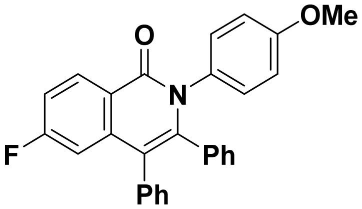


White solid, Mp = 223-225 °C. $R_f$ = 0.14 (toluene:EtOAc 10:1). Yield = 69%, m = 73.0 mg. **$^1$H NMR** (399.78 MHz, $CDCl_3$): δ 3.71 (s, 3H), 6.72 (d, *J* = 8.7, 2H), 6.84-6.93 (m, 6H), 7.00 (d, *J* = 8.7, 2H), 7.11 (d, *J* = 7.3, 2H), 7.17-7.25 (m, 4H), 8.57 (dd, *J* = 9.2, *J* = 6.0, 1H); **$^{13}$C NMR** (100.53 MHz, $CDCl_3$): δ 55.4, 110.9 (d, $J_{CF}$ = 22.9), 114.0, 115.5, (d, $J_{CF}$ = 22.9), 118.3, 122.2, 127.2, 127.3, 127.5, 128.3, 130.4, 130.9, 131.6, 131.7 (d, $J_{CF}$ = 10.5), 132.1, 134.7, 136.0, 140.2 (d, $J_{CF}$ = 9.5), 142.9, 158.6, 162.4, 165.6 (d, $J_{CF}$ = 250.8). **IR** (ATR): 2928 w,

1712 w, 1658 m, 1612 m, 1559 w, 1510 m, 1474 m, 1443 w, 1363 w, 1329 m, 1298 w, 1247 m, 1222 w, 1180 m, 1130 w, 1103 w, 1029 w, 987 w, 948 w, 871 w, 821 w, 782 w, 766 w, 733 m, 699 m. **MS** (EI+): 422 (30), 421 (M $^{+}$, 100), 420 (59). **HRMS** (EI+) Calcd for $C_{28}H_{20}FNO_2$: 421.1478; Found: 421.1474.

**2-(4-methoxyphenyl)-8-methyl-3,4-diphenylisoquinolin-1(2H)-one (2k)**

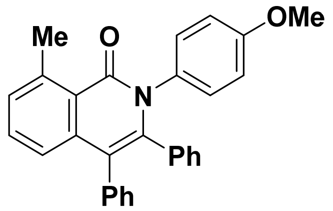


White solid, Mp = 255-256 °C. $R_f$ = 0.31 (toluene:EtOAc 10:1). Yield = 81%, m = 84.4 mg. **$^1$H NMR** (399.78 MHz, $CDCl_3$): δ 2.97 (s, 3H), 3.70 (s, 3H), 6.73 (d, *J* = 8.7, 2H), 6.86-6.93 (m, 5H), 7.01 (d, *J* = 8.7, 2H), 7.06 (d, *J* = 8.3, 1H), 7.10-7.21 (m, 5H), 7.27 (d, *J* = 6.9, 1H) 7.40 (dd, *J* = 7.8, *J* = 7.8, 1H); **$^{13}$C NMR** (100.53 MHz, $CDCl_3$): δ 24.5, 55.4, 114.1, 118.8, 124.1, 126.9, 127.2, 128.1, 130.2, 130.6, 131.0, 131.8, 131.9, 132.7, 135.2, 137.3, 139.6, 141.5, 142.6, 158.5, 163.9. **IR** (ATR): 1658 w, 1510 w, 1297 w, 1251 w, 1025 w, 773 w, 699 w. **MS** (EI+): 418 (31), 417 (M $^{+}$, 100), 416 (24) 294 (12), 210 (16). **HRMS** (EI+) Calcd for $C_{29}H_{23}NO_2$: 417.1729; Found: 417.1732.

**2-(4-methoxyphenyl)-3,4-diphenyl-7-(trifluoromethyl)isoquinolin-1(2H)-one (2l)**

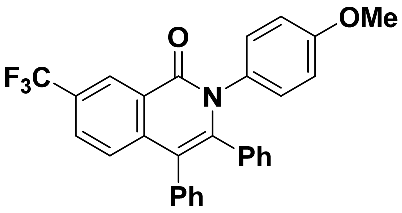


White solid, Mp = 143-144 °C. $R_f$ = 0.23 (toluene:EtOAc 10:1). Yield = 91%, m = 108.5 mg. **$^1$H NMR** (399.78 MHz, $CDCl_3$): δ 3.71 (s, 3H), 6.73 (d, *J* = 8.7, 2H), 6.86-6.90 (m, 2H), 6.91-6.95 (m, 3H), 7.00 (d, *J* = 9.2, 2H), 7.09-7.12 (m, 2H), 7.18-7.25 (m, 3H), 7.36 (d, *J* = 8.7, 1H), 7.75 (d, *J* = 8.7, 1H), 8.83 (s, 1H); **$^{13}$C NMR** (100.53 MHz, $CDCl_3$): δ 55.4, 114.1, 118.3, 124.1 (q, $J_{CF}$ = 270.7), 125.4, 126.1, 126.2, 126.6, 127.3, 127.4, 127.7, 128.3, 128.6, 128.8 (q, $J_{CF}$ = 32.0), 130.3, 130.8, 131.6, 131.8, 134.5, 135.8, 140.2, 143.9, 158.8, 162.4. **IR** (ATR): 1712 w, 1662 m, 1619 w, 1550 w, 1510 m, 1443 w, 1362 w, 1317 m, 1279 w, 1247 m, 1170 m, 1123 m, 1068 w, 1030 w, 927 w, 841 w, 794 w, 759 w, 740 w, 700 m, 676 w. **MS**

(EI+): 472 (31), 471 (M $^+$, 100), 470 (53). **HRMS** (EI+) Calcd for $C_{29}H_{20}F_3NO_2$: 471.1446; Found: 471.1450.

**2-(4-methoxyphenyl)-8-methyl-3,4-diphenylisoquinolin-1(2H)-one (2m)**

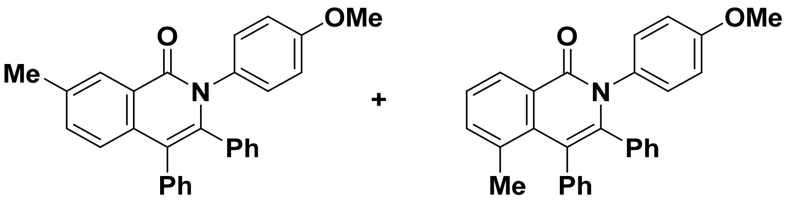

**2m** **2m'**

**2 : 1**

White solid, Mp = 223-225 °C. $R_f$ = 0.14 (toluene:EtOAc 10:1). Yield = 81%, m = 73.0 mg. **$^1$H NMR** (399.78 MHz, $CDCl_3$): δ for **2m**: 2.50 (s, 3H), 3.71 (s, 3H), 7.01 (d, *J* = 8.7, 2H), 8.36 (s, 1H). For **2m'**: 1.78 (s, 3H), 3.69 (s, 3H), 6.97 (d, *J* = 9.2, 2H), 8.53-8.56 (m, 1H); **$^{13}$C NMR** (100.53 MHz, $CDCl_3$): δ for **2m**: 21.5, 55.4, 163.0. For **2m'**: 24.0, 55.4, 163.2. **IR** (ATR): 3055 w, 1654 m, 1588 w, 1509 m, 1442 w, 1334 w, 1297 w, 1247 m, 1180 w, 1031 w, 799 w, 759 w, 738 w, 700 m. **MS** (EI+): 418 (31), 417 (M $^+$, 100), 416 (36), 210 (11). **HRMS** (EI+) Calcd for $C_{29}H_{23}NO_2$: 417.1729; Found: 417.1729.

**2-(4-methoxyphenyl)-7,8-dimethyl-3,4-diphenylisoquinolin-1(2H)-one (2n)**

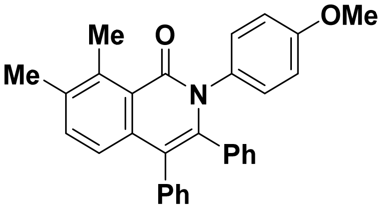

White solid, Mp = 230-231 °C. $R_f$ = 0.40 (toluene:EtOAc 10:1). Yield = 76%, m = 81.9 mg. **$^1$H NMR** (399.78 MHz, $CDCl_3$): δ 2.43 (s, 3H), 2.94 (s, 3H), 3.71 (s, 3H), 6.74 (d, *J* = 8.7, 2H), 6.86-6.93 (m, 5H), 6.97-7.03 (m, 3H), 7.10-7.22 (m, 5H), 7.35 (d, *J* = 8.7, 1H); **$^{13}$C NMR** (100.53 MHz, $CDCl_3$): δ 18.5, 21.4, 55.4, 114.1, 118.7, 123.2, 124.1, 126.8, 127.1, 127.2, 128.0, 130.6, 131.1, 131.9, 133.0, 134.3, 135.3, 136.3, 137.4, 137.8, 140.5, 140.6, 158.4, 164.2. **IR** (ATR): 3004 w, 1650 m, 1591 w, 1509 m, 1442 w, 1298 m, 1246 m, 1171 w, 1030 w, 830 w, 788 w, 699 m. **MS** (EI+): 432 (32), 431 (M $^+$, 100), 430 (16), 308 (11), 210 (16). **HRMS** (EI+) Calcd for $C_{30}H_{25}NO_2$: 431.1885; Found: 431.1888.

**5,7-dimethoxy-2-(4-methoxyphenyl)-3,4-diphenylisoquinolin-1(2H)-one (2o)**

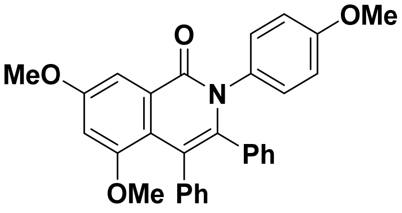


White solid, Mp = 248-250 °C. $R_f$ = 0.09 (toluene:EtOAc 10:1). Yield = 56%, m = 64.9 mg. **$^1$H NMR** (399.78 MHz, $CDCl_3$): δ 3.32 (s, 3H), 3.70 (s, 3H), 3.94 (s, 3H), 6.66 (s, 1H), 6.70 (d, *J* = 8.7, 2H), 6.80-6.89 (m, 5H), 6.95-7.06 (m, 7H), 7.66 (s, 1H); **$^{13}$C NMR** (100.53 MHz, $CDCl_3$): δ 55.4, 55.8, 55.9, 100.6, 105.3, 114.0, 116.9, 122.4, 125.5, 126.6, 127.0, 128.4, 130.4, 130.8, 131.6, 132.7, 135.2, 139.4, 140.5, 157.8, 158.5, 159.5, 162.4. **IR** (ATR): 2936 w, 1648 m, 1208 m, 1584 m, 1549 w, 1511 m, 1492 w, 1456 w, 1370 m, 1297 w, 1249 m, 1297 w, 1249 m, 1213 w, 1169 w, 1146 m, 1066 w, 1041 w, 841 w, 795 w, 770 m, 703 w. **MS** (EI+): 464 (35), 463 (M $^+$, 100), 210 (11). **HRMS** (EI+) Calcd for $C_{30}H_{25}NO_4$: 463.1784; Found: 463.1789.

**2-(4-methoxyphenyl)-3,4-diphenylbenzo[h]isoquinolin-1(2H)-one (2p)**

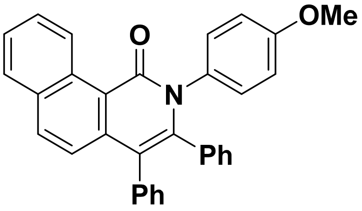


White solid, Mp = 268-270 °C. $R_f$ = 0.21 (toluene:EtOAc 10:1). Yield = 74%, m = 84.4 mg. **$^1$H NMR** (399.78 MHz, $CDCl_3$): δ 3.73 (s, 3H), 6.79 (d, *J* = 8.7, 2H), 6.92-6.95 (m, 5H), 7.09 (d, *J* = 9.2, 2H), 7.16-7.27 (m, 5H), 7.31 (d, *J* = 8.7, 1H), 7.62 (dd, *J* = 7.3, *J* = 7.3, 1H), 7.71 (dd, *J* = 7.8, *J* = 7.8, 1H), 7.89 (d, *J* = 7.6, 1H), 7.94 (d, *J* = 9.2, 1H), 10.29 (d, *J* = 8.7, 1H); **$^{13}$C NMR** (100.53 MHz, $CDCl_3$): δ 55.5, 114.2, 119.0, 119.2, 123.7, 126.6, 127.0, 127.3, 127.4, 128.0, 128.2, 128.3, 128.6, 130.5, 130.9, 132.0, 132.2, 132.4, 132.9, 134.0, 135.1, 137.1, 139.2, 143.2, 158.6, 163.4. **IR** (ATR): 3056 w, 1644 m, 1603 m, 1579 m, 1544 w, 1506 m, 1442 w, 1321 w, 1295 w, 1246 m, 1218 w, 1178 w, 1109 w, 1030 w, 840 w, 791 w, 763 m, 728 w, 699 m, 661 w. **MS** (EI+): 454 (34), 453 (M $^+$, 100), 452 (34), 330 (12), 210 (14). **HRMS** (EI+) Calcd for $C_{32}H_{23}NO_2$: 453.1729; Found: 453.1729.

**6-(4-methoxyphenyl)-4,5-diphenylfuro[2,3-c]pyridin-7(6H)-one (2q)**

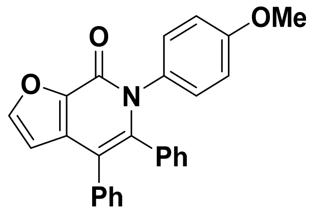

White solid, Mp = 224-225 °C. $R_f$ = 0.14 (toluene:EtOAc 10:1). Yield = 32%, m = 31.9 mg. **$^1$H NMR** (399.78 MHz, $CDCl_3$): δ 3.72 (s, 3H), 6.57 (d, *J* = 1.8, 1H), 6.73 (d, *J* = 8.7, 2H), 6.87-6.90 (m, 2H), 6.94-7.02 (m, 5H), 7.09 (d, *J* = 7.8, 2H), 7.13-7.21 (m, 3H), 7.79 (d, *J* = 1.8, 1H); **$^{13}$C NMR** (100.53 MHz, $CDCl_3$): δ 55.4, 107.7, 114.0, 114.9, 126.9, 127.5, 127.6, 128.1, 130.4, 130.6, 131.5, 131.6, 134.2, 134.5, 136.3, 142.0, 142.6, 148.5, 153.9, 158.8. **IR** (ATR): 1679 m, 1586 w, 1545 w, 1510 m, 1492 m, 1442 w, 1365 w, 1280 w, 1247 m, 1145 w, 1087 w, 1029 w, 898 w, 789 w, 761 w, 732 w, 706 w. **MS** (EI+): 394 (28), 393 ($M^+$, 100), 392 (72). **HRMS** (EI+) Calcd for $C_{26}H_{19}NO_3$: 393.1365; Found: 393.1364.

**2-(4-methoxyphenyl)-4-methyl-3-phenylisoquinolin-1(2H)-one (2r)**

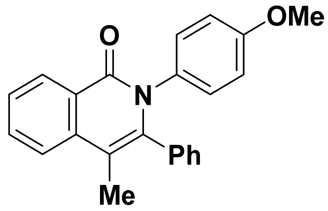

White solid, Mp = 205-206 °C. $R_f$ = 0.11 (toluene:EtOAc 10:1). Yield = 77%, m = 65.4 mg. **$^1$H NMR** (399.78 MHz, $CDCl_3$): δ 2.10 (s, 3H), 3.71 (s, 3H), 6.71 (d, *J* = 8.7, 2H), 6.94 (d, *J* = 8.7, 2H), 7.06 (d, *J* = 6.9, 2H), 7.14-7.22 (m, 3H), 7.54-7.58 (m, 1H), 7.74-7.79 (m, 2H), 8.55 (d, *J* = 7.8, 1H); **$^{13}$C NMR** (100.53 MHz, $CDCl_3$): δ 15.1, 55.4, 110.4, 114.0, 123.5, 125.9, 126.8, 127.9, 128.0, 128.7, 130.5, 130.6, 132.6, 132.7, 135.6, 137.7, 140.6, 158.5, 163.0. **IR** (ATR): 3000 w, 1651 m, 1589 m, 1509 m, 1483 m, 1442 w, 1329 w, 1298 w, 1246 m, 1172 w, 1133 w, 1106 w, 1031 m, 883 w, 823 w, 698 m. **MS** (EI+): 342 (25), 341 ($M^+$, 100), 340 (63). **HRMS** (EI+) Calcd for $C_{23}H_{19}NO_2$: 341.1416; Found: 341.1415.

**6-methoxy-2-(4-methoxyphenyl)-4-methyl-3-phenylisoquinolin-1(2H)-one (2s)**

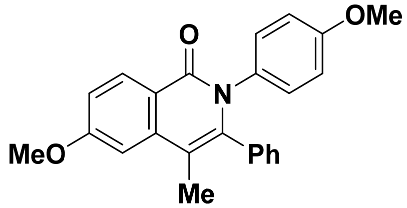

White solid, Mp = 198-200 °C. $R_f$ = 0.06 (toluene:EtOAc 10:1). Yield = 51%, m = 47.8 mg. **$^{1}$H NMR** (399.78 MHz, $CDCl_3$): δ 2.05 (s, 3H), 3.71 (s, 3H), 3.97 (s, 3H), 6.70 (d, *J* = 8.7, 2H), 6.93 (d, *J* = 8.7, 2H), 7.04-7.21 (m, 7H), 8.47 (d, *J* = 8.7, 1H); **$^{13}$C NMR** (100.53 MHz, $CDCl_3$): δ 15.2, 55.4, 55.6, 105.5, 110.0, 113.9, 115.3, 119.8, 127.9, 128.0, 130.5, 130.6, 130.9, 132.7, 135.8, 139.8, 141.4, 158.4, 162.7, 163.2. **IR** (ATR): 2360 w, 1650 m, 1604 m, 1509 m, 1485 m, 1376 w, 1324 w, 1287 w, 1247 m, 1173 m, 1105 w, 1062 w, 1031 m, 939 w, 822 w, 699 m. **MS** (EI+): 372 (26), 371 ($M^+$, 100), 370 (72), 224 (12). **HRMS** (EI+) Calcd for $C_{24}H_{21}NO_3$: 371.1521; Found: 371.1523.

**2-(4-methoxyphenyl)-4-methyl-3-phenyl-6-(trifluoromethyl)isoquinolin-1(2H)-one (2t)**

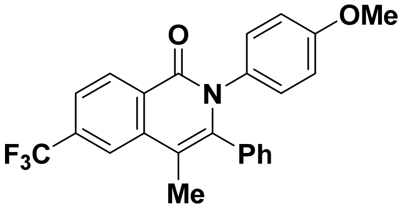


White solid, Mp = 93-96 °C. $R_f$ = 0.21 (toluene:EtOAc 10:1). Yield = 76%, m = 77.8 mg. **$^{1}$H NMR** (399.78 MHz, $CDCl_3$): δ 2.12. (s, 3H), 3.71 (s, 3H), 6.72 (d, *J* = 9.2, 2H), 6.93 (d, *J* = 9.2, 2H), 7.06 (d, *J* = 6.0, 2H), 7.17-7.24 (m, 3H), 7.74 (d, *J* = 8.7, 1H), 8.01 (s, 1H), 8.65 (d, *J* = 8.2, 1H); **$^{13}$C NMR** (100.53 MHz, $CDCl_3$): δ 15.0, 55.4, 110.2, 114.1, 114.1, 120.9, 122.7, 126.8 (q, $J_{CF}$ = 279.5), 128.2, 128.2, 129.8, 130.3, 130.4, 132.2, 134.3 (q, $J_{CF}$ = 32.0), 135.1, 137.9, 142.3, 158.7, 162.2. **IR** (ATR): 2360 w, 1658 m, 1593 w, 1564 w, 1510 m, 1434 w, 1310 m, 1280 w, 1247 m, 1169 m, 1125 m, 1278 m, 1032 w, 908 w, 821 w, 699 m. **MS** (EI+): 410 (26), 409 ($M^+$, 100), 408 (67). **HRMS** (EI+) Calcd for $C_{24}H_{18}F_3NO_2$: 409.1290; Found: 409.1292.

**2,3-bis(4-methoxyphenyl)-4-methylisoquinolin-1(2H)-one (2u)**

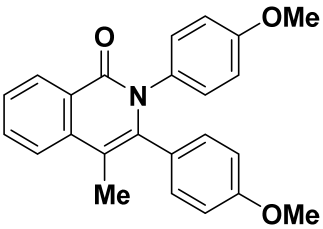


White solid, Mp = 178-180 °C. $R_f$ = 0.11 (toluene:EtOAc 10:1). Yield = 60%, m = 55.9 mg. **$^{1}$H NMR** (399.78 MHz, $CDCl_3$): δ 2.10 (s, 3H), 3.73 (s, 3H), 3.75 (s, 3H), 6.71-6.74 (m, 4H),

6.92-6.98 (m, 4H), 7.53-7.56 (m, 1H), 7.75-7.76 (m, 2H), 8.54 (d, *J* = 8.2, 1H); **$^{13}$C NMR** (100.53 MHz, $CDCl_3$): δ 15.1, 55.2, 55.4, 110.7, 113.3, 114.0, 123.5, 125.9, 126.7, 128.0, 128.7, 130.4, 131.8, 132.7, 132.8, 137.7, 140.5, 158.4, 158.8, 163.0. **IR** (ATR): 2927 w, 1711 w, 1653 m, 1611 m, 1509 m, 1484 w, 1329 w, 1290 m, 1244 s, 1175 m, 1108 w, 1031 m, 820 m, 768 m, 696 w, 664 w. **MS** (EI+): 372 (26), 371 ($M^+$, 100), 370 (38), 356 (16). **HRMS** (EI+) Calcd for $C_{24}H_{21}NO_3$: 371.1521; Found: 371.1523.

**4-butyl-2-(4-methoxyphenyl)-3-phenylisoquinolin-1(2H)-one (2v)**

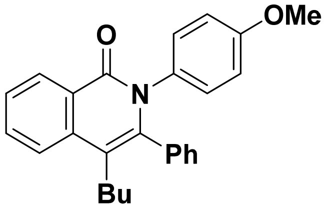


White solid, Mp = 144-145 °C. $R_f$ = 0.09 (toluene:EtOAc 10:1). Yield = 77%, m = 73.9 mg. **$^1$H NMR** (399.78 MHz, $CDCl_3$): δ 0.78 (t, *J* = 7.3, 3H), 1.20-1.26 (m, 2H), 1.48-1.52 (m, 2H), 2.42-2.46 (m, 2H), 3.70 (s, 3H), 6.70 (d, *J* = 8.7, 2H), 6.93 (d, *J* = 8.7, 2H), 7.07 (d, *J* = 8.0, 2H), 7.16-7.21 (m, 3H), 7.51-7.55 (m, 1H), 7.74-7.76 (m, 2H), 8.55 (d, *J* = 8.2, 1H); **$^{13}$C NMR** (100.53 MHz, $CDCl_3$): δ 13.8, 23.0, 28.3, 32.6, 55.4, 113.9, 113.9, 115.4, 123.6, 126.2, 126.6, 127.9, 127.9, 128.9, 130.4, 130.5, 132.6, 135.3, 136.9, 140.8, 158.4, 162.9. **IR** (ATR): 2956 w, 1712 w, 1654 m, 1589 w, 1555 w, 1510 m, 1484 w, 1360 w, 1330 w, 1297 w, 1245 m, 1174 w, 1107 w, 1032 w, 823 w, 768 m, 701 m, 660 w. **MS** (EI+): 384 (12), 383 ($M^+$, 43), 341 (25), 340 (100). **HRMS** (EI+) Calcd for $C_{26}H_{25}NO_2$: 383.1885; Found: 383.1881.

**4-(tert-butyl)-2-(4-methoxyphenyl)-3-phenylisoquinolin-1(2H)-one (2w)**

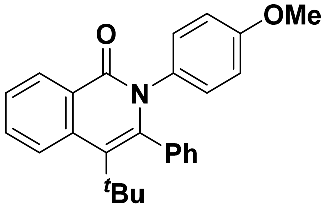


White solid, Mp = 216-217 °C. $R_f$ = 0.09 (toluene:EtOAc 10:1). Yield = 71%, m = 67.6 mg. **$^1$H NMR** (399.78 MHz, $CDCl_3$): δ 1.27 (s, 9H), 3.70 (s, 3H), 6.66 (d, *J* = 9.2, 2H), 6.81 (d, *J* = 9.2, 2H), 7.01-7.10 (m, 5H), 7.49 (dd, *J* = 7.5, *J* = 7.5, 1H), 7.69 (dd, *J* = 7.8, *J* = 7.8, 1H), 8.27 (d, *J* = 8.7, 1H), 8.56 (d, *J* = 8.0, 1H); **$^{13}$C NMR** (100.53 MHz, $CDCl_3$): δ 34.2, 36.2, 55.4, 113.8, 122.5, 126.0, 127.0, 127.1, 127.8, 128.0, 128.9, 130.7, 131.0, 132.3, 132.6, 137.4,

137.6, 140.5, 158.3, 162.6. **IR** (ATR): 2959 w, 2360 w, 1650 m, 1606 w, 1545 w, 1509 m, 1476 m, 1336 w, 1298 w, 1246 m, 1169 w, 1032 w, 821 w, 705 m. **MS** (EI+): 384(11), 383 ($M^+$, 39), 369 (27), 368 (100), 245 (14). **HRMS** (EI+) Calcd for $C_{26}H_{25}NO_2$: 383.1885; Found: 383.1881.

**2-(4-methoxyphenyl)-3,4-dipropylisoquinolin-1(2H)-one (2x)**

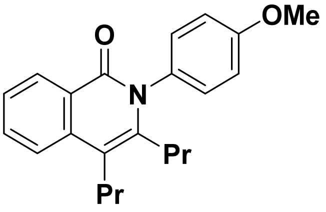


White solid, Mp = 108-114 °C. $R_f$ = 0.11 (toluene:EtOAc 10:1). Yield = 70%, m = 59.0 mg. **$^1$H NMR** (399.78 MHz, $CDCl_3$): δ 0.73 (t, *J* = 7.3, 3H), 1.10 (t, *J* = 7.3, 3H), 1.38-1.44 (m, 2H), 1.63-1.69 (m, 2H), 2.35-2.39 (m, 2H), 2.70-2.74 (m, 2H), 3.88 (s, 3H), 7.02 (d, *J* = 9.2, 2H), 7.16 (d, *J* = 8.7, 2H), 7.42-7.46 (m, 1H), 7.68-7.69 (m, 2H), 8.44 (d, *J* = 7.8, 1H); **$^{13}$C NMR** (100.53 MHz, $CDCl_3$): δ 14.4, 14.7, 23.0, 23.7, 30.0, 32.4, 55.6, 113.7, 114.7, 122.9, 125.5, 125.9, 128.7, 129.9, 132.4, 132.5, 137.2, 140.7, 159.3, 163.5. **IR** (ATR): 2961 w, 1709 w, 1651 w, 1612 w, 1589 w, 1510 w, 1463 w, 1363 w, 1297 w, 1245 w, 1092 w, 1032 w, 827 w, 768 w, 701 w. **MS** (EI+): 335 ($M^+$, 41), 307 (22), 306 (100). **HRMS** (EI+) Calcd for $C_{22}H_{25}NO_2$: 335.1885; Found: 335.1886.

**3-heptyl-2-(4-methoxyphenyl)-4-methylisoquinolin-1(2H)-one (2ya) + 4-heptyl-2-(4-methoxyphenyl)-3-methylisoquinolin-1(2H)-one (2yb)**

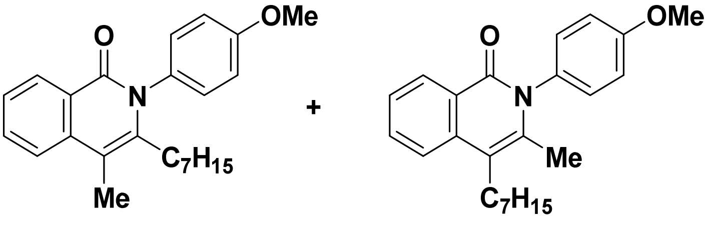


**2ya** **2yb**

**1 : 1**

Colorless oil. $R_f$ = 0.11 (toluene:EtOAc 10:1). Yield = 39%, m = 35.5 mg. **$^1$H NMR** (399.78 MHz, $CDCl_3$): δ 0.83-0.91 (m, 3H), 1.10-1.62 (c, 10H), [2.02 (s) **2yb**, 2.34 (s) **2ya**, 3H], [2.39-2.43 (m) **2ya**, 2.73-2.77 (m) **2yb**, 2H]], 3.86 (s, 3H), 7.01-7.04 (m, 2H), 7.12-7.17 (m, 2H), 7.42-7.47 (m, 2H), 7.42-7.47 (m, 1H), 7.69-7.70 (m, 2H), 8.43-8.47 (m, 1H); **$^{13}$C NMR**

(100.53 MHz, $CDCl_3$): δ 28.1, 28.6, 28.8, 29.3, 29.5, 29.9, 30.0, 30.6, 31.7, 32.0, 55.6, 108.6, 114.0, 114.7, 114.9, 122.7, 122.7, 125.1, 125.4, 125.8, 125.9, 128.5, 128.7, 129.5, 129.9, 132.4, 132.5, 132.8, 136.1, 137.1, 137.9, 140.8, 159.3, 159.3, 163.3, 163.5. **IR** (ATR): 2925 w, 1655 m, 1613 m, 1510 m, 1485 w, 1331 w, 1296 w, 1246 m, 1170 w, 1105 w, 1031 w, 825 w. **MS** (EI+): 364 (17), 363 ($M^+$, 67), 279 (53), 278 (100). **HRMS** (EI+) Calcd for $C_{24}H_{29}NO_2$: 363.2198; Found: 363.2198.

**2,3,4-triphenylisoquinolin-1(2H)-one (4a)**

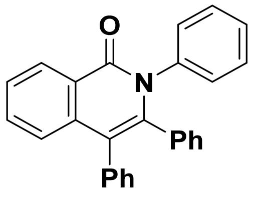


White solid, Mp = 216-218 °C. $R_f$ = 0.29 (toluene:EtOAc 10:1). Yield = 87%, m = 81.1 mg. **$^1$H NMR** (399.78 MHz, $CDCl_3$): δ 6.89 (s, 5H), 7.10-7.27 (m, 11H), 7.51-7.61 (m, 2H), 8.57 (d, $J$ = 7.8, 1H); **$^{13}$C NMR** (100.53 MHz, $CDCl_3$): δ 118.9, 125.6, 125.7, 127.0, 127.0, 127.2, 127.4, 127.7, 128.1, 128.4, 128.7, 129.6, 131.1, 131.7, 132.7, 134.9, 136.5, 137.7, 139.6, 141.2, 162.8. **IR** (ATR): 3060 w, 1655 m, 1587 w, 1554 w, 1491 m, 1442 w, 1328 m, 1251 w, 1211 w, 1141 w, 1073 w, 1030 w, 694 s. **MS** (EI+): 374 (28), 373 ($M^+$, 100), 372 (58). **HRMS** (EI+) Calcd for $C_{27}H_{19}NO$: 373.1467; Found: 373.1462.

**3,4-diphenyl-2-(4-(trifluoromethyl)phenyl)isoquinolin-1(2H)-one (4b)**

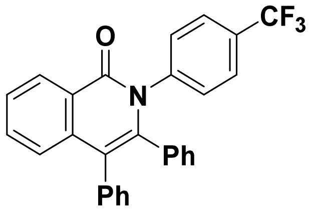


White solid, Mp = 228-229 °C. $R_f$ = 0.46 (toluene:EtOAc 10:1). Yield = 78%, m = 86.2 mg. **$^1$H NMR** (399.78 MHz, $CDCl_3$): δ 6.84-6.94 (m, 5H), 7.13-7.29 (m, 8H), 7.48 (d, $J$ = 8.2, 2H), 7.53-7.64 (m, 2H), 8.56 (d, $J$ = 7.8, 1H); **$^{13}$C NMR** (100.53 MHz, $CDCl_3$): δ 119.5, 125.5, 125.8, 125.9, 126.5 (q, $J_{CF}$ = 271.2), 127.2, 127.3, 127.6, 127.8, 128.2, 128.4, 129.7 (q, $J_{CF}$ = 32.0), 130.3, 131.0, 131.7, 133.0, 134.4, 136.2, 137.7, 140.4, 142.8, 162.6. **IR** (ATR): 2360 w, 1656 m, 1603 m, 1553 w, 1477 w, 1443 w, 1414 w, 1321 m, 1163 m, 1119 m, 1063 m, 1018

w, 920 w, 859 w, 812 w, 698 m. **MS** (EI+): 442 (30), 441 (M $^+$, 100), 440 (47). **HRMS** (EI+) Calcd for $C_{28}H_{18}F_3NO$: 441.1340; Found: 441.1337.

## 5. Procedure for Deprotection

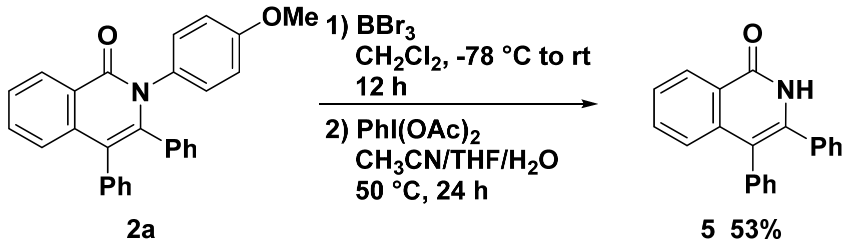

To a stirred solution of **2a** (40.3 mg, 0.1 mmol) in $CH_2Cl_2$ (3 mL) at -78 °C, $BBr_3$ was added (1.0 M in $CH_2Cl_2$, 0.4 mL, 0.4 mmol), and the resulting mixture was allowed to warm to room temperature over a period of 12 h. The reaction was quenched with 1 M aq. NaOH (5 mL) at 0 °C and neutralized with 1 M aq. HCl and the resulting mixture extracted with $CH_2Cl_2$ (3×15 mL). The combined organic layers were dried over $Na_2SO_4$, filtered, and concentrated under reduced pressure to give the crude demethylated product as a pale yellow solid. The obtained crude product was dissolved in a mixed solvent of $CH_3CN/THF/H_2O$ (5/1/2, 8 mL). To the stirred solution at 50 °C, $PhI(OAc)_2$ was added in five batches (5×19.3 mg, 0.3 mmol), and the resulting mixture was stirred at 50 °C for 24 h. The reaction was quenched by adding half-saturated aq. $NaHCO_3$ (20 mL) and the resulting mixture was extracted with $CHCl_3$/MeOH (3/1, 3×15 mL). The combined organic layers were dried over $Na_2SO_4$, filtered, and concentrated under reduced pressure. The residue was purified by column chromatography on silica gel (hexane/EtOAc = 2/1 to 3/2), giving the desired amide **5** (15.7 mg, 53% yield). An analytically pure sample was obtained by recrystallization from $CHCl_3$/hexane.

### 3,4-diphenylisoquinolin-1(2H)-one (5)

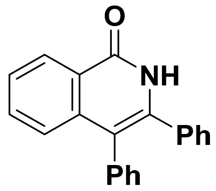

Yield = 53%, m = 15.7 mg. **$^1$H NMR** (399.78 MHz, $CDCl_3$): δ 6.99-7.38 (m, 11H), 7.51-7.63 (m, 2H), 8.51 (d, *J* = 7.8, 1H), 8.81 (br s, 1H); **$^{13}$C NMR** (100.53 MHz, $CDCl_3$): δ 117.5,

125.2, 125.8, 126.8, 127.5, 127.6, 128.6, 128.6, 128.8, 129.3, 131.9, 132.9, 135.2, 135.8, 137.1, 138.8, 162.8. **MS** (EI+): 298 (24), 297 ($M^+$, 100), 296 (44), 165 (12). **HRMS** (EI+) Calcd for $C_{21}H_{15}NO$: 297.1154; Found: 297.1150.

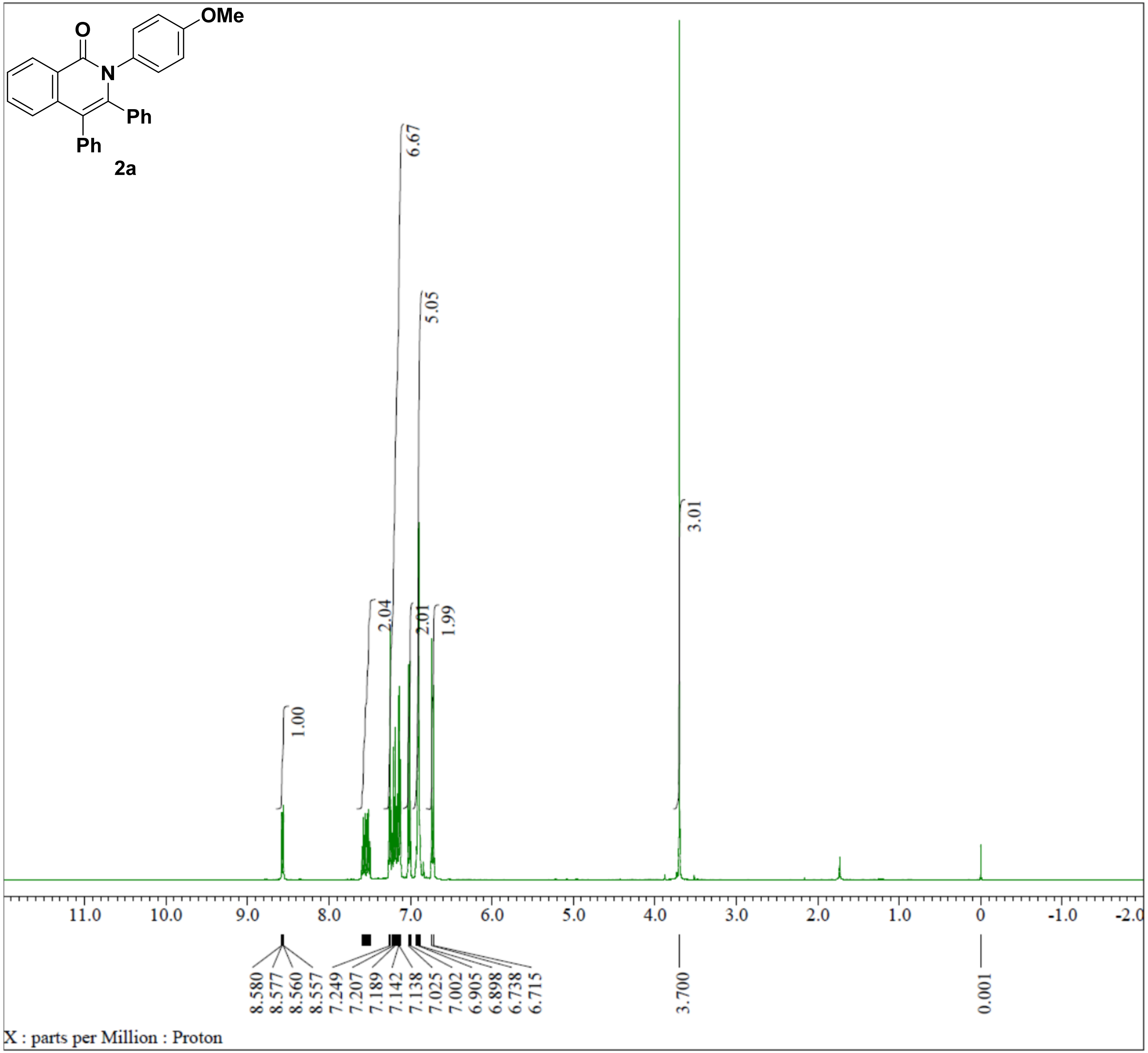


```
Filename             = AO-516 again_Proton-1-1.jd
Author               = delta
Experiment           = proton.jxp
Sample_Id            = AO-516 again
Solvent              = CHLOROFORM-D
Creation_Time        = 10-APR-2017 21:40:55
Revision_Time        = 10-APR-2017 22:05:33
Current_Time         = 21-JUN-2017 22:23:39

Comment              = AO-516 again
Data_Format          = 1D COMPLEX
Dim_Size             = 13107
Dim_Title            = Proton
Dim_Units            = [ppm]
Dimensions           = X
Site                 = JNM-ECS400
Spectrometer         = DELTA2_NMR

Field_Strength       = 9.389766[T] (400[MHz])
X_Acq_Duration       = 2.18365952[s]
X_Domain             = 1H
X_Freq               = 399.78219838[MHz]
X_Offset             = 5[ppm]
X_Points             = 16384
X_Prescans           = 1
X_Resolution         = 0.45794685[Hz]
X_Sweep              = 7.5030012[kHz]
X_Sweep_Clipped      = 6.00240096[kHz]
Irr_Domain           = Proton
Irr_Freq             = 399.78219838[MHz]
Irr_Offset           = 5[ppm]
Tri_Domain           = Proton
Tri_Freq             = 399.78219838[MHz]
Tri_Offset           = 5[ppm]
Clipped              = FALSE
Scans                = 8
Total_Scans          = 8

Relaxation_Delay     = 5[s]
Recvr_Gain           = 32
Temp_Get             = 17[dC]
X_90_Width           = 11.1[us]
X_Acq_Time           = 2.18365952[s]
X_Angle              = 45[deg]
X_Atn                = 1[dB]
X_Pulse              = 5.55[us]
Irr_Mode             = Off
Tri_Mode             = Off
Dante_Presat         = FALSE
Initial_Wait         = 1[s]
Repetition_Time      = 7.18365952[s]
```

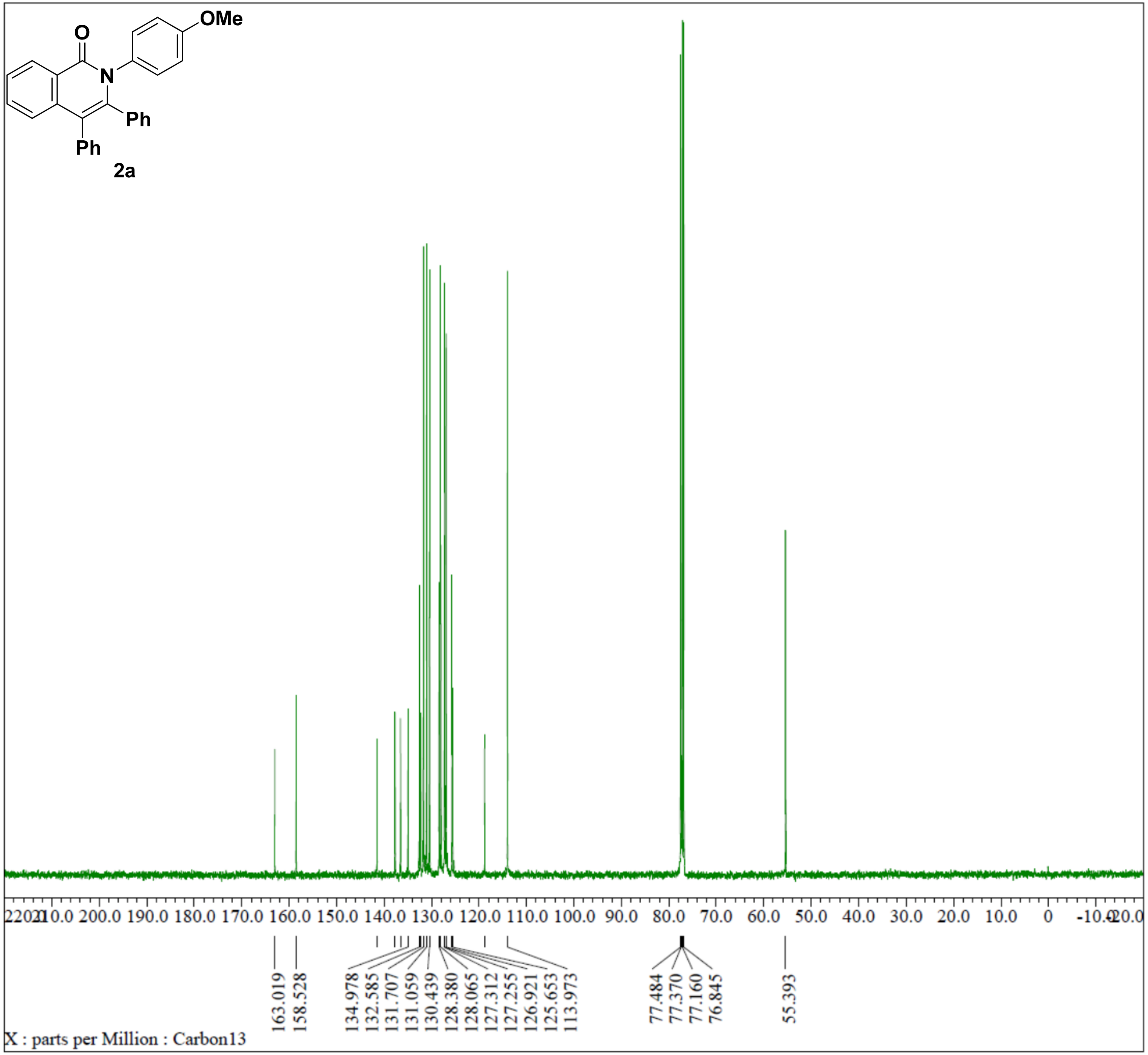


| | |
|---|---|
| Filename | = AO-516 again_Carbon-1-1.jd |
| Author | = delta |
| Experiment | = carbon.jxp |
| Sample_Id | = AO-516 again |
| Solvent | = CHLOROFORM-D |
| Creation_Time | = 11-APR-2017 02:06:40 |
| Revision_Time | = 11-APR-2017 09:44:35 |
| Current_Time | = 21-JUN-2017 22:22:36 |
| Comment | = AO-516 again |
| Data_Format | = 1D COMPLEX |
| Dim_Size | = 26214 |
| Dim_Title | = Carbon13 |
| Dim_Units | = [ppm] |
| Dimensions | = X |
| Site | = JNM-ECS400 |
| Spectrometer | = DELTA2_NMR |
| Field_Strength | = 9.389766[T] (400[MHz]) |
| X_Acq_Duration | = 1.04333312[s] |
| X_Domain | = 13C |
| X_Freq | = 100.52530333[MHz] |
| X_Offset | = 100[ppm] |
| X_Points | = 32768 |
| X_Prescans | = 4 |
| X_Resolution | = 0.95846665[Hz] |
| X_Sweep | = 31.40703518[kHz] |
| X_Sweep_Clipped | = 25.12562814[kHz] |
| Irr_Domain | = Proton |
| Irr_Freq | = 399.78219838[MHz] |
| Irr_Offset | = 5[ppm] |
| Clipped | = FALSE |
| Scans | = 1024 |
| Total_Scans | = 1024 |
| Relaxation_Delay | = 2[s] |
| Recvr_Gain | = 60 |
| Temp_Get | = 17.1[dC] |
| X_90_Width | = 9.9[us] |
| X_Acq_Time | = 1.04333312[s] |
| X_Angle | = 30[deg] |
| X_Atn | = 6[dB] |
| X_Pulse | = 3.3[us] |
| Irr_Atn_Dec | = 21.307[dB] |
| Irr_Atn_Noe | = 21.307[dB] |
| Irr_Noise | = WALTZ |
| Irr_Pwidth | = 0.115[ms] |
| Decoupling | = TRUE |
| Initial_Wait | = 1[s] |
| Noe | = TRUE |
| Noe_Time | = 2[s] |
| Repetition_Time | = 3.04333312[s] |

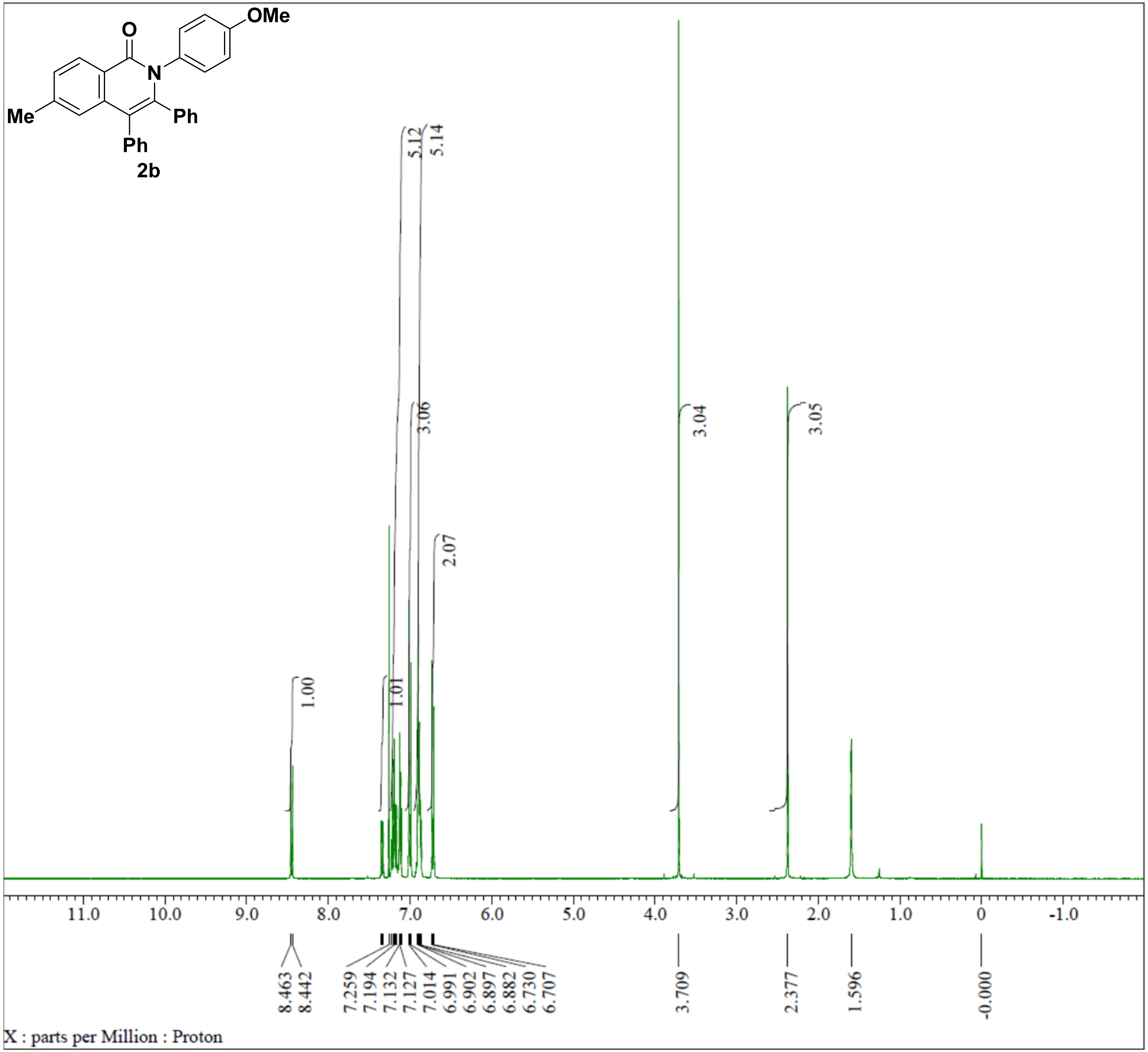


```
Filename          = AO-546 GPC_Proton-1-2.jdf
Author            = delta
Experiment        = proton.jxp
Sample_Id         = AO-546 GPC
Solvent           = CHLOROFORM-D
Creation_Time     = 17-OCT-2016 14:55:21
Revision_Time     = 22-JUN-2017 10:23:05
Current_Time      = 22-JUN-2017 10:23:11

Comment           = AO-546 GPC
Data_Format       = 1D COMPLEX
Dim_Size          = 13107
Dim_Title         = Proton
Dim_Units         = [ppm]
Dimensions        = X
Site              = JNM-ECS400
Spectrometer      = DELTA2_NMR

Field_Strength    = 9.389766[T] (400[MHz])
X_Acq_Duration    = 2.18365952[s]
X_Domain          = 1H
X_Freq            = 399.78219838[MHz]
X_Offset          = 5[ppm]
X_Points          = 16384
X_Prescans        = 1
X_Resolution      = 0.45794685[Hz]
X_Sweep           = 7.5030012[kHz]
X_Sweep_Clipped   = 6.00240096[kHz]
Irr_Domain        = Proton
Irr_Freq          = 399.78219838[MHz]
Irr_Offset        = 5[ppm]
Tri_Domain        = Proton
Tri_Freq          = 399.78219838[MHz]
Tri_Offset        = 5[ppm]
Clipped           = FALSE
Scans             = 8
Total_Scans       = 8

Relaxation_Delay  = 5[s]
Recvr_Gain        = 36
Temp_Get          = 23.6[dC]
X_90_Width        = 12.4[us]
X_Acq_Time        = 2.18365952[s]
X_Angle           = 45[deg]
X_Atn             = 1.5[dB]
X_Pulse           = 6.2[us]
Irr_Mode          = Off
Tri_Mode          = Off
Dante_Presat      = FALSE
Initial_Wait      = 1[s]
Repetition_Time   = 7.18365952[s]
```

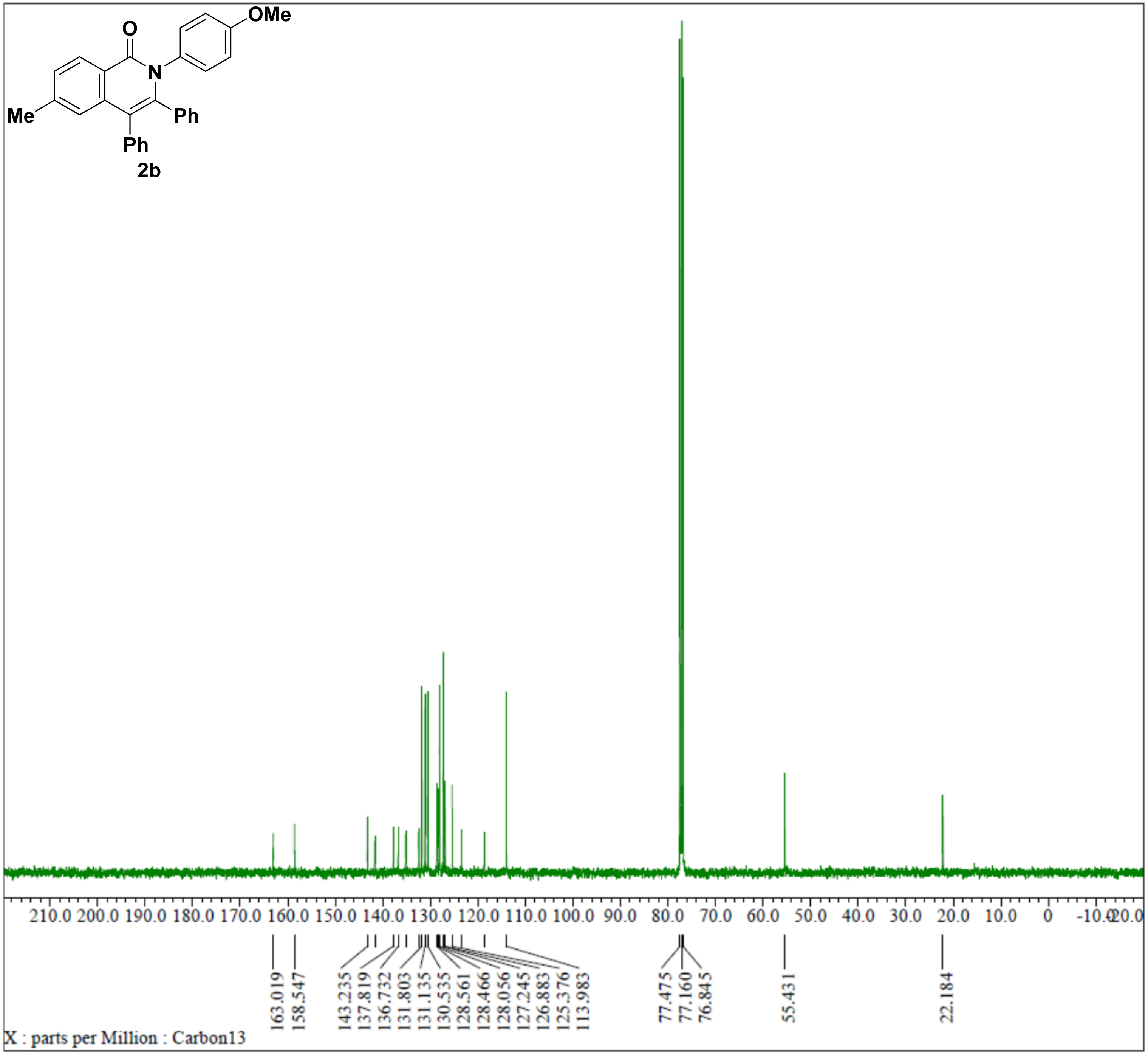


Filename = AO-546 GPC again_13C-1-1.j
Author = delta
Experiment = carbon.jxp
Sample_Id = AO-546 GPC again
Solvent = CHLOROFORM-D
Creation_Time = 18-OCT-2016 04:38:01
Revision_Time = 3-APR-2017 13:46:26
Current_Time = 21-JUN-2017 22:33:31

Comment = AO-546 GPC again_13C
Data_Format = 1D COMPLEX
Dim_Size = 26214
Dim_Title = Carbon13
Dim_Units = [ppm]
Dimensions = X
Site = JNM-ECS400
Spectrometer = DELTA2_NMR

Field_Strength = 9.389766[T] (400[MHz])
X_Acq_Duration = 1.04333312[s]
X_Domain = 13C
X_Freq = 100.52530333[MHz]
X_Offset = 100[ppm]
X_Points = 32768
X_Prescans = 4
X_Resolution = 0.95846665[Hz]
X_Sweep = 31.40703518[kHz]
X_Sweep_Clipped = 25.12562814[kHz]
Irr_Domain = Proton
Irr_Freq = 399.78219838[MHz]
Irr_Offset = 5[ppm]
Clipped = TRUE
Scans = 1024
Total_Scans = 1024

Relaxation_Delay = 2[s]
Recvr_Gain = 60
Temp_Get = 23.4[dC]
X_90_Width = 13.7[us]
X_Acq_Time = 1.04333312[s]
X_Angle = 30[deg]
X_Atn = 7[dB]
X_Pulse = 4.56666667[us]
Irr_Atn_Dec = 20.846[dB]
Irr_Atn_Noe = 20.846[dB]
Irr_Noise = WALTZ
Irr_Pwidth = 0.115[ms]
Decoupling = TRUE
Initial_Wait = 1[s]
Noe = TRUE
Noe_Time = 2[s]
Repetition_Time = 3.04333312[s]

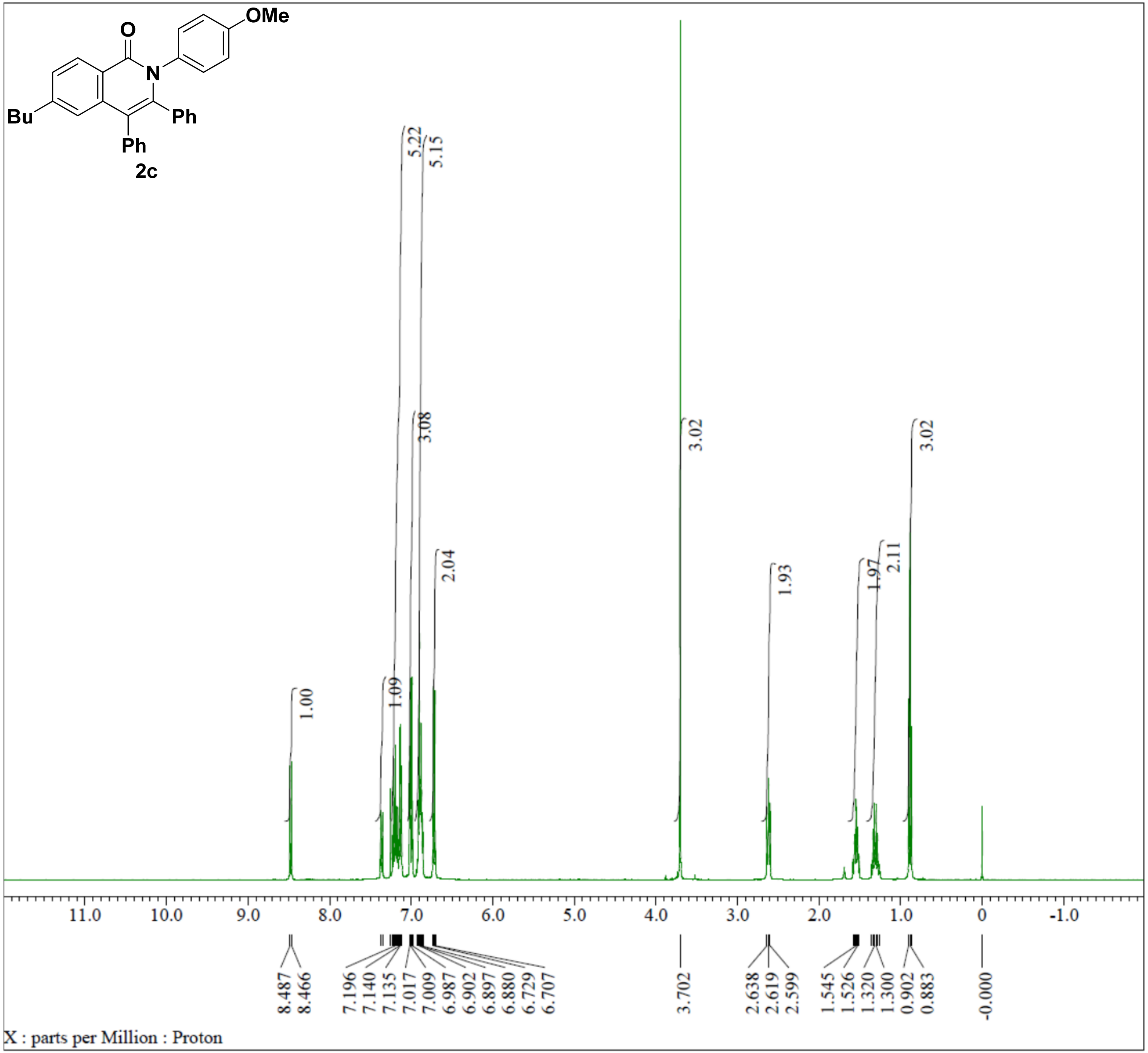


```
Filename           = AO-770_Proton-1-1.jdf
Author             = delta
Experiment         = proton.jxp
Sample_Id          = AO-770
Solvent            = CHLOROFORM-D
Creation_Time      = 25-MAR-2017 17:56:55
Revision_Time      = 27-MAR-2017 14:59:11
Current_Time       = 21-JUN-2017 22:47:25

Comment            = AO-770
Data_Format        = 1D COMPLEX
Dim_Size           = 13107
Dim_Title          = Proton
Dim_Units          = [ppm]
Dimensions         = X
Site               = JNM-ECS400
Spectrometer       = DELTA2_NMR

Field_Strength     = 9.389766[T] (400[MHz])
X_Acq_Duration     = 2.18365952[s]
X_Domain           = 1H
X_Freq             = 399.78219838[MHz]
X_Offset           = 5[ppm]
X_Points           = 16384
X_Prescans         = 1
X_Resolution       = 0.45794685[Hz]
X_Sweep            = 7.5030012[kHz]
X_Sweep_Clipped    = 6.00240096[kHz]
Irr_Domain         = Proton
Irr_Freq           = 399.78219838[MHz]
Irr_Offset         = 5[ppm]
Tri_Domain         = Proton
Tri_Freq           = 399.78219838[MHz]
Tri_Offset         = 5[ppm]
Clipped            = FALSE
Scans              = 8
Total_Scans        = 8

Relaxation_Delay   = 5[s]
Recvr_Gain         = 34
Temp_Get           = 16[dC]
X_90_Width         = 11.1[us]
X_Acq_Time         = 2.18365952[s]
X_Angle            = 45[deg]
X_Atn              = 1[dB]
X_Pulse            = 5.55[us]
Irr_Mode           = Off
Tri_Mode           = Off
Dante_Presat       = FALSE
Initial_Wait       = 1[s]
Repetition_Time    = 7.18365952[s]
```

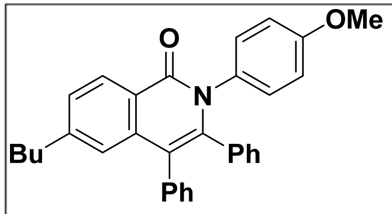


2c

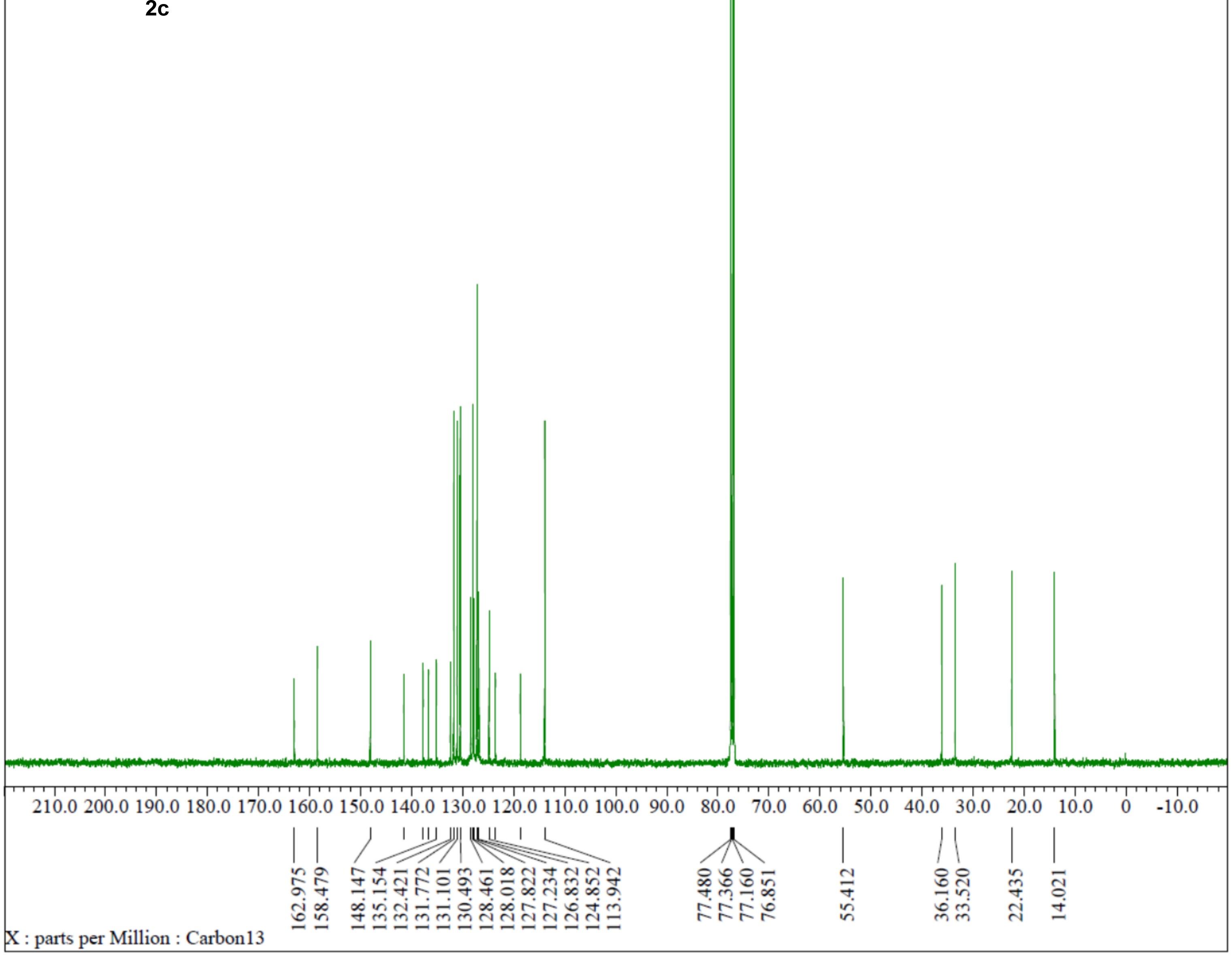


```
Filename          = AO-770_Carbon-1-1.jdf
Author            = delta
Experiment        = carbon.jxp
Sample_Id         = AO-770
Solvent           = CHLOROFORM-D
Creation_Time     = 26-MAR-2017 03:05:01
Revision_Time     =  3-APR-2017 13:20:35
Current_Time      = 21-JUN-2017 22:46:06

Comment           = AO-770
Data_Format       = 1D COMPLEX
Dim_Size          = 26214
Dim_Title         = Carbon13
Dim_Units         = [ppm]
Dimensions        = X
Site              = JNM-ECS400
Spectrometer      = DELTA2_NMR

Field_Strength    = 9.389766[T] (400[MHz])
X_Acq_Duration    = 0.96468992[s]
X_Domain          = 13C
X_Freq            = 100.52530333[MHz]
X_Offset          = 100[ppm]
X_Points          = 32768
X_Prescans        = 4
X_Resolution      = 1.03660252[Hz]
X_Sweep           = 33.9673913[kHz]
X_Sweep_Clipped   = 27.17391304[kHz]
Irr_Domain        = Proton
Irr_Freq          = 399.78219838[MHz]
Irr_Offset        = 5[ppm]
Clipped           = FALSE
Scans             = 1024
Total_Scans       = 1024

Relaxation_Delay  = 2[s]
Recvr_Gain        = 60
Temp_Get          = 16.6[dC]
X_90_Width        = 9.9[us]
X_Acq_Time        = 0.96468992[s]
X_Angle           = 30[deg]
X_Atn             = 6[dB]
X_Pulse           = 3.3[us]
Irr_Atn_Dec       = 21.307[dB]
Irr_Atn_Noe       = 21.307[dB]
Irr_Noise         = WALTZ
Irr_Pwidth        = 0.115[ms]
Decoupling        = TRUE
Initial_Wait      = 1[s]
Noe               = TRUE
Noe_Time          = 2[s]
Repetition_Time   = 2.96468992[s]
```

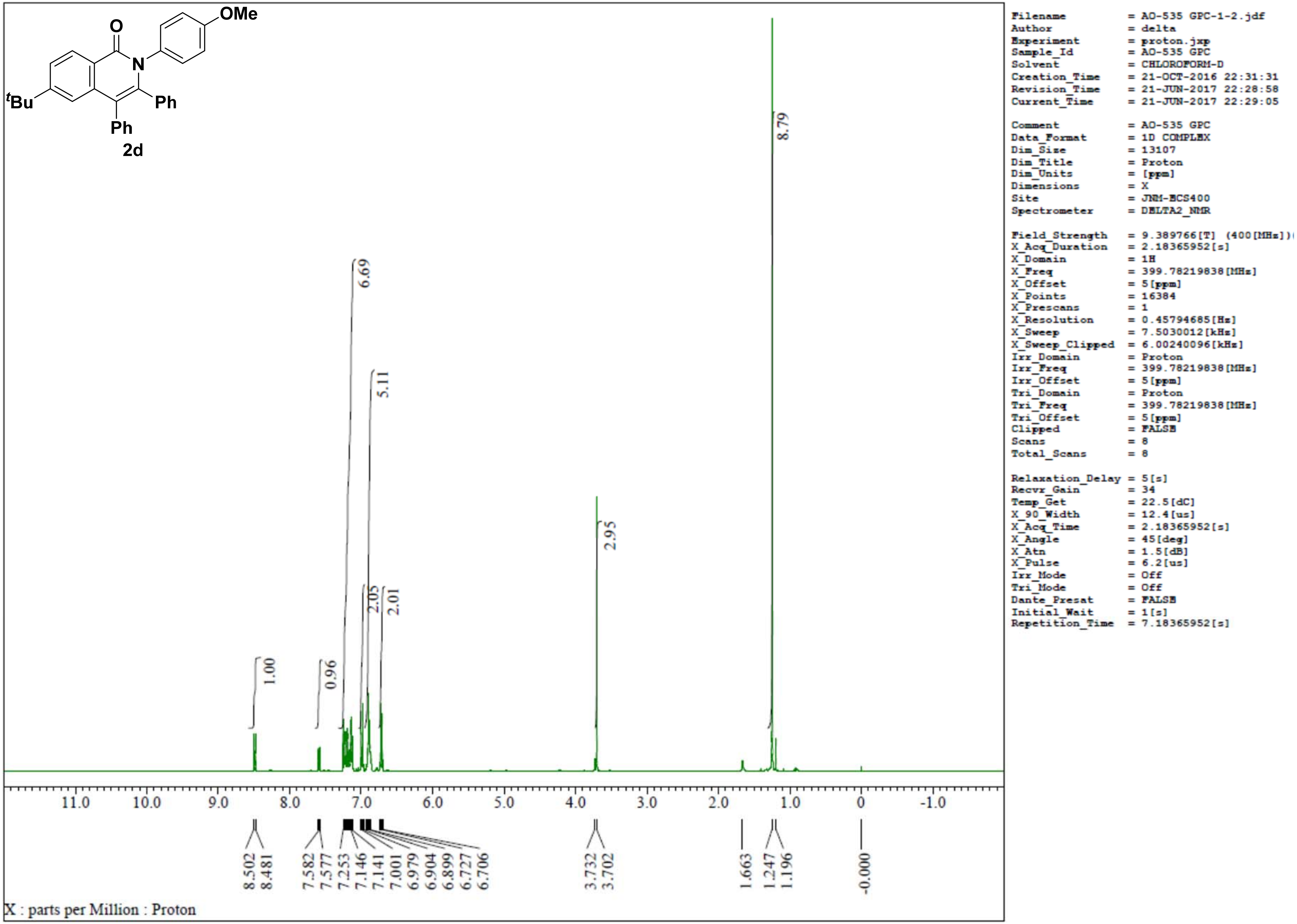
OMe
O
N
tBu
Ph
Ph
2d
1.00
0.96
6.69
5.11
2.05
2.01
2.95
8.79
11.0
10.0
9.0
8.0
7.0
6.0
5.0
4.0
3.0
2.0
1.0
0
-1.0
8.502
8.481
7.582
7.577
7.253
7.146
7.141
7.001
6.979
6.904
6.899
6.727
6.706
3.732
3.702
1.663
1.247
1.196
-0.000
X : parts per Million : Proton
Filename = AO-535 GPC-1-2.jdf
Author = delta
Experiment = proton.jxp
Sample_Id = AO-535 GPC
Solvent = CHLOROFORM-D
Creation_Time = 21-OCT-2016 22:31:31
Revision_Time = 21-JUN-2017 22:28:58
Current_Time = 21-JUN-2017 22:29:05
Comment = AO-535 GPC
Data_Format = 1D COMPLEX
Dim_Size = 13107
Dim_Title = Proton
Dim_Units = [ppm]
Dimensions = X
Site = JNM-ECS400
Spectrometer = DELTA2_NMR
Field_Strength = 9.389766[T] (400[MHz])
X_Acq_Duration = 2.18365952[s]
X_Domain = 1H
X_Freq = 399.78219838[MHz]
X_Offset = 5[ppm]
X_Points = 16384
X_Prescans = 1
X_Resolution = 0.45794685[Hz]
X_Sweep = 7.5030012[kHz]
X_Sweep_Clipped = 6.00240096[kHz]
Irr_Domain = Proton
Irr_Freq = 399.78219838[MHz]
Irr_Offset = 5[ppm]
Tri_Domain = Proton
Tri_Freq = 399.78219838[MHz]
Tri_Offset = 5[ppm]
Clipped = FALSE
Scans = 8
Total_Scans = 8
Relaxation_Delay = 5[s]
Recvr_Gain = 34
Temp_Get = 22.5[dC]
X_90_Width = 12.4[us]
X_Acq_Time = 2.18365952[s]
X_Angle = 45[deg]
X_Atn = 1.5[dB]
X_Pulse = 6.2[us]
Irr_Mode = Off
Tri_Mode = Off
Dante_Presat = FALSE
Initial_Wait = 1[s]
Repetition_Time = 7.18365952[s]

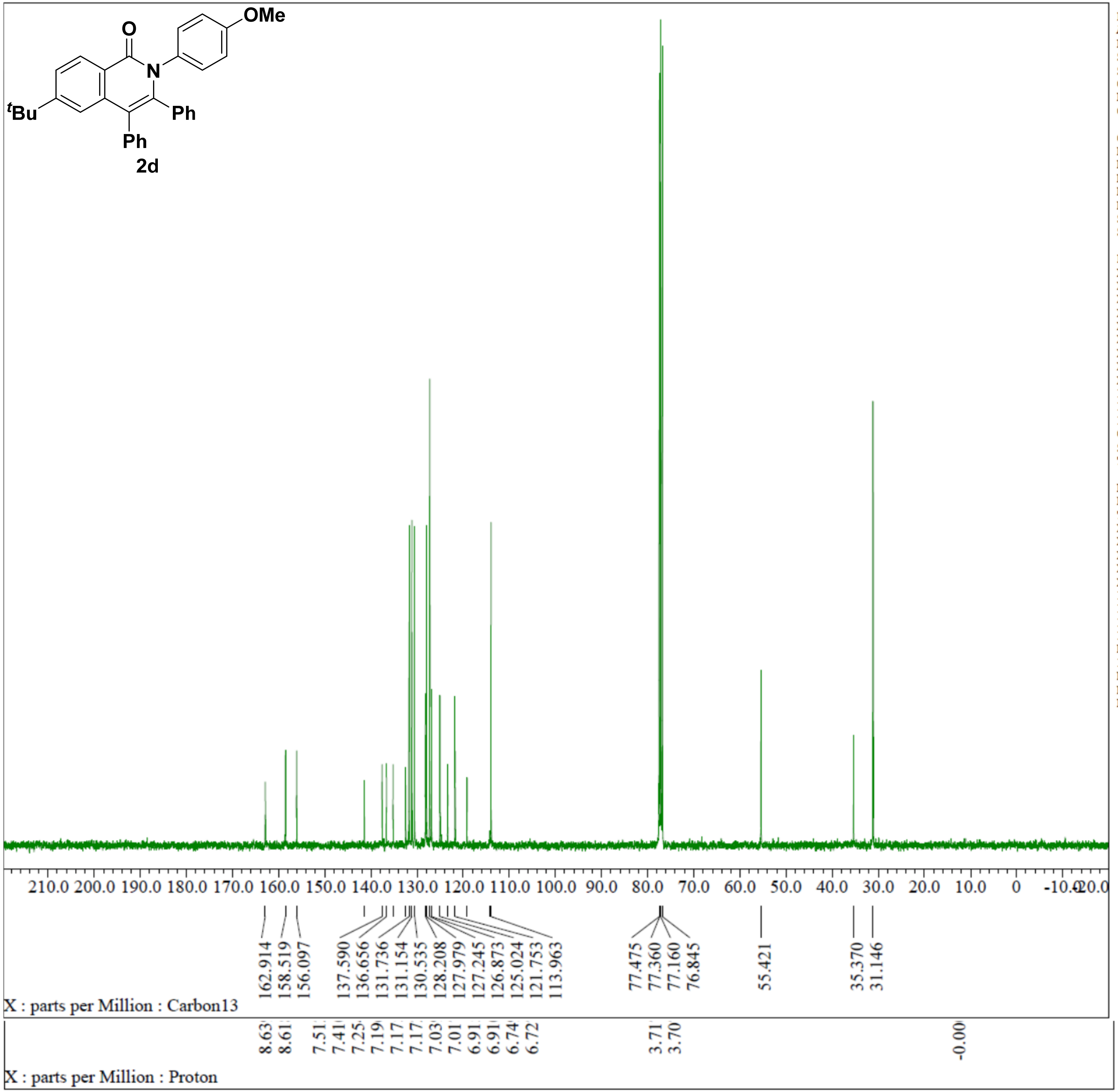


| | | |
|---|---|---|
| Filename | = | AO-535 GPC_13C_1-1-1.jdf |
| Author | = | delta |
| Experiment | = | carbon.jxp |
| Sample_Id | = | AO-535 GPC |
| Solvent | = | CHLOROFORM-D |
| Creation_Time | = | 24-OCT-2016 03:04:25 |
| Revision_Time | = | 24-OCT-2016 09:50:14 |
| Current_Time | = | 21-JUN-2017 22:30:33 |
| Comment | = | AO-535 GPC_13C_1 |
| Data_Format | = | 1D COMPLEX |
| Dim_Size | = | 26214 |
| Dim_Title | = | Carbo[illegible] |
| Dim_Units | = | [ppm] |
| Dimensions | = | X |
| Site | = | JNM-ECS400 |
| Spectrometer | = | DELTA2_NMR |
| Field_Strength | = | 9.389766[T] (400[MHz]) |
| X_Acq_Duration | = | 1.04333312[s] |
| X_Domain | = | 13C |
| X_Freq | = | 100.52530333[MHz] |
| X_Offset | = | 100[ppm] |
| X_Points | = | 32768 |
| X_Prescans | = | 4 |
| X_Resolution | = | 0.95846665[Hz] |
| X_Sweep | = | 31.40703518[kHz] |
| X_Sweep_Clipped | = | 25.12562814[kHz] |
| Irr_Domain | = | Proton |
| Irr_Freq | = | 399.78219838[MHz] |
| Irr_Offset | = | 5[ppm] |
| Clipped | = | FALSE |
| Scans | = | 1024 |
| Total_Scans | = | 1024 |
| Relaxation_Delay | = | 2[s] |
| Recvr_Gain | = | 60 |
| Temp_Get | = | 21.7[dC] |
| X_90_Width | = | 13.7[us] |
| X_Acq_Time | = | 1.04333312[s] |
| X_Angle | = | 30[deg] |
| X_Atn | = | 7[dB] |
| X_Pulse | = | 4.56666667[us] |
| Irr_Atn_Dec | = | 20.846[dB] |
| Irr_Atn_Noe | = | 20.846[dB] |
| Irr_Noise | = | WALTZ |
| Irr_Pwidth | = | 0.115[ms] |
| Decoupling | = | TRUE |
| Initial_Wait | = | 1[s] |
| Noe | = | TRUE |
| Noe_Time | = | 2[s] |
| Repetition_Time | = | 3.04333312[s] |

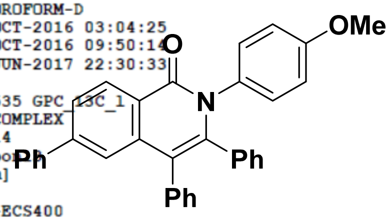


2e

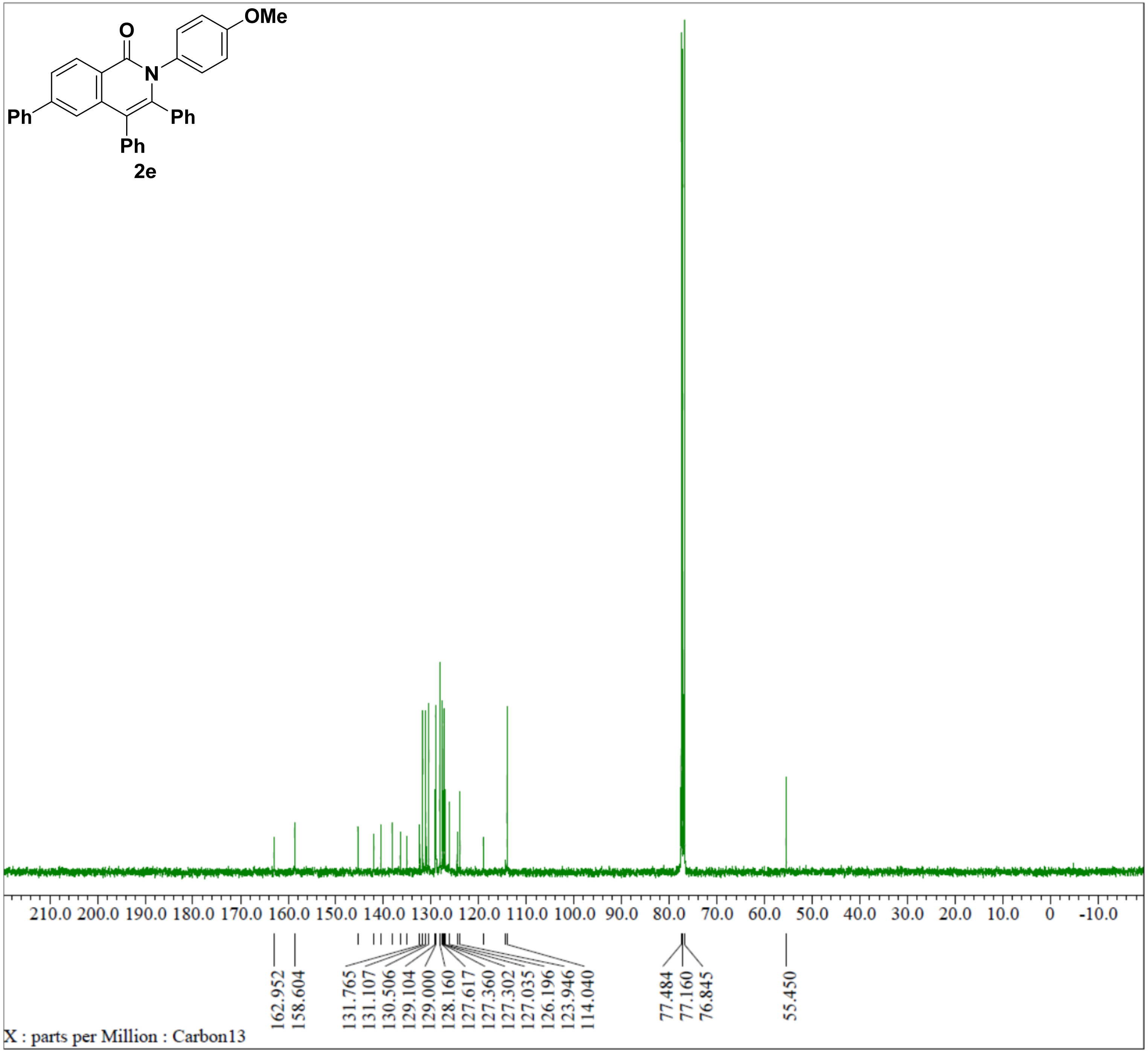


```
Filename          = AO-521 GPC_Carbon-1-2.jdf
Author            = delta
Experiment        = carbon.jxp
Sample_Id         = AO-521 GPC
Solvent           = CHLOROFORM-D
Creation_Time     =  8-NOV-2016 02:04:43
Revision_Time     =  6-APR-2017 19:48:59
Current_Time      = 21-JUN-2017 22:24:45

Comment           = AO-521 GPC_13C
Data_Format       = 1D COMPLEX
Dim_Size          = 26214
Dim_Title         = Carbon13
Dim_Units         = [ppm]
Dimensions        = X
Site              = JNM-ECS400
Spectrometer      = DELTA2_NMR

Field_Strength    = 9.389766[T] (400[MHz])
X_Acq_Duration    = 1.04333312[s]
X_Domain          = 13C
X_Freq            = 100.52530333[MHz]
X_Offset          = 100[ppm]
X_Points          = 32768
X_Prescans        = 4
X_Resolution      = 0.95846665[Hz]
X_Sweep           = 31.40703518[kHz]
X_Sweep_Clipped   = 25.12562814[kHz]
Irr_Domain        = Proton
Irr_Freq          = 399.78219838[MHz]
Irr_Offset        = 5[ppm]
Clipped           = FALSE
Scans             = 1024
Total_Scans       = 1024

Relaxation_Delay  = 2[s]
Recvr_Gain        = 60
Temp_Get          = 20.9[dC]
X_90_Width        = 13.7[us]
X_Acq_Time        = 1.04333312[s]
X_Angle           = 30[deg]
X_Atn             = 7[dB]
X_Pulse           = 4.56666667[us]
Irr_Atn_Dec       = 20.846[dB]
Irr_Atn_Noe       = 20.846[dB]
Irr_Noise         = WALTZ
Irr_Pwidth        = 0.115[ms]
Decoupling        = TRUE
Initial_Wait      = 1[s]
Noe               = TRUE
Noe_Time          = 2[s]
Repetition_Time   = 3.04333312[s]
```

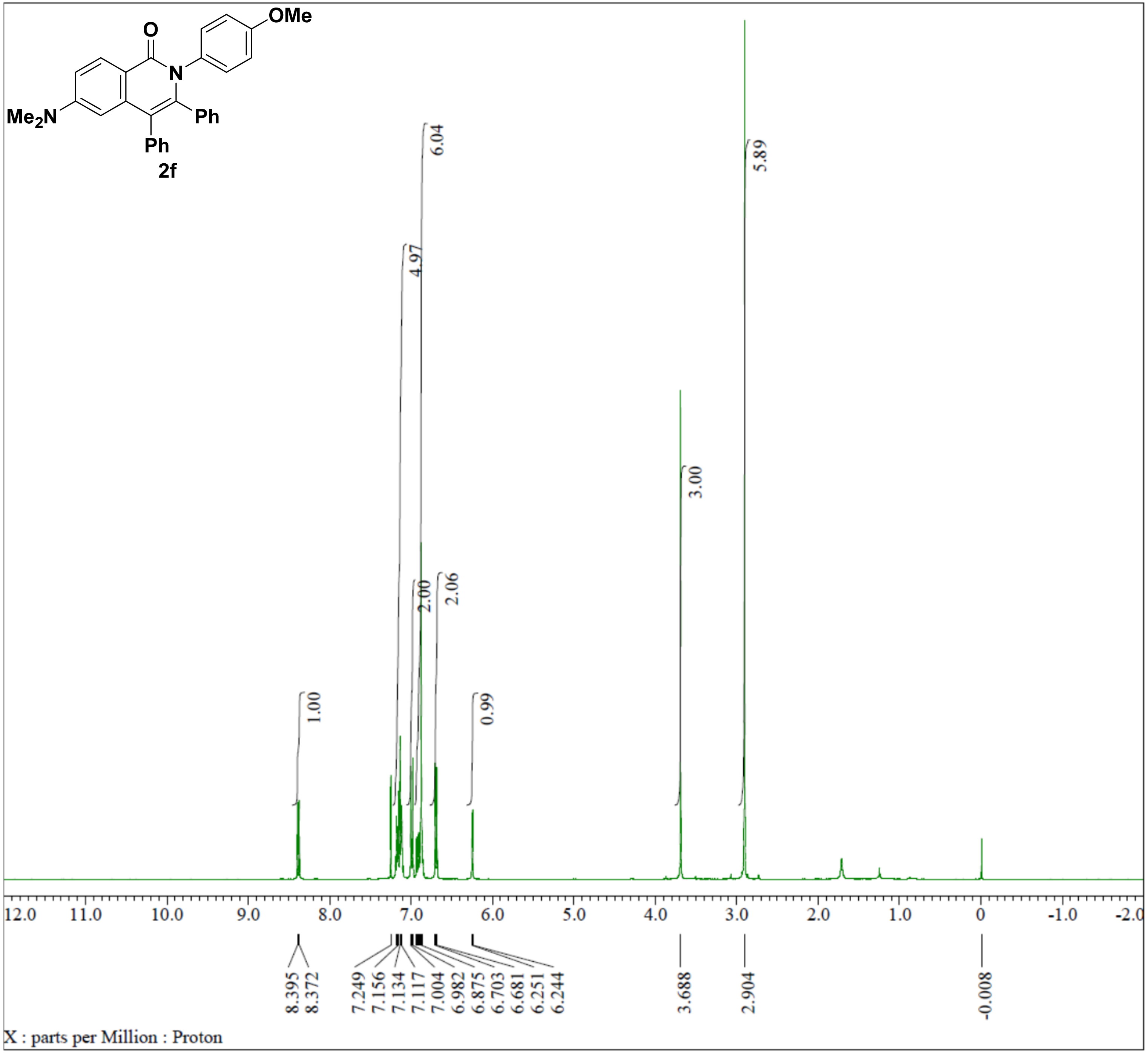


| | |
|---|---|
| Filename | = AO-576 again_Proton-1-1.jd |
| Author | = delta |
| Experiment | = proton.jxp |
| Sample_Id | = AO-576 again |
| Solvent | = CHLOROFORM-D |
| Creation_Time | = 7-APR-2017 22:27:31 |
| Revision_Time | = 8-APR-2017 11:01:44 |
| Current_Time | = 21-JUN-2017 22:32:37 |
| Comment | = AO-576 again |
| Data_Format | = 1D COMPLEX |
| Dim_Size | = 13107 |
| Dim_Title | = Proton |
| Dim_Units | = [ppm] |
| Dimensions | = X |
| Site | = JNM-ECS400 |
| Spectrometer | = DELTA2_NMR |
| Field_Strength | = 9.389766[T] (400[MHz]) |
| X_Acq_Duration | = 2.18365952[s] |
| X_Domain | = 1H |
| X_Freq | = 399.78219838[MHz] |
| X_Offset | = 5[ppm] |
| X_Points | = 16384 |
| X_Prescans | = 1 |
| X_Resolution | = 0.45794685[Hz] |
| X_Sweep | = 7.5030012[kHz] |
| X_Sweep_Clipped | = 6.00240096[kHz] |
| Irr_Domain | = Proton |
| Irr_Freq | = 399.78219838[MHz] |
| Irr_Offset | = 5[ppm] |
| Tri_Domain | = Proton |
| Tri_Freq | = 399.78219838[MHz] |
| Tri_Offset | = 5[ppm] |
| Clipped | = FALSE |
| Scans | = 8 |
| Total_Scans | = 8 |
| Relaxation_Delay | = 5[s] |
| Recvr_Gain | = 36 |
| Temp_Get | = 18.8[dC] |
| X_90_Width | = 11.1[us] |
| X_Acq_Time | = 2.18365952[s] |
| X_Angle | = 45[deg] |
| X_Atn | = 1[dB] |
| X_Pulse | = 5.55[us] |
| Irr_Mode | = Off |
| Tri_Mode | = Off |
| Dante_Presat | = FALSE |
| Initial_Wait | = 1[s] |
| Repetition_Time | = 7.18365952[s] |

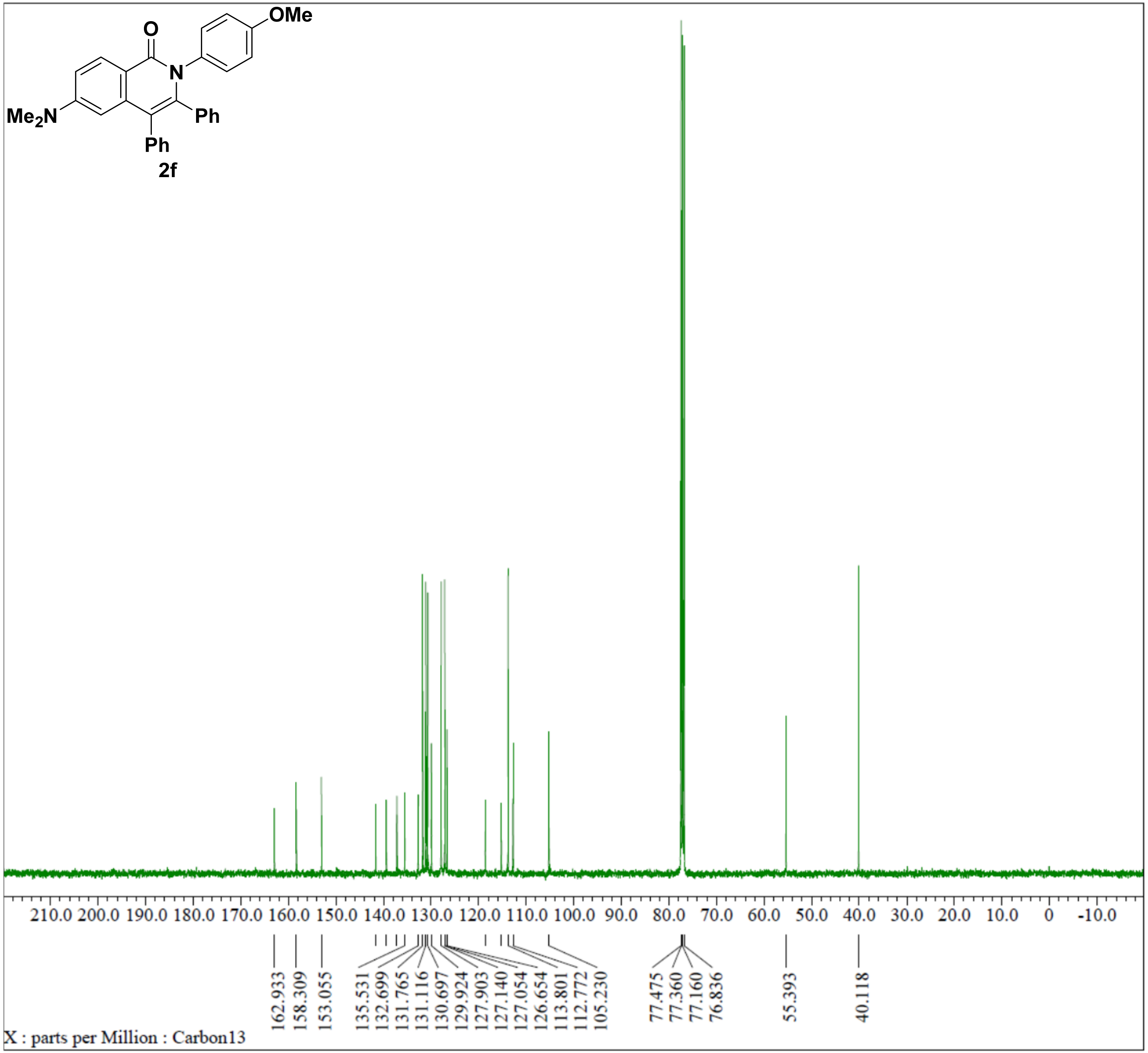


Filename = AO-576 again_Carbon-1-1.jd[illegible]
Author = delta
Experiment = carbon.jxp
Sample_Id = AO-576 again
Solvent = CHLOROFORM-D
Creation_Time = 8-APR-2017 03:06:28
Revision_Time = 8-APR-2017 11:02:54
Current_Time = 21-JUN-2017 22:35:18

Comment = AO-576 again
Data_Format = 1D COMPLEX
Dim_Size = 26214
Dim_Title = Carbon13
Dim_Units = [ppm]
Dimensions = X
Site = JNM-ECS400
Spectrometer = DELTA2_NMR

Field_Strength = 9.389766[T] (400[MHz])
X_Acq_Duration = 1.04333312[s]
X_Domain = 13C
X_Freq = 100.52530333[MHz]
X_Offset = 100[ppm]
X_Points = 32768
X_Prescans = 4
X_Resolution = 0.95846665[Hz]
X_Sweep = 31.40703518[kHz]
X_Sweep_Clipped = 25.12562814[kHz]
Irr_Domain = Proton
Irr_Freq = 399.78219838[MHz]
Irr_Offset = 5[ppm]
Clipped = FALSE
Scans = 1024
Total_Scans = 1024

Relaxation_Delay = 2[s]
Recvr_Gain = 60
Temp_Get = 18.7[dC]
X_90_Width = 9.9[us]
X_Acq_Time = 1.04333312[s]
X_Angle = 30[deg]
X_Atn = 6[dB]
X_Pulse = 3.3[us]
Irr_Atn_Dec = 21.307[dB]
Irr_Atn_Noe = 21.307[dB]
Irr_Noise = WALTZ
Irr_Pwidth = 0.115[ms]
Decoupling = TRUE
Initial_Wait = 1[s]
Noe = TRUE
Noe_Time = 2[s]
Repetition_Time = 3.04333312[s]

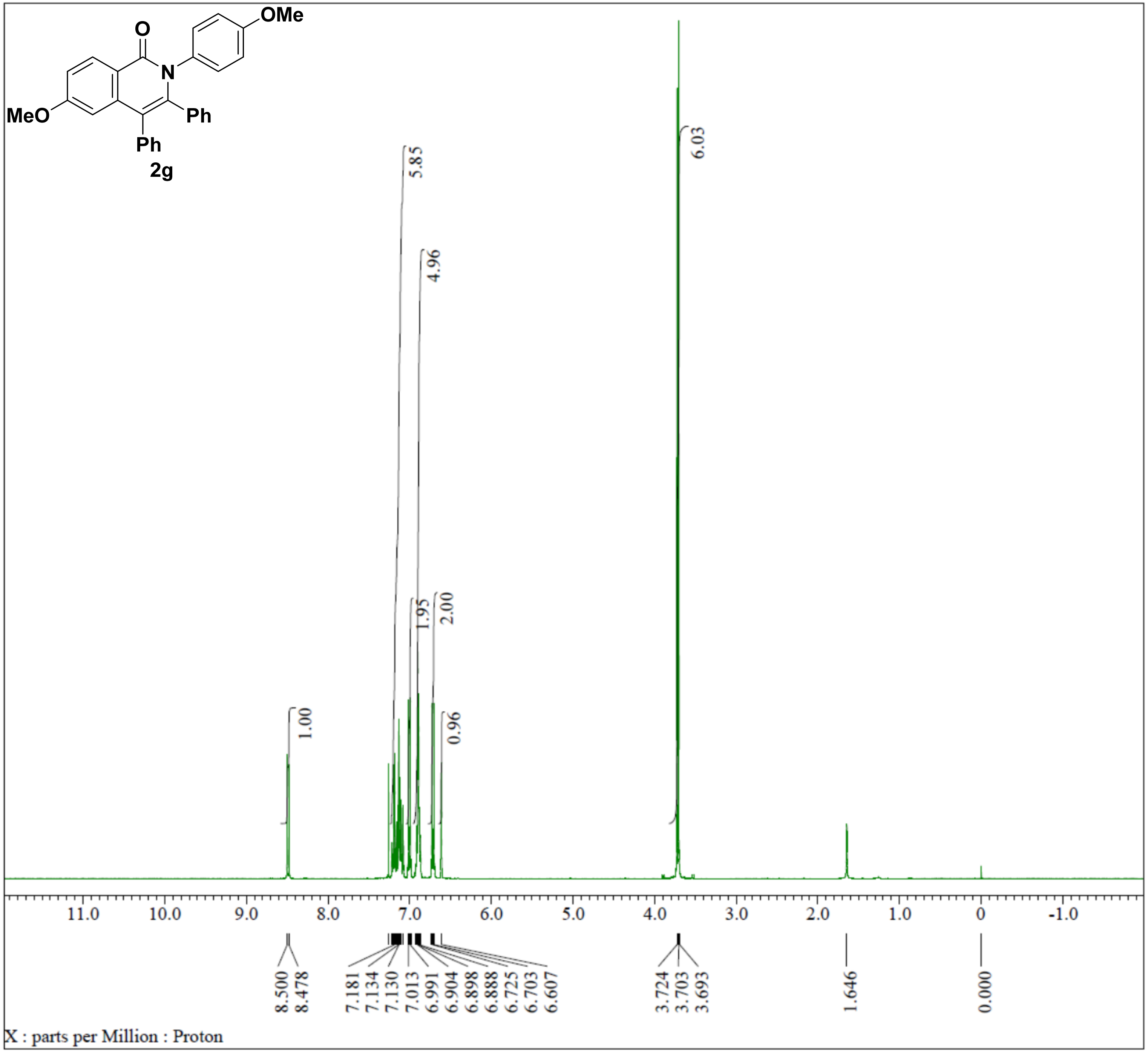


```
Filename             = AO-556 GPC-1-2.jdf
Author               = delta
Experiment           = proton.jxp
Sample_Id            = AO-556 GPC
Solvent              = CHLOROFORM-D
Creation_Time        = 21-OCT-2016 22:37:35
Revision_Time        = 22-JUN-2017 10:22:20
Current_Time         = 22-JUN-2017 10:22:24

Comment              = AO-556 GPC
Data_Format          = 1D COMPLEX
Dim_Size             = 13107
Dim_Title            = Proton
Dim_Units            = [ppm]
Dimensions           = X
Site                 = JNM-ECS400
Spectrometer         = DELTA2_NMR

Field_Strength       = 9.389766[T] (400[MHz])
X_Acq_Duration       = 2.18365952[s]
X_Domain             = 1H
X_Freq               = 399.78219838[MHz]
X_Offset             = 5[ppm]
X_Points             = 16384
X_Prescans           = 1
X_Resolution         = 0.45794685[Hz]
X_Sweep              = 7.5030012[kHz]
X_Sweep_Clipped      = 6.00240096[kHz]
Irr_Domain           = Proton
Irr_Freq             = 399.78219838[MHz]
Irr_Offset           = 5[ppm]
Tri_Domain           = Proton
Tri_Freq             = 399.78219838[MHz]
Tri_Offset           = 5[ppm]
Clipped              = FALSE
Scans                = 8
Total_Scans          = 8

Relaxation_Delay     = 5[s]
Recvr_Gain           = 36
Temp_Get             = 22.6[dC]
X_90_Width           = 12.4[us]
X_Acq_Time           = 2.18365952[s]
X_Angle              = 45[deg]
X_Atn                = 1.5[dB]
X_Pulse              = 6.2[us]
Irr_Mode             = Off
Tri_Mode             = Off
Dante_Presat         = FALSE
Initial_Wait         = 1[s]
Repetition_Time      = 7.18365952[s]
```

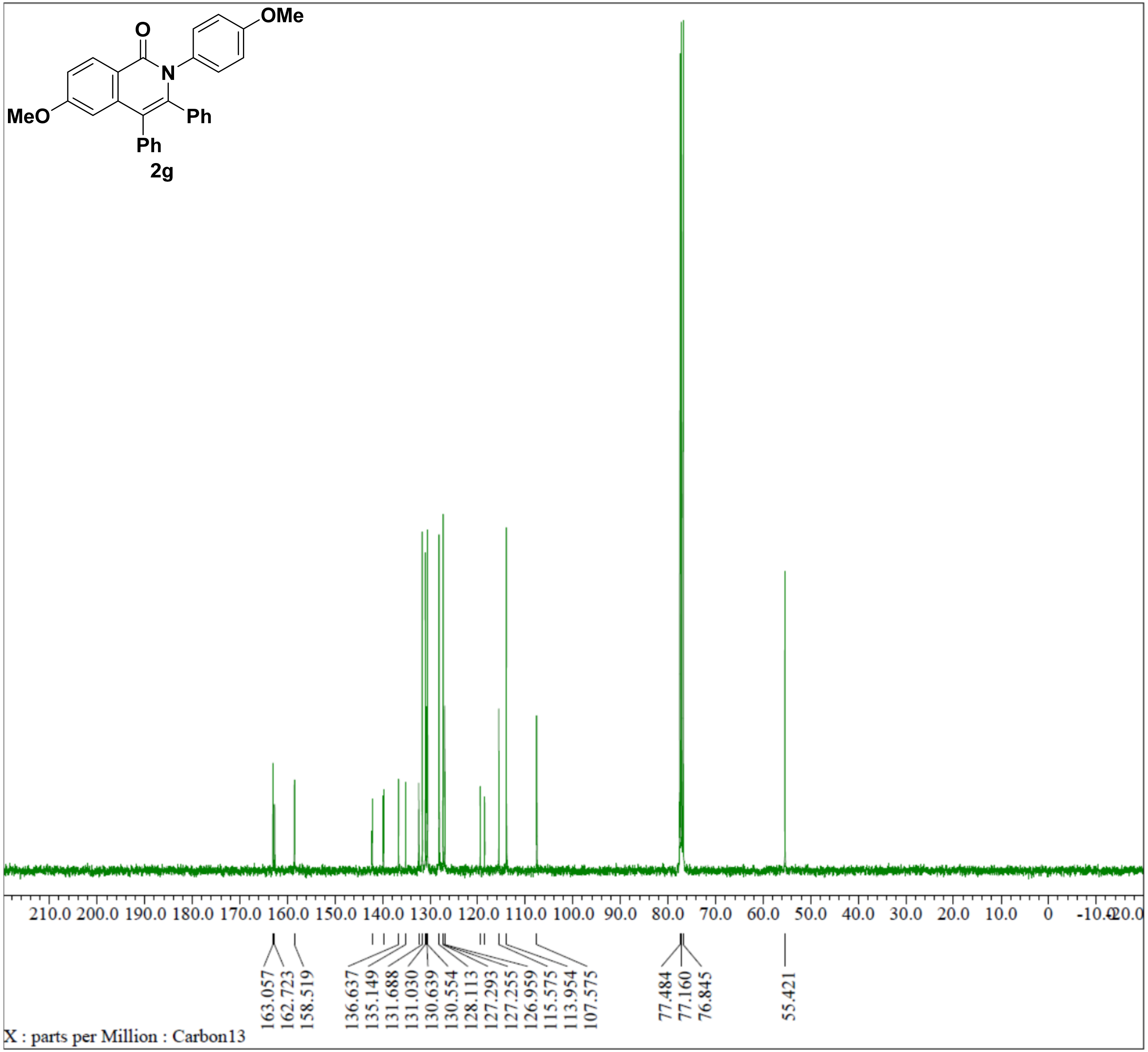


Filename = AO-556 GPC_13C_1-1-1.jdf
Author = delta
Experiment = carbon.jxp
Sample_Id = AO-556 GPC
Solvent = CHLOROFORM-D
Creation_Time = 24-OCT-2016 05:04:13
Revision_Time = 3-APR-2017 13:42:05
Current_Time = 21-JUN-2017 22:33:57

Comment = AO-556 GPC_13C_1
Data_Format = 1D COMPLEX
Dim_Size = 26214
Dim_Title = Carbon13
Dim_Units = [ppm]
Dimensions = X
Site = JNM-ECS400
Spectrometer = DELTA2_NMR

Field_Strength = 9.389766[T] (400[MHz])
X_Acq_Duration = 1.04333312[s]
X_Domain = 13C
X_Freq = 100.52530333[MHz]
X_Offset = 100[ppm]
X_Points = 32768
X_Prescans = 4
X_Resolution = 0.95846665[Hz]
X_Sweep = 31.40703518[kHz]
X_Sweep_Clipped = 25.12562814[kHz]
Irr_Domain = Proton
Irr_Freq = 399.78219838[MHz]
Irr_Offset = 5[ppm]
Clipped = FALSE
Scans = 1024
Total_Scans = 1024

Relaxation_Delay = 2[s]
Recvr_Gain = 60
Temp_Get = 21.4[dC]
X_90_Width = 13.7[us]
X_Acq_Time = 1.04333312[s]
X_Angle = 30[deg]
X_Atn = 7[dB]
X_Pulse = 4.56666667[us]
Irr_Atn_Dec = 20.846[dB]
Irr_Atn_Noe = 20.846[dB]
Irr_Noise = WALTZ
Irr_Pwidth = 0.115[ms]
Decoupling = TRUE
Initial_Wait = 1[s]
Noe = TRUE
Noe_Time = 2[s]
Repetition_Time = 3.04333312[s]

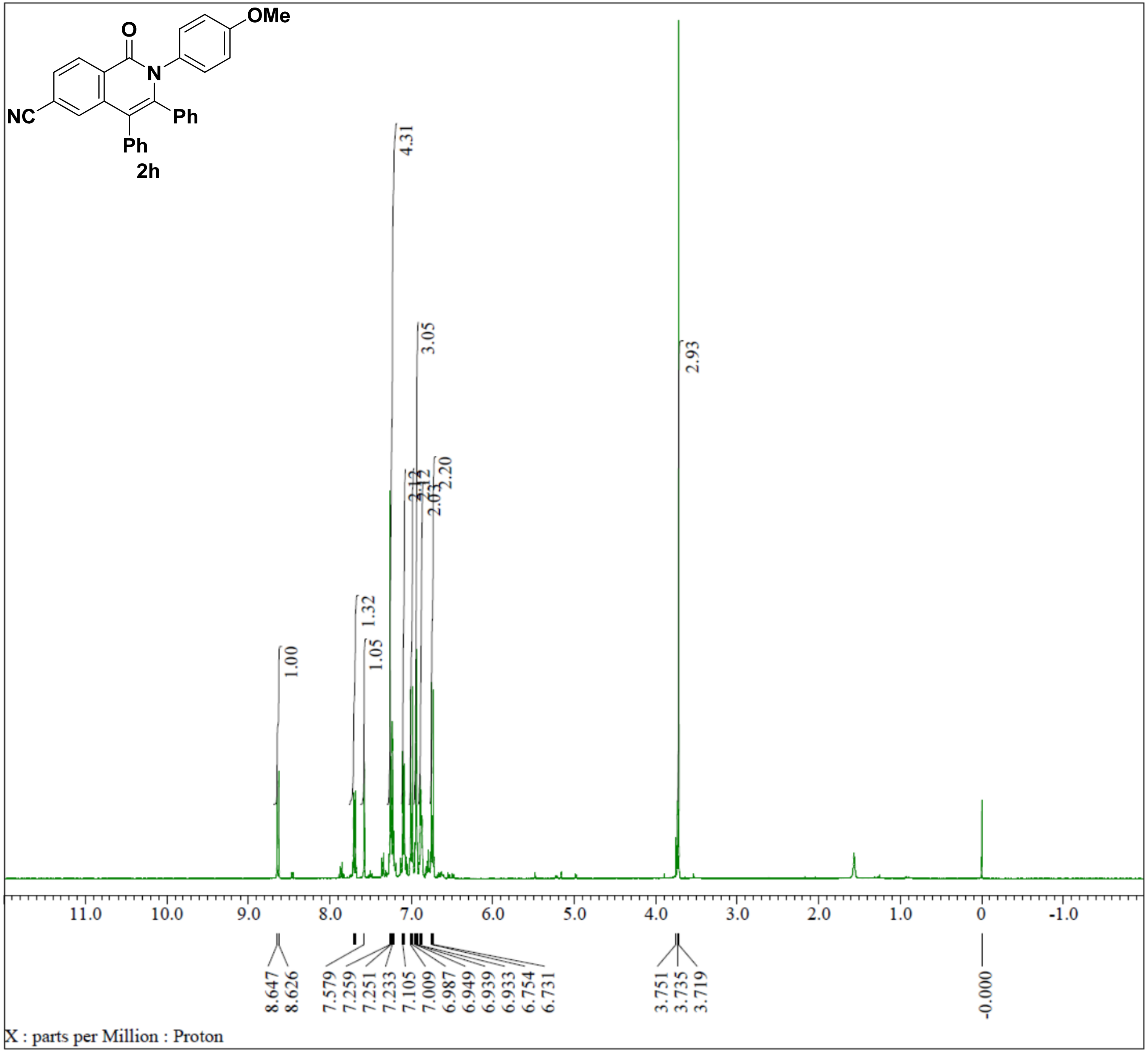


Filename = AO-660 GPC_Proton-1-2.jdf
Author = delta
Experiment = proton.jxp
Sample_Id = AO-660 GPC
Solvent = CHLOROFORM-D
Creation_Time = 25-JAN-2017 21:59:22
Revision_Time = 11-APR-2017 13:25:30
Current_Time = 21-JUN-2017 22:42:56

Comment = AO-660 GPC
Data_Format = 1D COMPLEX
Dim_Size = 13107
Dim_Title = Proton
Dim_Units = [ppm]
Dimensions = X
Site = JNM-ECS400
Spectrometer = DELTA2_NMR

Field_Strength = 9.389766[T] (400[MHz])
X_Acq_Duration = 2.18365952[s]
X_Domain = 1H
X_Freq = 399.78219838[MHz]
X_Offset = 5[ppm]
X_Points = 16384
X_Prescans = 1
X_Resolution = 0.45794685[Hz]
X_Sweep = 7.5030012[kHz]
X_Sweep_Clipped = 6.00240096[kHz]
Irr_Domain = Proton
Irr_Freq = 399.78219838[MHz]
Irr_Offset = 5[ppm]
Tri_Domain = Proton
Tri_Freq = 399.78219838[MHz]
Tri_Offset = 5[ppm]
Clipped = FALSE
Scans = 8
Total_Scans = 8

Relaxation_Delay = 5[s]
Recvr_Gain = 44
Temp_Get = 25[dC]
X_90_Width = 11.1[us]
X_Acq_Time = 2.18365952[s]
X_Angle = 45[deg]
X_Atn = 1[dB]
X_Pulse = 5.55[us]
Irr_Mode = Off
Tri_Mode = Off
Dante_Presat = FALSE
Initial_Wait = 1[s]
Repetition_Time = 7.18365952[s]

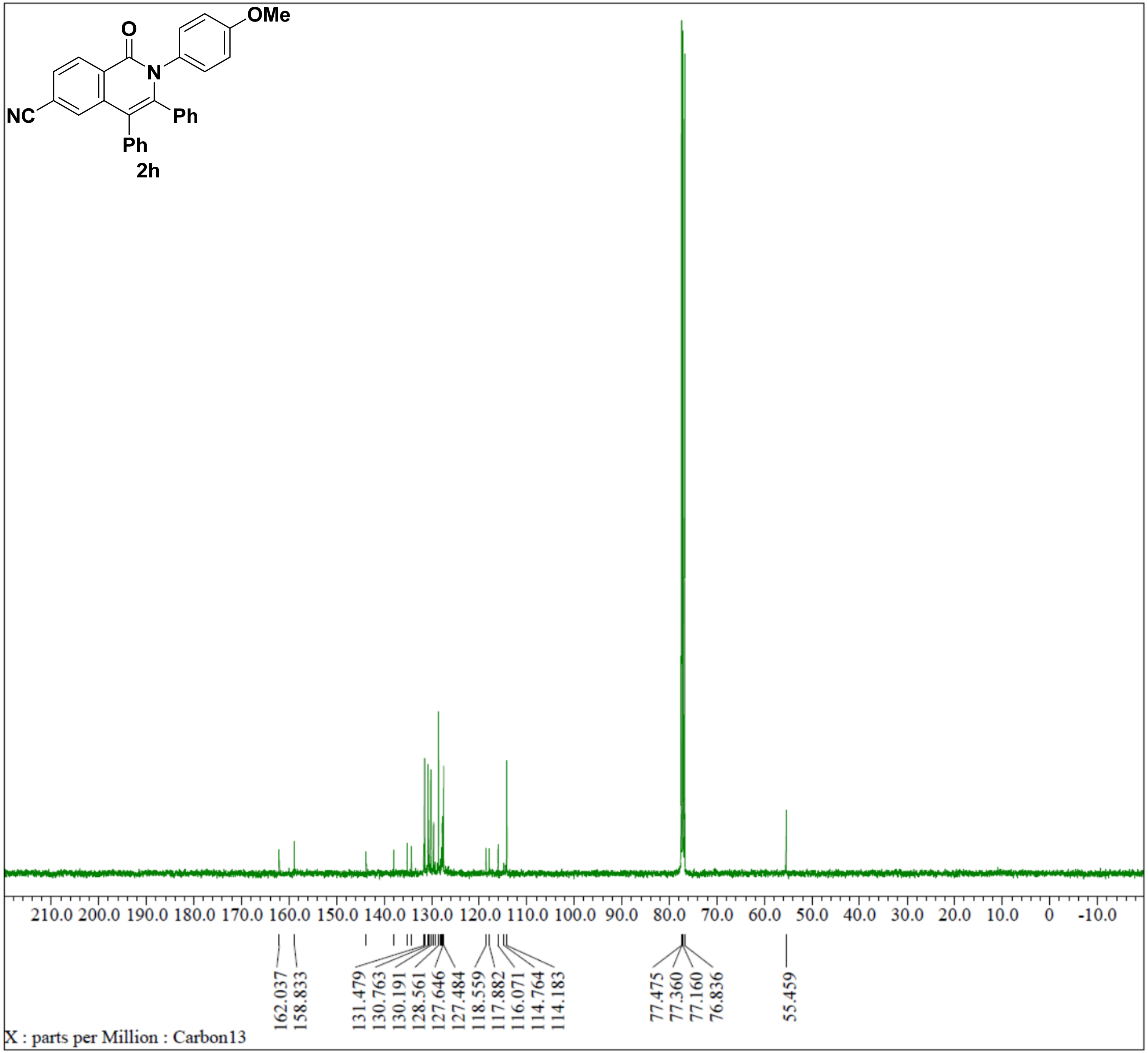


| | |
|---|---|
| Filename | = AO-660 GPC_Carbon-1-1.jdf |
| Author | = delta |
| Experiment | = carbon.jxp |
| Sample_Id | = AO-660 GPC |
| Solvent | = CHLOROFORM-D |
| Creation_Time | = 27-JAN-2017 03:04:38 |
| Revision_Time | = 3-APR-2017 13:33:13 |
| Current_Time | = 21-JUN-2017 22:42:23 |
| Comment | = AO-660 GPC_13C |
| Data_Format | = 1D COMPLEX |
| Dim_Size | = 26214 |
| Dim_Title | = Carbon13 |
| Dim_Units | = [ppm] |
| Dimensions | = X |
| Site | = JNM-ECS400 |
| Spectrometer | = DELTA2_NMR |
| Field_Strength | = 9.389766[T] (400[MHz]) |
| X_Acq_Duration | = 1.04333312[s] |
| X_Domain | = 13C |
| X_Freq | = 100.52530333[MHz] |
| X_Offset | = 100[ppm] |
| X_Points | = 32768 |
| X_Prescans | = 4 |
| X_Resolution | = 0.95846665[Hz] |
| X_Sweep | = 31.40703518[kHz] |
| X_Sweep_Clipped | = 25.12562814[kHz] |
| Irr_Domain | = Proton |
| Irr_Freq | = 399.78219838[MHz] |
| Irr_Offset | = 5[ppm] |
| Clipped | = FALSE |
| Scans | = 1024 |
| Total_Scans | = 1024 |
| Relaxation_Delay | = 2[s] |
| Recvr_Gain | = 60 |
| Temp_Get | = 14.9[dC] |
| X_90_Width | = 9.9[us] |
| X_Acq_Time | = 1.04333312[s] |
| X_Angle | = 30[deg] |
| X_Atn | = 6[dB] |
| X_Pulse | = 3.3[us] |
| Irr_Atn_Dec | = 21.307[dB] |
| Irr_Atn_Noe | = 21.307[dB] |
| Irr_Noise | = WALTZ |
| Irr_Pwidth | = 0.115[ms] |
| Decoupling | = TRUE |
| Initial_Wait | = 1[s] |
| Noe | = TRUE |
| Noe_Time | = 2[s] |
| Repetition_Time | = 3.04333312[s] |

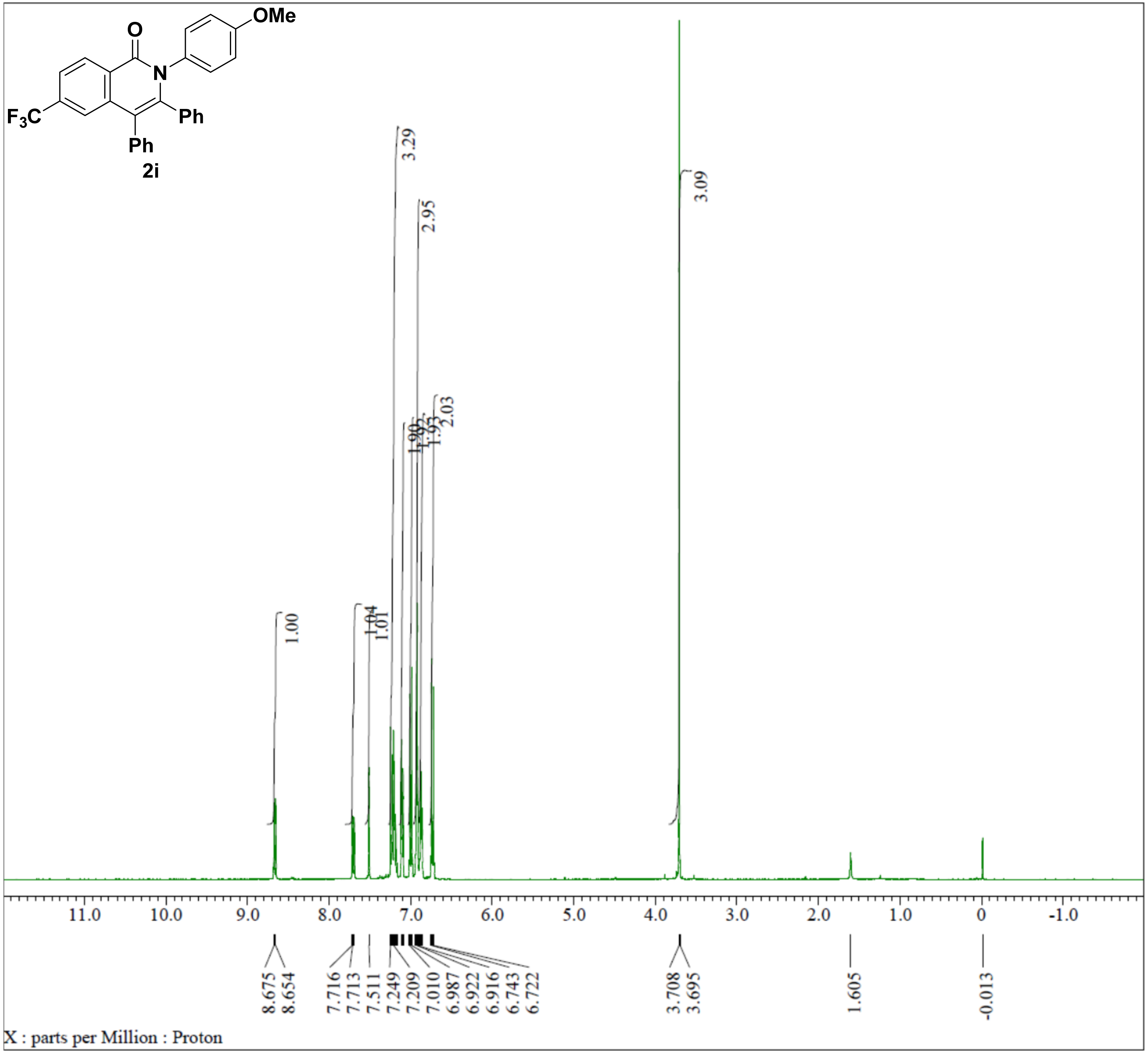


```
Filename            = AO-643 GPC_Proton-1-2.jdf
Author              = delta
Experiment          = proton.jxp
Sample_Id           = AO-643 GPC
Solvent             = CHLOROFORM-D
Creation_Time       = 25-JAN-2017 21:52:40
Revision_Time       = 22-JUN-2017 10:21:32
Current_Time        = 22-JUN-2017 10:21:36

Comment             = AO-643 GPC
Data_Format         = 1D COMPLEX
Dim_Size            = 13107
Dim_Title           = Proton
Dim_Units           = [ppm]
Dimensions          = X
Site                = JNM-ECS400
Spectrometer        = DELTA2_NMR

Field_Strength      = 9.389766[T] (400[MHz])
X_Acq_Duration      = 2.18365952[s]
X_Domain            = 1H
X_Freq              = 399.78219838[MHz]
X_Offset            = 5[ppm]
X_Points            = 16384
X_Prescans          = 1
X_Resolution        = 0.45794685[Hz]
X_Sweep             = 7.5030012[kHz]
X_Sweep_Clipped     = 6.00240096[kHz]
Irr_Domain          = Proton
Irr_Freq            = 399.78219838[MHz]
Irr_Offset          = 5[ppm]
Tri_Domain          = Proton
Tri_Freq            = 399.78219838[MHz]
Tri_Offset          = 5[ppm]
Clipped             = FALSE
Scans               = 8
Total_Scans         = 8

Relaxation_Delay    = 5[s]
Recvr_Gain          = 42
Temp_Get            = 15.9[dC]
X_90_Width          = 11.1[us]
X_Acq_Time          = 2.18365952[s]
X_Angle             = 45[deg]
X_Atn               = 1[dB]
X_Pulse             = 5.55[us]
Irr_Mode            = Off
Tri_Mode            = Off
Dante_Presat        = FALSE
Initial_Wait        = 1[s]
Repetition_Time     = 7.18365952[s]
```

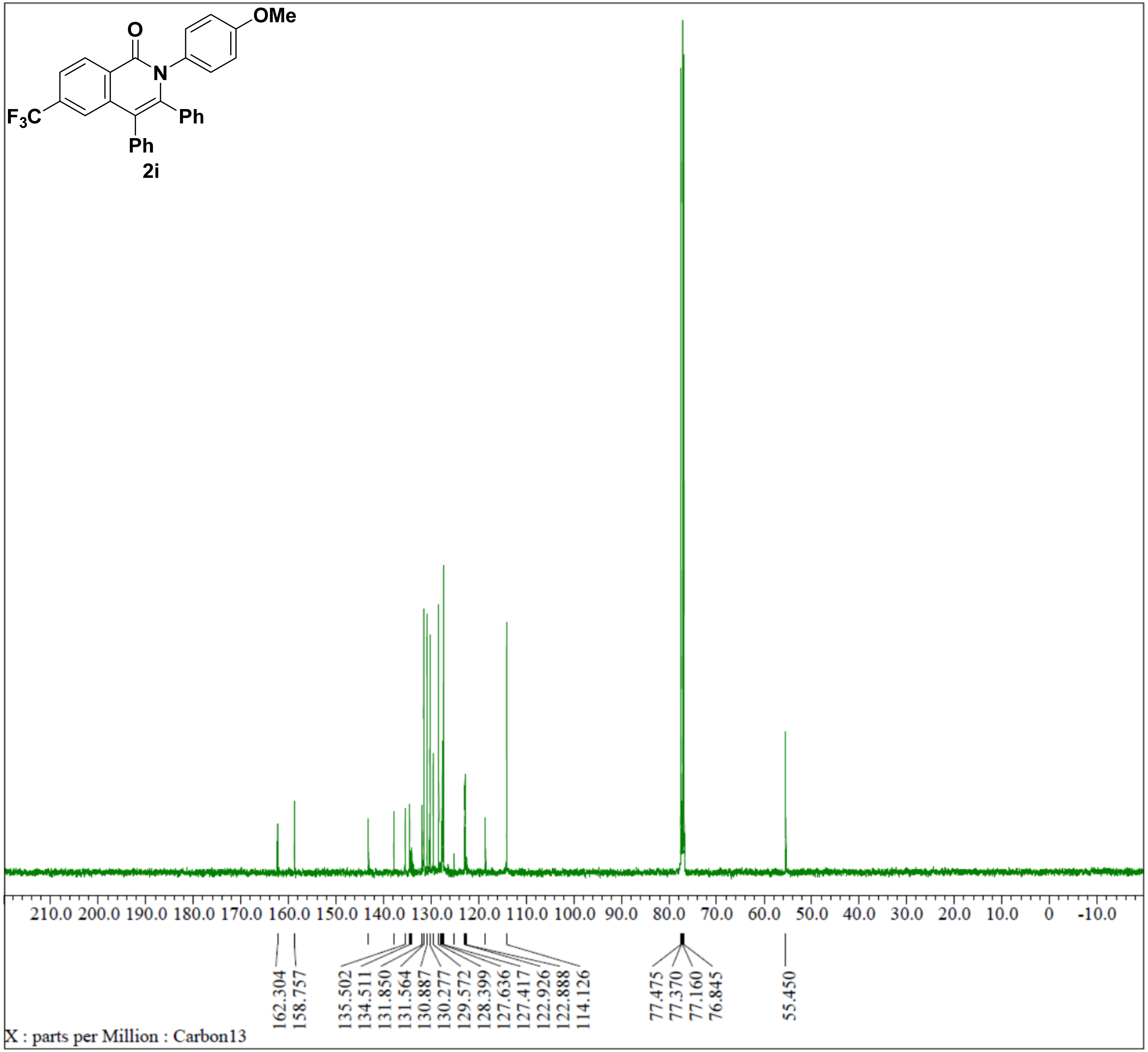


```
Filename           = AO-643 GPC_Carbon-1-2.jdf
Author             = delta
Experiment         = carbon.jxp
Sample_Id          = AO-643 GPC
Solvent            = CHLOROFORM-D
Creation_Time      = 27-JAN-2017 02:06:22
Revision_Time      = 22-JUN-2017 00:33:08
Current_Time       = 22-JUN-2017 00:33:24

Comment            = AO-643 GPC_13C
Data_Format        = 1D COMPLEX
Dim_Size           = 26214
Dim_Title          = Carbon13
Dim_Units          = [ppm]
Dimensions         = X
Site               = JNM-ECS400
Spectrometer       = DELTA2_NMR

Field_Strength     = 9.389766[T] (400[MHz])
X_Acq_Duration     = 1.04333312[s]
X_Domain           = 13C
X_Freq             = 100.52530333[MHz]
X_Offset           = 100[ppm]
X_Points           = 32768
X_Prescans         = 4
X_Resolution       = 0.95846665[Hz]
X_Sweep            = 31.40703518[kHz]
X_Sweep_Clipped    = 25.12562814[kHz]
Irr_Domain         = Proton
Irr_Freq           = 399.78219838[MHz]
Irr_Offset         = 5[ppm]
Clipped            = FALSE
Scans              = 1024
Total_Scans        = 1024

Relaxation_Delay   = 2[s]
Recvr_Gain         = 60
Temp_Get           = 15.1[dC]
X_90_Width         = 9.9[us]
X_Acq_Time         = 1.04333312[s]
X_Angle            = 30[deg]
X_Atn              = 6[dB]
X_Pulse            = 3.3[us]
Irr_Atn_Dec        = 21.307[dB]
Irr_Atn_Noe        = 21.307[dB]
Irr_Noise          = WALTZ
Irr_Pwidth         = 0.115[ms]
Decoupling         = TRUE
Initial_Wait       = 1[s]
Noe                = TRUE
Noe_Time           = 2[s]
Repetition_Time    = 3.04333312[s]
```

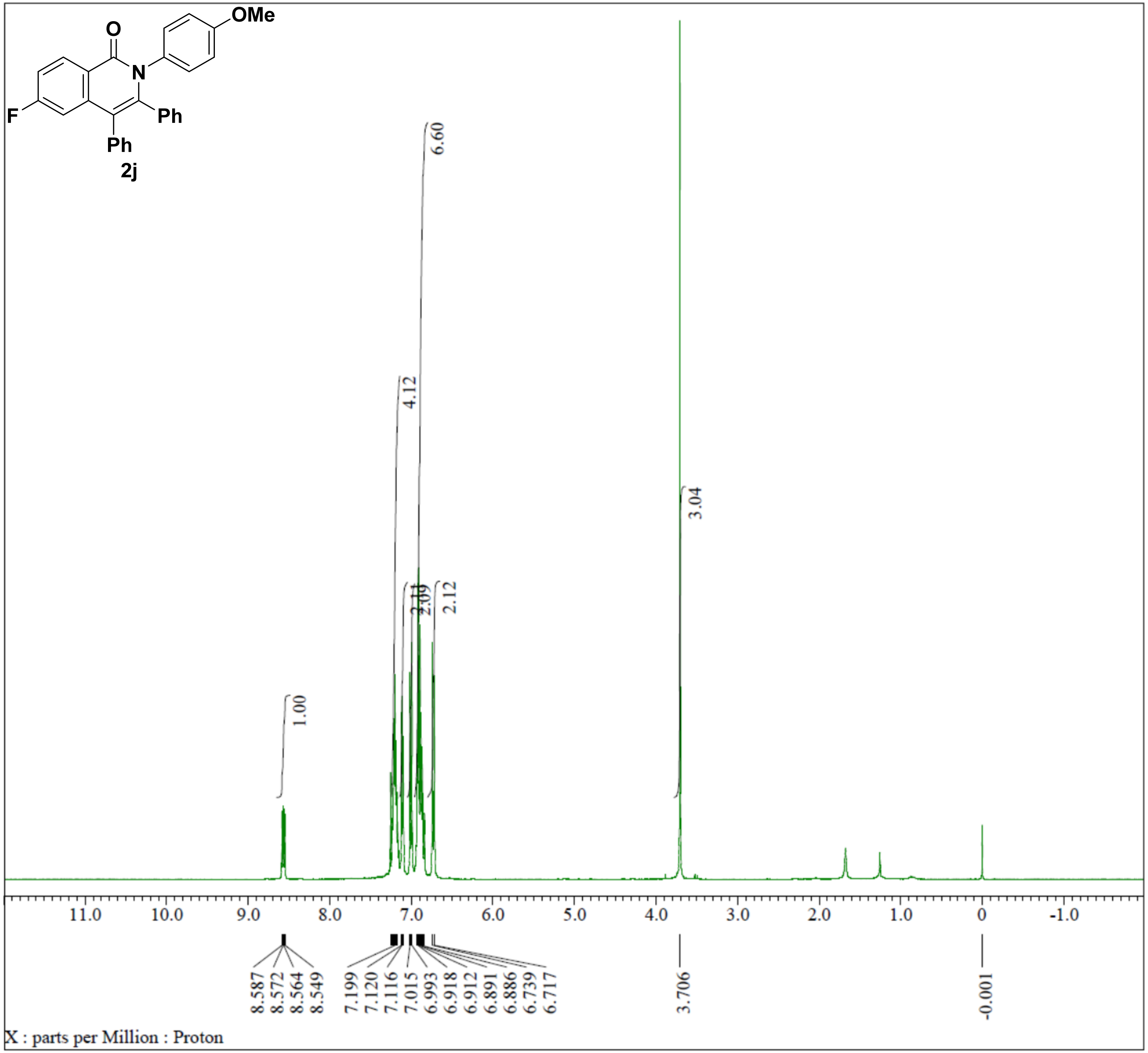


Filename = AO-527 again_Proton-1-2.jdl
Author = delta
Experiment = proton.jxp
Sample_Id = AO-527 again
Solvent = CHLOROFORM-D
Creation_Time = 7-APR-2017 22:20:34
Revision_Time = 21-JUN-2017 22:27:18
Current_Time = 21-JUN-2017 22:27:55

Comment = AO-527 again
Data_Format = 1D COMPLEX
Dim_Size = 13107
Dim_Title = Proton
Dim_Units = [ppm]
Dimensions = X
Site = JNM-ECS400
Spectrometer = DELTA2_NMR

Field_Strength = 9.389766[T] (400[MHz])
X_Acq_Duration = 2.18365952[s]
X_Domain = 1H
X_Freq = 399.78219838[MHz]
X_Offset = 5[ppm]
X_Points = 16384
X_Prescans = 1
X_Resolution = 0.45794685[Hz]
X_Sweep = 7.5030012[kHz]
X_Sweep_Clipped = 6.00240096[kHz]
Irr_Domain = Proton
Irr_Freq = 399.78219838[MHz]
Irr_Offset = 5[ppm]
Tri_Domain = Proton
Tri_Freq = 399.78219838[MHz]
Tri_Offset = 5[ppm]
Clipped = FALSE
Scans = 8
Total_Scans = 8

Relaxation_Delay = 5[s]
Recvr_Gain = 36
Temp_Get = 18.7[dC]
X_90_Width = 11.1[us]
X_Acq_Time = 2.18365952[s]
X_Angle = 45[deg]
X_Atn = 1[dB]
X_Pulse = 5.55[us]
Irr_Mode = Off
Tri_Mode = Off
Dante_Presat = FALSE
Initial_Wait = 1[s]
Repetition_Time = 7.18365952[s]

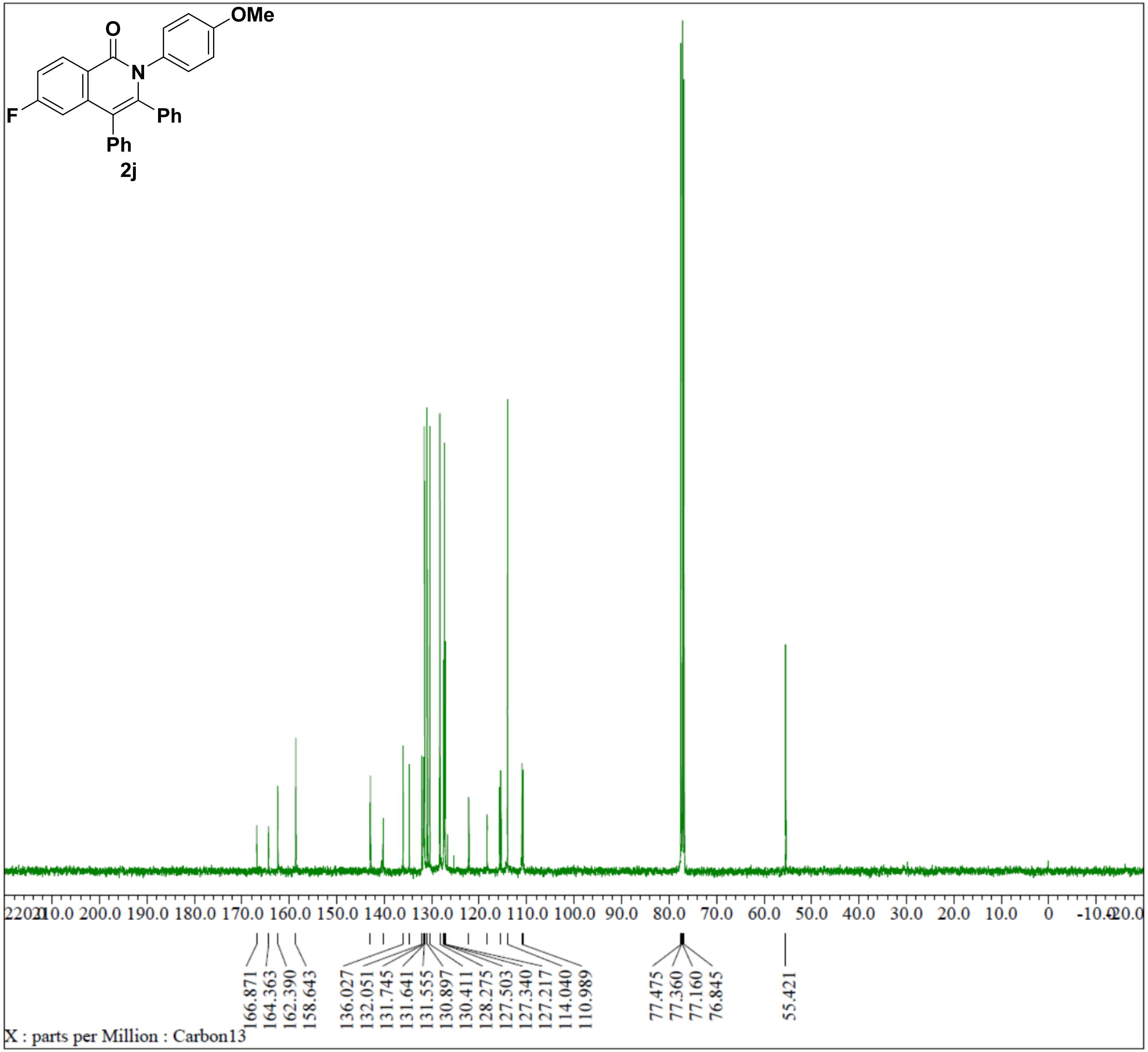


```
Filename          = AO-527 again_Carbon-1-1.jd
Author            = delta
Experiment        = carbon.jxp
Sample_Id         = AO-527 again
Solvent           = CHLOROFORM-D
Creation_Time     =  8-APR-2017 02:06:48
Revision_Time     =  8-APR-2017 11:00:21
Current_Time      = 21-JUN-2017 22:28:20

Comment           = AO-527 again
Data_Format       = 1D COMPLEX
Dim_Size          = 26214
Dim_Title         = Carbon13
Dim_Units         = [ppm]
Dimensions        = X
Site              = JNM-ECS400
Spectrometer      = DELTA2_NMR

Field_Strength    = 9.389766[T] (400[MHz])
X_Acq_Duration    = 1.04333312[s]
X_Domain          = 13C
X_Freq            = 100.52530333[MHz]
X_Offset          = 100[ppm]
X_Points          = 32768
X_Prescans        = 4
X_Resolution      = 0.95846665[Hz]
X_Sweep           = 31.40703518[kHz]
X_Sweep_Clipped   = 25.12562814[kHz]
Irr_Domain        = Proton
Irr_Freq          = 399.78219838[MHz]
Irr_Offset        = 5[ppm]
Clipped           = FALSE
Scans             = 1024
Total_Scans       = 1024

Relaxation_Delay  = 2[s]
Recvr_Gain        = 60
Temp_Get          = 18.9[dC]
X_90_Width        = 9.9[us]
X_Acq_Time        = 1.04333312[s]
X_Angle           = 30[deg]
X_Atn             = 6[dB]
X_Pulse           = 3.3[us]
Irr_Atn_Dec       = 21.307[dB]
Irr_Atn_Noe       = 21.307[dB]
Irr_Noise         = WALTZ
Irr_Pwidth        = 0.115[ms]
Decoupling        = TRUE
Initial_Wait      = 1[s]
Noe               = TRUE
Noe_Time          = 2[s]
Repetition_Time   = 3.04333312[s]
```

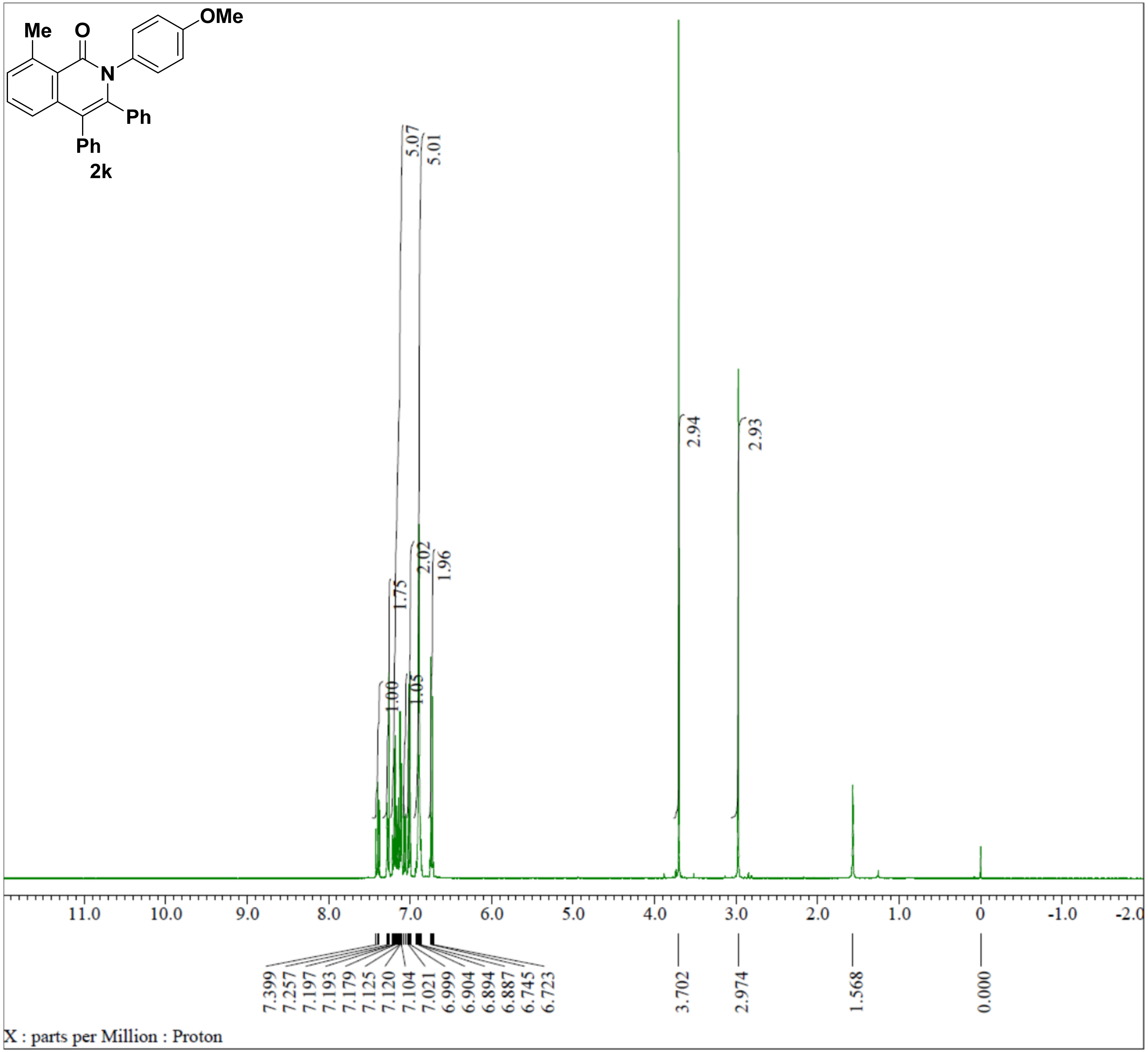


```
Filename         = AO-537 GPC-1-3.jdf
Author           = delta
Experiment       = proton.jxp
Sample_Id        = AO-o-Me GPC
Solvent          = CHLOROFORM-D
Creation_Time    = 21-OCT-2016 22:42:42
Revision_Time    = 21-JUN-2017 22:29:35
Current_Time     = 21-JUN-2017 22:29:47

Comment          = AO-o-Me GPC
Data_Format      = 1D COMPLEX
Dim_Size         = 13107
Dim_Title        = Proton
Dim_Units        = [ppm]
Dimensions       = X
Site             = JNM-ECS400
Spectrometer     = DELTA2_NMR

Field_Strength   = 9.389766[T] (400[MHz])
X_Acq_Duration   = 2.18365952[s]
X_Domain         = 1H
X_Freq           = 399.78219838[MHz]
X_Offset         = 5[ppm]
X_Points         = 16384
X_Prescans       = 1
X_Resolution     = 0.45794685[Hz]
X_Sweep          = 7.5030012[kHz]
X_Sweep_Clipped  = 6.00240096[kHz]
Irr_Domain       = Proton
Irr_Freq         = 399.78219838[MHz]
Irr_Offset       = 5[ppm]
Tri_Domain       = Proton
Tri_Freq         = 399.78219838[MHz]
Tri_Offset       = 5[ppm]
Clipped          = FALSE
Scans            = 8
Total_Scans      = 8

Relaxation_Delay = 5[s]
Recvr_Gain       = 38
Temp_Get         = 22.6[dC]
X_90_Width       = 12.4[us]
X_Acq_Time       = 2.18365952[s]
X_Angle          = 45[deg]
X_Atn            = 1.5[dB]
X_Pulse          = 6.2[us]
Irr_Mode         = Off
Tri_Mode         = Off
Dante_Presat     = FALSE
Initial_Wait     = 1[s]
Repetition_Time  = 7.18365952[s]
```

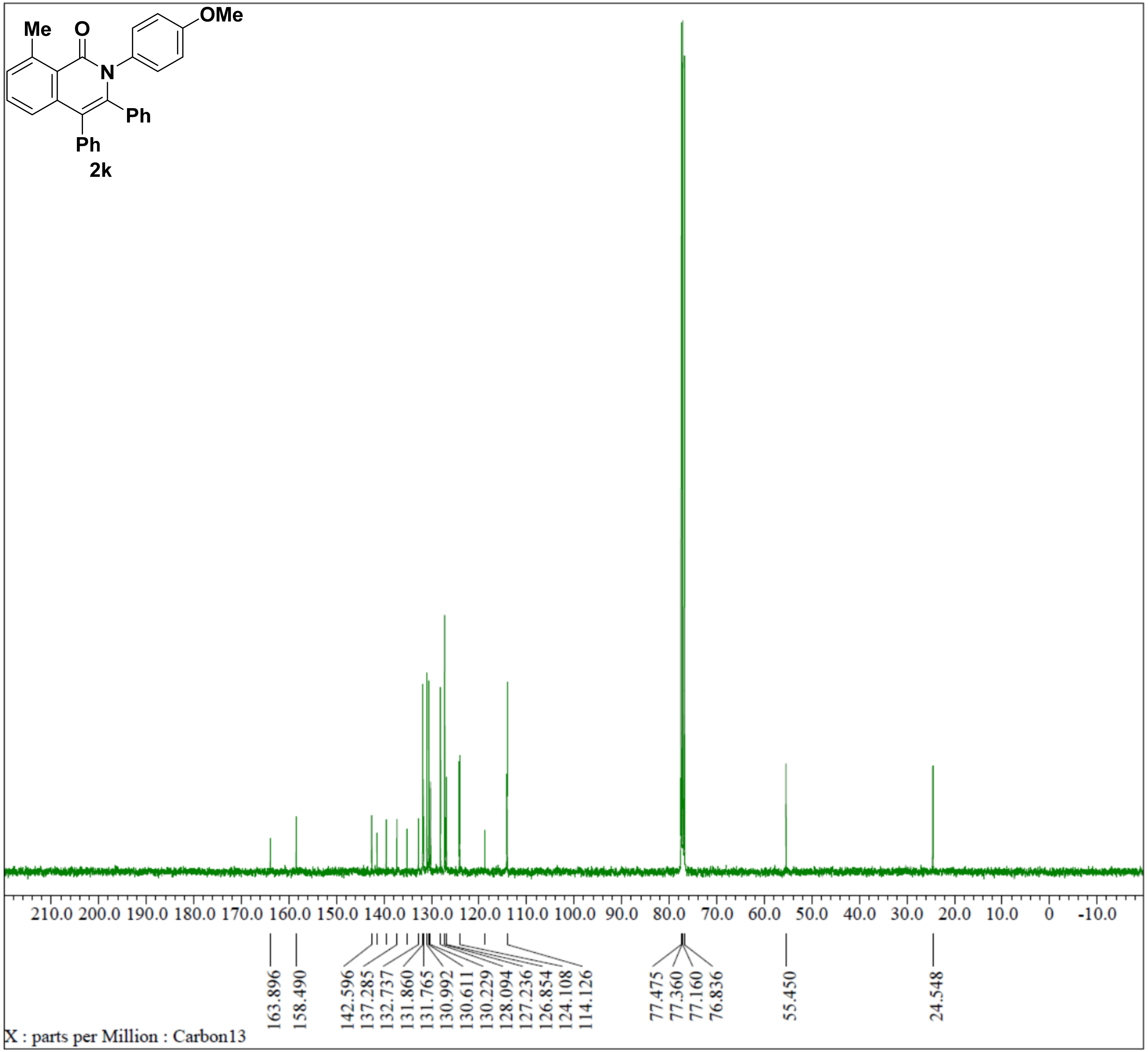


```
Filename           = AO-537 GPC_13C_1-1-2.jdf
Author             = delta
Experiment         = carbon.jxp
Sample_Id          = AO-537 GPC
Solvent            = CHLOROFORM-D
Creation_Time      = 24-OCT-2016 04:04:11
Revision_Time      =  3-APR-2017 13:47:51
Current_Time       = 21-JUN-2017 22:30:08

Comment            = AO-537 GPC_13C_1
Data_Format        = 1D COMPLEX
Dim_Size           = 26214
Dim_Title          = Carbon13
Dim_Units          = [ppm]
Dimensions         = X
Site               = JNM-ECS400
Spectrometer       = DELTA2_NMR

Field_Strength     = 9.389766[T] (400[MHz])
X_Acq_Duration     = 1.04333312[s]
X_Domain           = 13C
X_Freq             = 100.52530333[MHz]
X_Offset           = 100[ppm]
X_Points           = 32768
X_Prescans         = 4
X_Resolution       = 0.95846665[Hz]
X_Sweep            = 31.40703518[kHz]
X_Sweep_Clipped    = 25.12562814[kHz]
Irr_Domain         = Proton
Irr_Freq           = 399.78219838[MHz]
Irr_Offset         = 5[ppm]
Clipped            = FALSE
Scans              = 1024
Total_Scans        = 1024

Relaxation_Delay   = 2[s]
Recvr_Gain         = 60
Temp_Get           = 21.5[dC]
X_90_Width         = 13.7[us]
X_Acq_Time         = 1.04333312[s]
X_Angle            = 30[deg]
X_Atn              = 7[dB]
X_Pulse            = 4.56666667[us]
Irr_Atn_Dec        = 20.846[dB]
Irr_Atn_Noe        = 20.846[dB]
Irr_Noise          = WALTZ
Irr_Pwidth         = 0.115[ms]
Decoupling         = TRUE
Initial_Wait       = 1[s]
Noe                = TRUE
Noe_Time           = 2[s]
Repetition_Time    = 3.04333312[s]
```

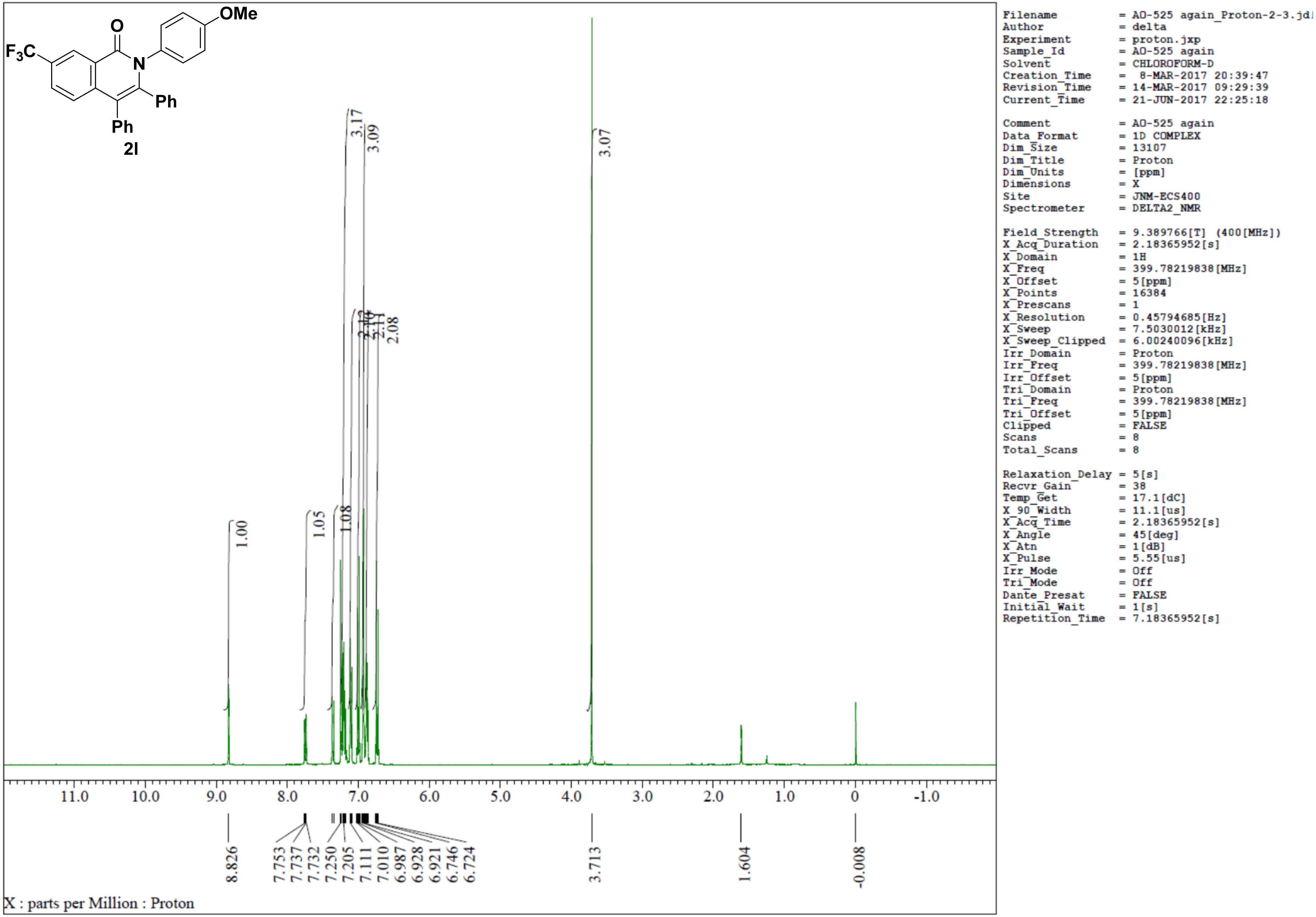
OMe
O
F3C
N
Ph
Ph
2l
3.17
3.09
3.07
2.12
2.10
2.11
2.08
1.00
1.05
1.08
11.0 10.0 9.0 8.0 7.0 6.0 5.0 4.0 3.0 2.0 1.0 0 -1.0
8.826
7.753
7.737
7.732
7.250
7.205
7.111
7.010
6.987
6.928
6.921
6.746
6.724
3.713
1.604
-0.008
X : parts per Million : Proton
Filename = AO-525 again_Proton-2-3.jdf
Author = delta
Experiment = proton.jxp
Sample_Id = AO-525 again
Solvent = CHLOROFORM-D
Creation_Time = 8-MAR-2017 20:39:47
Revision_Time = 14-MAR-2017 09:29:39
Current_Time = 21-JUN-2017 22:25:18
Comment = AO-525 again
Data_Format = 1D COMPLEX
Dim_Size = 13107
Dim_Title = Proton
Dim_Units = [ppm]
Dimensions = X
Site = JNM-ECS400
Spectrometer = DELTA2_NMR
Field_Strength = 9.389766[T] (400[MHz])
X_Acq_Duration = 2.18365952[s]
X_Domain = 1H
X_Freq = 399.78219838[MHz]
X_Offset = 5[ppm]
X_Points = 16384
X_Prescans = 1
X_Resolution = 0.45794685[Hz]
X_Sweep = 7.5030012[kHz]
X_Sweep_Clipped = 6.00240096[kHz]
Irr_Domain = Proton
Irr_Freq = 399.78219838[MHz]
Irr_Offset = 5[ppm]
Tri_Domain = Proton
Tri_Freq = 399.78219838[MHz]
Tri_Offset = 5[ppm]
Clipped = FALSE
Scans = 8
Total_Scans = 8
Relaxation_Delay = 5[s]
Recvr_Gain = 38
Temp_Get = 17.1[dC]
X_90_Width = 11.1[us]
X_Acq_Time = 2.18365952[s]
X_Angle = 45[deg]
X_Atn = 1[dB]
X_Pulse = 5.55[us]
Irr_Mode = Off
Tri_Mode = Off
Dante_Presat = FALSE
Initial_Wait = 1[s]
Repetition_Time = 7.18365952[s]

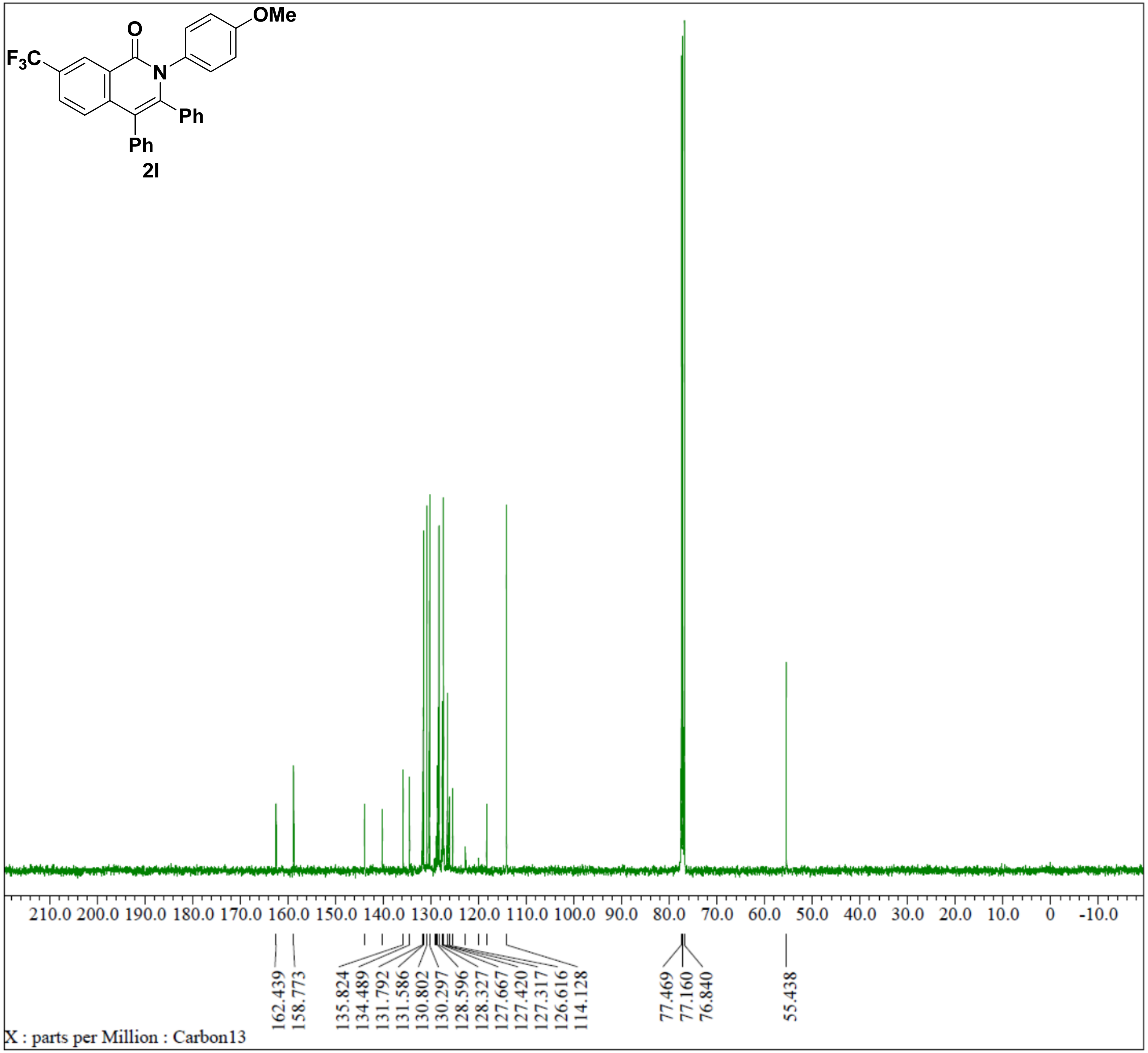


```
Filename           = AO-525_Carbon-1-1.jdf
Author             = delta
Experiment         = carbon.jxp
Sample_Id          = AO-525
Solvent            = CHLOROFORM-D
Creation_Time      =  8-MAR-2017 04:55:47
Revision_Time      = 14-MAR-2017 09:29:21
Current_Time       = 22-JUN-2017 00:31:58

Comment            = AO-525
Data_Format        = 1D COMPLEX
Dim_Size           = 26214
Dim_Title          = Carbon13
Dim_Units          = [ppm]
Dimensions         = X
Site               = JNM-ECS400
Spectrometer       = DELTA2_NMR

Field_Strength     = 9.389766[T] (400[MHz])
X_Acq_Duration     = 0.96468992[s]
X_Domain           = 13C
X_Freq             = 100.52530333[MHz]
X_Offset           = 100[ppm]
X_Points           = 32768
X_Prescans         = 4
X_Resolution       = 1.03660252[Hz]
X_Sweep            = 33.9673913[kHz]
X_Sweep_Clipped    = 27.17391304[kHz]
Irr_Domain         = Proton
Irr_Freq           = 399.78219838[MHz]
Irr_Offset         = 5[ppm]
Clipped            = FALSE
Scans              = 1024
Total_Scans        = 1024

Relaxation_Delay   = 2[s]
Recvr_Gain         = 60
Temp_Get           = 16.6[dC]
X_90_Width         = 9.9[us]
X_Acq_Time         = 0.96468992[s]
X_Angle            = 30[deg]
X_Atn              = 6[dB]
X_Pulse            = 3.3[us]
Irr_Atn_Dec        = 21.307[dB]
Irr_Atn_Noe        = 21.307[dB]
Irr_Noise          = WALTZ
Irr_Pwidth         = 0.115[ms]
Decoupling         = TRUE
Initial_Wait       = 1[s]
Noe                = TRUE
Noe_Time           = 2[s]
Repetition_Time    = 2.96468992[s]
```

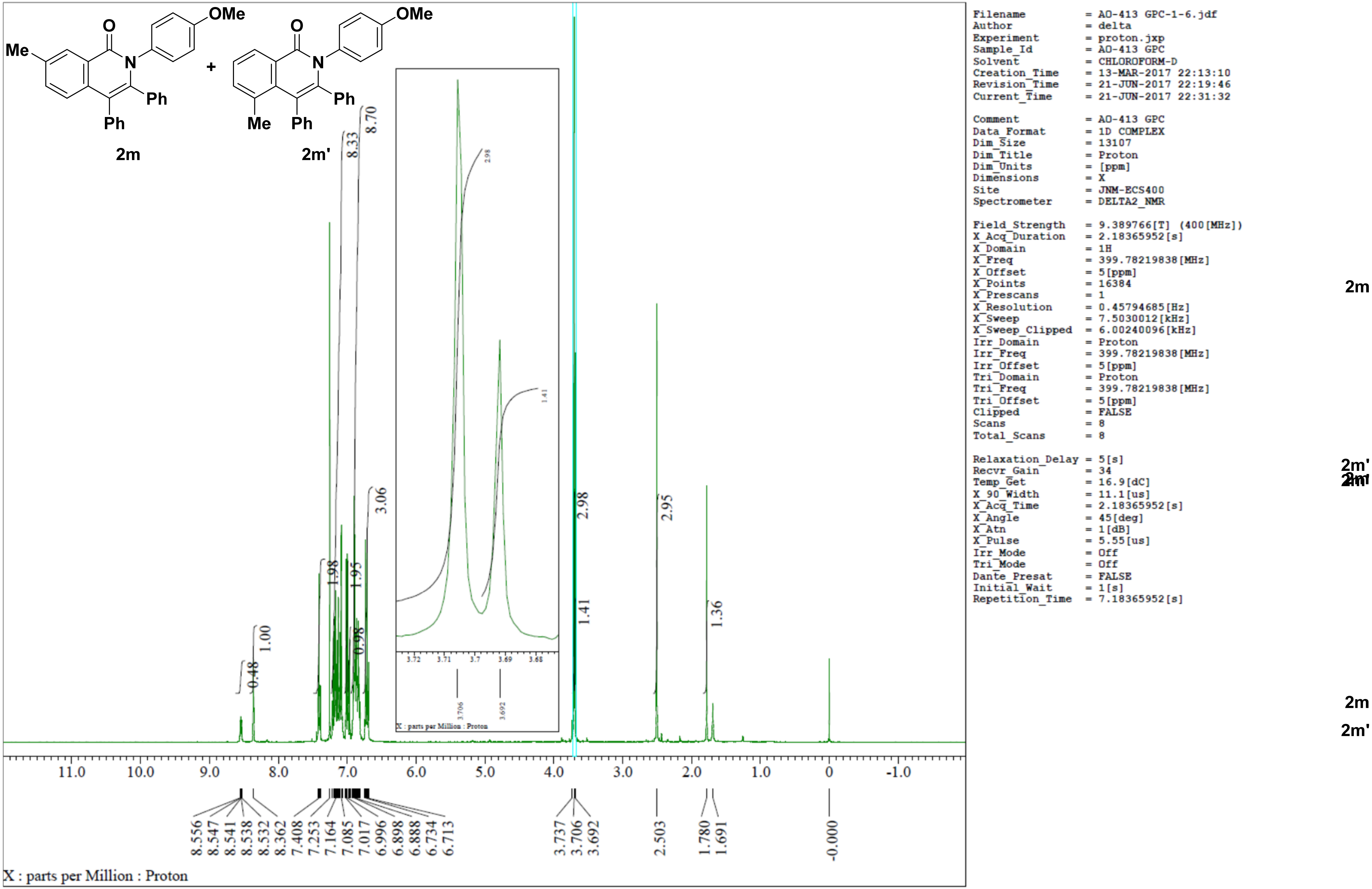
OMe
O
Me
N
Ph
Ph
2m
+
OMe
O
N
Me
Ph
Ph
2m'
8.70
8.33
3.06
1.98
1.95
0.98
1.00
0.48
2.98
1.41
2.95
1.36
8.556
8.547
8.541
8.538
8.532
8.362
7.408
7.253
7.164
7.085
7.017
6.996
6.898
6.888
6.734
6.713
3.737
3.706
3.692
2.503
1.780
1.691
-0.000
X : parts per Million : Proton
Filename = AO-413 GPC-1-6.jdf
Author = delta
Experiment = proton.jxp
Sample_Id = AO-413 GPC
Solvent = CHLOROFORM-D
Creation_Time = 13-MAR-2017 22:13:10
Revision_Time = 21-JUN-2017 22:19:46
Current_Time = 21-JUN-2017 22:31:32
Comment = AO-413 GPC
Data_Format = 1D COMPLEX
Dim_Size = 13107
Dim_Title = Proton
Dim_Units = [ppm]
Dimensions = X
Site = JNM-ECS400
Spectrometer = DELTA2_NMR
Field_Strength = 9.389766[T] (400[MHz])
X_Acq_Duration = 2.18365952[s]
X_Domain = 1H
X_Freq = 399.78219838[MHz]
X_Offset = 5[ppm]
X_Points = 16384
X_Prescans = 1
X_Resolution = 0.45794685[Hz]
X_Sweep = 7.5030012[kHz]
X_Sweep_Clipped = 6.00240096[kHz]
Irr_Domain = Proton
Irr_Freq = 399.78219838[MHz]
Irr_Offset = 5[ppm]
Tri_Domain = Proton
Tri_Freq = 399.78219838[MHz]
Tri_Offset = 5[ppm]
Clipped = FALSE
Scans = 8
Total_Scans = 8
Relaxation_Delay = 5[s]
Recvr_Gain = 34
Temp_Get = 16.9[dC]
X_90_Width = 11.1[us]
X_Acq_Time = 2.18365952[s]
X_Angle = 45[deg]
X_Atn = 1[dB]
X_Pulse = 5.55[us]
Irr_Mode = Off
Tri_Mode = Off
Dante_Presat = FALSE
Initial_Wait = 1[s]
Repetition_Time = 7.18365952[s]
2m
2m'
2m
2m'

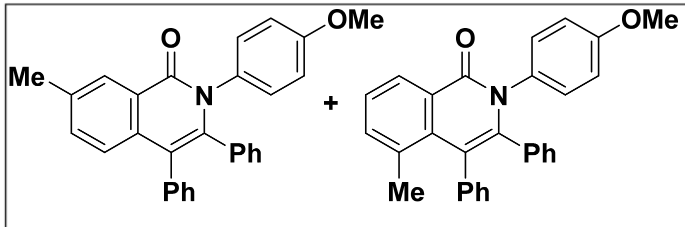


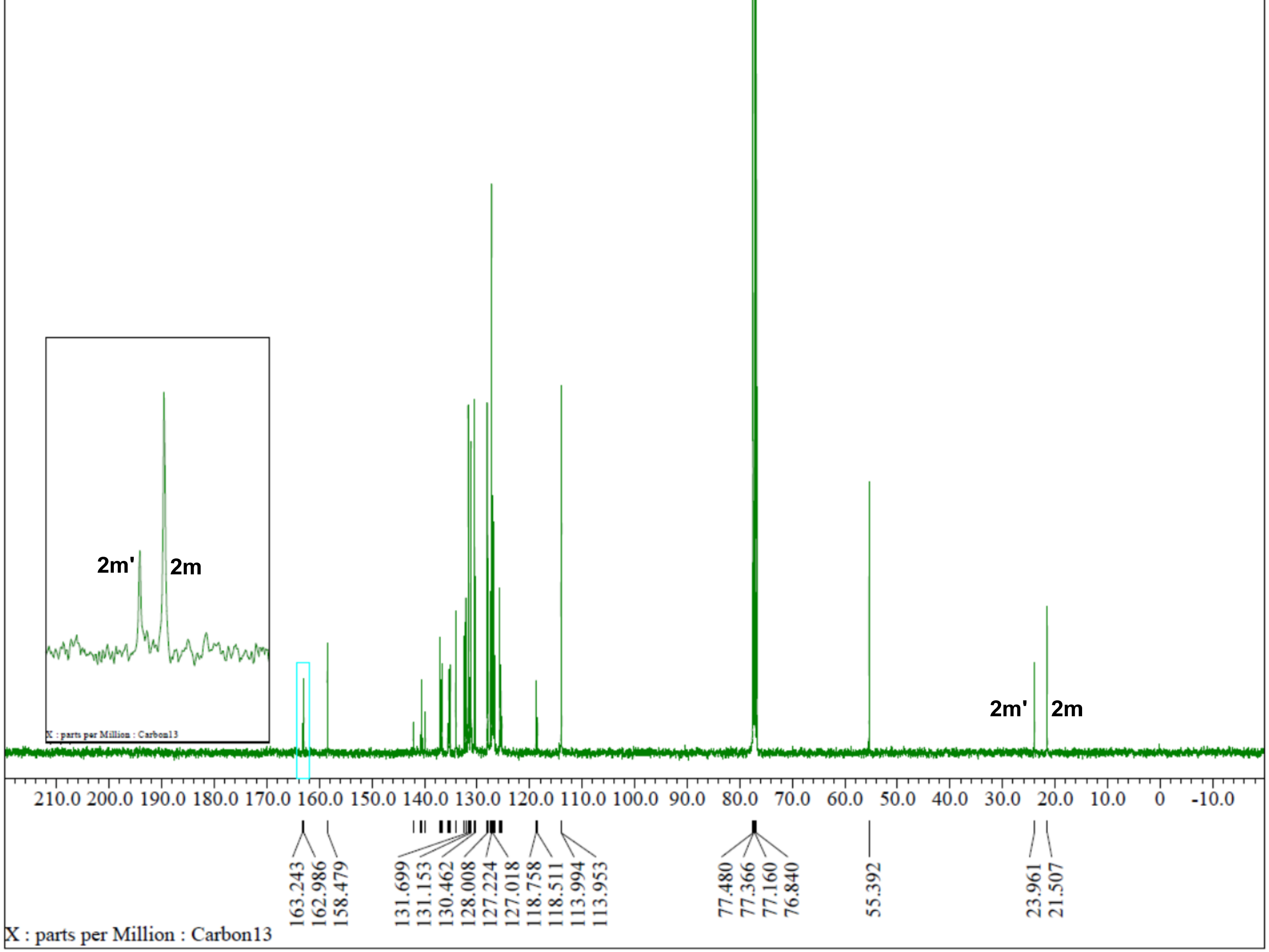


```
Filename            = AO-413 GPC carbon.jdf-2.jd
Author              = delta
Experiment          = carbon.jxp
Sample_Id           = AO-413 GPC
Solvent             = CHLOROFORM-D
Creation_Time       = 14-MAR-2017 06:06:23
Revision_Time       = 14-MAR-2017 09:31:34
Current_Time        = 21-JUN-2017 22:20:46

Comment             = AO-413 GPC
Data_Format         = 1D COMPLEX
Dim_Size            = 26214
Dim_Title           = Carbon13
Dim_Units           = [ppm]
Dimensions          = X
Site                = JNM-ECS400
Spectrometer        = DELTA2_NMR

Field_Strength      = 9.389766[T] (400[MHz])
X_Acq_Duration      = 0.96468992[s]
X_Domain            = 13C
X_Freq              = 100.52530333[MHz]
X_Offset            = 100[ppm]
X_Points            = 32768
X_Prescans          = 4
X_Resolution        = 1.03660252[Hz]
X_Sweep             = 33.9673913[kHz]
X_Sweep_Clipped     = 27.17391304[kHz]
Irr_Domain          = Proton
Irr_Freq            = 399.78219838[MHz]
Irr_Offset          = 5[ppm]
Clipped             = FALSE
Scans               = 1024
Total_Scans         = 1024

Relaxation_Delay    = 2[s]
Recvr_Gain          = 60
Temp_Get            = 17.5[dC]
X_90_Width          = 9.9[us]
X_Acq_Time          = 0.96468992[s]
X_Angle             = 30[deg]
X_Atn               = 6[dB]
X_Pulse             = 3.3[us]
Irr_Atn_Dec         = 21.307[dB]
Irr_Atn_Noe         = 21.307[dB]
Irr_Noise           = WALTZ
Irr_Pwidth          = 0.115[ms]
Decoupling          = TRUE
Initial_Wait        = 1[s]
Noe                 = TRUE
Noe_Time            = 2[s]
Repetition_Time     = 2.96468992[s]
```

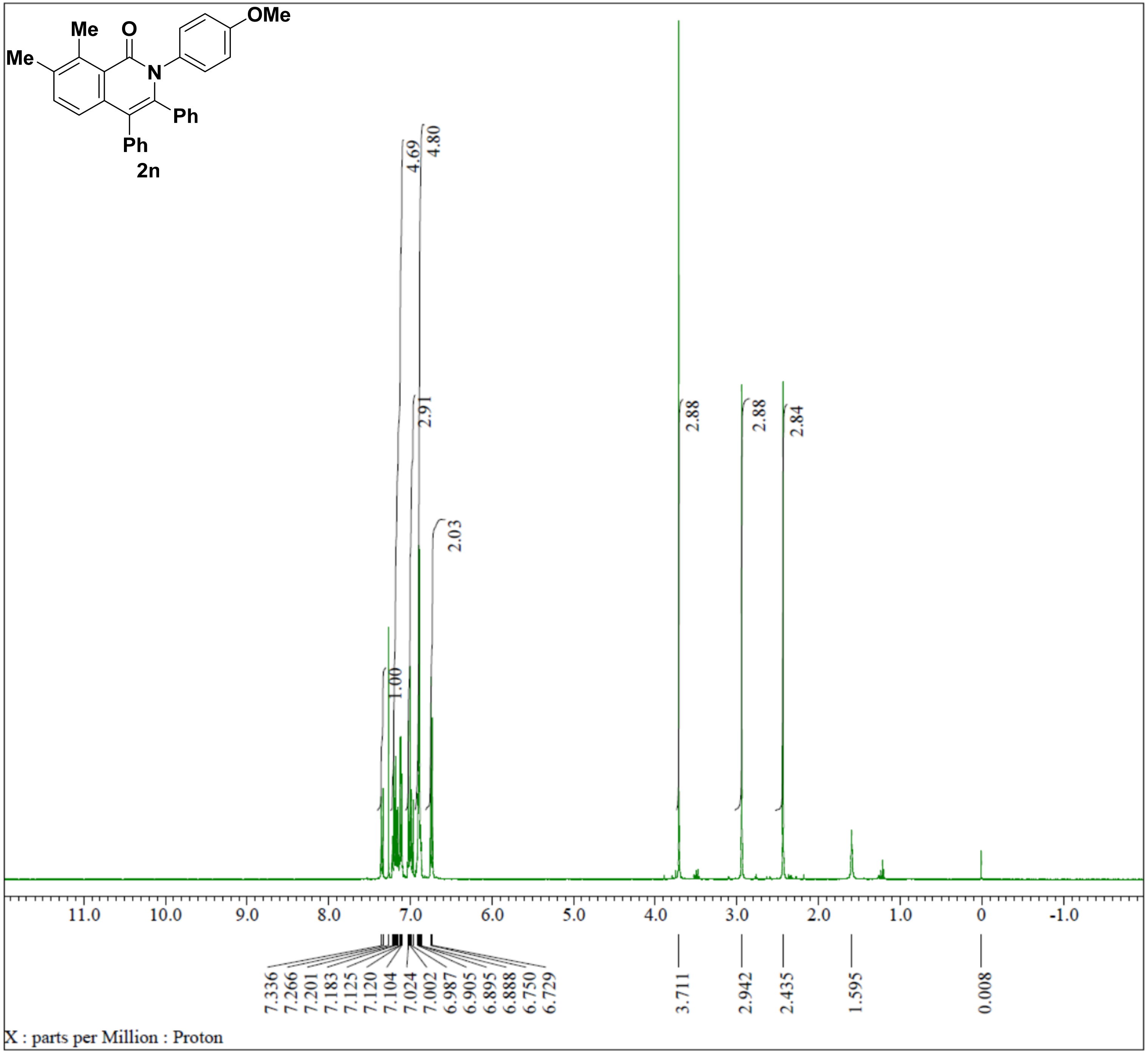


```
Filename          = AO-578 GPC 1_Proton-1-2.jd
Author            = delta
Experiment        = proton.jxp
Sample_Id         = AO-578 GPC 1
Solvent           = CHLOROFORM-D
Creation_Time     =  5-DEC-2016 11:14:09
Revision_Time     = 22-JUN-2017 00:34:05
Current_Time      = 22-JUN-2017 00:34:19

Comment           = AO-578 GPC 1
Data_Format       = 1D COMPLEX
Dim_Size          = 13107
Dim_Title         = Proton
Dim_Units         = [ppm]
Dimensions        = X
Site              = JNM-ECS400
Spectrometer      = DELTA2_NMR

Field_Strength    = 9.389766[T] (400[MHz])
X_Acq_Duration    = 2.18365952[s]
X_Domain          = 1H
X_Freq            = 399.78219838[MHz]
X_Offset          = 5[ppm]
X_Points          = 16384
X_Prescans        = 1
X_Resolution      = 0.45794685[Hz]
X_Sweep           = 7.5030012[kHz]
X_Sweep_Clipped   = 6.00240096[kHz]
Irr_Domain        = Proton
Irr_Freq          = 399.78219838[MHz]
Irr_Offset        = 5[ppm]
Tri_Domain        = Proton
Tri_Freq          = 399.78219838[MHz]
Tri_Offset        = 5[ppm]
Clipped           = FALSE
Scans             = 8
Total_Scans       = 8

Relaxation_Delay  = 5[s]
Recvr_Gain        = 38
Temp_Get          = 21[dC]
X_90_Width        = 12.4[us]
X_Acq_Time        = 2.18365952[s]
X_Angle           = 45[deg]
X_Atn             = 1.5[dB]
X_Pulse           = 6.2[us]
Irr_Mode          = Off
Tri_Mode          = Off
Dante_Presat      = FALSE
Initial_Wait      = 1[s]
Repetition_Time   = 7.18365952[s]
```

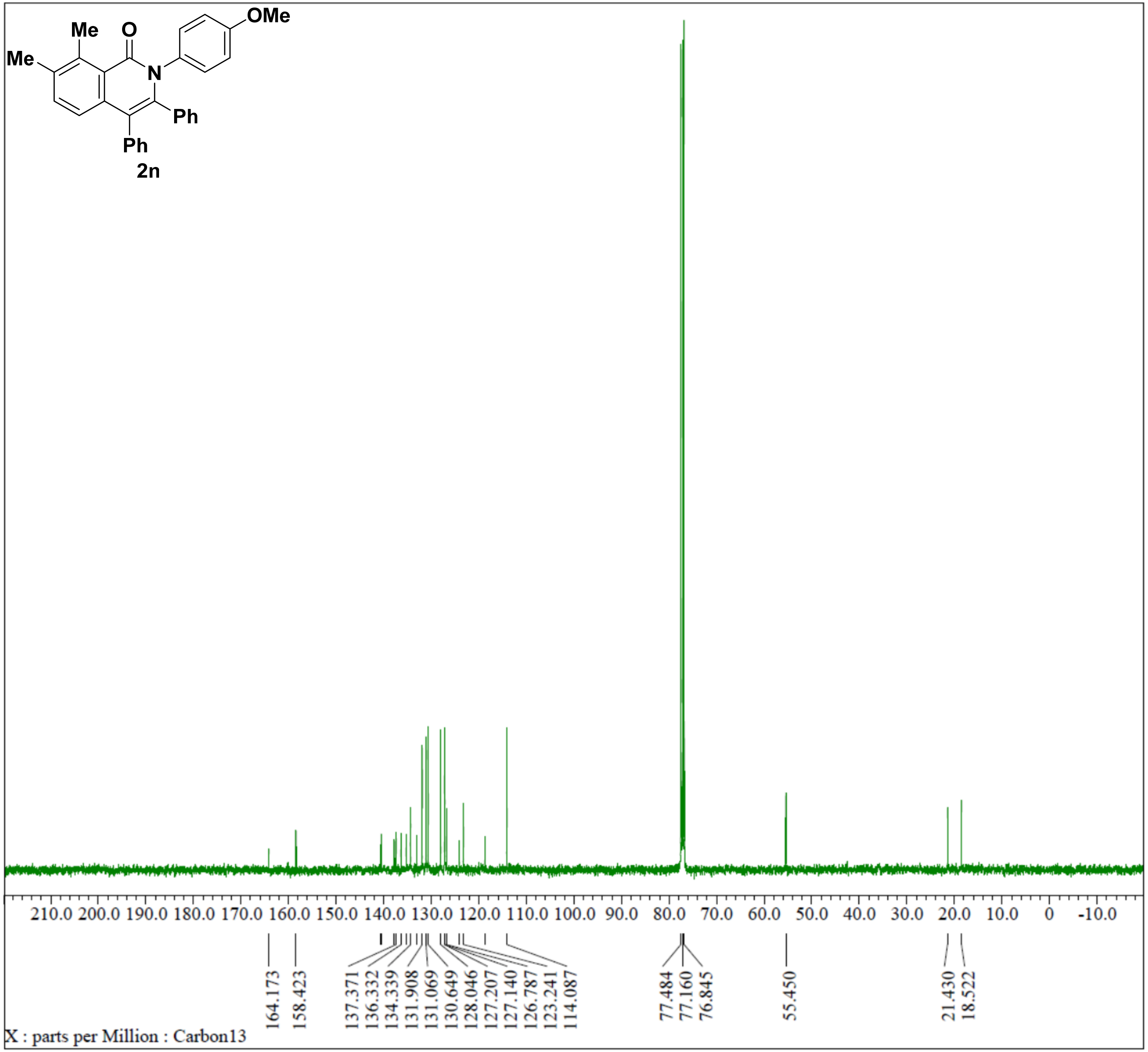


Filename = AO-578 GPC_Carbon-1-2.jdf
Author = delta
Experiment = carbon.jxp
Sample_Id = AO-578 GPC
Solvent = CHLOROFORM-D
Creation_Time = 3-DEC-2016 02:38:28
Revision_Time = 22-JUN-2017 00:34:39
Current_Time = 22-JUN-2017 00:34:54

Comment = AO-578 GPC_13C
Data_Format = 1D COMPLEX
Dim_Size = 26214
Dim_Title = Carbon13
Dim_Units = [ppm]
Dimensions = X
Site = JNM-ECS400
Spectrometer = DELTA2_NMR

Field_Strength = 9.389766[T] (400[MHz])
X_Acq_Duration = 1.04333312[s]
X_Domain = 13C
X_Freq = 100.52530333[MHz]
X_Offset = 100[ppm]
X_Points = 32768
X_Prescans = 4
X_Resolution = 0.95846665[Hz]
X_Sweep = 31.40703518[kHz]
X_Sweep_Clipped = 25.12562814[kHz]
Irr_Domain = Proton
Irr_Freq = 399.78219838[MHz]
Irr_Offset = 5[ppm]
Clipped = FALSE
Scans = 1024
Total_Scans = 1024

Relaxation_Delay = 2[s]
Recvr_Gain = 60
Temp_Get = 20.8[dC]
X_90_Width = 13.7[us]
X_Acq_Time = 1.04333312[s]
X_Angle = 30[deg]
X_Atn = 7[dB]
X_Pulse = 4.56666667[us]
Irr_Atn_Dec = 20.846[dB]
Irr_Atn_Noe = 20.846[dB]
Irr_Noise = WALTZ
Irr_Pwidth = 0.115[ms]
Decoupling = TRUE
Initial_Wait = 1[s]
Noe = TRUE
Noe_Time = 2[s]
Repetition_Time = 3.04333312[s]

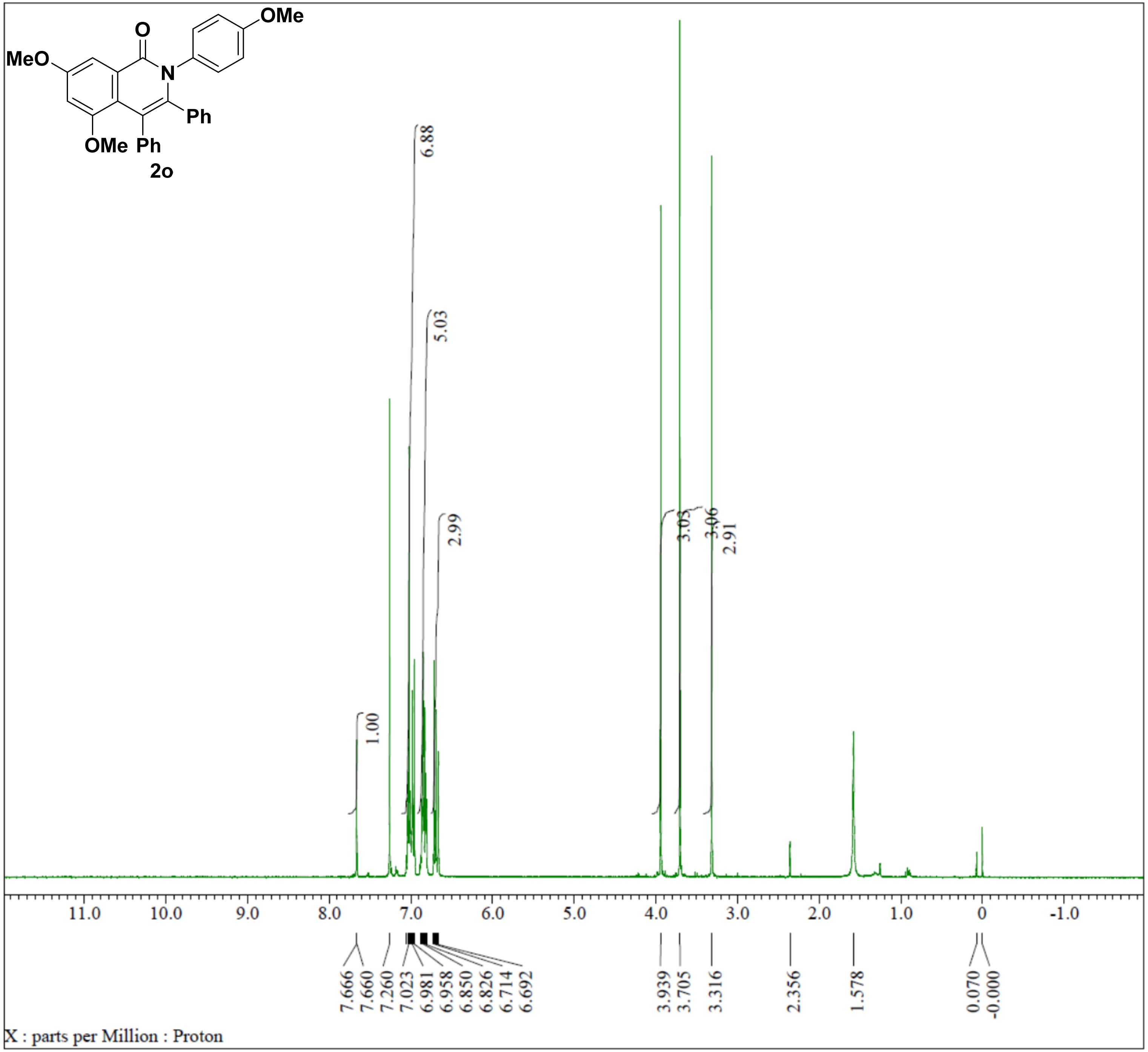
OMe
O
MeO
N
Ph
OMe Ph
2o
Filename = AO-632 column 25~35_Proton
Author = delta
Experiment = proton.jxp
Sample_Id = AO-632 column 25~35
Solvent = CHLOROFORM-D
Creation_Time = 21-NOV-2016 17:13:43
Revision_Time = 22-JUN-2017 00:35:42
Current_Time = 22-JUN-2017 00:35:53
Comment = AO-632 column 25~35
Data_Format = 1D COMPLEX
Dim_Size = 13107
Dim_Title = Proton
Dim_Units = [ppm]
Dimensions = X
Site = JNM-ECS400
Spectrometer = DELTA2_NMR
Field_Strength = 9.389766[T] (400[MHz])
X_Acq_Duration = 2.18365952[s]
X_Domain = 1H
X_Freq = 399.78219838[MHz]
X_Offset = 5[ppm]
X_Points = 16384
X_Prescans = 1
X_Resolution = 0.45794685[Hz]
X_Sweep = 7.5030012[kHz]
X_Sweep_Clipped = 6.00240096[kHz]
Irr_Domain = Proton
Irr_Freq = 399.78219838[MHz]
Irr_Offset = 5[ppm]
Tri_Domain = Proton
Tri_Freq = 399.78219838[MHz]
Tri_Offset = 5[ppm]
Clipped = FALSE
Scans = 8
Total_Scans = 8
Relaxation_Delay = 5[s]
Recvr_Gain = 40
Temp_Get = 21.3[dC]
X_90_Width = 12.4[us]
X_Acq_Time = 2.18365952[s]
X_Angle = 45[deg]
X_Atn = 1.5[dB]
X_Pulse = 6.2[us]
Irr_Mode = Off
Tri_Mode = Off
Dante_Presat = FALSE
Initial_Wait = 1[s]
Repetition_Time = 7.18365952[s]
1.00
6.88
5.03
2.99
3.03
3.06
2.91
11.0 10.0 9.0 8.0 7.0 6.0 5.0 4.0 3.0 2.0 1.0 0 -1.0
7.666
7.660
7.260
7.023
6.981
6.958
6.850
6.826
6.714
6.692
3.939
3.705
3.316
2.356
1.578
0.070
-0.000
X : parts per Million : Proton

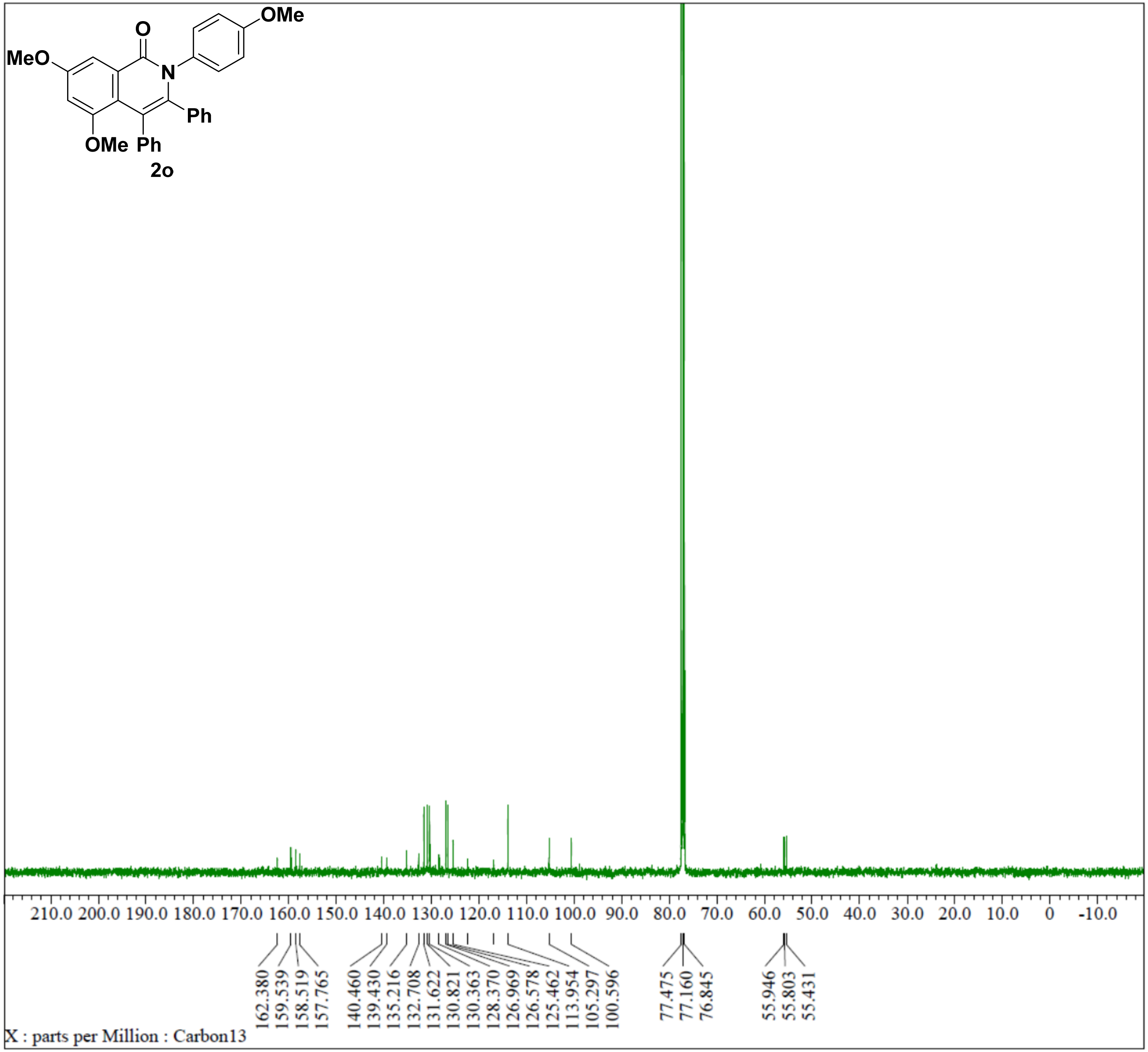


```
Filename            = AO-632 column 25~35_Carbon
Author              = delta
Experiment          = carbon.jxp
Sample_Id           = AO-632 column 25~35
Solvent             = CHLOROFORM-D
Creation_Time       = 23-NOV-2016 02:03:19
Revision_Time       =  3-APR-2017 13:39:39
Current_Time        = 21-JUN-2017 22:38:08

Comment             = AO-632 column 25~35_13C
Data_Format         = 1D COMPLEX
Dim_Size            = 26214
Dim_Title           = Carbon13
Dim_Units           = [ppm]
Dimensions          = X
Site                = JNM-ECS400
Spectrometer        = DELTA2_NMR

Field_Strength      = 9.389766[T] (400[MHz])
X_Acq_Duration      = 1.04333312[s]
X_Domain            = 13C
X_Freq              = 100.52530333[MHz]
X_Offset            = 100[ppm]
X_Points            = 32768
X_Prescans          = 4
X_Resolution        = 0.95846665[Hz]
X_Sweep             = 31.40703518[kHz]
X_Sweep_Clipped     = 25.12562814[kHz]
Irr_Domain          = Proton
Irr_Freq            = 399.78219838[MHz]
Irr_Offset          = 5[ppm]
Clipped             = FALSE
Scans               = 1024
Total_Scans         = 1024

Relaxation_Delay    = 2[s]
Recvr_Gain          = 60
Temp_Get            = 22.3[dC]
X_90_Width          = 13.7[us]
X_Acq_Time          = 1.04333312[s]
X_Angle             = 30[deg]
X_Atn               = 7[dB]
X_Pulse             = 4.56666667[us]
Irr_Atn_Dec         = 20.846[dB]
Irr_Atn_Noe         = 20.846[dB]
Irr_Noise           = WALTZ
Irr_Pwidth          = 0.115[ms]
Decoupling          = TRUE
Initial_Wait        = 1[s]
Noe                 = TRUE
Noe_Time            = 2[s]
Repetition_Time     = 3.04333312[s]
```

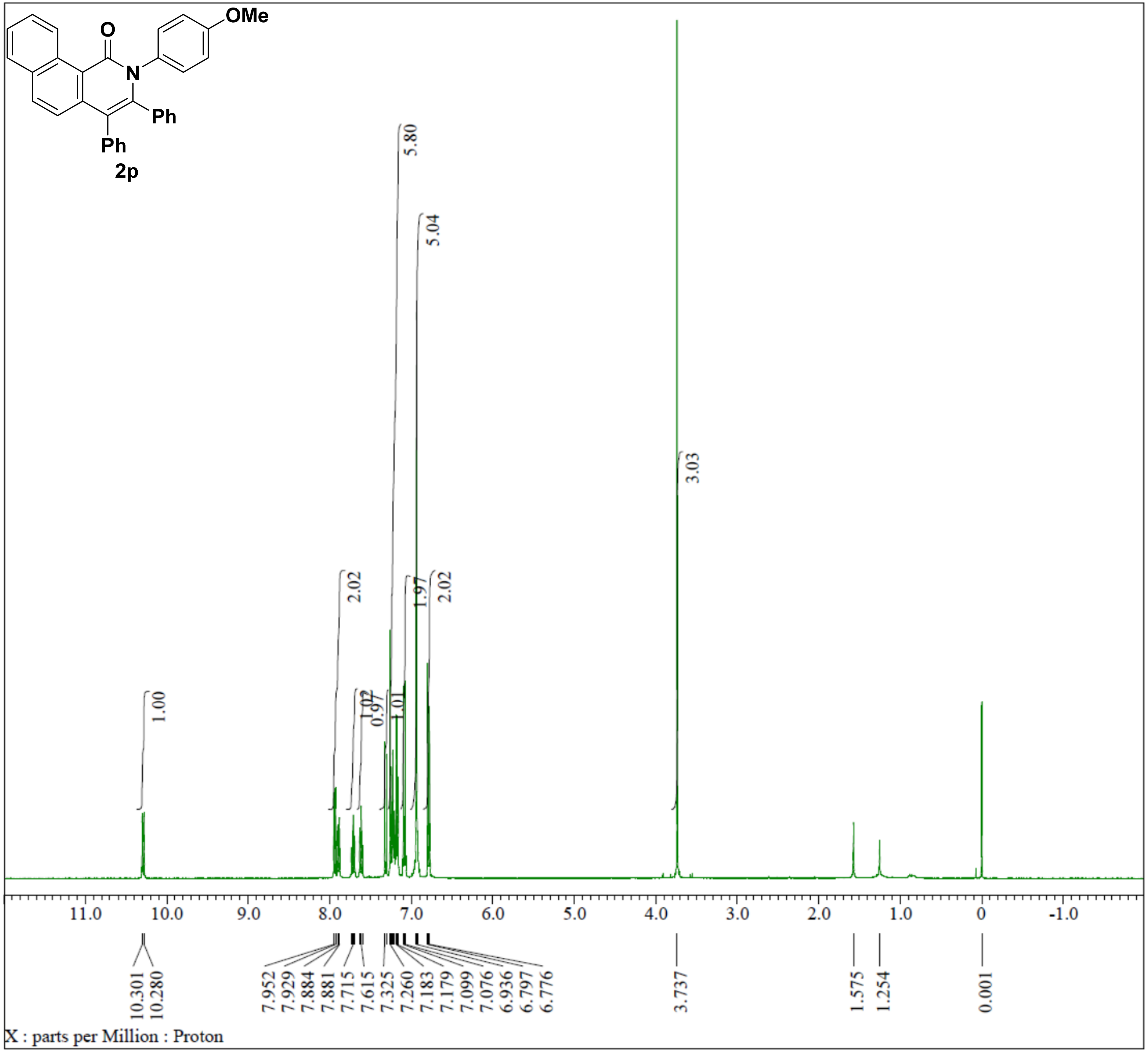


| | |
|---|---|
| Filename | = AO-644_Proton-1-2.jdf |
| Author | = delta |
| Experiment | = proton.jxp |
| Sample_Id | = AO-644 |
| Solvent | = CHLOROFORM-D |
| Creation_Time | = 25-MAR-2017 17:50:56 |
| Revision_Time | = 3-APR-2017 13:34:45 |
| Current_Time | = 21-JUN-2017 22:41:57 |
| Comment | = AO-644 |
| Data_Format | = 1D COMPLEX |
| Dim_Size | = 13107 |
| Dim_Title | = Proton |
| Dim_Units | = [ppm] |
| Dimensions | = X |
| Site | = JNM-ECS400 |
| Spectrometer | = DELTA2_NMR |
| Field_Strength | = 9.389766[T] (400[MHz]) |
| X_Acq_Duration | = 2.18365952[s] |
| X_Domain | = 1H |
| X_Freq | = 399.78219838[MHz] |
| X_Offset | = 5[ppm] |
| X_Points | = 16384 |
| X_Prescans | = 1 |
| X_Resolution | = 0.45794685[Hz] |
| X_Sweep | = 7.5030012[kHz] |
| X_Sweep_Clipped | = 6.00240096[kHz] |
| Irr_Domain | = Proton |
| Irr_Freq | = 399.78219838[MHz] |
| Irr_Offset | = 5[ppm] |
| Tri_Domain | = Proton |
| Tri_Freq | = 399.78219838[MHz] |
| Tri_Offset | = 5[ppm] |
| Clipped | = FALSE |
| Scans | = 8 |
| Total_Scans | = 8 |
| Relaxation_Delay | = 5[s] |
| Recvr_Gain | = 44 |
| Temp_Get | = 15.9[dC] |
| X_90_Width | = 11.1[us] |
| X_Acq_Time | = 2.18365952[s] |
| X_Angle | = 45[deg] |
| X_Atn | = 1[dB] |
| X_Pulse | = 5.55[us] |
| Irr_Mode | = Off |
| Tri_Mode | = Off |
| Dante_Presat | = FALSE |
| Initial_Wait | = 1[s] |
| Repetition_Time | = 7.18365952[s] |

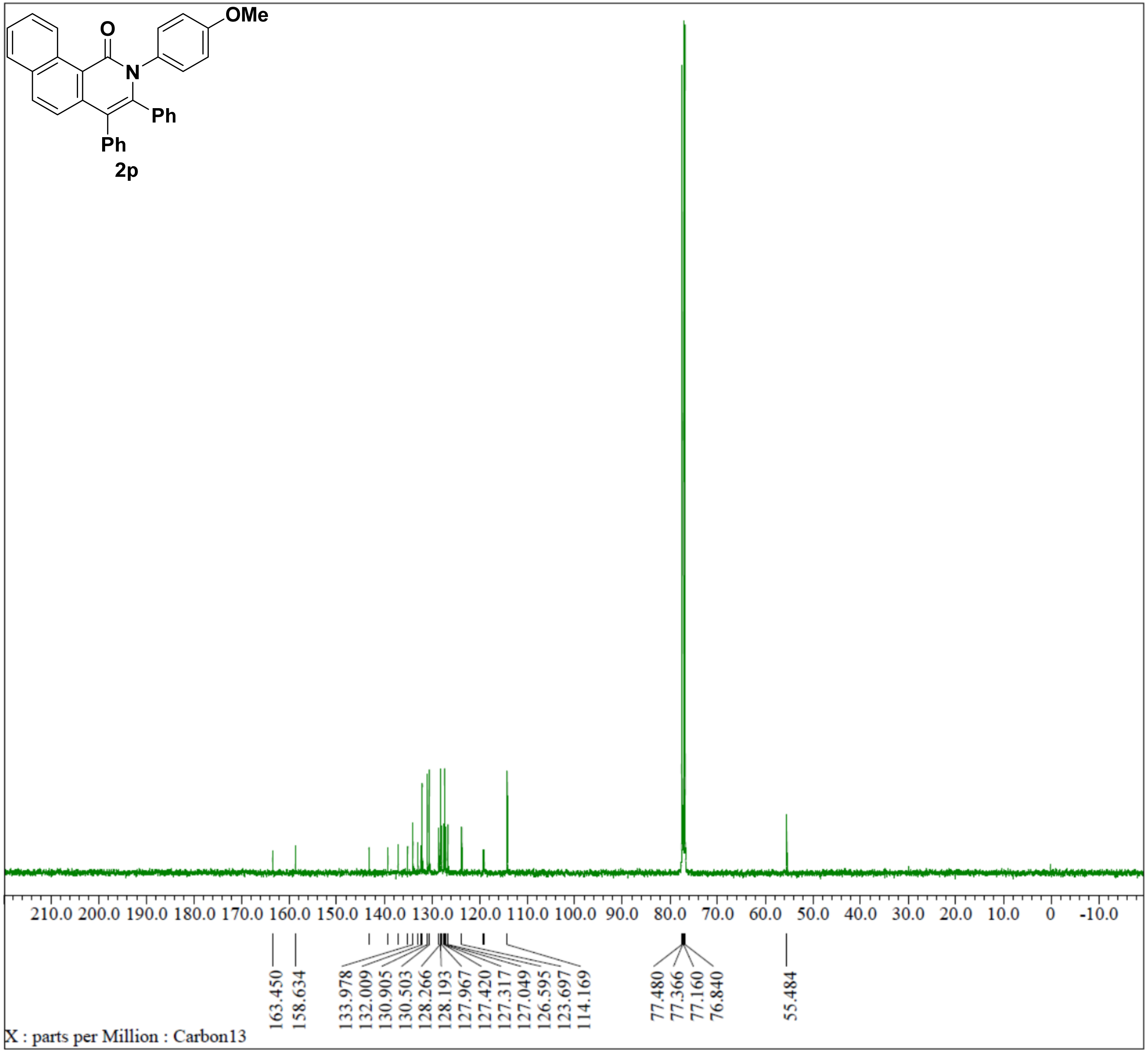


```
Filename           = AO-644_Carbon-1-1.jdf
Author             = delta
Experiment         = carbon.jxp
Sample_Id          = AO-644
Solvent            = CHLOROFORM-D
Creation_Time      = 26-MAR-2017 02:05:21
Revision_Time      = 27-MAR-2017 14:54:19
Current_Time       = 21-JUN-2017 22:41:33

Comment            = AO-644
Data_Format        = 1D COMPLEX
Dim_Size           = 26214
Dim_Title          = Carbon13
Dim_Units          = [ppm]
Dimensions         = X
Site               = JNM-ECS400
Spectrometer       = DELTA2_NMR

Field_Strength     = 9.389766[T] (400[MHz])
X_Acq_Duration     = 0.96468992[s]
X_Domain           = 13C
X_Freq             = 100.52530333[MHz]
X_Offset           = 100[ppm]
X_Points           = 32768
X_Prescans         = 4
X_Resolution       = 1.03660252[Hz]
X_Sweep            = 33.9673913[kHz]
X_Sweep_Clipped    = 27.17391304[kHz]
Irr_Domain         = Proton
Irr_Freq           = 399.78219838[MHz]
Irr_Offset         = 5[ppm]
Clipped            = FALSE
Scans              = 1024
Total_Scans        = 1024

Relaxation_Delay   = 2[s]
Recvr_Gain         = 60
Temp_Get           = 16.6[dC]
X_90_Width         = 9.9[us]
X_Acq_Time         = 0.96468992[s]
X_Angle            = 30[deg]
X_Atn              = 6[dB]
X_Pulse            = 3.3[us]
Irr_Atn_Dec        = 21.307[dB]
Irr_Atn_Noe        = 21.307[dB]
Irr_Noise          = WALTZ
Irr_Pwidth         = 0.115[ms]
Decoupling         = TRUE
Initial_Wait       = 1[s]
Noe                = TRUE
Noe_Time           = 2[s]
Repetition_Time    = 2.96468992[s]
```

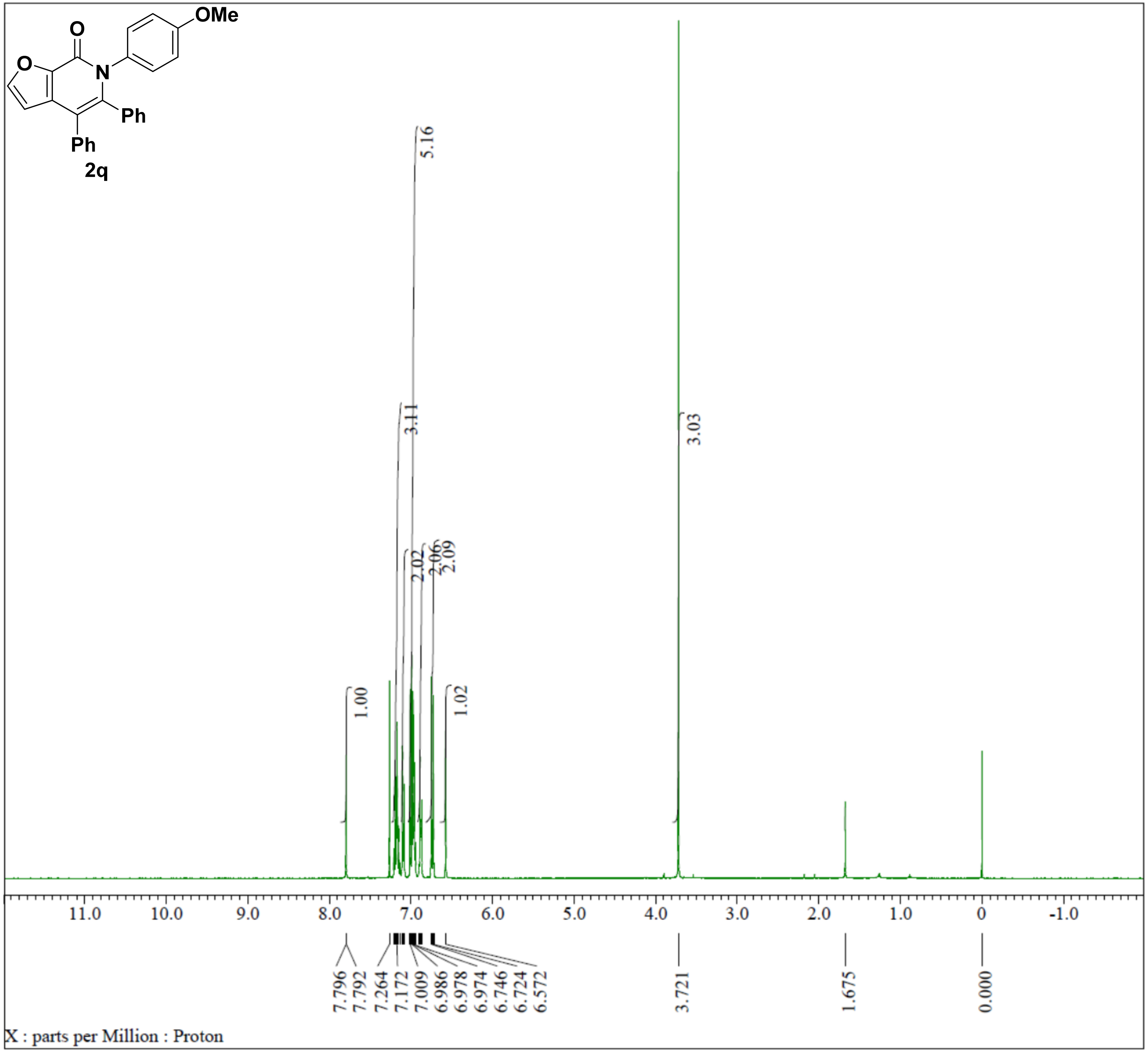


```
Filename           = AO-633 GPC_Proton-1-2.jdf
Author             = delta
Experiment         = proton.jxp
Sample_Id          = AO-633 GPC
Solvent            = CHLOROFORM-D
Creation_Time      =  7-MAR-2017 23:32:27
Revision_Time      =  8-MAR-2017 10:34:33
Current_Time       = 21-JUN-2017 22:39:20

Comment            = AO-633 GPC
Data_Format        = 1D COMPLEX
Dim_Size           = 13107
Dim_Title          = Proton
Dim_Units          = [ppm]
Dimensions         = X
Site               = JNM-ECS400
Spectrometer       = DELTA2_NMR

Field_Strength     = 9.389766[T] (400[MHz])
X_Acq_Duration     = 2.18365952[s]
X_Domain           = 1H
X_Freq             = 399.78219838[MHz]
X_Offset           = 5[ppm]
X_Points           = 16384
X_Prescans         = 1
X_Resolution       = 0.45794685[Hz]
X_Sweep            = 7.5030012[kHz]
X_Sweep_Clipped    = 6.00240096[kHz]
Irr_Domain         = Proton
Irr_Freq           = 399.78219838[MHz]
Irr_Offset         = 5[ppm]
Tri_Domain         = Proton
Tri_Freq           = 399.78219838[MHz]
Tri_Offset         = 5[ppm]
Clipped            = FALSE
Scans              = 8
Total_Scans        = 8

Relaxation_Delay   = 5[s]
Recvr_Gain         = 42
Temp_Get           = 16.2[dC]
X_90_Width         = 11.1[us]
X_Acq_Time         = 2.18365952[s]
X_Angle            = 45[deg]
X_Atn              = 1[dB]
X_Pulse            = 5.55[us]
Irr_Mode           = Off
Tri_Mode           = Off
Dante_Presat       = FALSE
Initial_Wait       = 1[s]
Repetition_Time    = 7.18365952[s]
```

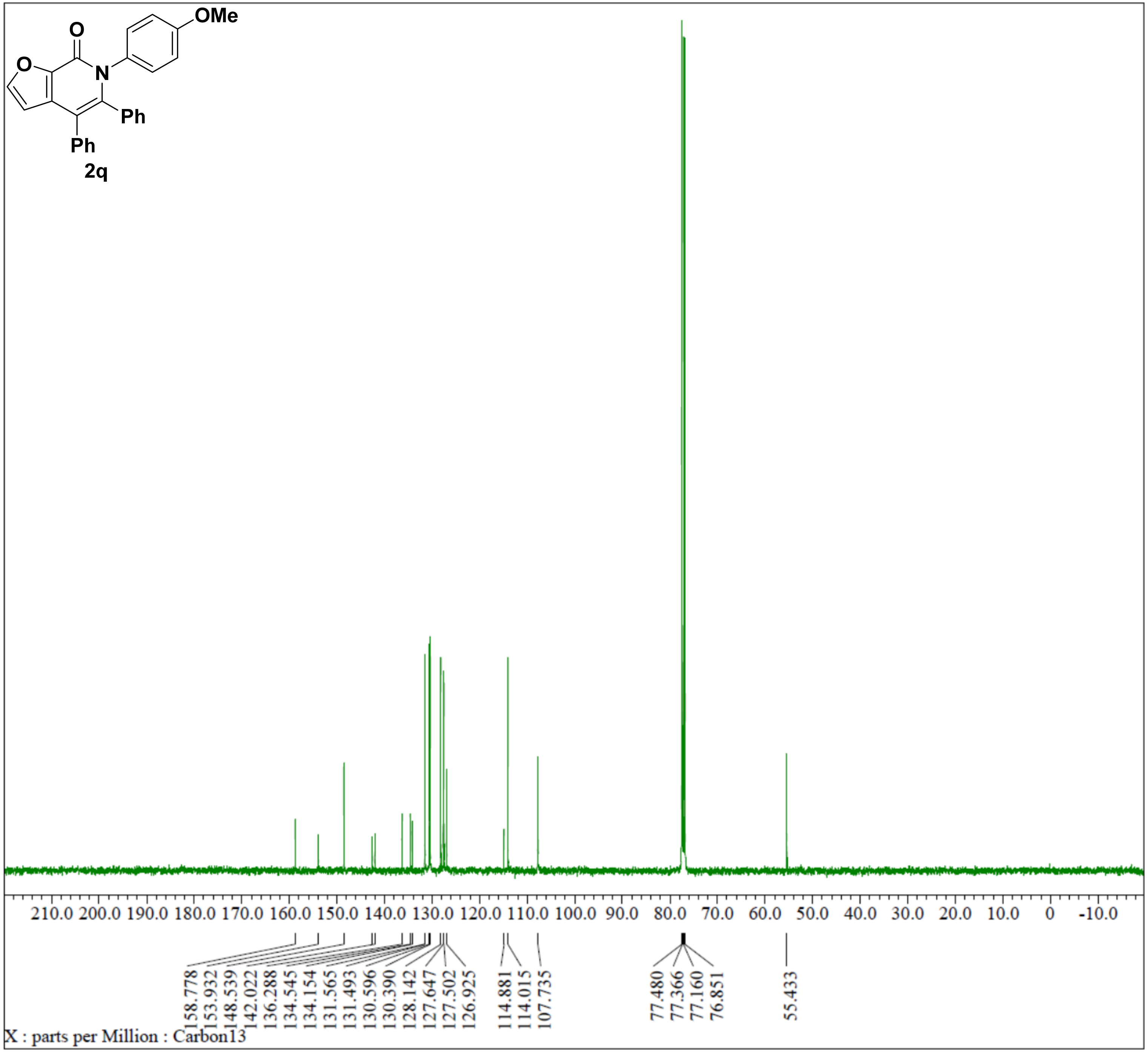


```
Filename           = AO-633 GPC_Carbon-1-1.jdf
Author             = delta
Experiment         = carbon.jxp
Sample_Id          = AO-633 GPC
Solvent            = CHLOROFORM-D
Creation_Time      =  8-MAR-2017 05:51:12
Revision_Time      =  8-MAR-2017 10:37:47
Current_Time       = 21-JUN-2017 22:39:52

Comment            = AO-633 GPC
Data_Format        = 1D COMPLEX
Dim_Size           = 26214
Dim_Title          = Carbon13
Dim_Units          = [ppm]
Dimensions         = X
Site               = JNM-ECS400
Spectrometer       = DELTA2_NMR

Field_Strength     = 9.389766[T] (400[MHz])
X_Acq_Duration     = 0.96468992[s]
X_Domain           = 13C
X_Freq             = 100.52530333[MHz]
X_Offset           = 100[ppm]
X_Points           = 32768
X_Prescans         = 4
X_Resolution       = 1.03660252[Hz]
X_Sweep            = 33.9673913[kHz]
X_Sweep_Clipped    = 27.17391304[kHz]
Irr_Domain         = Proton
Irr_Freq           = 399.78219838[MHz]
Irr_Offset         = 5[ppm]
Clipped            = FALSE
Scans              = 1024
Total_Scans        = 1024

Relaxation_Delay   = 2[s]
Recvr_Gain         = 60
Temp_Get           = 16.8[dC]
X_90_Width         = 9.9[us]
X_Acq_Time         = 0.96468992[s]
X_Angle            = 30[deg]
X_Atn              = 6[dB]
X_Pulse            = 3.3[us]
Irr_Atn_Dec        = 21.307[dB]
Irr_Atn_Noe        = 21.307[dB]
Irr_Noise          = WALTZ
Irr_Pwidth         = 0.115[ms]
Decoupling         = TRUE
Initial_Wait       = 1[s]
Noe                = TRUE
Noe_Time           = 2[s]
Repetition_Time    = 2.96468992[s]
```

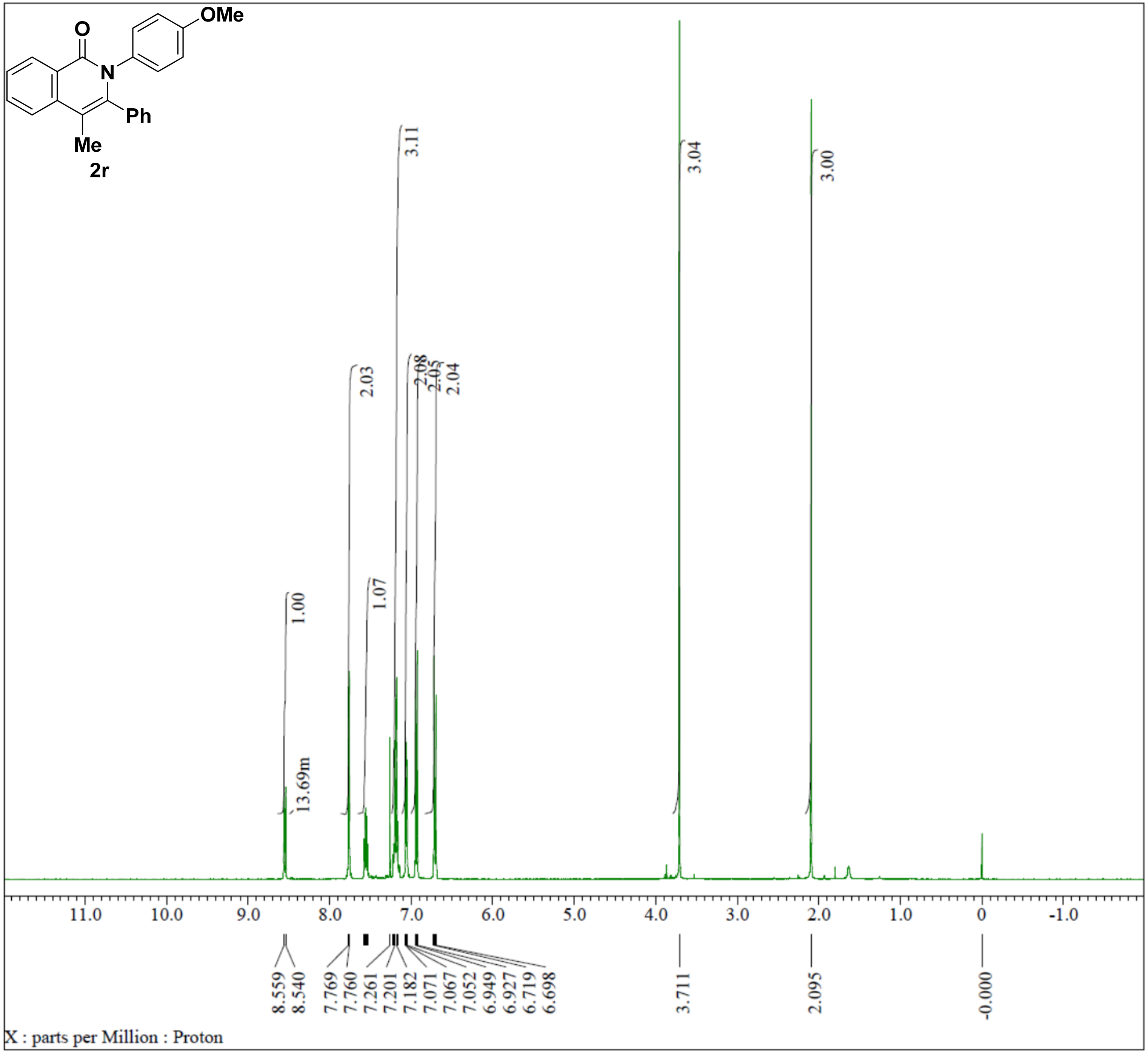


Filename = AO-759 column 24~35 again_
Author = delta
Experiment = proton.jxp
Sample_Id = AO-759 column 24~35 again
Solvent = CHLOROFORM-D
Creation_Time = 3-FEB-2017 22:52:42
Revision_Time = 22-JUN-2017 10:24:03
Current_Time = 22-JUN-2017 10:24:20

Comment = AO-759 column 24~35 again
Data_Format = 1D COMPLEX
Dim_Size = 13107
Dim_Title = Proton
Dim_Units = [ppm]
Dimensions = X
Site = JNM-ECS400
Spectrometer = DELTA2_NMR

Field_Strength = 9.389766[T] (400[MHz])
X_Acq_Duration = 2.18365952[s]
X_Domain = 1H
X_Freq = 399.78219838[MHz]
X_Offset = 5[ppm]
X_Points = 16384
X_Prescans = 1
X_Resolution = 0.45794685[Hz]
X_Sweep = 7.5030012[kHz]
X_Sweep_Clipped = 6.00240096[kHz]
Irr_Domain = Proton
Irr_Freq = 399.78219838[MHz]
Irr_Offset = 5[ppm]
Tri_Domain = Proton
Tri_Freq = 399.78219838[MHz]
Tri_Offset = 5[ppm]
Clipped = FALSE
Scans = 8
Total_Scans = 8

Relaxation_Delay = 5[s]
Recvr_Gain = 42
Temp_Get = 16.7[dC]
X_90_Width = 11.1[us]
X_Acq_Time = 2.18365952[s]
X_Angle = 45[deg]
X_Atn = 1[dB]
X_Pulse = 5.55[us]
Irr_Mode = Off
Tri_Mode = Off
Dante_Presat = FALSE
Initial_Wait = 1[s]
Repetition_Time = 7.18365952[s]

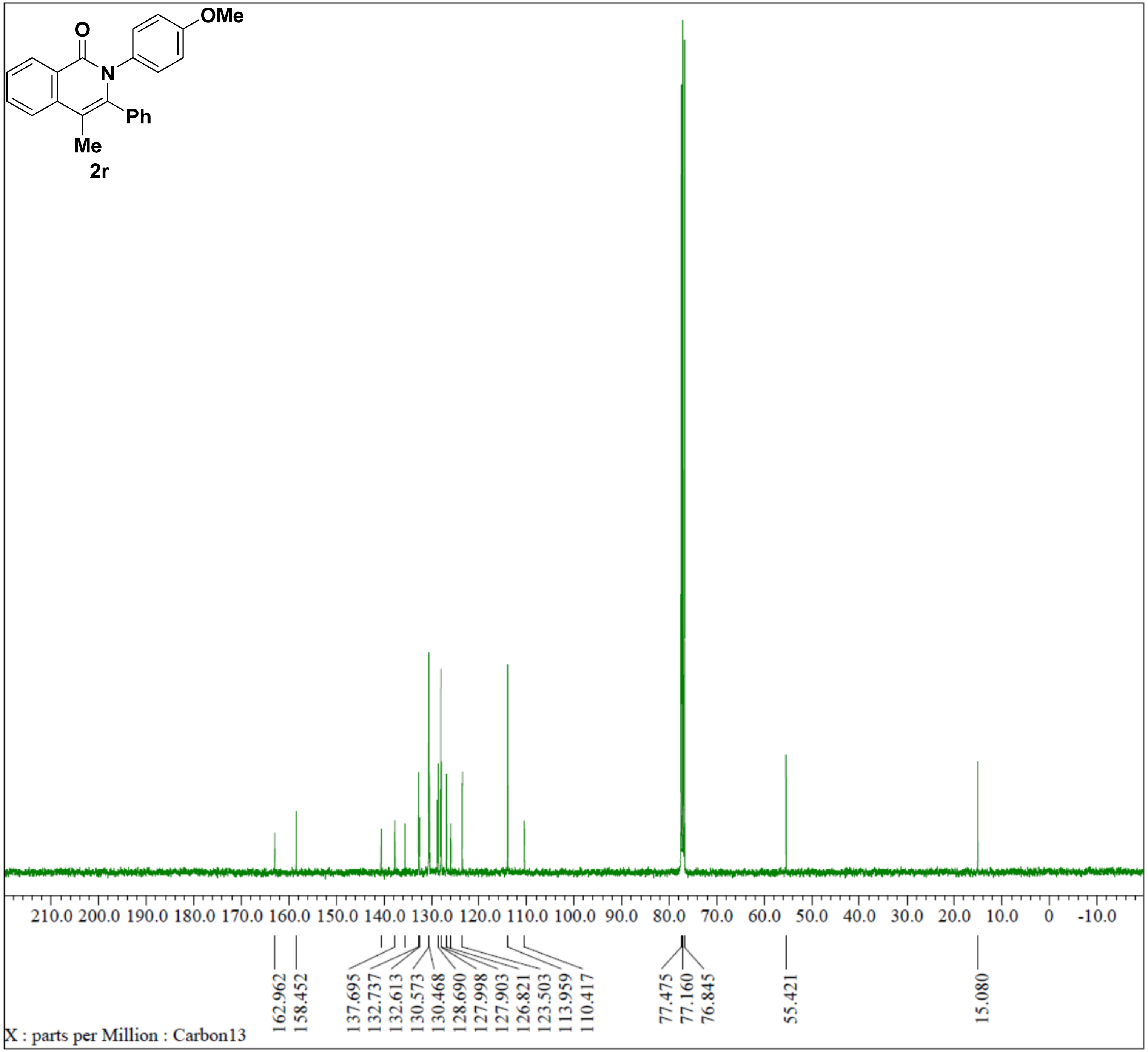


```
Filename           = AO-759 column 24~35 again_
Author             = delta
Experiment         = carbon.jxp
Sample_Id          = AO-759 column 24~35 again
Solvent            = CHLOROFORM-D
Creation_Time      =  4-FEB-2017 07:43:18
Revision_Time      =  4-FEB-2017 10:36:40
Current_Time       = 21-JUN-2017 22:43:43

Comment            = AO-759 column 24~35 again_
Data_Format        = 1D COMPLEX
Dim_Size           = 26214
Dim_Title          = Carbon13
Dim_Units          = [ppm]
Dimensions         = X
Site               = JNM-ECS400
Spectrometer       = DELTA2_NMR

Field_Strength     = 9.389766[T] (400[MHz])
X_Acq_Duration     = 1.04333312[s]
X_Domain           = 13C
X_Freq             = 100.52530333[MHz]
X_Offset           = 100[ppm]
X_Points           = 32768
X_Prescans         = 4
X_Resolution       = 0.95846665[Hz]
X_Sweep            = 31.40703518[kHz]
X_Sweep_Clipped    = 25.12562814[kHz]
Irr_Domain         = Proton
Irr_Freq           = 399.78219838[MHz]
Irr_Offset         = 5[ppm]
Clipped            = TRUE
Scans              = 1024
Total_Scans        = 1024

Relaxation_Delay   = 2[s]
Recvr_Gain         = 60
Temp_Get           = 15.6[dC]
X_90_Width         = 9.9[us]
X_Acq_Time         = 1.04333312[s]
X_Angle            = 30[deg]
X_Atn              = 6[dB]
X_Pulse            = 3.3[us]
Irr_Atn_Dec        = 21.307[dB]
Irr_Atn_Noe        = 21.307[dB]
Irr_Noise          = WALTZ
Irr_Pwidth         = 0.115[ms]
Decoupling         = TRUE
Initial_Wait       = 1[s]
Noe                = TRUE
Noe_Time           = 2[s]
Repetition_Time    = 3.04333312[s]
```

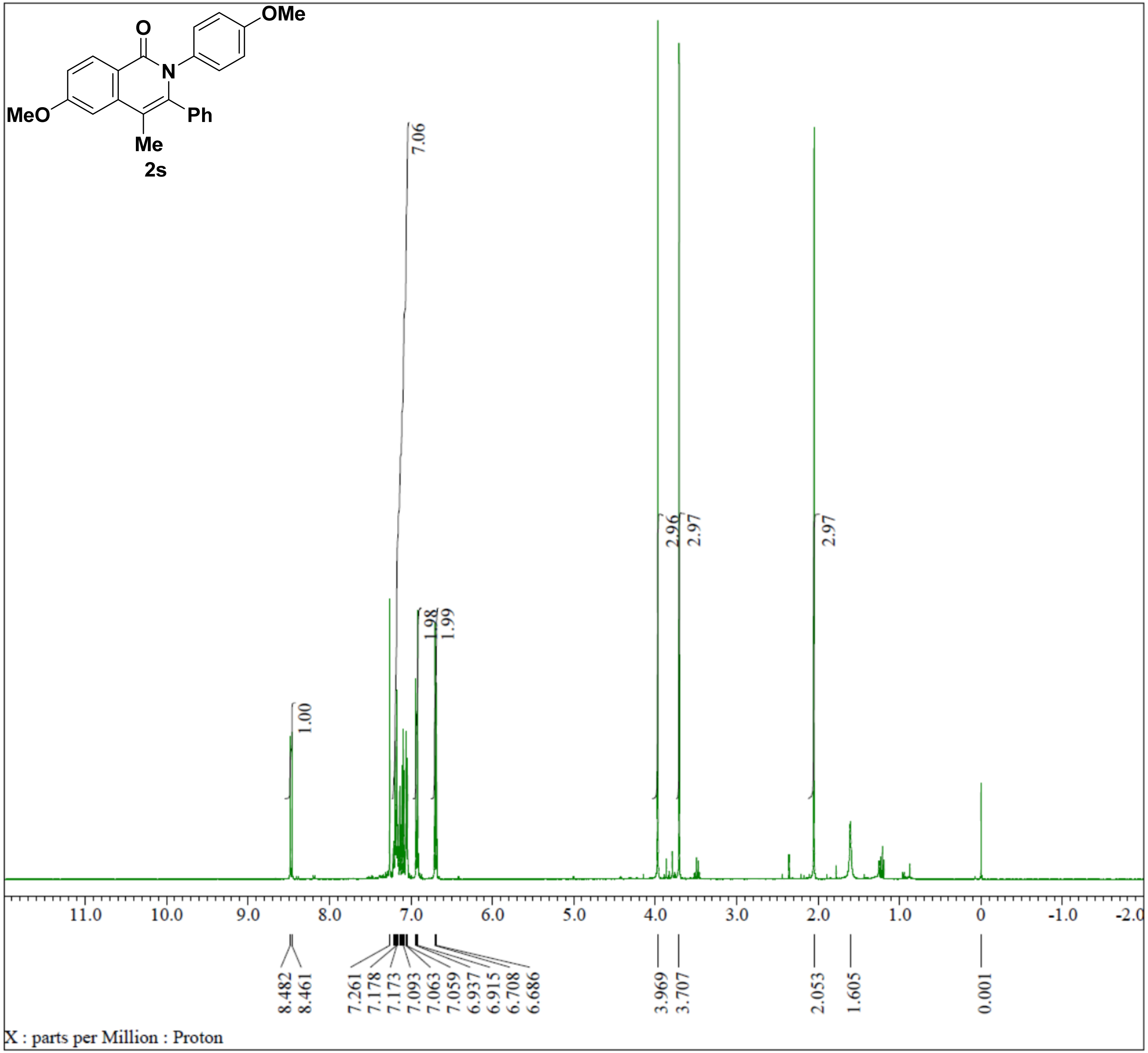


```
Filename          = AO-880 HPLC 4, 5_Proton-1-
Author            = delta
Experiment        = proton.jxp
Sample_Id         = AO-880 HPLC 4, 5
Solvent           = CHLOROFORM-D
Creation_Time     = 15-JUN-2017 13:51:05
Revision_Time     = 21-JUN-2017 22:57:46
Current_Time      = 21-JUN-2017 22:58:03

Comment           = AO-880 HPLC 4, 5
Data_Format       = 1D COMPLEX
Dim_Size          = 13107
Dim_Title         = Proton
Dim_Units         = [ppm]
Dimensions        = X
Site              = JNM-ECS400
Spectrometer      = DELTA2_NMR

Field_Strength    = 9.389766[T] (400[MHz])
X_Acq_Duration    = 2.18365952[s]
X_Domain          = 1H
X_Freq            = 399.78219838[MHz]
X_Offset          = 5[ppm]
X_Points          = 16384
X_Prescans        = 1
X_Resolution      = 0.45794685[Hz]
X_Sweep           = 7.5030012[kHz]
X_Sweep_Clipped   = 6.00240096[kHz]
Irr_Domain        = Proton
Irr_Freq          = 399.78219838[MHz]
Irr_Offset        = 5[ppm]
Tri_Domain        = Proton
Tri_Freq          = 399.78219838[MHz]
Tri_Offset        = 5[ppm]
Clipped           = FALSE
Scans             = 8
Total_Scans       = 8

Relaxation_Delay  = 5[s]
Recvr_Gain        = 44
Temp_Get          = 21.2[dC]
X_90_Width        = 11.1[us]
X_Acq_Time        = 2.18365952[s]
X_Angle           = 45[deg]
X_Atn             = 1[dB]
X_Pulse           = 5.55[us]
Irr_Mode          = Off
Tri_Mode          = Off
Dante_Presat      = FALSE
Initial_Wait      = 1[s]
Repetition_Time   = 7.18365952[s]
```

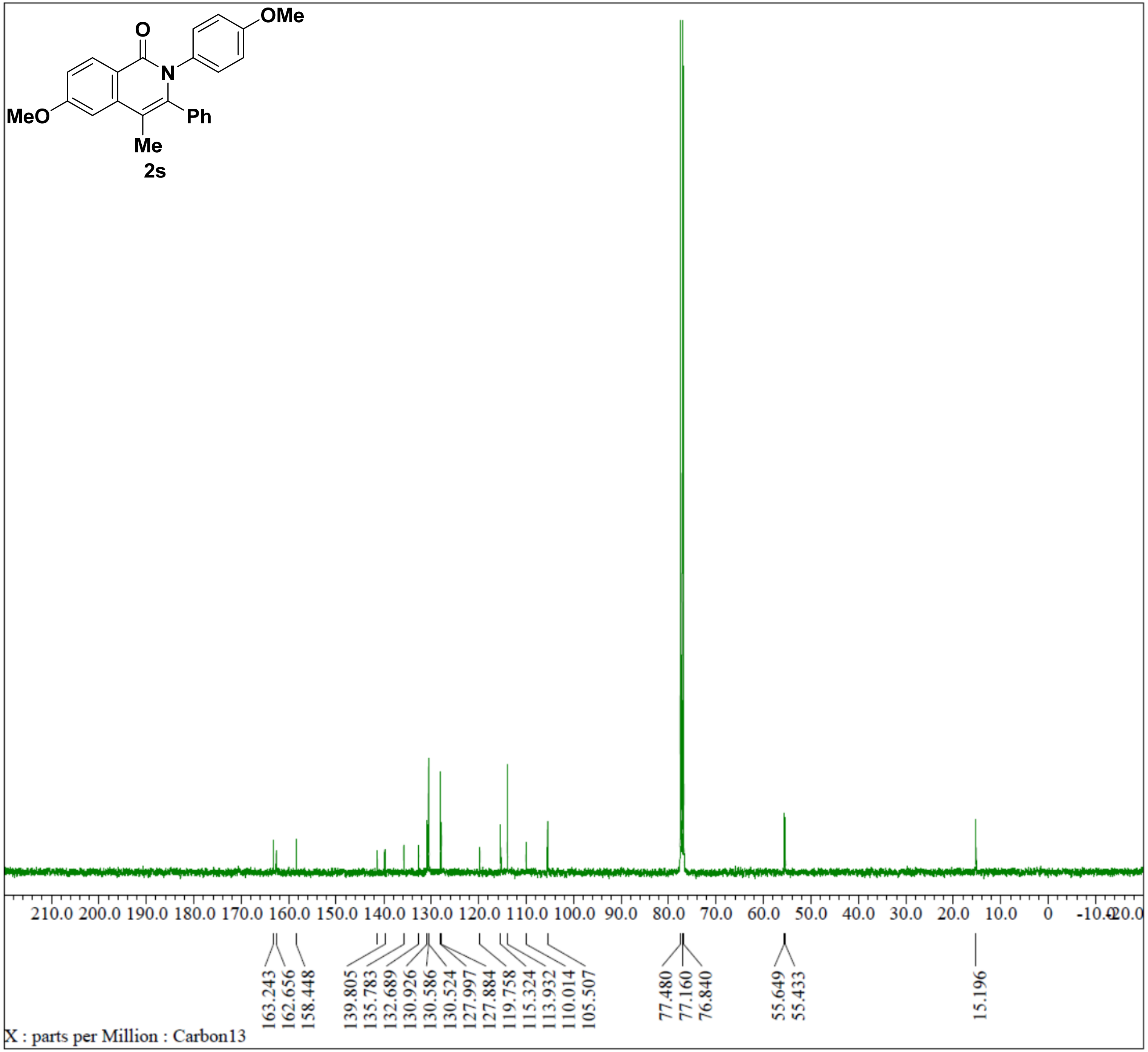


```
Filename           = AO-880 HPLC 4, 5_Carbon-1-
Author             = delta
Experiment         = carbon.jxp
Sample_Id          = AO-880 HPLC 4, 5
Solvent            = CHLOROFORM-D
Creation_Time      = 17-JUN-2017 02:07:50
Revision_Time      = 19-JUN-2017 14:34:34
Current_Time       = 21-JUN-2017 22:58:29

Comment            = AO-880 HPLC 4, 5
Data_Format        = 1D COMPLEX
Dim_Size           = 26214
Dim_Title          = Carbon13
Dim_Units          = [ppm]
Dimensions         = X
Site               = JNM-ECS400
Spectrometer       = DELTA2_NMR

Field_Strength     = 9.389766[T] (400[MHz])
X_Acq_Duration     = 0.96468992[s]
X_Domain           = 13C
X_Freq             = 100.52530333[MHz]
X_Offset           = 100[ppm]
X_Points           = 32768
X_Prescans         = 4
X_Resolution       = 1.03660252[Hz]
X_Sweep            = 33.9673913[kHz]
X_Sweep_Clipped    = 27.17391304[kHz]
Irr_Domain         = Proton
Irr_Freq           = 399.78219838[MHz]
Irr_Offset         = 5[ppm]
Clipped            = TRUE
Scans              = 1024
Total_Scans        = 1024

Relaxation_Delay   = 2[s]
Recvr_Gain         = 60
Temp_Get           = 19.9[dC]
X_90_Width         = 9.9[us]
X_Acq_Time         = 0.96468992[s]
X_Angle            = 30[deg]
X_Atn              = 6[dB]
X_Pulse            = 3.3[us]
Irr_Atn_Dec        = 21.307[dB]
Irr_Atn_Noe        = 21.307[dB]
Irr_Noise          = WALTZ
Irr_Pwidth         = 0.115[ms]
Decoupling         = TRUE
Initial_Wait       = 1[s]
Noe                = TRUE
Noe_Time           = 2[s]
Repetition_Time    = 2.96468992[s]
```

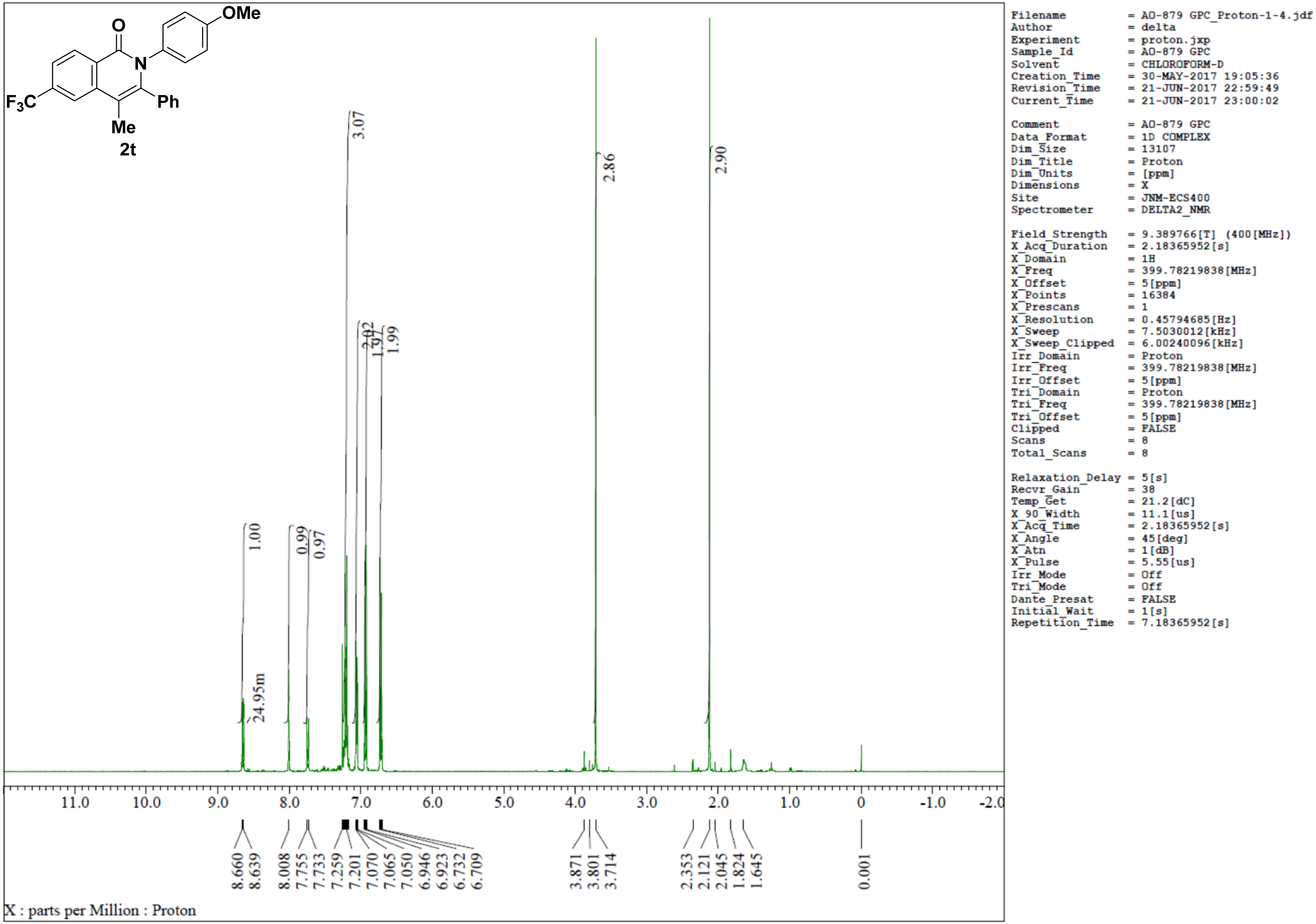

O
N
OMe
Ph
F3C
Me
2t
3.07
2.86
2.90
2.02
1.97
1.99
1.00
0.99
0.97
24.95m
11.0
10.0
9.0
8.0
7.0
6.0
5.0
4.0
3.0
2.0
1.0
0
-1.0
-2.0
8.660
8.639
8.008
7.755
7.733
7.259
7.201
7.070
7.065
7.050
6.946
6.923
6.732
6.709
3.871
3.801
3.714
2.353
2.121
2.045
1.824
1.645
0.001
X : parts per Million : Proton
Filename = AO-879 GPC_Proton-1-4.jdf
Author = delta
Experiment = proton.jxp
Sample_Id = AO-879 GPC
Solvent = CHLOROFORM-D
Creation_Time = 30-MAY-2017 19:05:36
Revision_Time = 21-JUN-2017 22:59:49
Current_Time = 21-JUN-2017 23:00:02
Comment = AO-879 GPC
Data_Format = 1D COMPLEX
Dim_Size = 13107
Dim_Title = Proton
Dim_Units = [ppm]
Dimensions = X
Site = JNM-ECS400
Spectrometer = DELTA2_NMR
Field_Strength = 9.389766[T] (400[MHz])
X_Acq_Duration = 2.18365952[s]
X_Domain = 1H
X_Freq = 399.78219838[MHz]
X_Offset = 5[ppm]
X_Points = 16384
X_Prescans = 1
X_Resolution = 0.45794685[Hz]
X_Sweep = 7.5030012[kHz]
X_Sweep_Clipped = 6.00240096[kHz]
Irr_Domain = Proton
Irr_Freq = 399.78219838[MHz]
Irr_Offset = 5[ppm]
Tri_Domain = Proton
Tri_Freq = 399.78219838[MHz]
Tri_Offset = 5[ppm]
Clipped = FALSE
Scans = 8
Total_Scans = 8
Relaxation_Delay = 5[s]
Recvr_Gain = 38
Temp_Get = 21.2[dC]
X_90_Width = 11.1[us]
X_Acq_Time = 2.18365952[s]
X_Angle = 45[deg]
X_Atn = 1[dB]
X_Pulse = 5.55[us]
Irr_Mode = Off
Tri_Mode = Off
Dante_Presat = FALSE
Initial_Wait = 1[s]
Repetition_Time = 7.18365952[s]

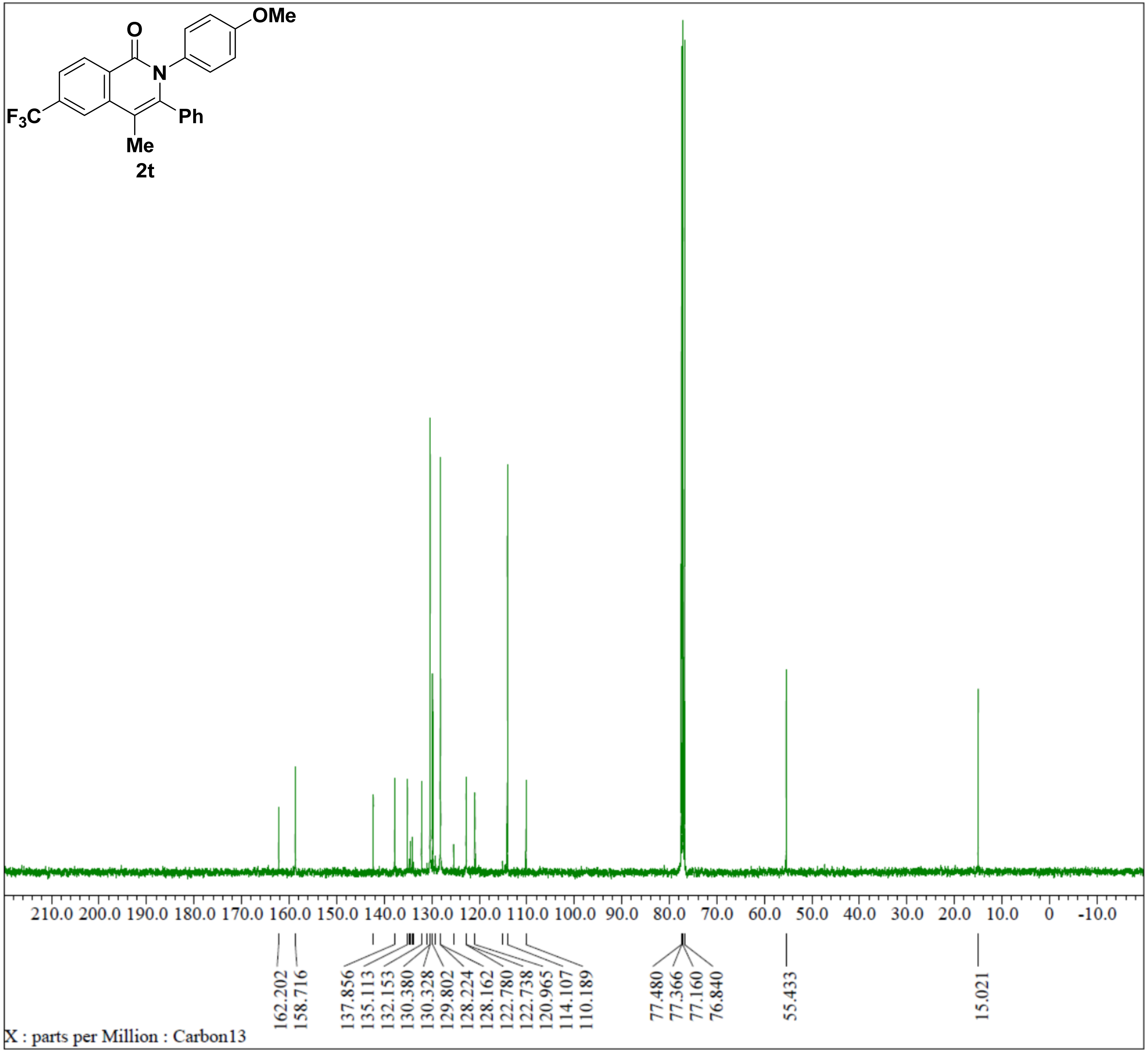


```
Filename            = AO-879 GPC_Carbon-1-1.jdf
Author              = delta
Experiment          = carbon.jxp
Sample_Id           = AO-889 GPC
Solvent             = CHLOROFORM-D
Creation_Time       =  1-JUN-2017 04:05:12
Revision_Time       = 16-JUN-2017 15:08:32
Current_Time        = 21-JUN-2017 22:56:50

Comment             = AO-889 GPC
Data_Format         = 1D COMPLEX
Dim_Size            = 26214
Dim_Title           = Carbon13
Dim_Units           = [ppm]
Dimensions          = X
Site                = JNM-ECS400
Spectrometer        = DELTA2_NMR

Field_Strength      = 9.389766[T] (400[MHz])
X_Acq_Duration      = 0.96468992[s]
X_Domain            = 13C
X_Freq              = 100.52530333[MHz]
X_Offset            = 100[ppm]
X_Points            = 32768
X_Prescans          = 4
X_Resolution        = 1.03660252[Hz]
X_Sweep             = 33.9673913[kHz]
X_Sweep_Clipped     = 27.17391304[kHz]
Irr_Domain          = Proton
Irr_Freq            = 399.78219838[MHz]
Irr_Offset          = 5[ppm]
Clipped             = FALSE
Scans               = 1024
Total_Scans         = 1024

Relaxation_Delay    = 2[s]
Recvr_Gain          = 58
Temp_Get            = 22.1[dC]
X_90_Width          = 9.9[us]
X_Acq_Time          = 0.96468992[s]
X_Angle             = 30[deg]
X_Atn               = 6[dB]
X_Pulse             = 3.3[us]
Irr_Atn_Dec         = 21.307[dB]
Irr_Atn_Noe         = 21.307[dB]
Irr_Noise           = WALTZ
Irr_Pwidth          = 0.115[ms]
Decoupling          = TRUE
Initial_Wait        = 1[s]
Noe                 = TRUE
Noe_Time            = 2[s]
Repetition_Time     = 2.96468992[s]
```

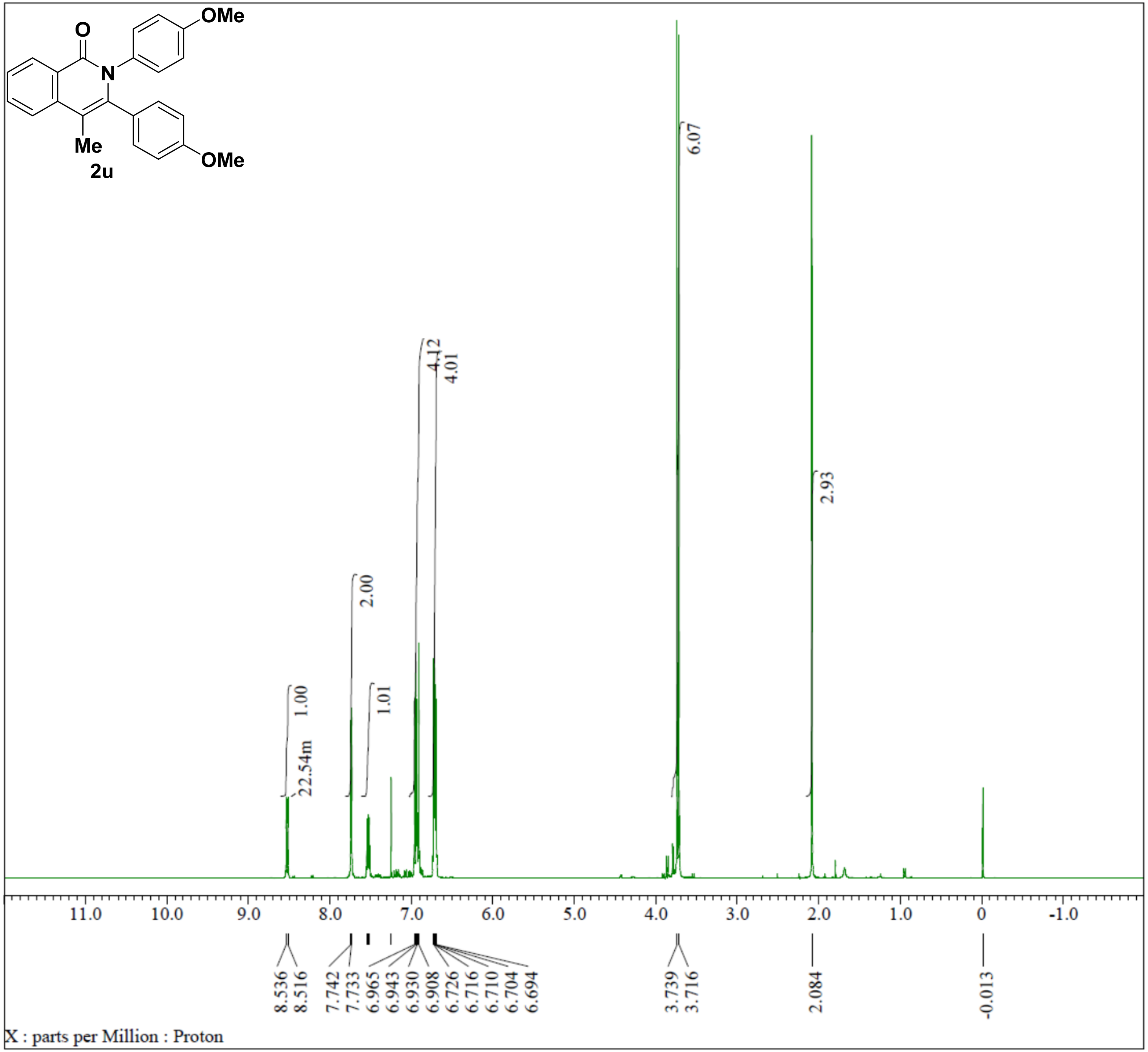


```
Filename          = AO-794 GPC again_Proton-1-
Author            = delta
Experiment        = proton.jxp
Sample_Id         = AO-794 GPC  again
Solvent           = CHLOROFORM-D
Creation_Time     = 25-FEB-2017 19:37:36
Revision_Time     = 21-JUN-2017 22:53:41
Current_Time      = 21-JUN-2017 22:54:01

Comment           = AO-794 GPC  again
Data_Format       = 1D COMPLEX
Dim_Size          = 13107
Dim_Title         = Proton
Dim_Units         = [ppm]
Dimensions        = X
Site              = JNM-ECS400
Spectrometer      = DELTA2_NMR

Field_Strength    = 9.389766[T] (400[MHz])
X_Acq_Duration    = 2.18365952[s]
X_Domain          = 1H
X_Freq            = 399.78219838[MHz]
X_Offset          = 5[ppm]
X_Points          = 16384
X_Prescans        = 1
X_Resolution      = 0.45794685[Hz]
X_Sweep           = 7.5030012[kHz]
X_Sweep_Clipped   = 6.00240096[kHz]
Irr_Domain        = Proton
Irr_Freq          = 399.78219838[MHz]
Irr_Offset        = 5[ppm]
Tri_Domain        = Proton
Tri_Freq          = 399.78219838[MHz]
Tri_Offset        = 5[ppm]
Clipped           = FALSE
Scans             = 8
Total_Scans       = 8

Relaxation_Delay  = 5[s]
Recvr_Gain        = 36
Temp_Get          = 16.4[dC]
X_90_Width        = 11.1[us]
X_Acq_Time        = 2.18365952[s]
X_Angle           = 45[deg]
X_Atn             = 1[dB]
X_Pulse           = 5.55[us]
Irr_Mode          = Off
Tri_Mode          = Off
Dante_Presat      = FALSE
Initial_Wait      = 1[s]
Repetition_Time   = 7.18365952[s]
```

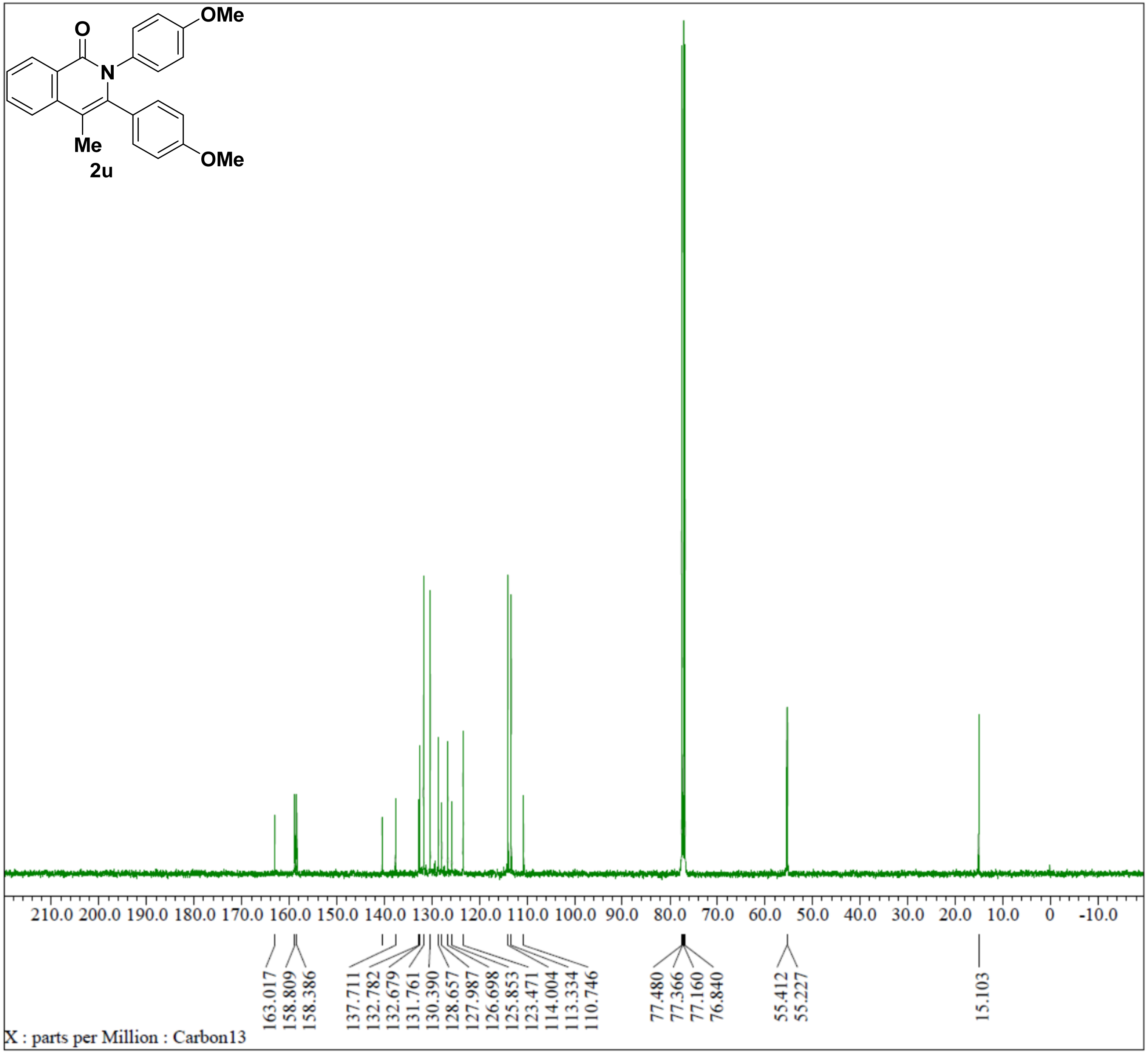


| | |
|---|---|
| Filename | = AO-794 GPC again_Carbon-1- |
| Author | = delta |
| Experiment | = carbon.jxp |
| Sample_Id | = AO-794 GPC again |
| Solvent | = CHLOROFORM-D |
| Creation_Time | = 26-FEB-2017 07:05:21 |
| Revision_Time | = 26-FEB-2017 15:14:26 |
| Current_Time | = 21-JUN-2017 22:50:01 |
| Comment | = AO-794 GPC again |
| Data_Format | = 1D COMPLEX |
| Dim_Size | = 26214 |
| Dim_Title | = Carbon13 |
| Dim_Units | = [ppm] |
| Dimensions | = X |
| Site | = JNM-ECS400 |
| Spectrometer | = DELTA2_NMR |
| Field_Strength | = 9.389766[T] (400[MHz]) |
| X_Acq_Duration | = 0.96468992[s] |
| X_Domain | = 13C |
| X_Freq | = 100.52530333[MHz] |
| X_Offset | = 100[ppm] |
| X_Points | = 32768 |
| X_Prescans | = 4 |
| X_Resolution | = 1.03660252[Hz] |
| X_Sweep | = 33.9673913[kHz] |
| X_Sweep_Clipped | = 27.17391304[kHz] |
| Irr_Domain | = Proton |
| Irr_Freq | = 399.78219838[MHz] |
| Irr_Offset | = 5[ppm] |
| Clipped | = FALSE |
| Scans | = 1024 |
| Total_Scans | = 1024 |
| Relaxation_Delay | = 2[s] |
| Recvr_Gain | = 50 |
| Temp_Get | = 16.6[dC] |
| X_90_Width | = 9.9[us] |
| X_Acq_Time | = 0.96468992[s] |
| X_Angle | = 30[deg] |
| X_Atn | = 6[dB] |
| X_Pulse | = 3.3[us] |
| Irr_Atn_Dec | = 21.307[dB] |
| Irr_Atn_Noe | = 21.307[dB] |
| Irr_Noise | = WALTZ |
| Irr_Pwidth | = 0.115[ms] |
| Decoupling | = TRUE |
| Initial_Wait | = 1[s] |
| Noe | = TRUE |
| Noe_Time | = 2[s] |
| Repetition_Time | = 2.96468992[s] |

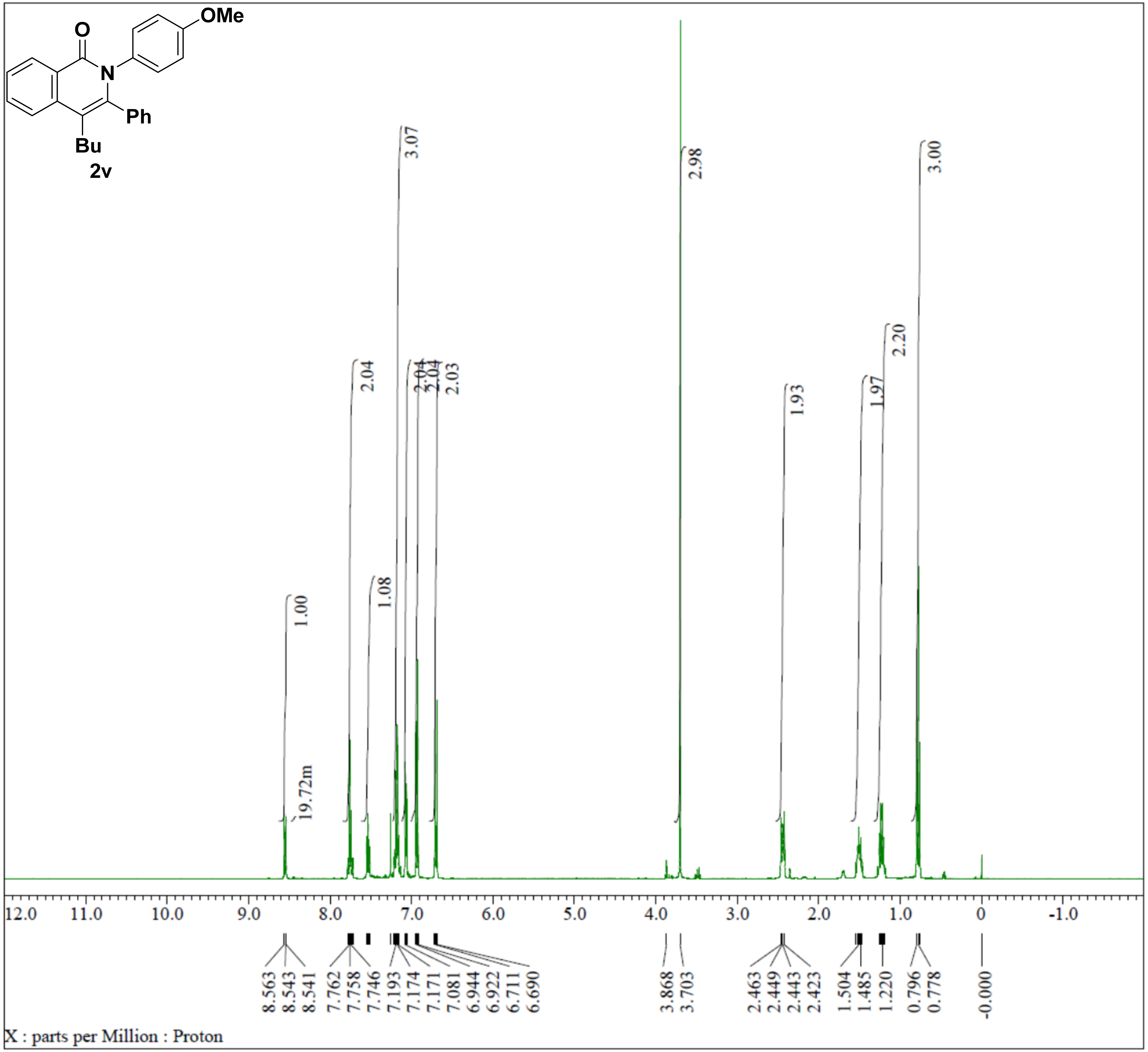


```
Filename          = AO-760 columnn 21~32-1-2.j
Author            = delta
Experiment        = proton.jxp
Sample_Id         = AO-760 columnn 21~32
Solvent           = CHLOROFORM-D
Creation_Time     = 31-JAN-2017 19:53:09
Revision_Time     = 21-JUN-2017 22:48:19
Current_Time      = 21-JUN-2017 22:48:41

Comment           = AO-760 columnn 21~32
Data_Format       = 1D COMPLEX
Dim_Size          = 13107
Dim_Title         = Proton
Dim_Units         = [ppm]
Dimensions        = X
Site              = JNM-ECS400
Spectrometer      = DELTA2_NMR

Field_Strength    = 9.389766[T] (400[MHz])
X_Acq_Duration    = 2.18365952[s]
X_Domain          = 1H
X_Freq            = 399.78219838[MHz]
X_Offset          = 5[ppm]
X_Points          = 16384
X_Prescans        = 1
X_Resolution      = 0.45794685[Hz]
X_Sweep           = 7.5030012[kHz]
X_Sweep_Clipped   = 6.00240096[kHz]
Irr_Domain        = Proton
Irr_Freq          = 399.78219838[MHz]
Irr_Offset        = 5[ppm]
Tri_Domain        = Proton
Tri_Freq          = 399.78219838[MHz]
Tri_Offset        = 5[ppm]
Clipped           = FALSE
Scans             = 8
Total_Scans       = 8

Relaxation_Delay  = 5[s]
Recvr_Gain        = 34
Temp_Get          = 16[dC]
X_90_Width        = 11.1[us]
X_Acq_Time        = 2.18365952[s]
X_Angle           = 45[deg]
X_Atn             = 1[dB]
X_Pulse           = 5.55[us]
Irr_Mode          = Off
Tri_Mode          = Off
Dante_Presat      = FALSE
Initial_Wait      = 1[s]
Repetition_Time   = 7.18365952[s]
```

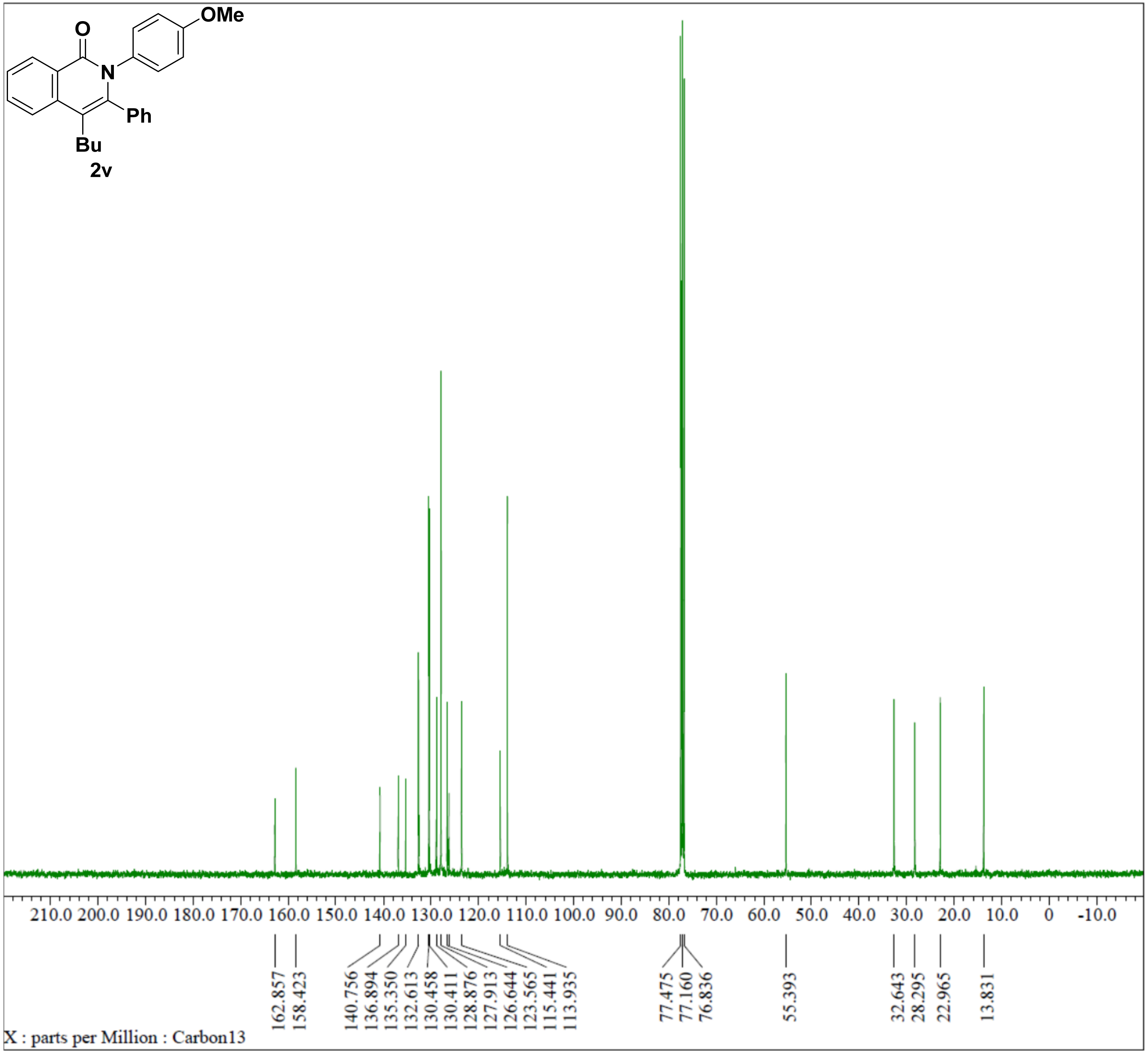


```
Filename            = AO-760 column 21~32_Carbon
Author              = delta
Experiment          = carbon.jxp
Sample_Id           = AO-760 column 21~32
Solvent             = CHLOROFORM-D
Creation_Time       =  4-FEB-2017 08:42:11
Revision_Time       =  4-FEB-2017 10:37:26
Current_Time        = 21-JUN-2017 22:44:35

Comment             = AO-760 column 21~32_13C
Data_Format         = 1D COMPLEX
Dim_Size            = 26214
Dim_Title           = Carbon13
Dim_Units           = [ppm]
Dimensions          = X
Site                = JNM-ECS400
Spectrometer        = DELTA2_NMR

Field_Strength      = 9.389766[T] (400[MHz])
X_Acq_Duration      = 1.04333312[s]
X_Domain            = 13C
X_Freq              = 100.52530333[MHz]
X_Offset            = 100[ppm]
X_Points            = 32768
X_Prescans          = 4
X_Resolution        = 0.95846665[Hz]
X_Sweep             = 31.40703518[kHz]
X_Sweep_Clipped     = 25.12562814[kHz]
Irr_Domain          = Proton
Irr_Freq            = 399.78219838[MHz]
Irr_Offset          = 5[ppm]
Clipped             = FALSE
Scans               = 1024
Total_Scans         = 1024

Relaxation_Delay    = 2[s]
Recvr_Gain          = 60
Temp_Get            = 16[dC]
X_90_Width          = 9.9[us]
X_Acq_Time          = 1.04333312[s]
X_Angle             = 30[deg]
X_Atn               = 6[dB]
X_Pulse             = 3.3[us]
Irr_Atn_Dec         = 21.307[dB]
Irr_Atn_Noe         = 21.307[dB]
Irr_Noise           = WALTZ
Irr_Pwidth          = 0.115[ms]
Decoupling          = TRUE
Initial_Wait        = 1[s]
Noe                 = TRUE
Noe_Time            = 2[s]
Repetition_Time     = 3.04333312[s]
```

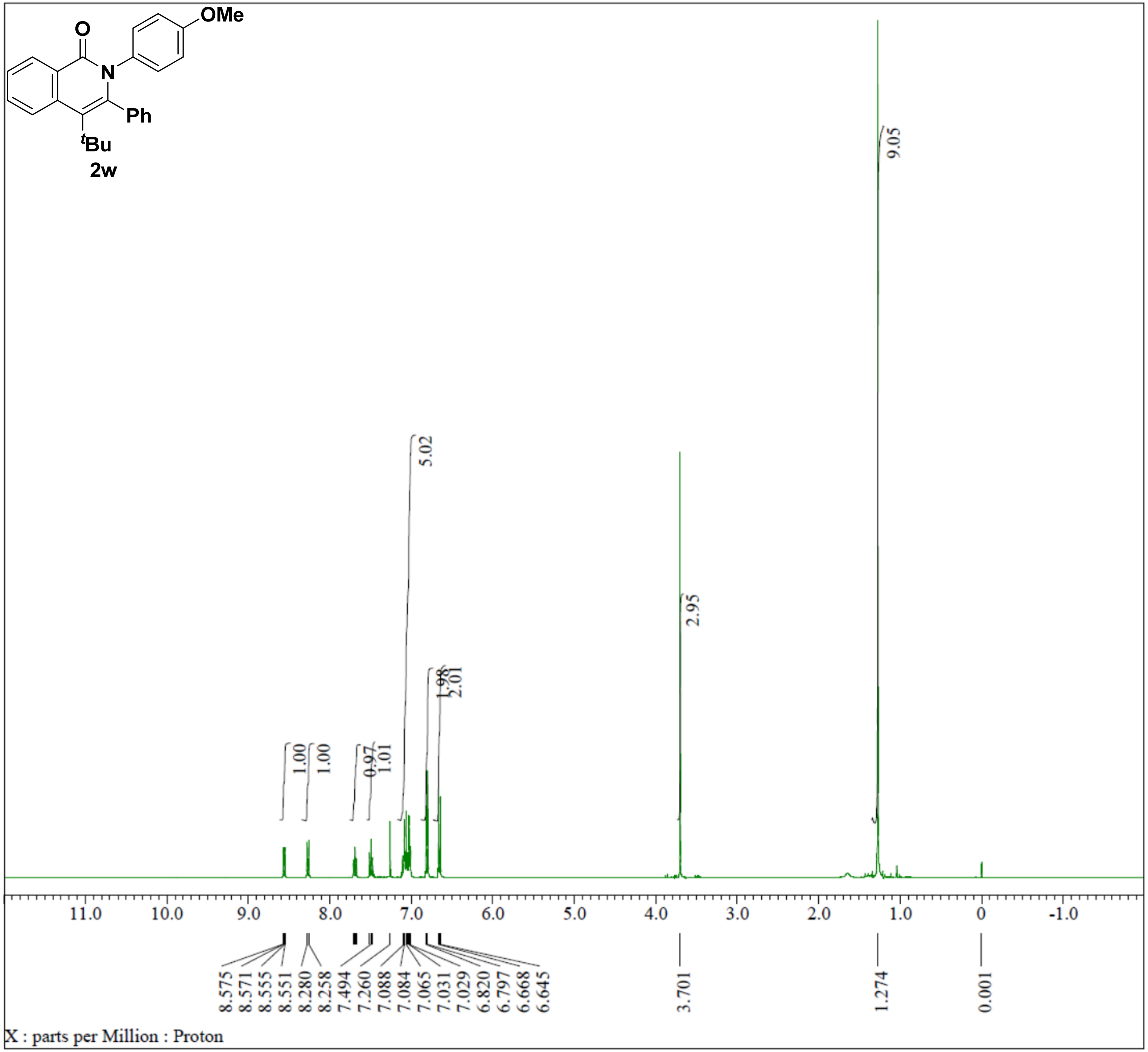


```
Filename          = AO-889 column f27-36_Proto
Author            = delta
Experiment        = proton.jxp
Sample_Id         = AO-889 column f27-36
Solvent           = CHLOROFORM-D
Creation_Time     =  9-JUN-2017 21:31:13
Revision_Time     = 16-JUN-2017 15:16:01
Current_Time      = 21-JUN-2017 23:01:08

Comment           = AO-889 column f27-36_1H
Data_Format       = 1D COMPLEX
Dim_Size          = 13107
Dim_Title         = Proton
Dim_Units         = [ppm]
Dimensions        = X
Site              = JNM-ECS400
Spectrometer      = DELTA2_NMR

Field_Strength    = 9.389766[T] (400[MHz])
X_Acq_Duration    = 2.18365952[s]
X_Domain          = 1H
X_Freq            = 399.78219838[MHz]
X_Offset          = 5[ppm]
X_Points          = 16384
X_Prescans        = 1
X_Resolution      = 0.45794685[Hz]
X_Sweep           = 7.5030012[kHz]
X_Sweep_Clipped   = 6.00240096[kHz]
Irr_Domain        = Proton
Irr_Freq          = 399.78219838[MHz]
Irr_Offset        = 5[ppm]
Tri_Domain        = Proton
Tri_Freq          = 399.78219838[MHz]
Tri_Offset        = 5[ppm]
Clipped           = FALSE
Scans             = 8
Total_Scans       = 8

Relaxation_Delay  = 5[s]
Recvr_Gain        = 40
Temp_Get          = 20.5[dC]
X_90_Width        = 11.1[us]
X_Acq_Time        = 2.18365952[s]
X_Angle           = 45[deg]
X_Atn             = 1[dB]
X_Pulse           = 5.55[us]
Irr_Mode          = Off
Tri_Mode          = Off
Dante_Presat      = FALSE
Initial_Wait      = 1[s]
Repetition_Time   = 7.18365952[s]
```

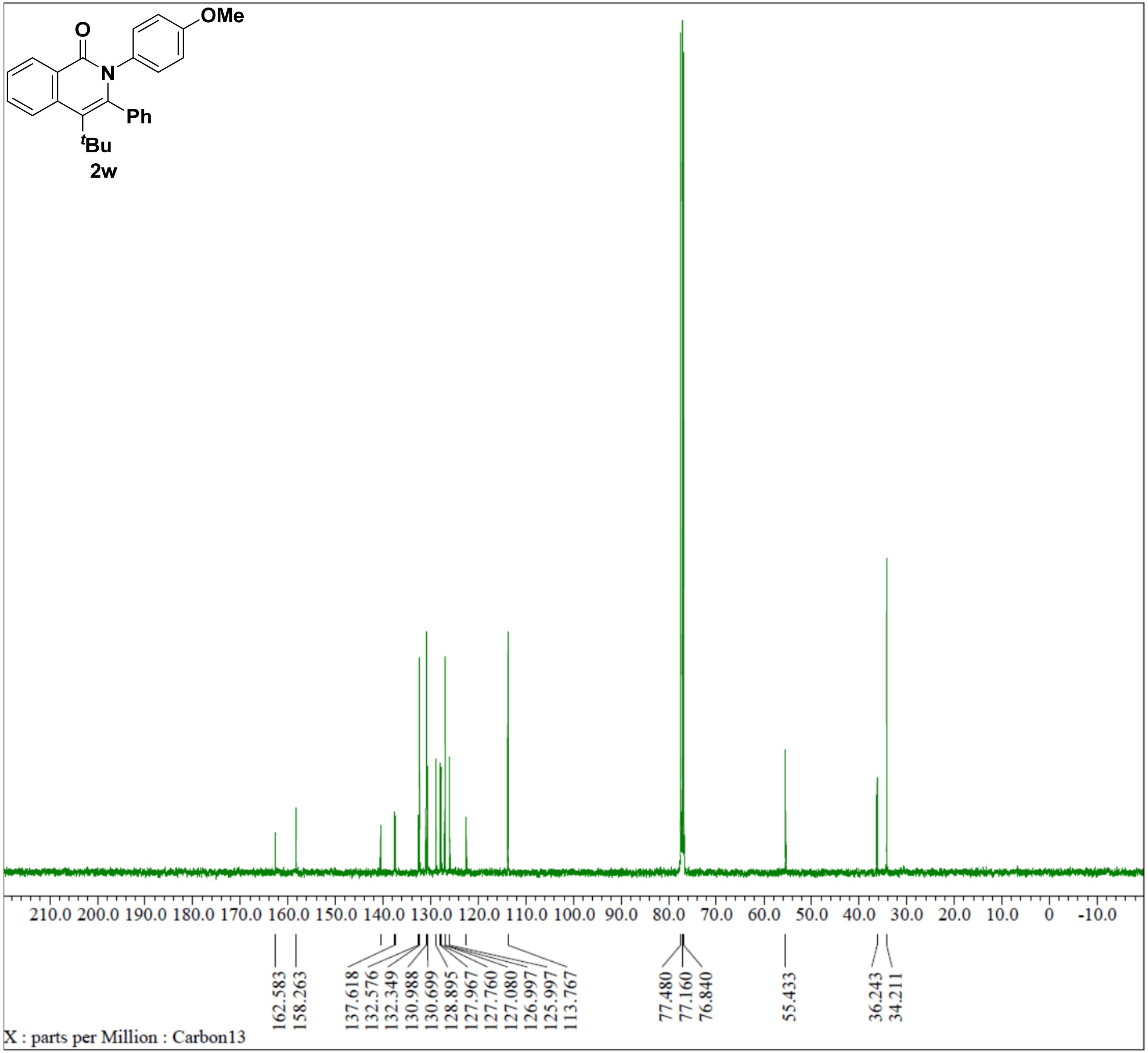


```
Filename          = AO-889 column f27-36_Carbo
Author            = delta
Experiment        = carbon.jxp
Sample_Id         = AO-889 column f27-36
Solvent           = CHLOROFORM-D
Creation_Time     = 10-JUN-2017 03:05:10
Revision_Time     = 16-JUN-2017 15:16:32
Current_Time      = 21-JUN-2017 23:00:38

Comment           = AO-889 column f27-36
Data_Format       = 1D COMPLEX
Dim_Size          = 26214
Dim_Title         = Carbon13
Dim_Units         = [ppm]
Dimensions        = X
Site              = JNM-ECS400
Spectrometer      = DELTA2_NMR

Field_Strength    = 9.389766[T] (400[MHz])
X_Acq_Duration    = 0.96468992[s]
X_Domain          = 13C
X_Freq            = 100.52530333[MHz]
X_Offset          = 100[ppm]
X_Points          = 32768
X_Prescans        = 4
X_Resolution      = 1.03660252[Hz]
X_Sweep           = 33.9673913[kHz]
X_Sweep_Clipped   = 27.17391304[kHz]
Irr_Domain        = Proton
Irr_Freq          = 399.78219838[MHz]
Irr_Offset        = 5[ppm]
Clipped           = TRUE
Scans             = 1024
Total_Scans       = 1024

Relaxation_Delay  = 2[s]
Recvr_Gain        = 60
Temp_Get          = 20.5[dC]
X_90_Width        = 9.9[us]
X_Acq_Time        = 0.96468992[s]
X_Angle           = 30[deg]
X_Atn             = 6[dB]
X_Pulse           = 3.3[us]
Irr_Atn_Dec       = 21.307[dB]
Irr_Atn_Noe       = 21.307[dB]
Irr_Noise         = WALTZ
Irr_Pwidth        = 0.115[ms]
Decoupling        = TRUE
Initial_Wait      = 1[s]
Noe               = TRUE
Noe_Time          = 2[s]
Repetition_Time   = 2.96468992[s]
```

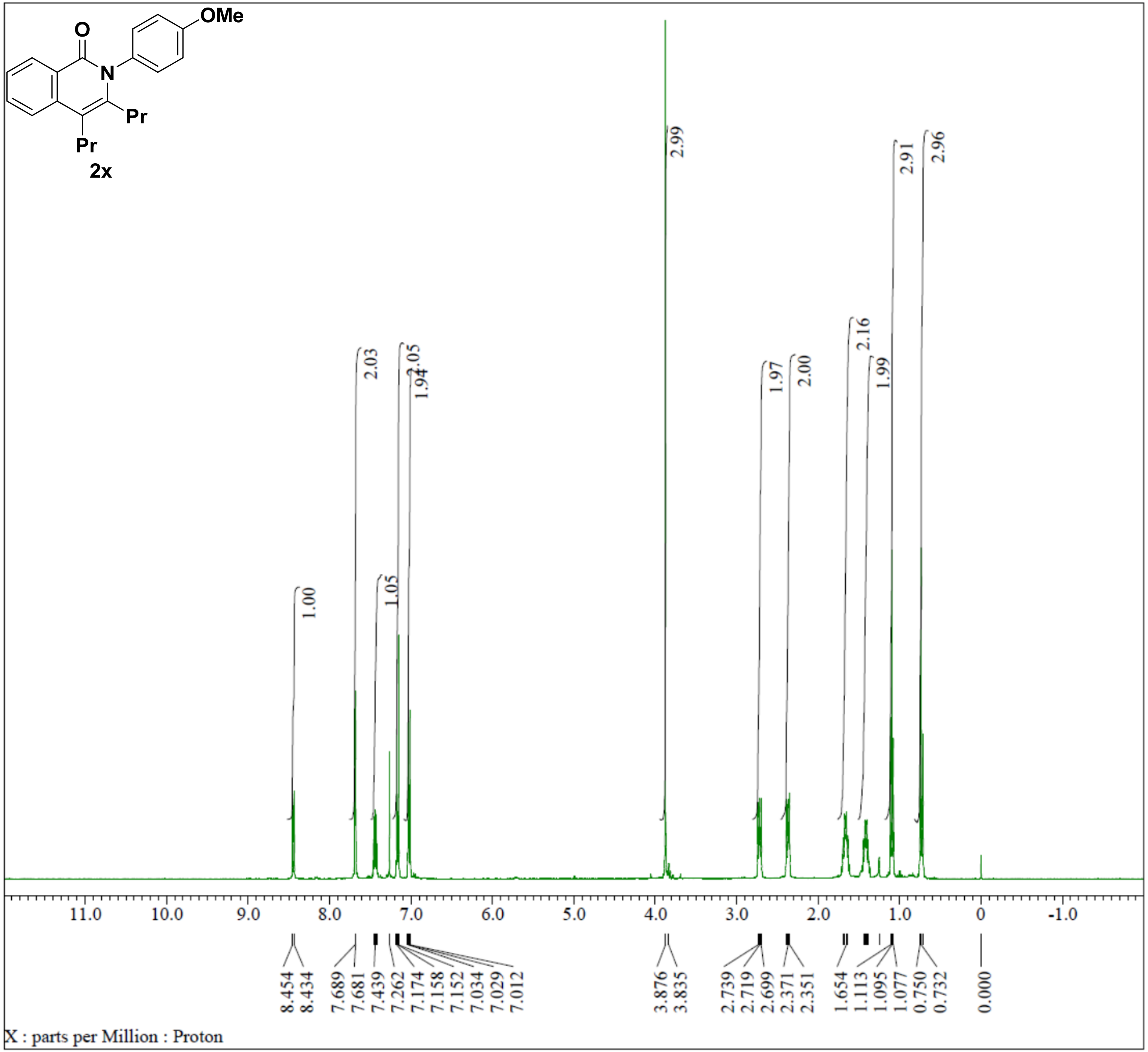


```
Filename            = AO-766 column 33~39_Proton
Author              = delta
Experiment          = proton.jxp
Sample_Id           = AO-766 column 33~39
Solvent             = CHLOROFORM-D
Creation_Time       = 13-FEB-2017 14:43:02
Revision_Time       = 21-JUN-2017 22:46:43
Current_Time        = 21-JUN-2017 22:46:54

Comment             = AO-766 column 33~39
Data_Format         = 1D COMPLEX
Dim_Size            = 13107
Dim_Title           = Proton
Dim_Units           = [ppm]
Dimensions          = X
Site                = JNM-ECS400
Spectrometer        = DELTA2_NMR

Field_Strength      = 9.389766[T] (400[MHz])
X_Acq_Duration      = 2.18365952[s]
X_Domain            = 1H
X_Freq              = 399.78219838[MHz]
X_Offset            = 5[ppm]
X_Points            = 16384
X_Prescans          = 1
X_Resolution        = 0.45794685[Hz]
X_Sweep             = 7.5030012[kHz]
X_Sweep_Clipped     = 6.00240096[kHz]
Irr_Domain          = Proton
Irr_Freq            = 399.78219838[MHz]
Irr_Offset          = 5[ppm]
Tri_Domain          = Proton
Tri_Freq            = 399.78219838[MHz]
Tri_Offset          = 5[ppm]
Clipped             = FALSE
Scans               = 8
Total_Scans         = 8

Relaxation_Delay    = 5[s]
Recvr_Gain          = 38
Temp_Get            = 14.9[dC]
X_90_Width          = 11.1[us]
X_Acq_Time          = 2.18365952[s]
X_Angle             = 45[deg]
X_Atn               = 1[dB]
X_Pulse             = 5.55[us]
Irr_Mode            = Off
Tri_Mode            = Off
Dante_Presat        = FALSE
Initial_Wait        = 1[s]
Repetition_Time     = 7.18365952[s]
```

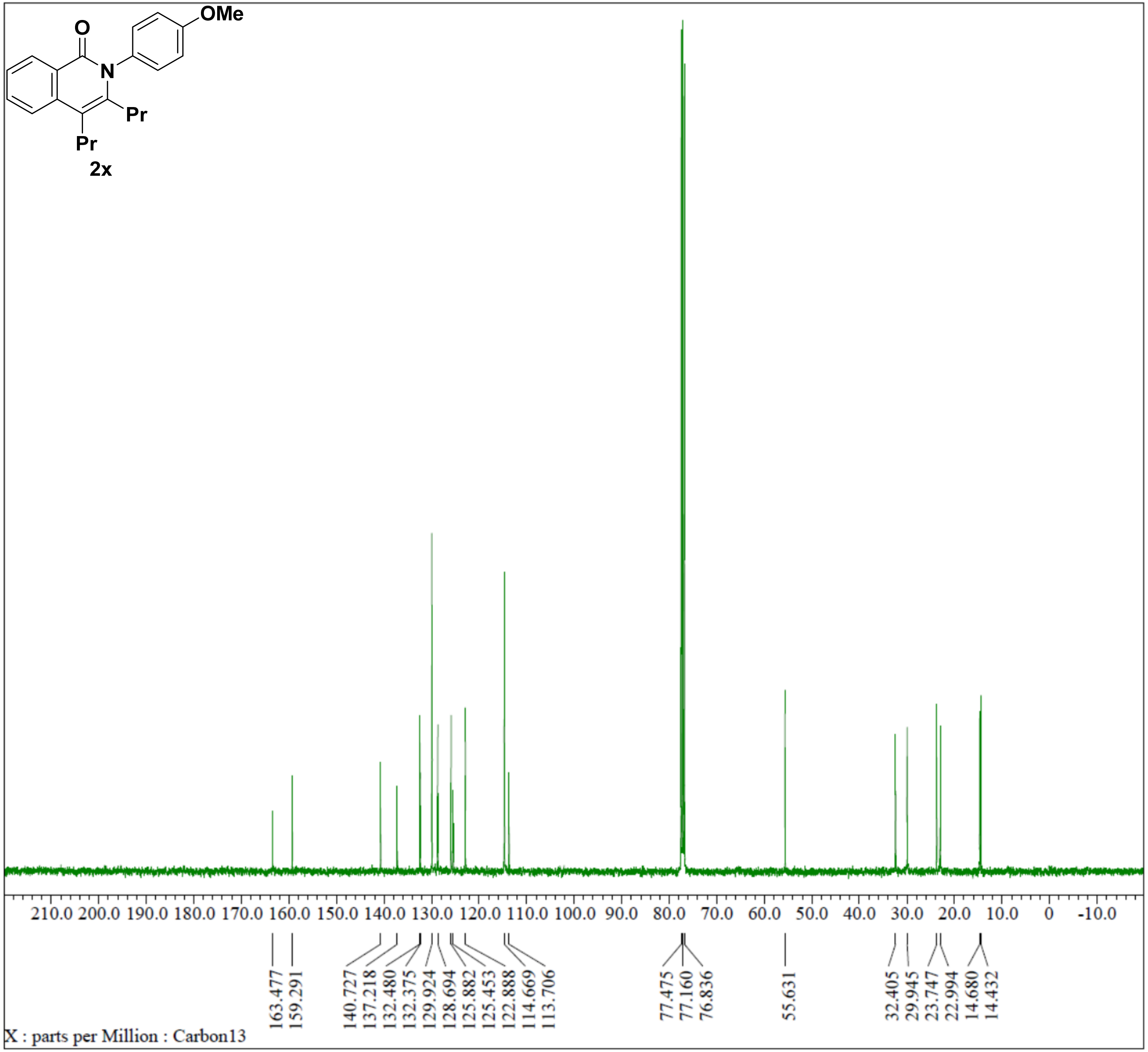


```
Filename            = AO-766 column 33~39_Carbon
Author              = delta
Experiment          = carbon.jxp
Sample_Id           = AO-766 column 33~39
Solvent             = CHLOROFORM-D
Creation_Time       = 14-FEB-2017 05:47:39
Revision_Time       = 14-FEB-2017 09:58:08
Current_Time        = 21-JUN-2017 22:47:49

Comment             = AO-766 column 33~39
Data_Format         = 1D COMPLEX
Dim_Size            = 26214
Dim_Title           = Carbon13
Dim_Units           = [ppm]
Dimensions          = X
Site                = JNM-ECS400
Spectrometer        = DELTA2_NMR

Field_Strength      = 9.389766[T] (400[MHz])
X_Acq_Duration      = 1.04333312[s]
X_Domain            = 13C
X_Freq              = 100.52530333[MHz]
X_Offset            = 100[ppm]
X_Points            = 32768
X_Prescans          = 4
X_Resolution        = 0.95846665[Hz]
X_Sweep             = 31.40703518[kHz]
X_Sweep_Clipped     = 25.12562814[kHz]
Irr_Domain          = Proton
Irr_Freq            = 399.78219838[MHz]
Irr_Offset          = 5[ppm]
Clipped             = FALSE
Scans               = 1024
Total_Scans         = 1024

Relaxation_Delay    = 2[s]
Recvr_Gain          = 60
Temp_Get            = 16.3[dC]
X_90_Width          = 9.9[us]
X_Acq_Time          = 1.04333312[s]
X_Angle             = 30[deg]
X_Atn               = 6[dB]
X_Pulse             = 3.3[us]
Irr_Atn_Dec         = 21.307[dB]
Irr_Atn_Noe         = 21.307[dB]
Irr_Noise           = WALTZ
Irr_Pwidth          = 0.115[ms]
Decoupling          = TRUE
Initial_Wait        = 1[s]
Noe                 = TRUE
Noe_Time            = 2[s]
Repetition_Time     = 3.04333312[s]
```

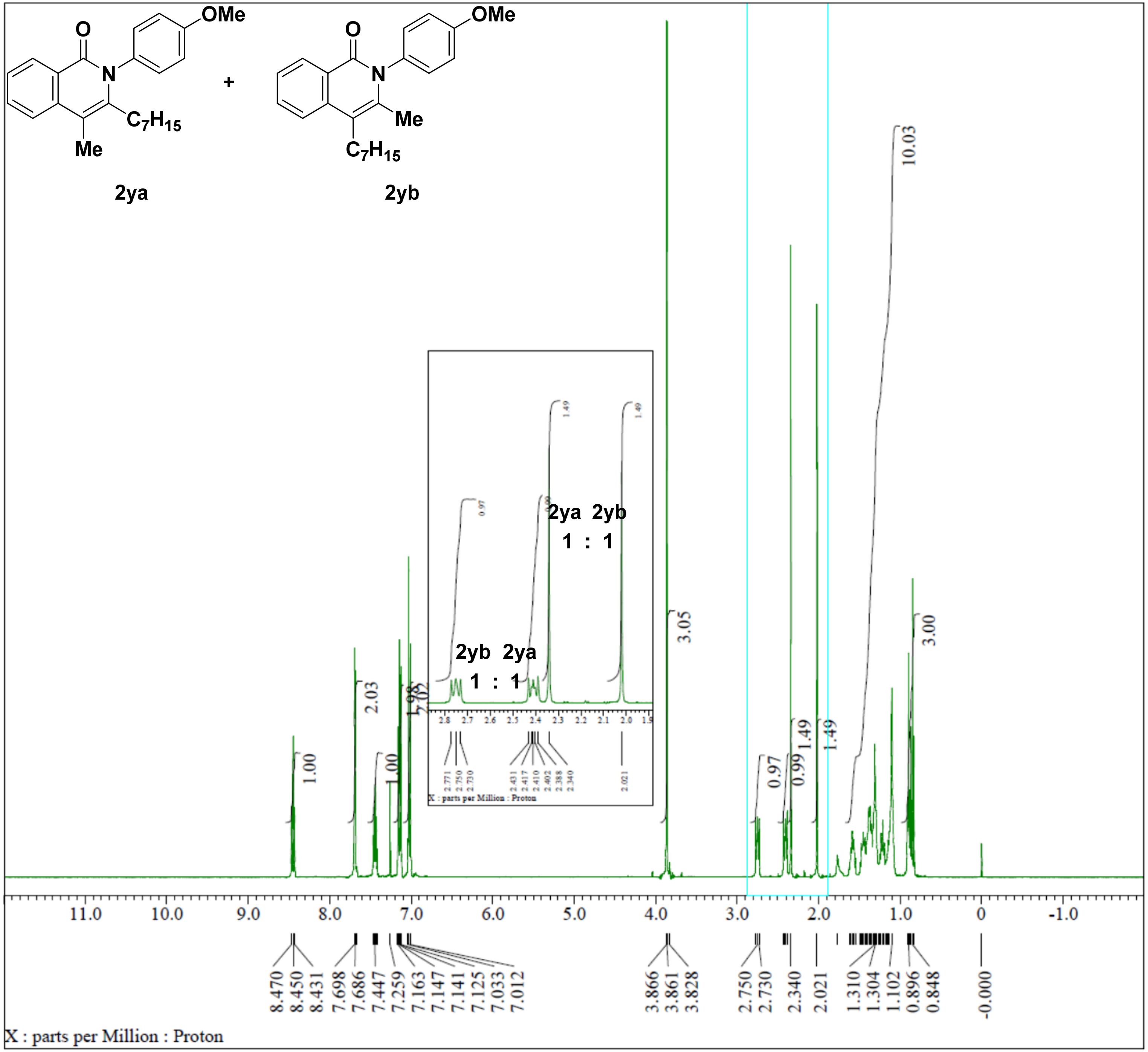


```
Filename          = AO-896 GPC_Proton-1-1.jdf
Author            = delta
Experiment        = proton.jxp
Sample_Id         = AO-896 GPC
Solvent           = CHLOROFORM-D
Creation_Time     = 13-JUN-2017 17:56:16
Revision_Time     = 16-JUN-2017 15:18:16
Current_Time      = 21-JUN-2017 23:02:02

Comment           = AO-896 GPC
Data_Format       = 1D COMPLEX
Dim_Size          = 13107
Dim_Title         = Proton
Dim_Units         = [ppm]
Dimensions        = X
Site              = JNM-ECS400
Spectrometer      = DELTA2_NMR

Field_Strength    = 9.389766[T] (400[MHz])
X_Acq_Duration    = 2.18365952[s]
X_Domain          = 1H
X_Freq            = 399.78219838[MHz]
X_Offset          = 5[ppm]
X_Points          = 16384
X_Prescans        = 1
X_Resolution      = 0.45794685[Hz]
X_Sweep           = 7.5030012[kHz]
X_Sweep_Clipped   = 6.00240096[kHz]
Irr_Domain        = Proton
Irr_Freq          = 399.78219838[MHz]
Irr_Offset        = 5[ppm]
Tri_Domain        = Proton
Tri_Freq          = 399.78219838[MHz]
Tri_Offset        = 5[ppm]
Clipped           = FALSE
Scans             = 8
Total_Scans       = 8

Relaxation_Delay  = 5[s]
Recvr_Gain        = 30
Temp_Get          = 20[dC]
X_90_Width        = 11.1[us]
X_Acq_Time        = 2.18365952[s]
X_Angle           = 45[deg]
X_Atn             = 1[dB]
X_Pulse           = 5.55[us]
Irr_Mode          = Off
Tri_Mode          = Off
Dante_Presat      = FALSE
Initial_Wait      = 1[s]
Repetition_Time   = 7.18365952[s]
```

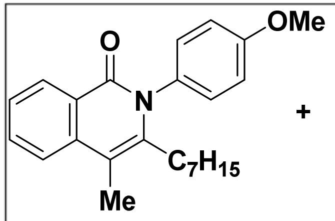

2ya

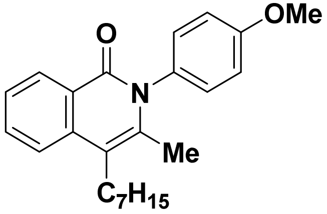

2yb

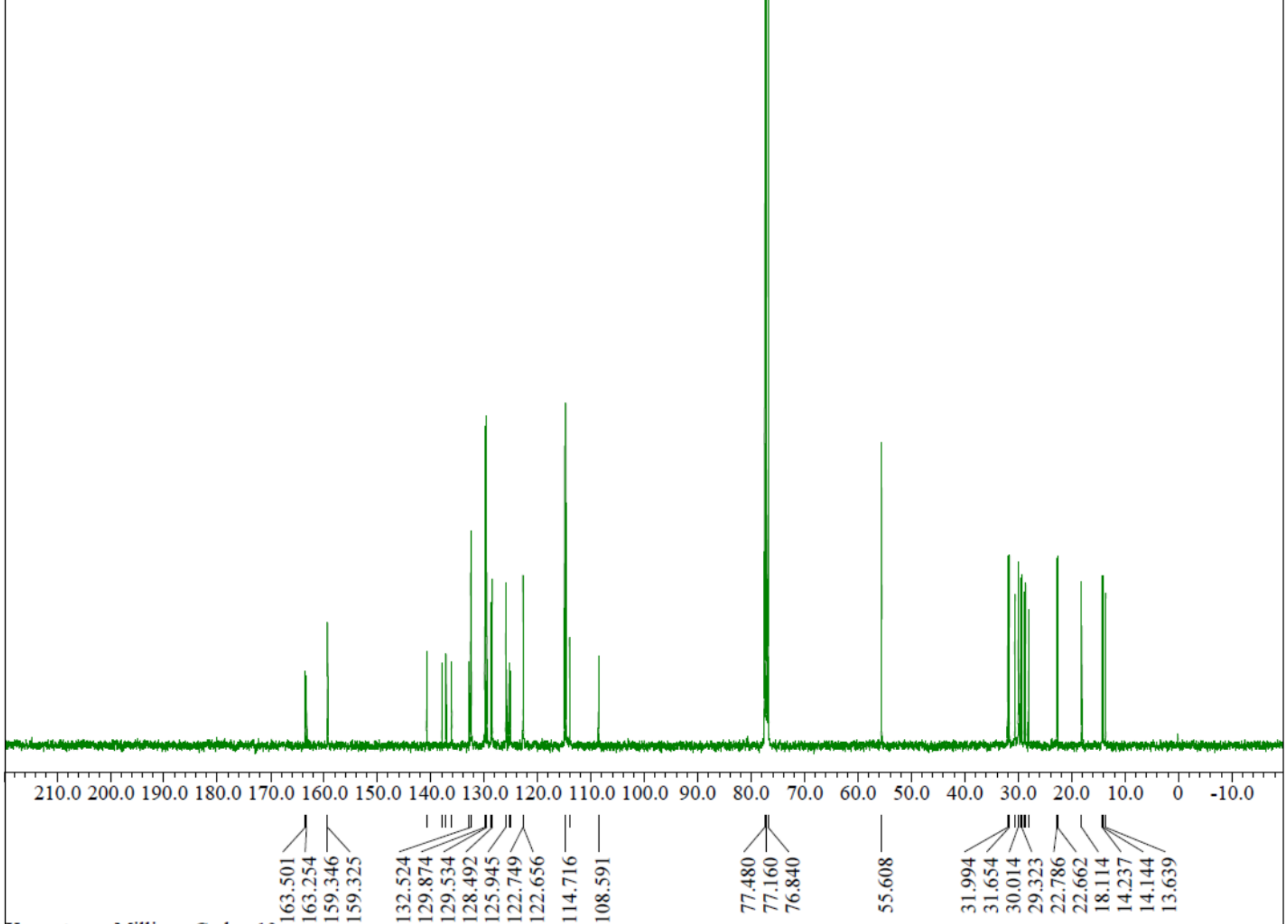


```
Filename          = AO-896 GPC_Carbon-1-1.jdf
Author            = delta
Experiment        = carbon.jxp
Sample_Id         = AO-896 GPC
Solvent           = CHLOROFORM-D
Creation_Time     = 14-JUN-2017 04:06:53
Revision_Time     = 16-JUN-2017 15:18:55
Current_Time      = 21-JUN-2017 23:02:22

Comment           = AO-896 GPC
Data_Format       = 1D COMPLEX
Dim_Size          = 26214
Dim_Title         = Carbon13
Dim_Units         = [ppm]
Dimensions        = X
Site              = JNM-ECS400
Spectrometer      = DELTA2_NMR

Field_Strength    = 9.389766[T] (400[MHz])
X_Acq_Duration    = 0.96468992[s]
X_Domain          = 13C
X_Freq            = 100.52530333[MHz]
X_Offset          = 100[ppm]
X_Points          = 32768
X_Prescans        = 4
X_Resolution      = 1.03660252[Hz]
X_Sweep           = 33.9673913[kHz]
X_Sweep_Clipped   = 27.17391304[kHz]
Irr_Domain        = Proton
Irr_Freq          = 399.78219838[MHz]
Irr_Offset        = 5[ppm]
Clipped           = FALSE
Scans             = 1024
Total_Scans       = 1024

Relaxation_Delay  = 2[s]
Recvr_Gain        = 60
Temp_Get          = 20[dC]
X_90_Width        = 9.9[us]
X_Acq_Time        = 0.96468992[s]
X_Angle           = 30[deg]
X_Atn             = 6[dB]
X_Pulse           = 3.3[us]
Irr_Atn_Dec       = 21.307[dB]
Irr_Atn_Noe       = 21.307[dB]
Irr_Noise         = WALTZ
Irr_Pwidth        = 0.115[ms]
Decoupling        = TRUE
Initial_Wait      = 1[s]
Noe               = TRUE
Noe_Time          = 2[s]
Repetition_Time   = 2.96468992[s]
```

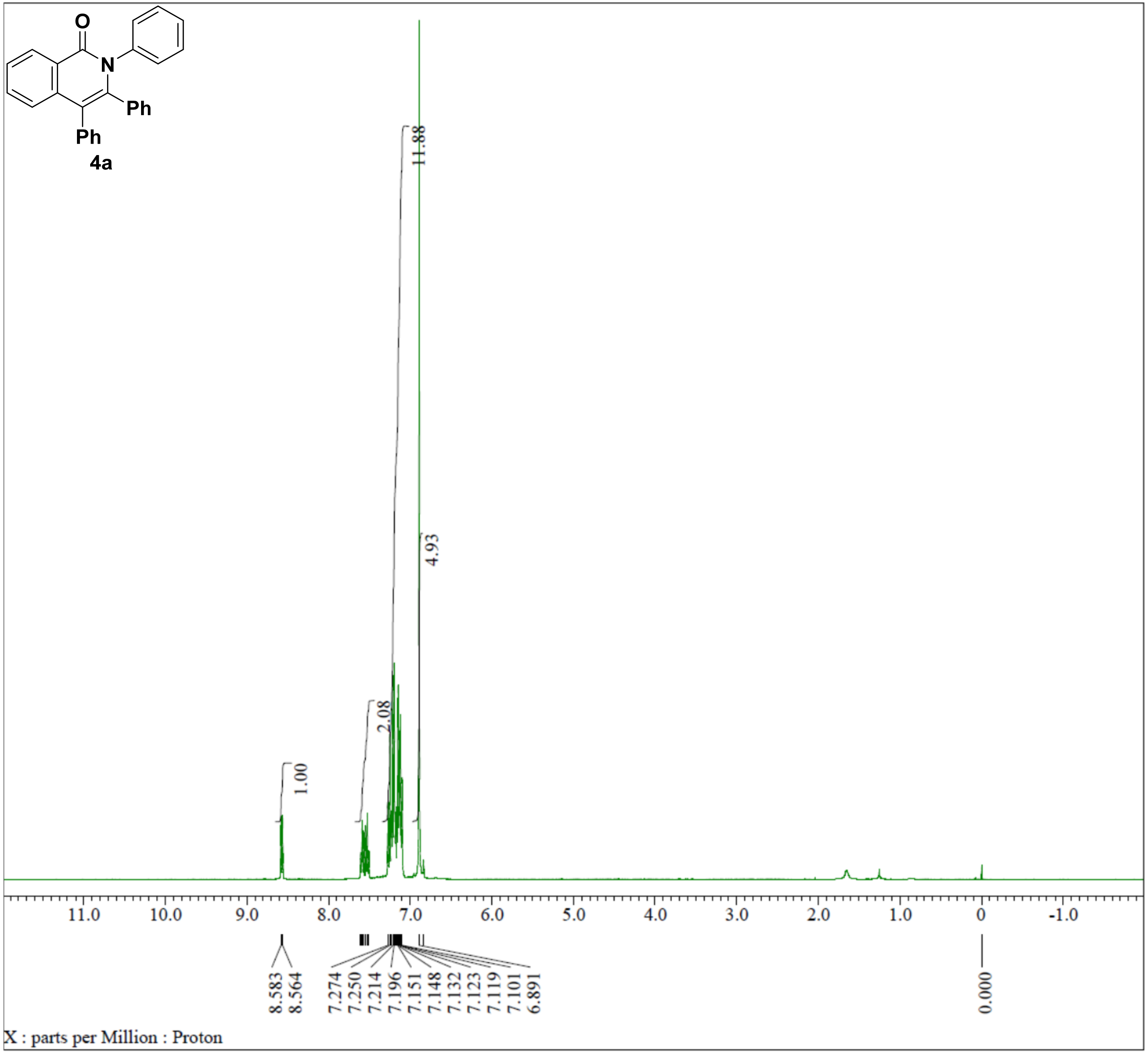


| | |
|---|---|
| Filename | = AO-374_Proton-1-1.jdf |
| Author | = delta |
| Experiment | = proton.jxp |
| Sample_Id | = AO-374 |
| Solvent | = CHLOROFORM-D |
| Creation_Time | = 10-JUN-2017 21:27:13 |
| Revision_Time | = 16-JUN-2017 15:00:03 |
| Current_Time | = 21-JUN-2017 22:55:31 |
| Comment | = AO-374_1H |
| Data_Format | = 1D COMPLEX |
| Dim_Size | = 13107 |
| Dim_Title | = Proton |
| Dim_Units | = [ppm] |
| Dimensions | = X |
| Site | = JNM-ECS400 |
| Spectrometer | = DELTA2_NMR |
| Field_Strength | = 9.389766[T] (400[MHz]) |
| X_Acq_Duration | = 2.18365952[s] |
| X_Domain | = 1H |
| X_Freq | = 399.78219838[MHz] |
| X_Offset | = 5[ppm] |
| X_Points | = 16384 |
| X_Prescans | = 1 |
| X_Resolution | = 0.45794685[Hz] |
| X_Sweep | = 7.5030012[kHz] |
| X_Sweep_Clipped | = 6.00240096[kHz] |
| Irr_Domain | = Proton |
| Irr_Freq | = 399.78219838[MHz] |
| Irr_Offset | = 5[ppm] |
| Tri_Domain | = Proton |
| Tri_Freq | = 399.78219838[MHz] |
| Tri_Offset | = 5[ppm] |
| Clipped | = FALSE |
| Scans | = 8 |
| Total_Scans | = 8 |
| Relaxation_Delay | = 5[s] |
| Recvr_Gain | = 36 |
| Temp_Get | = 20.4[dC] |
| X_90_Width | = 11.1[us] |
| X_Acq_Time | = 2.18365952[s] |
| X_Angle | = 45[deg] |
| X_Atn | = 1[dB] |
| X_Pulse | = 5.55[us] |
| Irr_Mode | = Off |
| Tri_Mode | = Off |
| Dante_Presat | = FALSE |
| Initial_Wait | = 1[s] |
| Repetition_Time | = 7.18365952[s] |

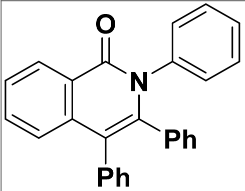

4a

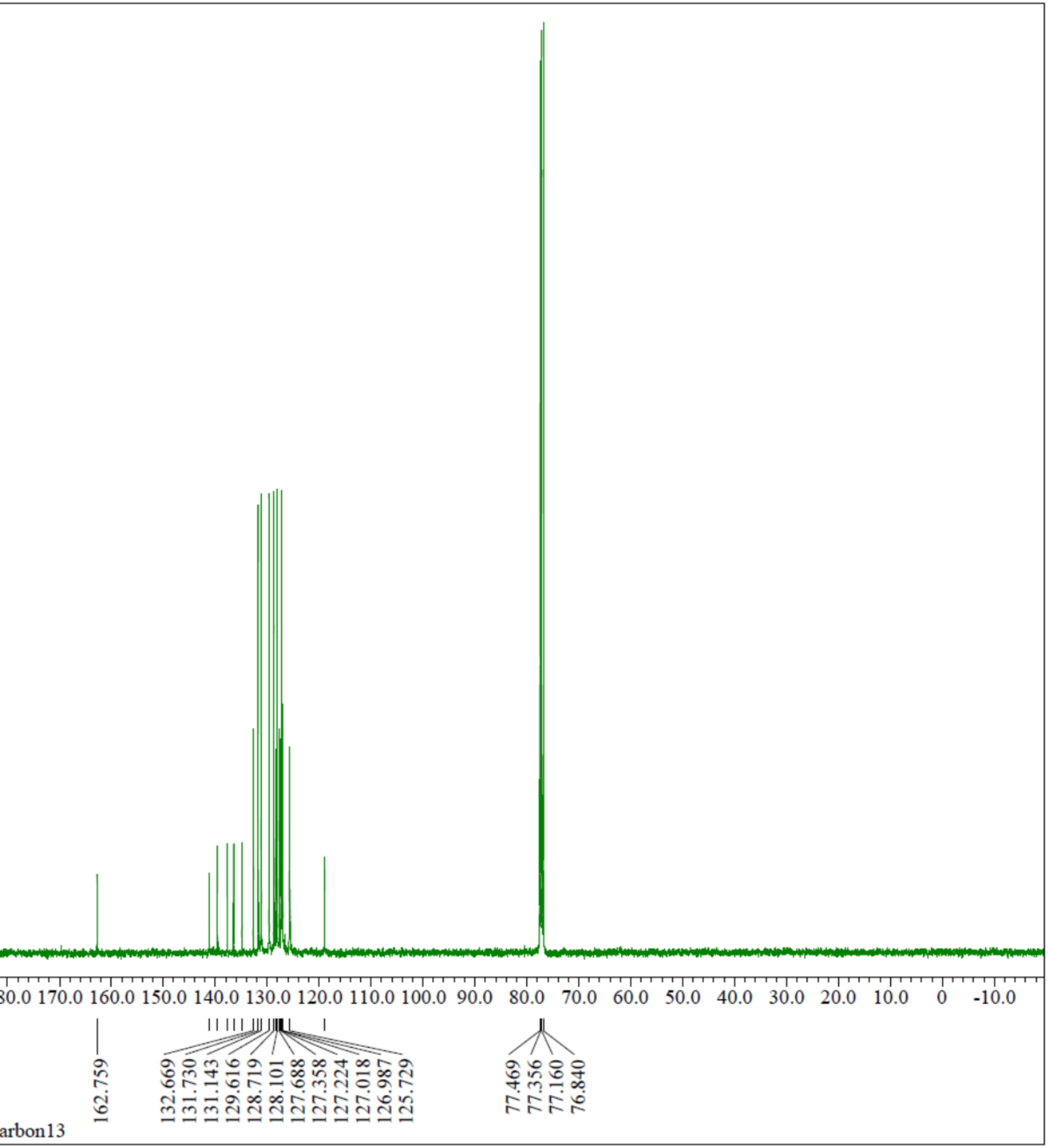


```
Filename          = AO-374 1_Carbon-1-1.jdf
Author            = delta
Experiment        = carbon.jxp
Sample_Id         = AO-374 1
Solvent           = CHLOROFORM-D
Creation_Time     = 12-JUN-2017 05:06:54
Revision_Time     = 16-JUN-2017 15:00:46
Current_Time      = 21-JUN-2017 22:55:53

Comment           = AO-374 1
Data_Format       = 1D COMPLEX
Dim_Size          = 26214
Dim_Title         = Carbon13
Dim_Units         = [ppm]
Dimensions        = X
Site              = JNM-ECS400
Spectrometer      = DELTA2_NMR

Field_Strength    = 9.389766[T] (400[MHz])
X_Acq_Duration    = 0.96468992[s]
X_Domain          = 13C
X_Freq            = 100.52530333[MHz]
X_Offset          = 100[ppm]
X_Points          = 32768
X_Prescans        = 4
X_Resolution      = 1.03660252[Hz]
X_Sweep           = 33.9673913[kHz]
X_Sweep_Clipped   = 27.17391304[kHz]
Irr_Domain        = Proton
Irr_Freq          = 399.78219838[MHz]
Irr_Offset        = 5[ppm]
Clipped           = FALSE
Scans             = 1024
Total_Scans       = 1024

Relaxation_Delay  = 2[s]
Recvr_Gain        = 60
Temp_Get          = 19.5[dC]
X_90_Width        = 9.9[us]
X_Acq_Time        = 0.96468992[s]
X_Angle           = 30[deg]
X_Atn             = 6[dB]
X_Pulse           = 3.3[us]
Irr_Atn_Dec       = 21.307[dB]
Irr_Atn_Noe       = 21.307[dB]
Irr_Noise         = WALTZ
Irr_Pwidth        = 0.115[ms]
Decoupling        = TRUE
Initial_Wait      = 1[s]
Noe               = TRUE
Noe_Time          = 2[s]
Repetition_Time   = 2.96468992[s]
```

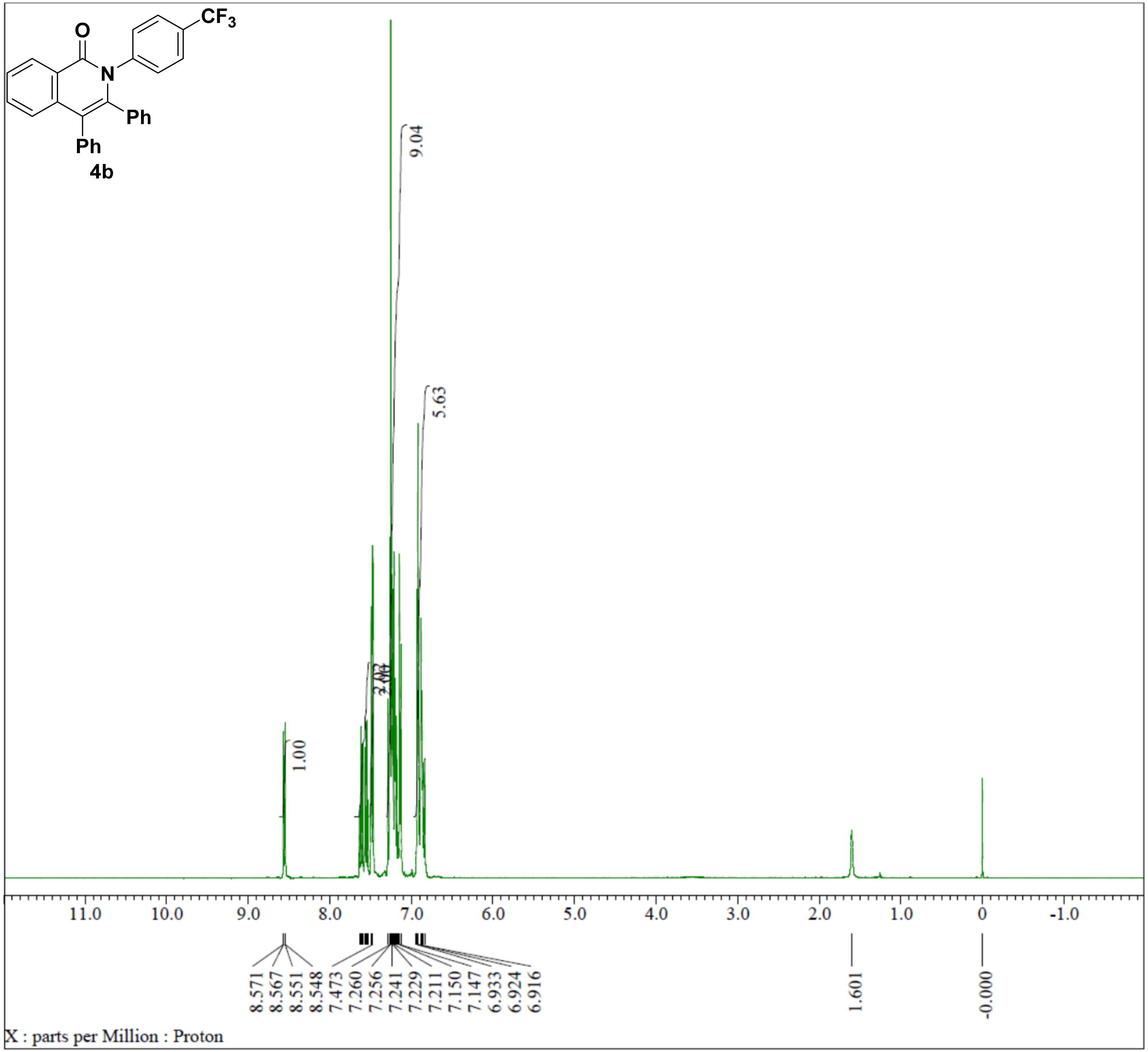


```
Filename          = AO-886 GPC_Proton-1-1.jdf
Author            = delta
Experiment        = proton.jxp
Sample_Id         = AO-886 GPC
Solvent           = CHLOROFORM-D
Creation_Time     =  9-JUN-2017 21:24:06
Revision_Time     = 16-JUN-2017 15:11:47
Current_Time      = 22-JUN-2017 10:19:50

Comment           = AO-886 GPC_1H
Data_Format       = 1D COMPLEX
Dim_Size          = 13107
Dim_Title         = Proton
Dim_Units         = [ppm]
Dimensions        = X
Site              = JNM-ECS400
Spectrometer      = DELTA2_NMR

Field_Strength    = 9.389766[T] (400[MHz])
X_Acq_Duration    = 2.18365952[s]
X_Domain          = 1H
X_Freq            = 399.78219838[MHz]
X_Offset          = 5[ppm]
X_Points          = 16384
X_Prescans        = 1
X_Resolution      = 0.45794685[Hz]
X_Sweep           = 7.5030012[kHz]
X_Sweep_Clipped   = 6.00240096[kHz]
Irr_Domain        = Proton
Irr_Freq          = 399.78219838[MHz]
Irr_Offset        = 5[ppm]
Tri_Domain        = Proton
Tri_Freq          = 399.78219838[MHz]
Tri_Offset        = 5[ppm]
Clipped           = FALSE
Scans             = 8
Total_Scans       = 8

Relaxation_Delay  = 5[s]
Recvr_Gain        = 40
Temp_Get          = 20.5[dC]
X_90_Width        = 11.1[us]
X_Acq_Time        = 2.18365952[s]
X_Angle           = 45[deg]
X_Atn             = 1[dB]
X_Pulse           = 5.55[us]
Irr_Mode          = Off
Tri_Mode          = Off
Dante_Presat      = FALSE
Initial_Wait      = 1[s]
Repetition_Time   = 7.18365952[s]
```

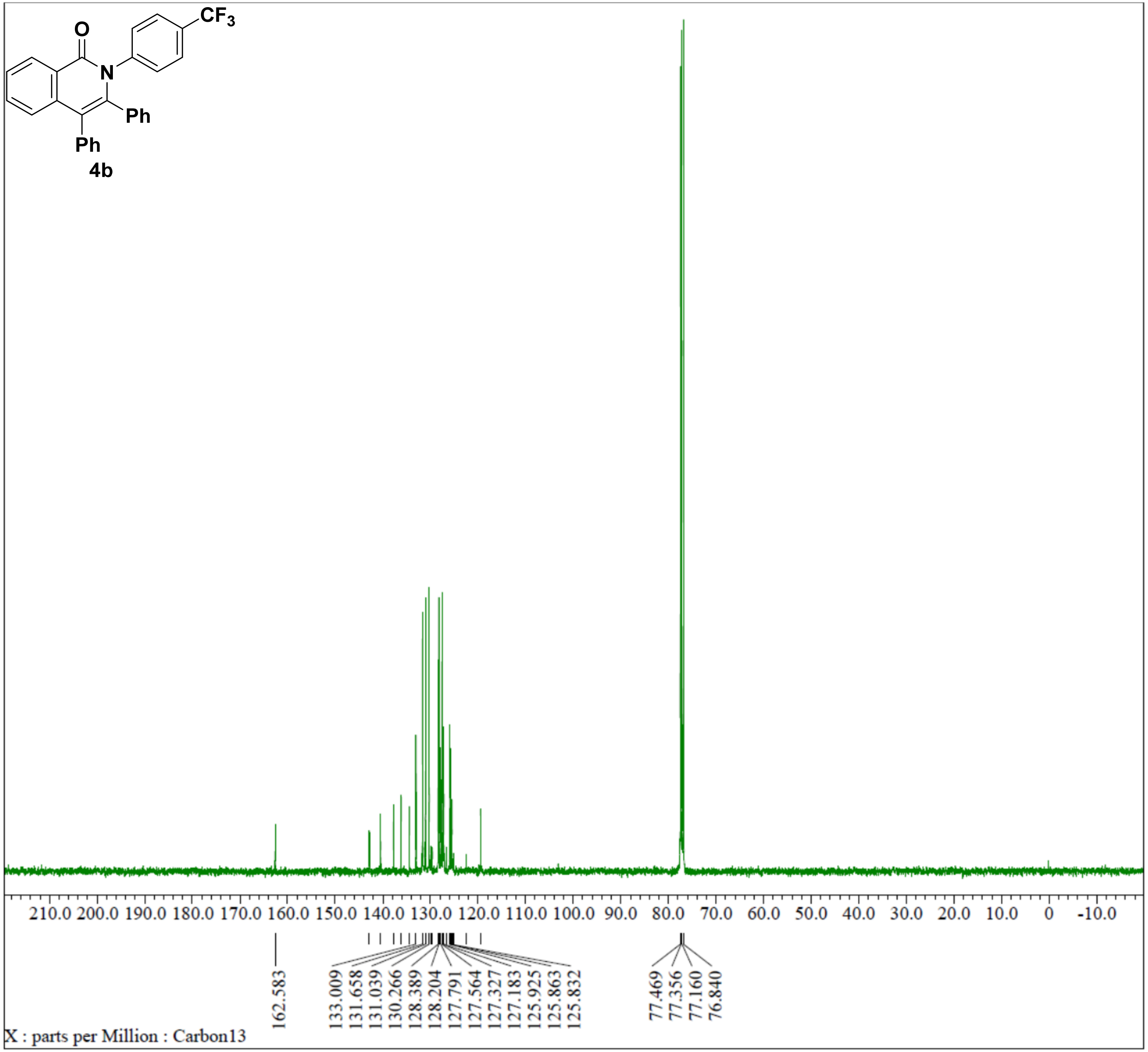


Filename = AO-886 GPC_Carbon-1-1.jdf
Author = delta
Experiment = carbon.jxp
Sample_Id = AO-886 GPC
Solvent = CHLOROFORM-D
Creation_Time = 10-JUN-2017 02:04:59
Revision_Time = 16-JUN-2017 15:10:07
Current_Time = 22-JUN-2017 10:20:18

Comment = AO-886 GPC
Data_Format = 1D COMPLEX
Dim_Size = 26214
Dim_Title = Carbon13
Dim_Units = [ppm]
Dimensions = X
Site = JNM-ECS400
Spectrometer = DELTA2_NMR

Field_Strength = 9.389766[T] (400[MHz])
X_Acq_Duration = 0.96468992[s]
X_Domain = 13C
X_Freq = 100.52530333[MHz]
X_Offset = 100[ppm]
X_Points = 32768
X_Prescans = 4
X_Resolution = 1.03660252[Hz]
X_Sweep = 33.9673913[kHz]
X_Sweep_Clipped = 27.17391304[kHz]
Irr_Domain = Proton
Irr_Freq = 399.78219838[MHz]
Irr_Offset = 5[ppm]
Clipped = FALSE
Scans = 1024
Total_Scans = 1024

Relaxation_Delay = 2[s]
Recvr_Gain = 60
Temp_Get = 20.7[dC]
X_90_Width = 9.9[us]
X_Acq_Time = 0.96468992[s]
X_Angle = 30[deg]
X_Atn = 6[dB]
X_Pulse = 3.3[us]
Irr_Atn_Dec = 21.307[dB]
Irr_Atn_Noe = 21.307[dB]
Irr_Noise = WALTZ
Irr_Pwidth = 0.115[ms]
Decoupling = TRUE
Initial_Wait = 1[s]
Noe = TRUE
Noe_Time = 2[s]
Repetition_Time = 2.96468992[s]

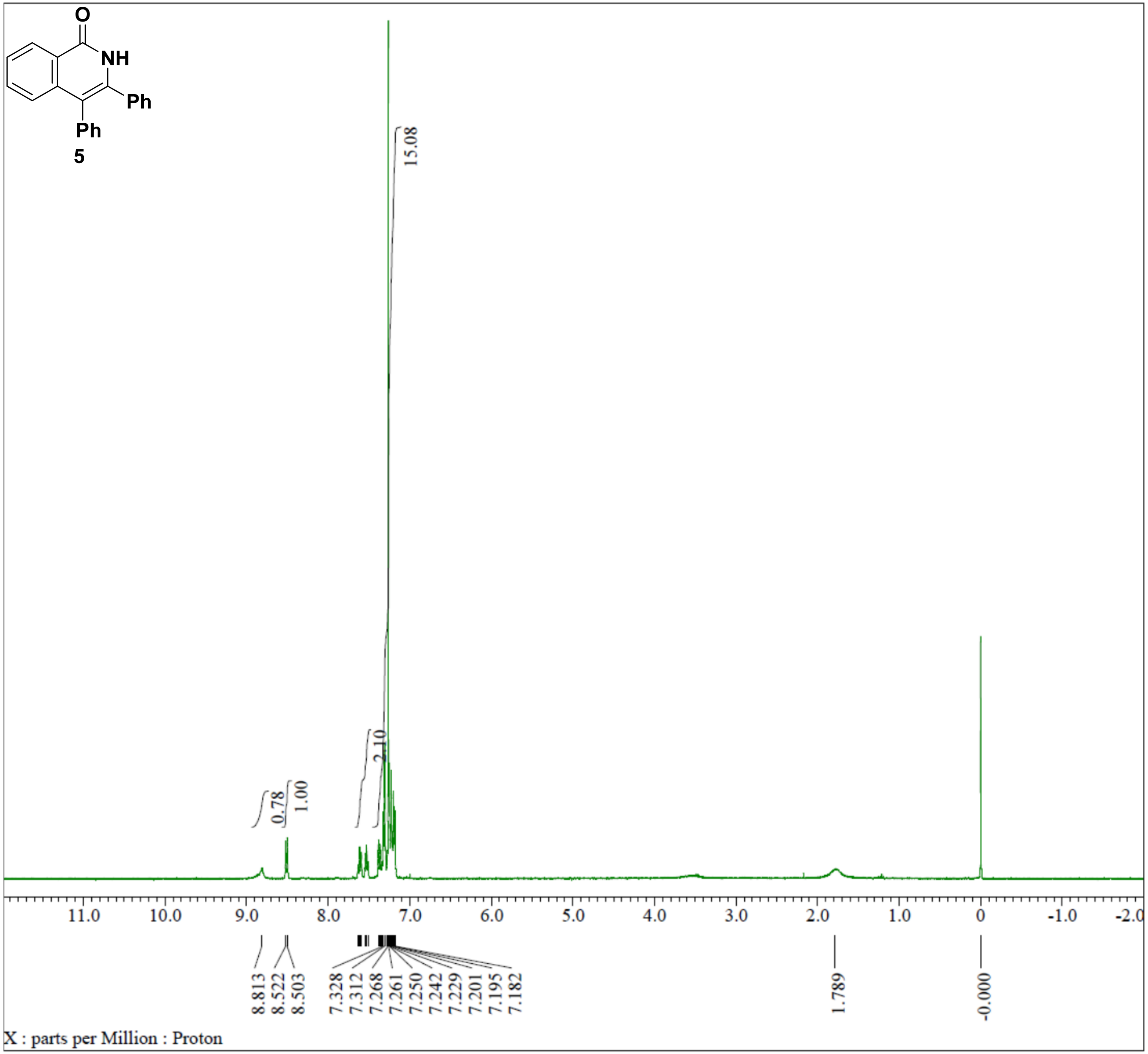


```
Filename          = PMP-removal_Proton-1-4.jdf
Author            = delta
Experiment        = proton.jxp
Sample_Id         = PMP removal 1
Solvent           = CHLOROFORM-D
Creation_Time     = 17-JUN-2017 15:15:22
Revision_Time     = 21-JUN-2017 23:06:46
Current_Time      = 21-JUN-2017 23:07:12

Comment           = PMP removal 1
Data_Format       = 1D COMPLEX
Dim_Size          = 13107
Dim_Title         = Proton
Dim_Units         = [ppm]
Dimensions        = X
Site              = JNM-ECS400
Spectrometer      = DELTA2_NMR

Field_Strength    = 9.389766[T] (400[MHz])
X_Acq_Duration    = 2.18365952[s]
X_Domain          = 1H
X_Freq            = 399.78219838[MHz]
X_Offset          = 5[ppm]
X_Points          = 16384
X_Prescans        = 1
X_Resolution      = 0.45794685[Hz]
X_Sweep           = 7.5030012[kHz]
X_Sweep_Clipped   = 6.00240096[kHz]
Irr_Domain        = Proton
Irr_Freq          = 399.78219838[MHz]
Irr_Offset        = 5[ppm]
Tri_Domain        = Proton
Tri_Freq          = 399.78219838[MHz]
Tri_Offset        = 5[ppm]
Clipped           = FALSE
Scans             = 8
Total_Scans       = 8

Relaxation_Delay  = 5[s]
Recvr_Gain        = 54
Temp_Get          = 20.4[dC]
X_90_Width        = 11.1[us]
X_Acq_Time        = 2.18365952[s]
X_Angle           = 45[deg]
X_Atn             = 1[dB]
X_Pulse           = 5.55[us]
Irr_Mode          = Off
Tri_Mode          = Off
Dante_Presat      = FALSE
Initial_Wait      = 1[s]
Repetition_Time   = 7.18365952[s]
```

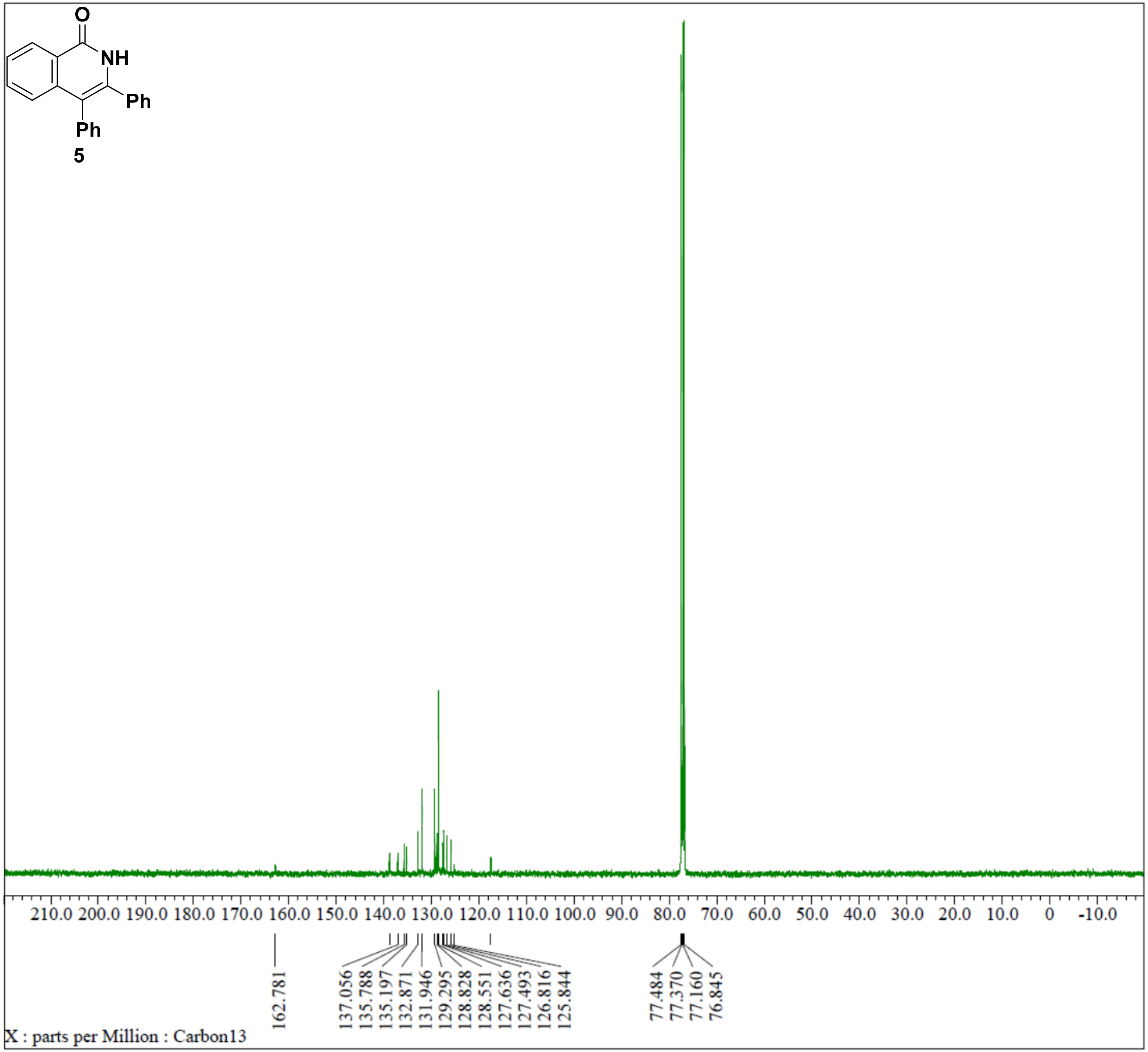


| | |
|---|---|
| Filename | = PMP-removal_Carbon-1-3.jdf |
| Author | = delta |
| Experiment | = carbon.jxp |
| Sample_Id | = PMP-removal |
| Solvent | = CHLOROFORM-D |
| Creation_Time | = 16-JUN-2017 03:06:30 |
| Revision_Time | = 21-JUN-2017 23:05:16 |
| Current_Time | = 21-JUN-2017 23:07:40 |
| Comment | = PMP-removal_13C |
| Data_Format | = 1D COMPLEX |
| Dim_Size | = 26214 |
| Dim_Title | = Carbon13 |
| Dim_Units | = [ppm] |
| Dimensions | = X |
| Site | = JNM-ECS400 |
| Spectrometer | = DELTA2_NMR |
| Field_Strength | = 9.389766[T] (400[MHz]) |
| X_Acq_Duration | = 1.04333312[s] |
| X_Domain | = 13C |
| X_Freq | = 100.52530333[MHz] |
| X_Offset | = 100[ppm] |
| X_Points | = 32768 |
| X_Prescans | = 4 |
| X_Resolution | = 0.95846665[Hz] |
| X_Sweep | = 31.40703518[kHz] |
| X_Sweep_Clipped | = 25.12562814[kHz] |
| Irr_Domain | = Proton |
| Irr_Freq | = 399.78219838[MHz] |
| Irr_Offset | = 5[ppm] |
| Clipped | = TRUE |
| Scans | = 2048 |
| Total_Scans | = 2048 |
| Relaxation_Delay | = 2[s] |
| Recvr_Gain | = 60 |
| Temp_Get | = 20.3[dC] |
| X_90_Width | = 9.9[us] |
| X_Acq_Time | = 1.04333312[s] |
| X_Angle | = 30[deg] |
| X_Atn | = 6[dB] |
| X_Pulse | = 3.3[us] |
| Irr_Atn_Dec | = 21.307[dB] |
| Irr_Noise | = WALTZ |
| Irr_Pwidth | = 0.115[ms] |
| Decoupling | = TRUE |
| Initial_Wait | = 1[s] |
| Noe | = FALSE |
| Repetition_Time | = 3.04333312[s] |
